# A Potent Oral Sialylation Inhibitor Augments the Immunotherapy in Pancreatic Ductal Adenocarcinoma

**DOI:** 10.1021/acscentsci.5c00939

**Published:** 2025-09-03

**Authors:** Jiahui Mou, Runqiu Chen, Zihao Dai, Hao Yang, Feiyashan Suo, Yifan Li, Yangxu Ye, Pengfei Fang, Fang Bai, Yachen Zhao, Rong Zhang, Yiru Long, Likun Gong, Jing Wang, Biao Yu

**Affiliations:** † State Key Laboratory of Chemical Biology, 58309 Shanghai Institute of Organic Chemistry, Chinese Academy of Sciences, Shanghai 200032, China; ‡ Shanghai Institute of Infectious Diseases and Biosecurity, 12478 Fudan University, Shanghai 200032, China; § State Key Laboratory of Drug Research, Shanghai Institute of Materia Medica, Chinese Academy of Sciences, Shanghai 201203, China; ∥ School of Chemistry and Materials Science, Hangzhou Institute for Advanced Study, 638898 University of Chinese Academy of Sciences, Hangzhou 310024, China; ⊥ School of Life Science and Technology, and Shanghai Institute for Advanced Immunochemical Studies, 387433 ShanghaiTech University, Shanghai 201210, China; # School of Public Health, China Medical University, Shenyang 110122, China

## Abstract

Pancreatic ductal adenocarcinoma (PDAC) remains refractory to current immune checkpoint blockade (ICB) therapies, necessitating innovative therapeutic strategies. Emerging evidence implicates aberrant sialoglycan upregulation as a key mediator of immune evasion in PDAC. Herein, we report Y-320, a highly potent oral sialylation inhibitor discovered through high-throughput screening. Y-320 suppresses α-2,3/2,6-sialylation in PDAC cells (IC_50_ ≈ 200 nM) with >300-fold higher activity than the known pan-inhibitor P-3F_ax_-Neu5Ac. Structural analyses reveal competitive occupation of multiple sialyltransferases’ substrate-binding pockets as Y-320’s action mechanism. *In vivo*, Y-320 significantly inhibits tumor growth and remodels the tumor immune microenvironment. Mechanistic studies establish that the therapeutic efficacy of Y-320 depends on the coordinated engagement between CD8^+^ T cell and macrophage. Importantly, Y-320 synergizes with anti-PD-1 therapy to overcome ICB resistance in PDAC, demonstrating superior tumor suppression compared to monotherapies. Our findings demonstrate that Y-320 shows promise for use as a therapeutic agent for cancer and validates sialylation inhibition as a novel glycoimmune checkpoint strategy for PDAC and other immunotherapy-resistant malignancies.

## Graphical abstract

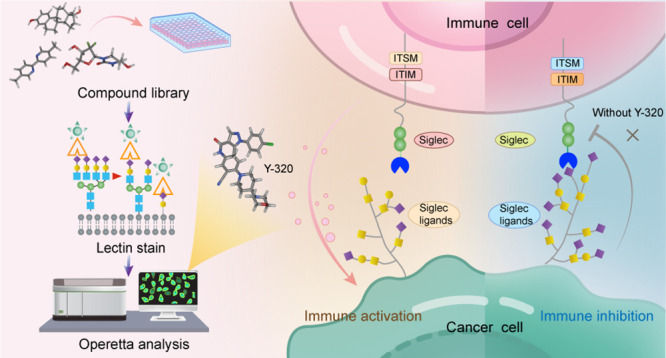

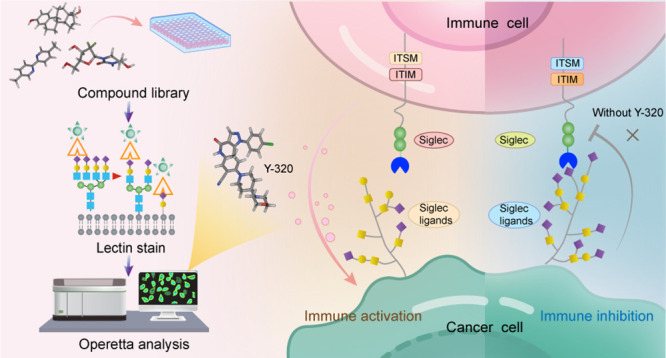

## Introduction

Sialylation, a critical subset of glycosylation, involves the attachment of sialic acid—a nine-carbon monosaccharide—to terminal positions of glycans. Aberrant sialylation is a hallmark of cancer, with tumor surfaces exhibiting ≥ 40% higher sialic acid levels than normal tissues.^1–3^ Since the 1970s, studies have linked hypersialylation to tumor progression, metastasis, and immune evasion,^4–8^ making hypersialylation a broad-spectrum clinical biomarker for malignancies.^9^ These findings collectively establish sialylation as a key regulator of cancer pathogenesis.

Among cancers, pancreatic ductal adenocarcinoma (PDAC) exemplifies the clinical urgency of targeting sialylation. PDAC is one of the most lethal malignancies, with a dismal 5-year survival rate of only 9%. Overexpression of sialylated structures such as sLeX and sTn in PDAC correlates with poor prognosis, while CA19–9 (sLeA), a sialylation-dependent biomarker, is routinely used for PDAC diagnosis and monitoring.^10,11^

Sialylation drives oncogenesis by dysregulating cellular recognition, adhesion, and signaling,^9,12^ thereby promoting tumor extravasation, angiogenesis, apoptotic resistance, and drug tolerance.^5–7^ Particularly, as a mammalian-specific “self” signal, tumor-associated sialic acids engage sialic acid-binding immunoglobulin-type lectins (Siglecs) on immune cells to initiate immunosuppressive mechanisms, including steric masking of surface antigens to evade immune detection and activation of inhibitory Siglec signaling to suppress cytotoxic responses from CD8^+^ T cells, NK cells, and macrophages.^13–16^ Therapeutic interventions that disrupt sialylation (e.g., with sialidase conjugates) could reverse these effects to restoring immune-mediated tumor killing,^17–19^ further positioning sialylation as a key mediator of tumor progression and thus a promising target for cancer immunotherapy.

Central to sialylation regulation is the dynamic balance between sialidases (which remove terminal sialic acids) and sialyltransferases (STs) (which catalyze sialylation of glycans).^20^ Critically, the pathological overexpression of STs has been shown to be the principal driver of tumor-associated hypersialylation,^21^ prompting intensive research into developing ST-targeted inhibitors for potential anticancer therapeutics.^22–27^

However, targeting individual STs poses a fundamental biological challenge. The human genome encodes 20 ST genes organized into four subfamilies based on their linkage specificity, including (i) ST3Gal (α2–3 linkage; Sia→Gal), (ii) ST6Gal (α2–6 linkage; Sia→Gal), (iii) ST6GalNAc (α2–6 linkages; Sia→GalNAc/GlcNAc), and (iv) ST8Sia (α2–8 linkages; Sia→Sia).^28^ Members of each subfamily exhibit remarkable functional redundancy. For example, the ST3Gal subfamily (ST3Gal1–6) members catalyze the formation of α2–3 linkages with compensatory enzymatic capabilities; the ST6Gal subfamily (ST6Gal1–2) enzymes mediate the formation of α2–6 linkages and demonstrate cooperative overexpression in tumors. This robust functional overlap creates a biological “whack-a-mole” phenomenon—selectively inhibiting a single sialyltransferase often triggers compensatory upregulation of others.^10,28,29^

This compensatory mechanism necessitates the development of pan-sialylation inhibitors. Indeed, the current leading ST inhibitor, P-3F_ax_-Neu5Ac, functions as a pan-sialyltransferase inhibitor. This compound’s *O*-acetyl modifications enable its cellular permeability, after which intracellular esterases cleave these groups to reveal the active metabolite—a structural analogue of the natural donor substrate CMP-Neu5Ac, achieving cellular activity (IC_50_ = 26.8 ± 5.7 μM) despite being inactive *in vitro*.^30^ While representing a critical proof-of-concept for pan-inhibition, P-3F_ax_-Neu5Ac’s moderate potency highlights two key unmet needs: (1) broad-spectrum inhibition to overcome enzymatic redundancy and (2) improved pharmacological properties for clinical translation.

To address these challenges, our study employs a new approach to pan-ST inhibition. We developed a high-throughput phenotypic screening platform to identify pan-sialylation inhibitors from a curated library of 1,729 compounds comprising FDA-approved drugs and clinical trial candidates. Compound Y-320 was identified to inhibit the α-2,3SA and α-2,6SA levels with IC_50_s around 150–240 nM in multiple PDAC cells, approximately 300- to 500-fold greater potency than the positive control P-3F_ax_-Neu5Ac. Mechanistic studies revealed Y-320 could directly bind and inhibit key sialyltransferases in PDAC (including ST3Gal1, ST3Gal2, and ST6GalNAc2) to significantly reduce tumor-specific sialic acid levels both *in vitro* and *in vivo* and enhance antitumor immunity.

## Results

### High-Throughput Screening Identifies Y-320 as a Sialylation Inhibitor in PDAC Cells

To explore efficient sialylation inhibitors with favorable membrane permeability, we developed a Cell-based Phenotypic Screening for Sialylation Inhibitors (CPS-SI) assay. This assay employs lectins to quantitatively assess changes in the cell surface sialylation levels following chemical treatment (). Given that PDAC is characterized by elevated levels of α-2,3- and α-2,6-linked sialylation, which are closely associated with immune escape,^10^ we selected the human PDAC cell line PANC-1 as the screening model. We used lectin Maackia Amurensis Lectin II (MALII), which preferentially recognizes α-2,3-linked sialic acid (α-2,3SA) on glycans (Figure 1A, ), to quantitatively measure the levels of PANC-1 cell surface α-2,3-linked sialylation before and after drug treatment. In this system, P-3F_ax_-Neu5Ac was employed as a positive control due to its well-documented inhibitory effect on sialylation.^31,32^ A library of 1729 compounds, including FDA-approved drugs and those in clinical trials, was screened in this study (Figure 1B). The assay demonstrated robust performance, with a signal-to-background ratio of 28.59 and a Z-factor of 0.51 (), confirming its reliability for high-throughput screening. Since cell state significantly affects staining outcomes, to minimize confounding effects, compounds causing severe cytotoxicity (cell viability <10%) were excluded from further analysis. To further validate the screening results, two independent replicate experiments were performed on the initial hits. Among these, 29 compounds were identified, each exhibiting sialylation inhibition rates exceeding 25% at a concentration of 10 μM ().

**Figure 1:**
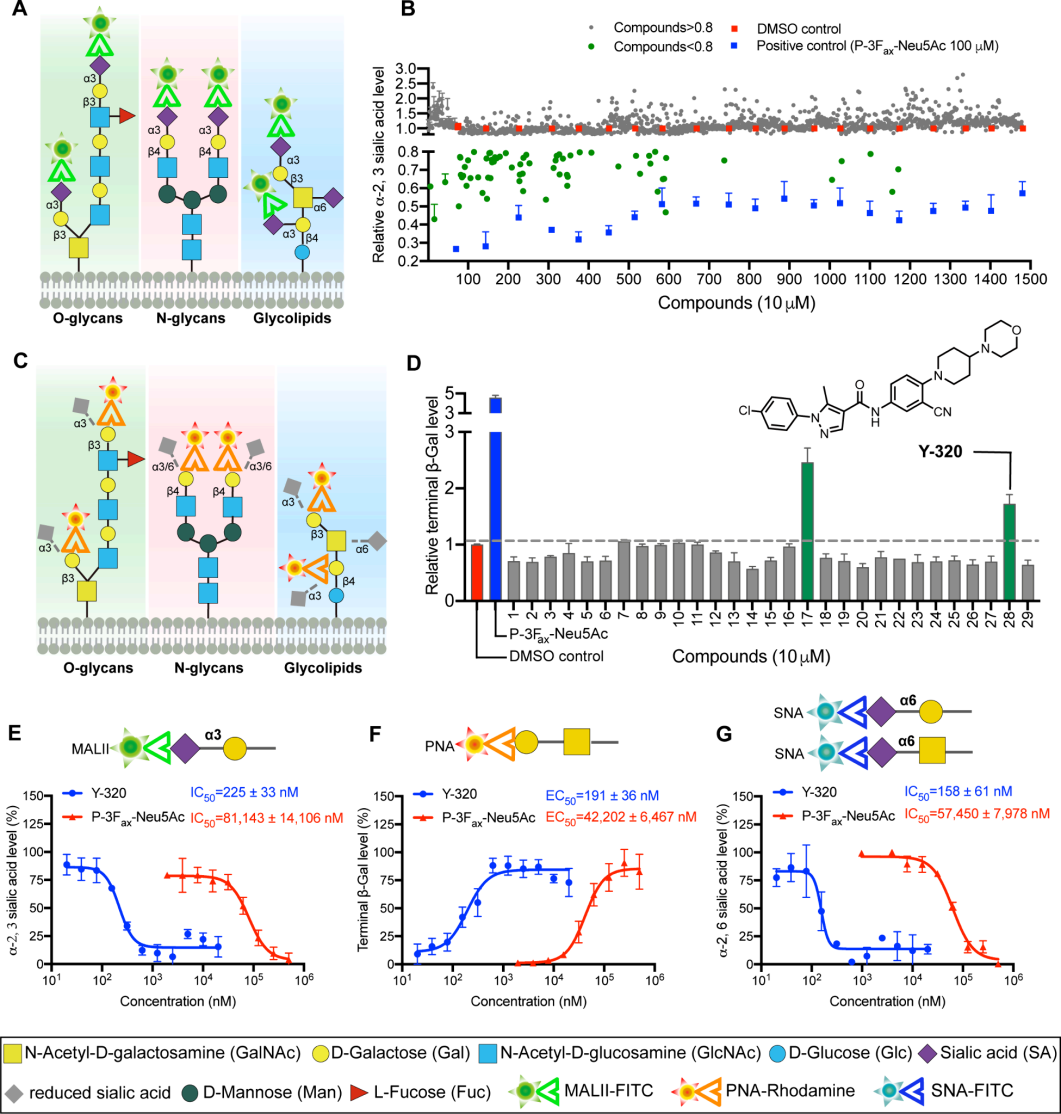
High-throughput screening identifies Y-320 as a potential sialylation inhibitor. (A) Glycan structures recognized by lectin MALII. (B) Primary screening: relative α-2,3 sialic acid level in PANC-1 cells measured 72 h post-treatment with library drugs at a final drug concentration of 10 μM. Mean ± SD are provided. (C) Glycan structures recognized by lectin PNA and the schematic diagram of the connection between sialic acid and β-galactose (β-gal). (D) Secondary screening: relative terminal β-gal level in PANC-1 cells measured 72 h after treatment with drugs at a final concentration of 10 μM. Compounds increasing terminal β-gal levels are highlighted in green. Insets: chemical structure of Y-320. Result is the average of three independent experiments and mean ± SD are provided. (E-G) Top: glycan structure recognized by MALII (E), PNA (F) and SNA (G). Bottom: a plot showing the percentage of α-2,3SA (E), terminal β-gal (F) and α-2,6SA (G) level in PANC-1 cells treated with escalating doses of Y-320 or P-3F_ax_-Neu5Ac. Activity curves were normalized relative to the maximum and minimum measured values (100% and 0%, respectively). Result is the average of three independent experiments and mean ± SD are provided.

Notably, these 29 compounds could modulate α2,3-SA levels through diverse mechanisms—either by directly altering terminal sialic acid or indirectly affecting upstream glycan structures. To distinguish direct sialylation inhibitors, we designed a secondary assay detecting terminal galactose (β-Gal) exposure, a hallmark of sialic acid addition blockade (Figure 1C). Staining with lectin Peanut Agglutinin (PNA), which binds preferentially to exposed galactose upon desialylation, we identified two compounds that significantly increased β-Gal levels, confirming their direct inhibition of sialylation (Figure 1D). Among these, Y-320 was prioritized for further investigation due to its superior stability and synthetic accessibility.

Using MALII staining to monitor α-2,3SA levels, we observed that Y-320 significantly reduced α-2,3SA levels in PANC-1 human pancreatic cancer cells, with an IC_50_ of 225 ± 33 nM, demonstrating approximately 360-fold greater potency than the positive control P-3F_ax_-Neu5Ac (IC_50_ = 81,143 ± 14,106 nM) (Figure 1E, ). Consistent results were observed in MIA PaCa-2 cells, where Y-320 inhibited α-2,3SA levels with an IC_50_ of 235 ± 46 nM, similar to its activity in PANC-1 cells (). In addition, we observed a dose-dependent increase in terminal β-Gal levels following Y-320 treatment with an EC_50_ of 191 ± 36 nM in PANC-1 and an EC_50_ of 215 ± 66 nM in MIA PaCa-2 (Figure 1F, , and ). Notably, P-3F_ax_-Neu5Ac treatment exhibited an EC_50_ of 42,202 ± 6,467 nM in PANC-1 and an EC_50_ of 87,737 ± 10,302 nM in MIA PaCa-2 (Figure 1F, , ). Collectively, these findings demonstrate that Y-320 significantly inhibits the formation of α-2,3SA in PDAC cells with the most potent efficacy at the cellular level currently known.

Next, we assessed the effect of Y-320 on the formation of α-2,6-linked sialic acid (α-2,6SA) using *Sambucus nigra* lectin (SNA), which preferentially recognizes α-2,6SA (). We observed that Y-320 inhibited α-2,6SA formation with an IC_50_ of 158 ± 61 nM in PANC-1 cells and 215 ± 66 nM in MIA PaCa-2 cells. Of note, the inhibitory efficiency of Y-320 was 300 to 500-fold higher than that of P-3F_ax_-Neu5Ac (Figure 1G, , ).

Together, these results indicate that Y-320 may act as a pan-inhibitor suppressing both α-2,3-linked and α-2,6-linked sialylation, suggesting its potent and broad-spectrum sialylation inhibitory activity.

### Y-320 Inhibits the Activity of Sialyltransferases

To elucidate the mechanism by which Y-320 inhibits sialylation, we focused on the four major protein classes involved in this process: sialyltransferases, sialidases, donor synthases, and transporters. First, we verified the expression regulation of these proteins by Y-320. The results showed that Y-320 had no significant effect on the mRNA levels of these proteins (Figure 2A). Similarly, immunoblot analysis of selected sialyltransferases showed no notable alterations in protein expression (). These results indicate that Y-320 does not influence sialylation by modulating enzyme expression but likely through post-translational regulation of their activity, affecting sialic acid transfer, synthesis, or transport.

**Figure 2:**
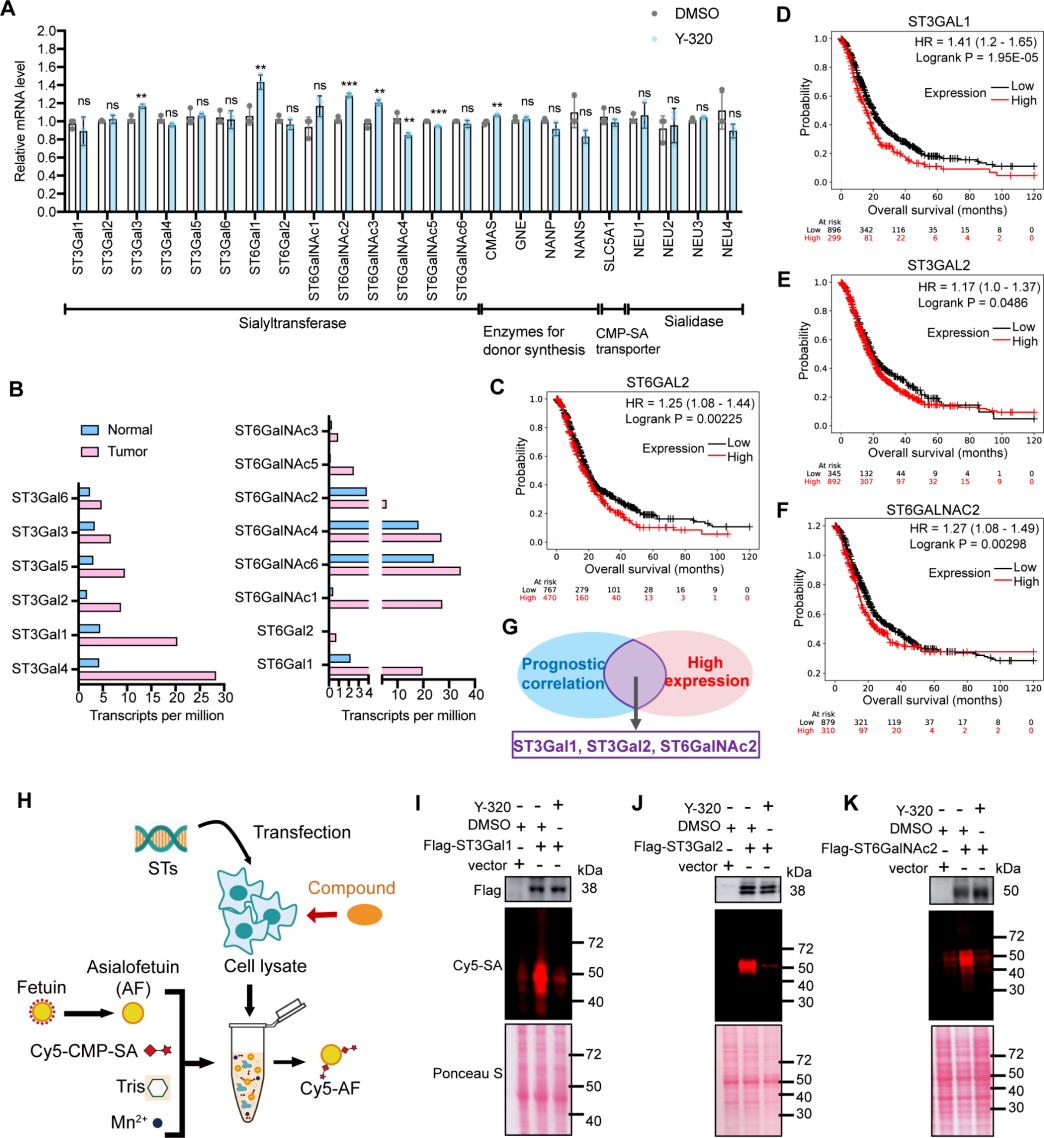
Y-320 inhibits the activity of sialyltransferases. (A) mRNA levels of sialyltransferases and relevant enzymes in PANC-1 cells treated with or without Y-320, as detected by RT-qPCR. The result is the average of three independent experiments and mean ± SD are provided. (B) Transcriptional expression levels of sialyltransferases in PDAC patients from TCGA. (C–F) Kaplan–Meier survival analysis of PDAC patients stratified by sialyltransferases expression (ST3Gal1 (C), ST3Gal2 (D), ST6Gal2 (E), ST6GalNAc2 (F)) using data from The Cancer Genome Atlas (TCGA). (G) Schematic diagram of sialyltransferase member selection. (H) Schematic diagram of sialyltransferase activity detection at the cellular level using direct fluorescent sialic acid (SA) labeling. (I–K) Sialic acid transfer activity in PANC-1 cells transfected with flag-tagged ST3Gal1 (I), flag-tagged ST3Gal2 (J), or flag-tagged ST6GalNAc2 (K) overexpression plasmids, measured before and after treatment with 1 μM Y-320.

Next, we performed reverse molecular docking and molecular dynamics simulation to examine the potential binding targets of Y-320 among the aforementioned enzymes. Docking analysis revealed weak binding affinity between Y-320 and CMP-Neu5Ac synthase (docking GScore > −5 kcal/mol, ), suggesting a low likelihood of interaction. In contrast, Y-320 exhibited favorable binding modes with multiple sialyltransferases, as evidenced by significantly lower docking scores (). While these scores primarily assess binding mode plausibility rather than absolute affinity, the results suggest a higher probability of interaction with sialyltransferases. Furthermore, molecular dynamics simulations indicated that Y-320 maintained a stable conformation at the binding sites, exhibiting robust kinetic stability (). Consequently, we prioritized the verification of sialyltransferases as potential targets.

The addition of α-2,3SA and α-2,6SA could involve 14 sialyltransferases. To identify the most clinically relevant targets, we prioritized sialyltransferases based on two criteria: (1) high expression in PDAC tissues (Figure 2B) and (2) a strong association between their high expression and poor patient prognosis (Figure 2C–F, ). Applying these standards, we focused on ST3Gal1, ST3Gal2, and ST6GalNAc2 as primary candidates for further investigation (Figure 2G).

We evaluated the effect of Y-320 on the enzymatic activity of those sialyltransferases at the cellular level. In a cell-based experimental setup, Flag-ST3Gal1, Flag-ST3Gal2, Flag-ST6GalNAc2, and the Flag control were transiently overexpressed in PANC-1 cells, respectively. Cell lysates were then added to a reaction system containing a fluorophore-labeled donor (Cy5-CMP-SA) and the acceptor substrate (asialofetuin) (Figure 2H). We observed that overexpression of each sialyltransferase significantly increased Cy5-asialofetuin formation compared to the Flag control, confirming their sialylation activity. Treatment with 1 μM Y-320 markedly suppressed sialylation induced by all three sialyltransferases, suggesting its ability to suppress the enzymatic activities of these sialyltransferases (Figure 2I–K).

### Y-320 Directly Binds to Sialyltransferases *in Vitro* and in Cells

To characterize the molecular interactions between Y-320 and sialyltransferases, we first synthesized a biotin labeled Y-320 (denoted as Y-320-Biotin, Figure 3A). Enzyme activity assays confirmed that this modified compound maintained a potent sialylation inhibition capability (Figure 3B–C). Using streptavidin beads-based pull-down assays with Y-320-Biotin, we observed significant interaction between Y-320 and endogenous ST3Gal1, ST3Gal2, and ST6GalNAc2 in PANC-1 cells. Of note, Y-320 strongly bound to endogenous ST3Gal1, while its interactions with ST3Gal2 and ST6GalNAc2 were comparatively weaker (Figure 3D).

**Figure 3:**
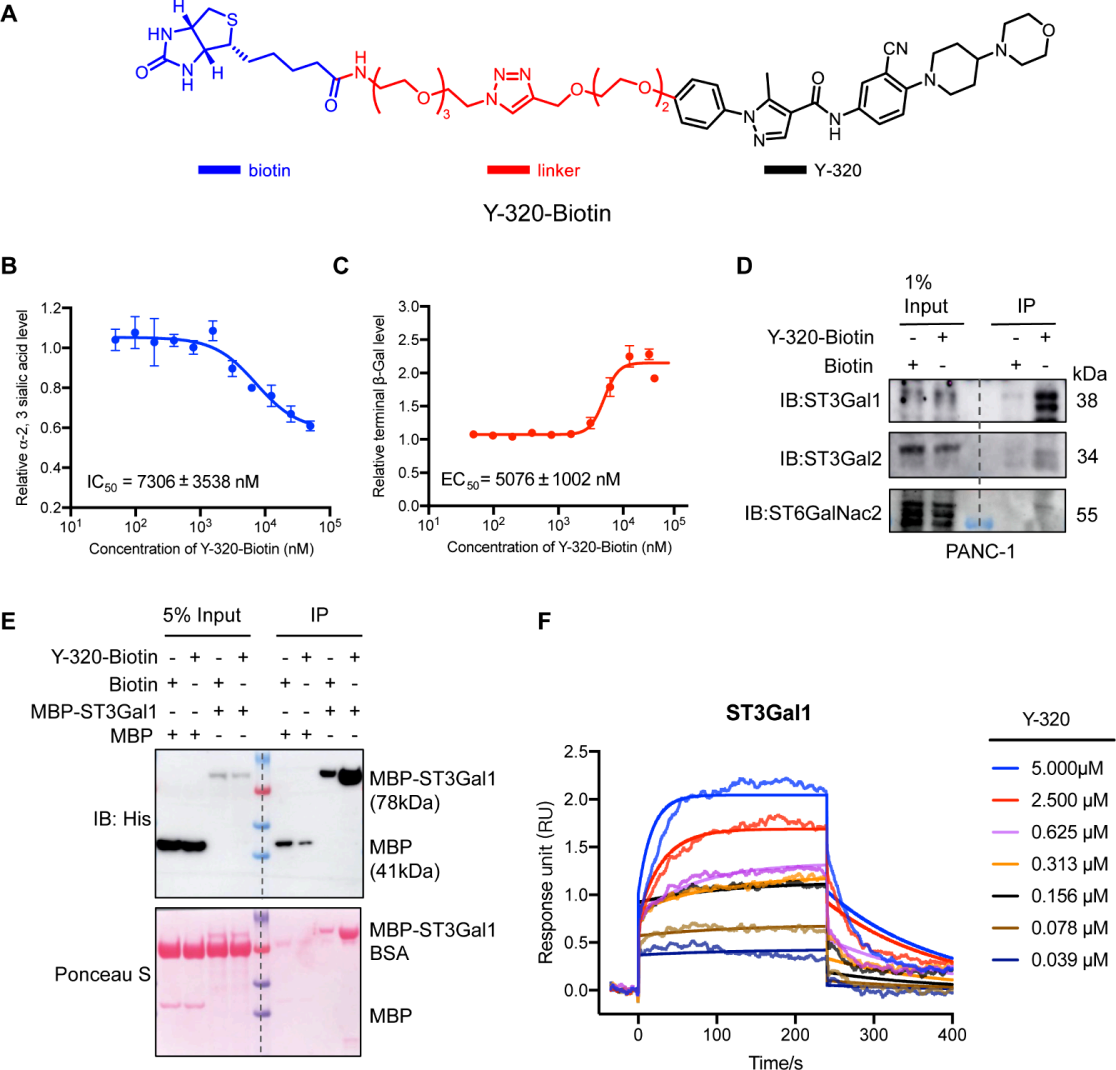
Binding affinity of Y-320 to ST3Gal1 quantified using biotinylated Y-320. (A) chemical structure of biotinylated Y-320 (Y-320-Biotin). (B) PANC-1 cells treated with serially diluted Y-320-Biotin, followed by measurement of α-2,3SA levels. Result is the average of three independent experiments and mean ± SD are provided. (C) PANC-1 cells treated with serially diluted Y-320-Biotin, followed by measurement of terminal β-gal levels. Result is the average of three independent experiments and mean ± SD are provided. (D) Coimmunoprecipitation (Co-IP) of endogenous ST3Gal1, ST3Gal2, and ST6GalNAc2 from PANC-1 cell lysates using Y-320-Biotin. IB, immunoblot; IP, immunoprecipitation. (E) Pull down analysis of the interactions between Y-320 and His-MBP tagged pig-ST3Gal1, using Y-320-Biotin immobilized on streptavidin beads. (F) Surface plasmon resonance (SPR) analysis of the interaction between Y-320 and pig ST3Gal1. Binding kinetics: Ka = 6.56 × 10^3^ (Ms)^−1^, *K*_d_ = 4.65 × 10^–3^ s^–1^, and KD = 709 nM.

To further investigate these interactions, we expressed and purified the recombinant pig ST3Gal1 protein (Δ45). *In vitro* pull-down assay using Y-320-Biotin demonstrated a significant interaction between Y-320 and the purified ST3Gal1 protein, consistent with the results obtained from the cell-based pull-down analysis (Figure 3E). Surface plasmon resonance (SPR) analysis with ST3Gal1 protein immobilized on CM5 chips further revealed that Y-320 binds to ST3Gal1 with Ka = 6.56 × 10^3^ (Ms)^−1^, *K*_d_ = 4.65 × 10^–3^ s^–1^, and equilibrium dissociation constant KD = 709 nM (Figure 3F).

Besides, we expressed and purified the recombinant human ST6Gal1 protein (Δ131) and further validated its interaction with Y-320. Unlike ST3Gal1, ST6Gal1 represents a distinct sialyltransferase subtype, and both are among the most well-characterized sialyltransferases to date. Pull-down and SPR analyses confirmed that Y-320 binds to ST6Gal1 with high affinity, Ka = 6.14 × 10^3^ (Ms)^−1^, *K*_d_ = 5.44 × 10^–3^ s^–1^, and KD = 885 nM (). These results again suggest that Y-320 acts as a pan-sialyltransferase inhibitor.

### Docking and Structure–Activity Relationship Analysis Reveal Mode of Action of Y-320 on ST3Gal1

To further characterize the interaction between Y-320 and ST3Gal1, a preliminary structure–activity relationship (SAR) analysis was conducted. Initially, two substructures bisected from Y-320 (Figure 4A, ) were proven to be inactive (Figure 4A, ), confirming that the intact molecular scaffold of Y-320 is indispensable for its biological function. Guided by the molecular docking predictions, analogues with systematic substitution of the key functional groups were synthesized, and their impact on the activity was evaluated. The terminal oxygen atom of the morpholine ring in Y-320 was found to form a critical hydrogen bond with G290 (Figure 4B–C). Structural modifications replacing the morpholine ring with a piperidine ring or removing either the morpholine or the adjacent piperidine ring abolished sialylation inhibition activity (Figure 4D, ). A derivative without the cyano group (**BY-007**) exhibited a marked decrease in activity (Figure 4E), highlighting its essential role in binding; this critical hydrogen bond interaction was also predicted by molecular docking (Figure 4B–C).

**Figure 4:**
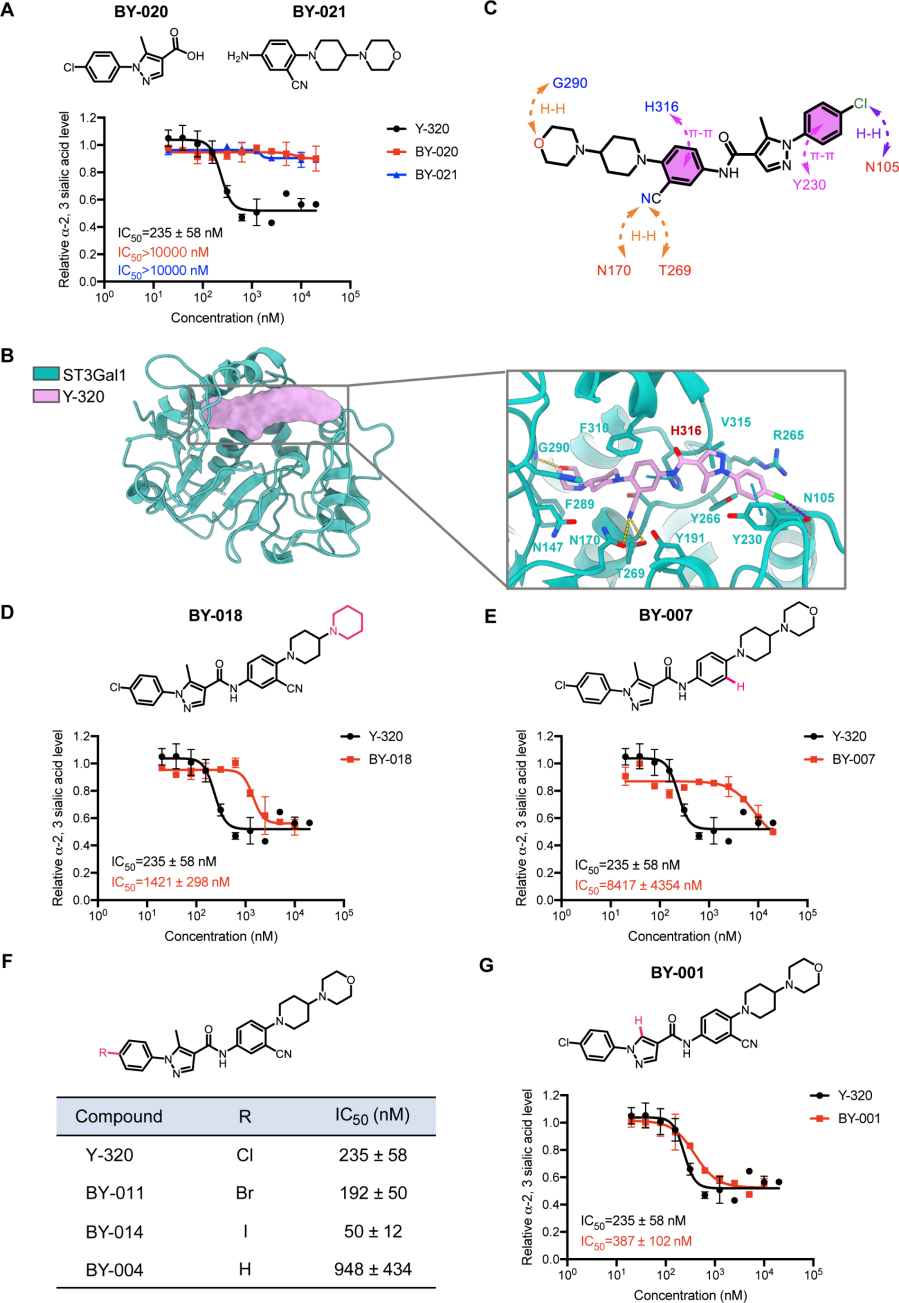
Mode of action (MOA) of Y-320 on ST3Gal1. (A) Chemical structures and inhibitory activities of compounds BY-020 and BY-021. Inhibition of α-2,3 sialylation was assessed using MALII lectin staining. (B–C) Molecular docking and molecular dynamics simulations reveal that Y-320 binds to the catalytic pocket of ST3Gal1. (D-G) Chemical structures and inhibitory activities of additional Y-320 analogues. Inhibition of α-2,3 sialylation was assessed using MALII lectin staining. Result is the average of three independent experiments and mean ± SD are provided in A, D, E, G.

Additionally, the 4-chlorophenyl moiety was proven to be pivotal (Figure 4B–C). On the one hand, the absence of the heteroatom at this site (**BY-004**) induced a 4-fold activity reduction (Figure 4F), suggesting a moderate but discernible contribution of this heteroatom to binding affinity by means of hydrogen bond interactions. On the other hand, a slight activity enhancement was observed after the more electron-rich 4-bromo/iodo-phenyl moiety (**BY-011** and **BY-014**) were adopted, validating the importance of the relevant π-π stacking interactions (Figure 4F). In contrast, the removal of the 5-methyl group from the pyrazole ring (**BY-001**) had a negligible impact on activity (Figure 4G), as predicted by the docking model (Figure 4B–C). Further, substitution of 5-methyl with bulkier ethyl or isopropyl groups (**BY-019** and **BY-024**) also did not affect the activity (), suggesting the tolerance of this position to modest steric modifications. Collectively, these SAR findings provided robust evidence supporting the molecular binding basis between ST3Gal1 and Y-320 and could offer insights for the rational design of more potent inhibitors.

### Y-320 Remodels the Tumor Immune Microenvironment for Antitumor Effect

We demonstrated that Y-320, as a pan-sialylation inhibitor, could effectively suppress sialylation on the surface of PDAC cells. To assess the potential *in vivo* antitumor activity of Y-320, we performed several subcutaneous tumor models in immune-competent mice. In the CT26 colon carcinoma model (Figure 5A), Y-320 treatment significantly deterred tumor progression with a tumor growth inhibition (TGI) of 51.0% (Figure 5B) and no obvious weight loss in mice (Figure 5C). Meanwhile, treatment with Y-320 markedly reduced the α-2,3SA level of tumor tissues by immunohistochemistry (Figure 5D).

**Figure 5:**
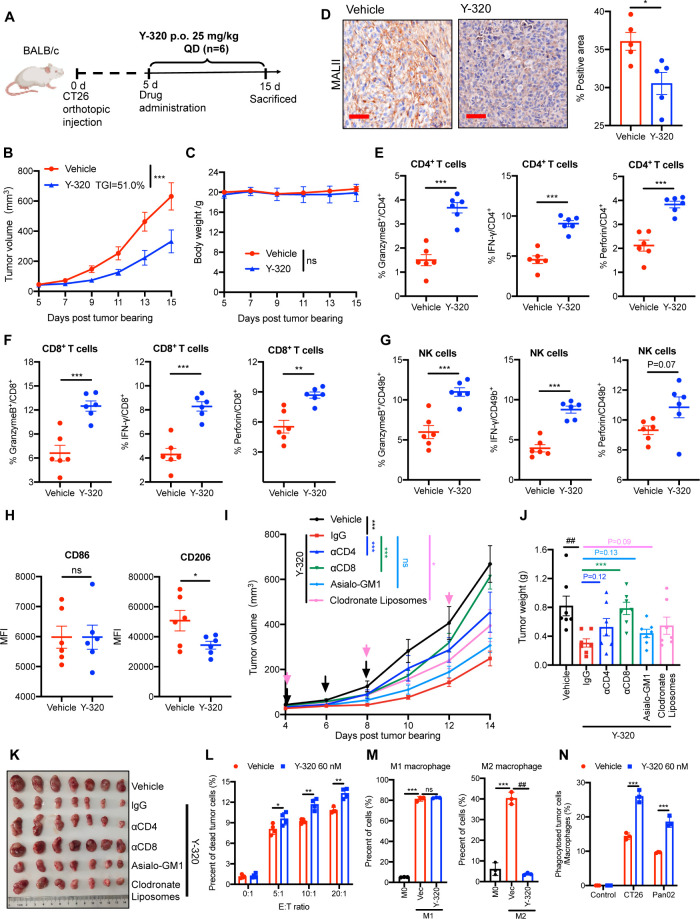
Y-320 induces antitumor effects in CD8^+^T cells and macrophages. (A) Experimental design for CT26 tumor treatment. Mice bearing CT26 tumors were orally administered Y-320 25 mg/kg daily (*n* = 6 per group). (B) Tumor volumes in mice treated with Y-320 or solvent control. (C) Body weight changes in mice during treatment. (D) Left, representative images of IHC staining of α-2,3SA treatment in CT26 tumor tissues; Scale bar, 50 μm. (E-G) Flow cytometry analysis of key cytokine secretion by CD4^+^T cells (e), CD8^+^T cells (f), and NK cells (g) in dLNs of CT26 tumor-bearing mice treated with Y-320. (H) Expression of CD86 and CD206 in tumor tissues by flow cytometry following treatment with 25 mg/kg Y-320 (*n* = 6). (I–K) CT26 tumor-bearing mice were pretreated with anti-CD4 and anti-CD8 antibodies, anti-Asialo-GM1 antibody, or clodronate liposomes, followed by oral administration of Y-320. (I) Tumor growth curves (*n* = 7). (J) Tumor weights at the experimental end point. (K) Representative images of dissected subcutaneous tumors at the end point. (L) Flow cytometry analysis of T cells cytotoxicity against Pan-02 cells. (M) Flow cytometry analysis of peritoneal macrophages differentiation *in vitro*, demonstrating the effects of 60 nM Y-320 treatment on macrophage polarization markers. (N) Flow cytometry analysis of macrophage phagocytosis against CT26 and Pan-02 cells. Mean ± SEM are provided in B-G. Mean ± SD are provided in L-N. Statistical analysis was performed using a two-way ANOVA for B, C, I; a Student’s unpaired two-tailed *t* test for D-H, L; a multiple *t* test for J and a one-way ANOVA for M, N. *, *P* < 0.05; **, *P* < 0.01; ***, *P* < 0.001 and ^##^, *P* < 0.01 as indicated.

Considering that sialoglycans on the surface of tumor cells could act as a glycoimmune checkpoint, we intended to further analyze the fluctuations of intratumoral comprehensive immunoprofiling. Through flow cytometry, we found that Y-320 increased the frequency of granzyme B^+^, IFN-γ and perforin^+^ CD4^+^ T cells and CD8^+^ T cells in draining lymph nodes (dLNs) (Figure 5E-F), indicating increased cytotoxic activity of T cells. Additionally, there was also an increase in the expression of effector molecules in natural killer cells (NK cells) after Y-320 administration (Figure 5G). Further analysis of macrophage polarization in tumor tissues demonstrated that Y-320 significantly reduced the proportion of M2 macrophages, while leaving M1 macrophages unaffected (Figure 5H). This aligns with reports that the Siglec-sialic acid axis drives macrophage polarization toward the M2 phenotype, which supports cancer progression.^10,33^

We also validated the efficacy of Y-320 in the MC38 colon carcinoma model (). Consistent with the CT26 model results, Y-320 demonstrated significant tumor suppression with a favorable safety profile (). Immunophenotyping also revealed increased frequencies of granzyme B^+^ and IFN-γ^+^ T cells and NK cells (), confirming Y-320 immune-activating properties. Collectively, these findings demonstrate that Y-320 can halt the progression of “hot” tumors and remodel the tumor’s immune microenvironment.

Further, to identify the specific immune cells contributing to Y-320-mediated tumor control, we selectively depleted key immune cell populations—CD4^+^ T cells, CD8^+^ T cells, NK cells, and macrophages—in CT26 tumor-bearing mice using anti-CD4 antibodies, anti-CD8 antibodies, anti-Asialo-GM1 antibodies, and clodronate liposomes, respectively (Figure 5I–K). The body weights of treated mice increased similarly over the course of the experiments among all treatment groups, suggesting no signs of acute toxicity (). The results showed that the depletion of CD8^+^ T cells nearly completely abrogated the antitumor efficacy of Y-320, underscoring the critical role of CD8^+^ T cells in Y-320-mediated tumor-targeted desialylation (Figure 5I–K), consistent with prior studies.^33^ Besides, macrophage depletion attenuated Y-320 therapeutic effect, indicating that macrophages could also be essential (Figure 5I–K).

To further validate these dependencies, we assessed the immune regulation effects of Y-320 on T cells and macrophages *in vitro*. When the mouse splenic T cells were cocultured with mouse pancreatic cancer cell line Pan-02, Y-320 promoted T cell killing against Pan-02 with a 22.8% increase (Figure 5L). As for macrophages, similar to the *in vivo* results, Y-320 significantly suppressed peritoneal macrophages (PM) and bone marrow-derived macrophages (BMDM) differentiation toward pro-tumorigenic M2 (Figure 5M, ). When mouse M2-type PM and Pan-02 cells were cocultured, Y-320 treatment increased the phagocytosis of Pan-02 cells by PM to 1.92-fold (Figure 5N, ). These results implied that Y-320 could directly regulate T cell-tumor cell interactions and macrophage-tumor cell interactions and boost their antitumor functions, which may contribute to the *in vivo* efficacy of Y-320.

### Combination of Y-320 and PD-1 Blockade Synergistically Enhances Antitumor Immunity in PDAC

Interestingly, Y-320 treatment unexpectedly increased the frequency of PD-1^+^ immune cells within tumors (), suggesting a potential feedback mechanism between the Siglec-SA and PD-1/PD-L1 signaling pathways. Bertozzi et al. reported that both genetic and therapeutic desialylation enhanced the efficacy of immune checkpoint blockade (ICB), supporting the rationale for combining Y-320 with ICB strategies.^18,33^ Given the aggressive and immunosuppressive microenvironment of PDAC and the limited effectiveness of anti-PD-1 immunotherapy in this malignancy, we tested whether Y-320 could enhance the efficacy of PD-1 blockade in the “cold” and αPD-1-resistant tumor model Pan-02 (Figure 6A). We found that αPD-1 alone had no antitumor effect in the Pan-02 model, while Y-320 monotherapy had limited efficacy with a TGI of 53.7%. Notably, the combination therapy demonstrated significantly stronger antitumor effects with an TGI of 85.4% (Figure 6B) and good tolerability in mice (). Moreover, the combination index (CI) could reach 1.53, suggesting that Y-320 and αPD-1 demonstrated a significant synergistic effect in the PDAC model.

**Figure 6:**
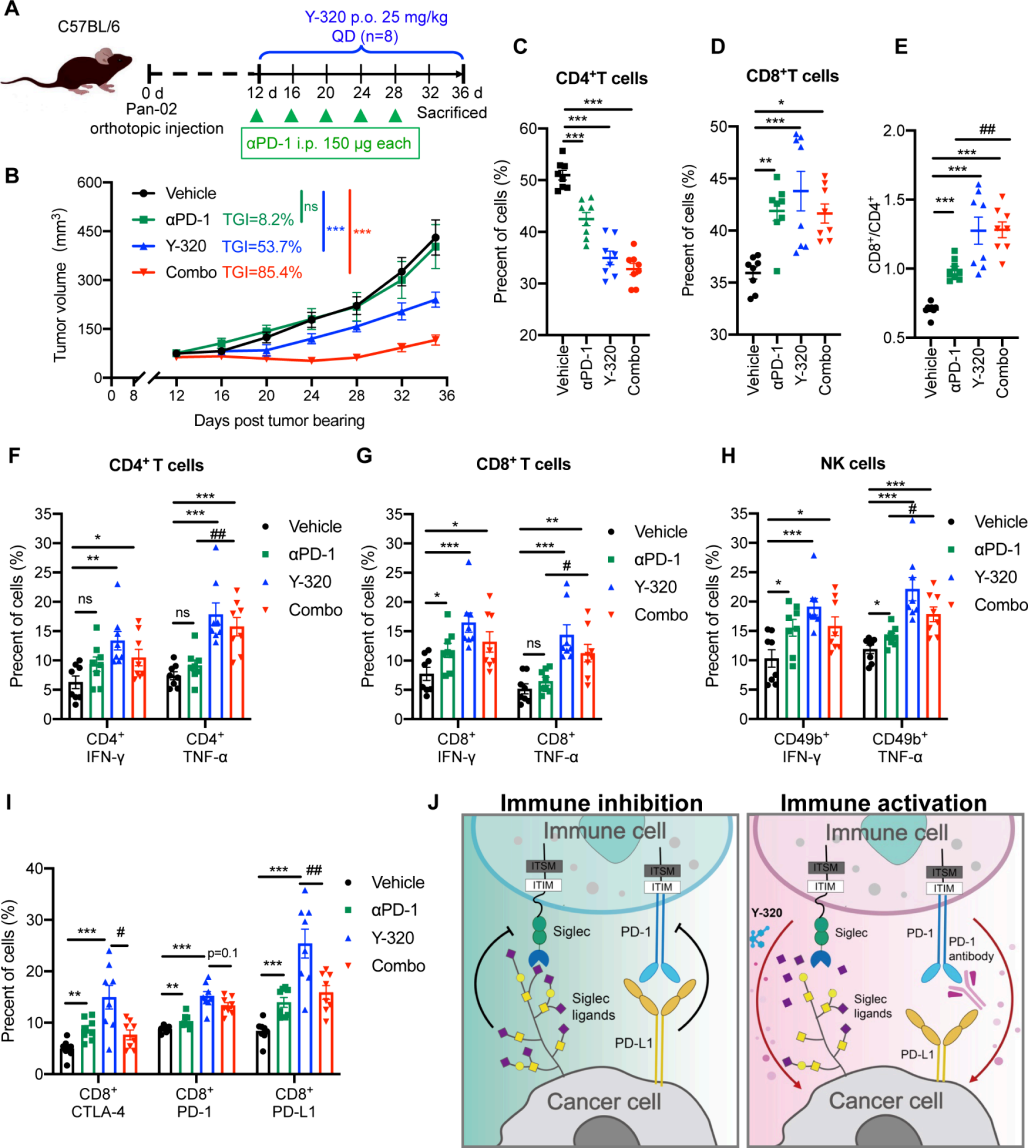
Y-320 enhances the antitumor effect of PD-1 antibody in Pan-02 model. (A) Experimental design for Pan-02 tumor treatment. Mice bearing Pan-02 tumors were treated with PD-1 antibody (150 μg, intravenously every 4 days) and/or 25 mg/kg Y-320 (orally daily) (*n* = 8 per group). (B) Tumor growth curves of Pan-02 tumors. (C–D) The frequency of CD4^+^ T cells and CD8^+^ T cells for CD3^+^ lymphocytes in dLN. (E) The ratio of CD8^+^T cells to CD4^+^T cells in mice was calculated. (F) Flow cytometry analysis of the frequency of INF-γ^+^ or TNF-α^+^ CD4^+^ T cells in dLN. (G) Flow cytometry analysis of the frequency of INF-γ^+^ or TNF-α^+^ CD8^+^ T cells in dLN. (H) Flow cytometry analysis of the frequency of INF-γ^+^ or TNF-α^+^ NK cells. (I) Flow cytometry analysis of the frequency of CTLA-4^+^, PD-1^+^, and PD-L1^+^ in CD8^+^T cells in dLN. (J) Schematic diagram of the combination of Y-320 and PD-1 antibody. Mean ± SEM are provided in B–I. Statistical analysis was performed using a two-way ANOVA for B and a multiple *t* test in C–I. *, *P* < 0.05; **, *P* < 0.01; ***, *P* < 0.001; ^#^, *P* < 0.05; ^##^, *P* < 0.01 as indicated.

Subsequently, we conducted an analysis of the immune microenvironment within dLNs by using flow cytometry. Y-320 treatment significantly reduced the frequency of CD4^+^ T cells (Figure 6C) while increasing the proportion of CD8^+^ T cells (Figure 6D), leading to a decreased CD4/CD8 ratio, indicative of more robust antitumor immune responses (Figure 6E). Furthermore, Y-320 monotherapy and combination therapy both enhanced the expression of cytotoxic markers in CD4^+^ T cells (Figure 6F, ), CD8^+^ T cells (Figure 6G, ), and NK cells (Figure 6H, and ). Notably, while Y-320 monotherapy increased the proportion of exhausted CD8^+^ T cells, combination therapy reduced this population, alleviating T cell exhaustion (Figure 6I, ). Collectively, this combination therapy could synergistically activate cytotoxic immune responses, overcoming the immunosuppressive tumor microenvironment and significantly improving therapeutic outcomes in PDAC (Figure 6J).

## Discussion

In this study, we identify small molecule Y-320 through cell-based high-throughput screening, which functions as a pan-sialylation inhibitor and potently suppresses α-2,3/2,6-sialylation in PDAC cells. In *in vivo* experiments, Y-320 significantly inhibits tumor growth by remodeling the tumor immune microenvironment. Importantly, Y-320 enhances the efficacy of PD-1 antibody, expected to be a clinical candidate to overcome ICB resistance in PDAC.

We found that Y-320 could promote cytotoxicity of T cells, inhibit M2 differentiation, promote phagocytosis of tumors by macrophages, and be mainly dependent on CD8^+^ T cells and macrophages for its efficacy. These results match the function of sialylation reported in previous studies,^10,33^ supporting that sialylation is an important player in the tumor immune microenvironment. Moreover, our findings reveal a negative feedback loop between sialic acid and the PD-1 mediated pathway, confirming that concurrent inhibition of both targets can overcome immune resistance.^18,33^ Y-320 ability to modulate the immune microenvironment suggests its potential application in the treatment of refractory tumors by ICB, offering opportunities for combination strategies to improve treatment outcomes across diverse cancer types.

The Y-320 molecule was initially discovered to inhibit IL-17 production by CD4^+^ T cells stimulated with IL-15 and used in autoimmune diseases such as rheumatoid arthritis.^34^ As an immune modulator, previous studies have demonstrated its ability to suppress immune responses in rheumatoid arthritis models. In contrast, the present research reveals a novel mechanism by which Y-320 activates antitumor immunity through the inhibition of multiple sialyltransferases and the reduction of sialylation on cancer cell surfaces. Moreover, Y-320 has also been reported to be able to enhance the sensitivity of multidrug-resistant tumor cells to lower doses of cytotoxic agents, including paclitaxel, vinorelbine, and doxorubicin.^35^ This chemosensitizing effect may also be attributed to its regulation of sialylation, as studies have shown that knockdown of ST6Gal1, a key sialyltransferase, could enhance sensitivity to gemcitabine, a frontline treatment for pancreatic cancer.^36^ Besides, previous study has identified Y-320 as a potent enhancer of premature termination codons (PTC) readthrough.^37^

Our molecular docking analysis revealed that Y-320 bound to the substrate pocket of several ST3- and ST6-sialyltransferases, specifically interacting with the highly conserved histidine residue within the VS motif (). This histidine residue, conserved across all 20 sialyltransferase members,^38^ actively participates in binding interactions with Y-320. These results support the observed phenotype that Y-320 simultaneously inhibits the formation of both α-2,3SA and α-2,6SA, even in the presence of compensatory mechanisms mediated by multiple sialyltransferase members.

Our observation that Y-320 suppresses M2 macrophage polarization (Figure 5M, ) further supports the established role of tumor-derived sialic acids in driving tumor-associated macrophage differentiation.^10^ Current evidence indicates that immune cells display abundant surface sialylation which functionally regulate their behavior, including macrophage differentiation.^3^ We therefore propose that Y-320 may target sialic acid not only on tumor cells but also on immune cells, offering a promising strategy to disrupt the immunosuppressive tumor microenvironment, pending further experimental confirmation. Recent advances in research on the regulatory role of Siglec signaling in immune cells, such as Tregs and B cells,^39,40^ within the tumor microenvironment suggest that we need to conduct more in-depth studies in the future to evaluate the effects of Y-320 on various players in the tumor immune microenvironment, as well as on specific innate and adaptive immune responses.

The cell-based high-throughput screening method for sialylation developed in this study can be extended to investigate other glycosylation modifications. This is particularly important given the growing recognition of the role of glycans in various diseases, including cancer, infectious diseases, and autoimmune disorders.^41^ The ability to screen for compounds that modulate specific glycan structures could lead to the development of novel treatments for a wide range of conditions.

The present study has identified a highly potent small-molecule sialylation inhibitor that effectively overcomes the low cellular activity limitation of existing inhibitors. Furthermore, Y-320 stands out among other desialylation therapies due to its oral bioavailability, ease of preparation,^42^ and favorable patient compliance. Additionally, Y-320 is simpler and more cost-effective to produce compared with antibody-based drugs, while its oral administration enhances patient compliance and clinical applicability. Elevated levels of sialic acid have been associated with poor prognosis in various cancers, including breast, lung, and colorectal cancers.^43^ Since hypersialylation is not exclusive to PDAC but is a common feature across numerous malignancies,^20^ Y-320 holds promise as a therapeutic agent for a broad spectrum of hypersialylation-driven cancers.

Notwithstanding, this study has several limitations that warrant further investigation. First, the preference of Y-320 for inhibiting specific sialylated glycoforms and its selectivity among members of the sialyltransferase family remain unclear. This gap in understanding highlights the need for more detailed biochemical and structural analyses to elucidate the molecular interactions underlying Y-320 activity. Second, the mechanism by which Y-320 relies on CD8^+^ T cells and macrophages to regulate tumor immunity needs to be thoroughly investigated. Furthermore, the *in vivo* safety and pharmacokinetics of Y-320 and its active analogues need to be systematically evaluated to assess their potential for clinical translation. Although at the administered dose, Y-320 did not exhibit risks such as renal toxicity similar to that caused by P-3F_ax_-Neu5Ac (), dose–response analysis and major organ pathology analysis still need to be conducted.^44^ And targeting delivery to tumors via antibody- or liposome-dependent strategies should be considered to enhance the safety and efficacy of Y-320.

In summary, the present disclosure of Y-320 represents a major step forward in the development of sialylation inhibitors, offering a potent, orally available, and easily manufacturable solution for targeting hypersialylation. Its ability to synergize with immune checkpoint blockade and modulate the tumor immune microenvironment should position it as a promising candidate for clinical development. In the future, further structural modifications of Y-320 will be pursued to achieve enhanced therapeutic potency, optimized druglike properties, and reduced adverse effects.

## Methods and Materials

### Cell Lines and Cell Culture

The PANC-1 and MIA PaCa-2 cells were obtained from the ATCC, and were incubated at 37 °C with 5% CO_2_ and grown in Dulbecco’s Modified Eagle Medium (DMEM; SH30022.01, Hyclone) supplemented with 10% heat-inactivated fetal bovine serum (FBS; 10100147, Life Technologies). CT26 cell line was purchased from Procell. MC38 cell line was purchased from BNCC. The Pan-02 cell line was purchased from BeNa. Pan-02 cells were cultured in DMEM (MA0212, Meilunbio) supplemented with 1% penicillin/streptomycin (P/S; 15140122, Invitrogen) and 10% FBS (10100147, Life Technologies). CT26 and MC38 were cultured in RPMI1640 medium (MA0215, Meilunbio) containing 1% P/S and 10% FBS. All cells were cultured at 37 °C in a 5% CO_2_ humidified atmosphere. All cell lines were authenticated by STR and tested for Mycoplasma contamination.

### Lectin Stain

Cells treated with drugs for 48 h were fixed in 4% paraformaldehyde for 5 min and then fixed in methanol stored at −20 °C for 10 min, and then blocked with 1% goat serum (C510001, Sangon Biotech) in PBS for 1 h. For MALII staining, the cells were then incubated with MALII-biotin (B-1265–1, Vectorlabs), overnight at 4 °C, and then stained with streptavidin conjugated with Fluorescein (SA-5001–1, Vectorlabs) for 4 h at room temperature. For SNA and PNA staining, the cells were incubated with SNA-Fluorescein (FL-1301–2, Vectorlabs) or PNA-Rhodamine (RL-1072–5, Vectorlabs) for 4 h at room temperature. Nuclei were stained with DAPI (D9542, Sigma). A high-content screening instrument (Operetta) was used for image capture and quantification.

### RT-qPCR

Total RNA was extracted by TRizol reagent (Invitrogen) from cells and reverse-transcribed into cDNA using HiScript III All-in-one RT SuperMix (R333–01, Vazyme). QPCR was performed to detect mRNA expression of genes by the ChamQ STBR qPCR Master Mix (Q331–02, Vazyme) and primers are shown in .

### SA-Label Assay

The CDS (coding sequence) regions of ST3Gal1, ST3Gal2 and ST6GalNAc2 were cloned into pcDNA3.1 vectors. PANC-1 cells were transfected with the plasmids in 6 cm dishes for 24 h, and then 1 μM Y-320 was added into cells treated for 48 h. Next, cells were resuspended with 200 μL of 25 mM Tris buffer pH 7.5 added with 2% glycerol and protease inhibitors and phosphatase inhibitors. Then, 30% power ultrasound at 20 J for each sample and the cell lysate were used after centrifugation.

The reaction was carried out in a 20 μL buffer containing 25 mM Tris (pH 7.5), the aforementioned cell lysate, 10 μg of asialofetuin (AF, A1908, Sigma), 10 mM MgCl_2_, and 0.2 nmol of Cy5-conjugated CMP-SA (ES302–050, R&D) at 37 °C for 1–2 h. The reaction products were then analyzed by SDS-PAGE. Ponceau S was used to stain total protein for control.

### Protein Preparation

For MBP fused pig ST3Gal1, the gene encoding ST3Gal1 (UniProt: Q02745, Δ45) was codon-optimized for *E. coli* and cloned into pET28a with an N-terminal 6xHis-MBP tag. The plasmid was transformed to *E. coli* Origami2 (DE3) cells. Protein expression was induced with 0.5 mM IPTG at OD600 = 0.8, followed by 18 h incubation at 16 °C. Cells were harvested, resuspended in lysis buffer (25 mM Tris, 150 mM NaCl, 20 mM imidazole, 5 mM β-mercaptoethanol, 5% glycerol, pH 8.2), and lysed. The supernatant was purified using a HisTrap FF column (17525501, Cytiva) with an imidazole gradient (20–500 mM). Target fractions were pooled, concentrated using 10 kDa ultrafiltration tubes (UFC901096, Millipore), and buffer-exchanged to 25 mM Tris, 150 mM NaCl, pH 8.2. Final purification was performed using a Superdex 200 10/300 GL column (28990944, Cytiva) to obtain the high-purity protein.

For human ST6Gal1, the DNA encoding ST6GAL1 (UniProt: P15907, Δ131) was cloned into the psectag-2A-3 × FLAG vector and transfected into HEK 293F cells using PEI 40000 (40816ES03, Yeasen). After 4.5 days of culture, the supernatant was collected for purification. Flag affinity chromatography was performed using an Anti-DYKDDDDK G1 Affinity Resin gravity column (L00432, GenScript) followed by competitive elution with 200 μg/mL FLAG peptide in TBS. After concentration, final purification was performed using a Superdex 200 10/300 GL column (28990944, Cytiva) to obtain the high-purity protein.

### Coimmunoprecipitation and Pull-Down Assay

Preparation of the Streptavidin Beads: Streptavidin beads (Thermo Scientific, 88817) were incubated with 50 μM Y-320-Biotin or D-biotin (negative control) in phosphate-buffered saline (PBS) for 1 h at 4 °C. The beads were washed three times with 0.1% PBST (PBS containing 0.1% Tween-20) to remove unbound compounds.

For the pull-down assay, 100 μg of purified His-MBP-pST3Gal1, His-MBP (negative control for ST3Gal1), or Flag-ST6Gal1 was incubated with the prepared streptavidin beads at 4 °C for 4 h with gentle rotation. After incubation, the beads were washed five times with ice-cold wash buffer (20 mM Tris-HCl, pH 7.5, 250 mM NaCl, 0.5% NP-40, and 0.1% BSA) to remove nonspecifically bound proteins.

For co-IP experiments, clarified cell lysates were incubated with streptavidin beads preloaded with Y-320-Biotin or D-biotin (negative control) at 4 °C overnight. The beads were then washed five times with ice-cold 0.1% PBST to eliminate nonspecific interactions.

Elution and Analysis: Bound proteins were eluted by boiling the beads in 2x Laemmli sample buffer at 95 °C for 10 min. The eluted samples were resolved by SDS-PAGE and analyzed by Western blotting with the corresponding antibodies listed in .

### Surface Plasmon Resonance (SPR) Assay

The experiment was conducted with a Biacore T800 instrument. Recombinant wild type ST3Gal1 or ST6Gal1 protein was coupled on a CM5 chip (Series S, Cytiva). Y-320 was first dissolved in DMSO as 20 mM stock and then diluted in PBS buffer as mobile phase flowing through the ST3Gal1 or ST6Gal1 coupled chip. Dissociation constant was calculated in kinetic analysis mode by using Biacore evaluation software. The final figure was displayed by using GraphPad (version 8.0).

### Reverse Molecular Docking

Reverse molecular docking was performed using several modules in Maestro 2023–4 (Schrödinger Inc.). The molecular structure of Y-320 was processed with the LigPrep module, and Epik^45^ generated various protonation states under pH 7.0 ± 2.0 conditions. Structures of ST3 and ST6-sialyltransferases, donor synthases, and transporters were retrieved from the AlphaFold Protein Structure Database^46^ and optimized with the Protein Preparation Workflow. Cross docking was then conducted using Xglide,^47^ using these protein structures as the receptor library and Y-320 as the query ligand. Active sites for docking were identified using the Sitemap module with the docking precision set to SP and only the top-ranked complex retained.

### Molecular Dynamics (MD) Simulation

MD simulations were performed using Desmond^48^ package of Schrödinger 2023–4 with the OPLS4 ^49^ force field. The complexes from reverse molecular docking were further optimized by the Minimization module as the initial states for MD simulations. The optimized complex was explicitly solvated with TIP3P water molecules under cubic periodic boundary conditions for a 15 Å buffer region. The overlapping water molecules were deleted, and 0.15 M KCl was added. The systems were neutralized by adding K^+^ as counterions. Brownian motion simulation was used to relax these systems into local energy minimum states separately. An ensemble (NPT) was then applied to maintain the constant temperature (310 K) and pressure (1.01325 bar) of the systems, and the simulations were started with three different random initial velocities in triplicate at the temperature of 310 K. The analysis of the trajectories was using AutoTRJ,^50^ which integrated simulation analysis scripts from Schrödinger, Inc. Additionally, the three-dimensional structures of proteins were visualized using ChimeraX.^51^

### Animals

Female BALB/c and C57BL/6 mice of 6–8 weeks, purchased from Vital River Laboratory, were raised in the SPF environment of the Shanghai Institute of Materia Medica, Chinese Academy of Sciences (SIMM). Animal care and experiments were performed according to protocols approved by the Institutional Animal Care and Use Committee (IACUC).

### *In Vivo* Antitumor Efficacy Study of Y-320

The CT26, MC38, and Pan-02 tumor models were established by subcutaneously injecting 1 × 10^6^ CT26 cells into female BALB/c mice, 1 × 10^6^ MC38 cells into C57BL/6 mice, and 2 × 10^6^ Pan-02 cells into C57BL/6 mice, respectively, on the dorsal side of the right forelimb. Tumor-bearing mice were randomly assigned to receive intragastric administration of Y-320 (25 mg/kg) daily and weighed. For the Pan-02 model, the αPD-1 antibody (BE0273, InvivoMab) was administered intraperitoneally every 4 days (150 μg/each) until the animal was dissected. Tumor volume was calculated as tumor volume (mm^3^) = tumor length × width × width/2. The formula for calculating the combination index is CI = E­(A+B)/(EA+EB-EA × EB), where EA is the tumor inhibition rate of drug A, EB is the tumor inhibition rate of drug B, and E­(A+B) is the tumor inhibition rate of the combination of the two drugs. When the CI value is >1.15, it indicates synergy. During the dissection, tumor tissues were collected for photography, weighing, and flow cytometry. The draining lymph nodes were also collected for flow cytometry analysis.

### Immune Cell Depletion Study

The CT26 tumor model was constructed as described above. On the fourth, sixth, and eighth days after tumor implantation, the mice were intraperitoneally injected with anti-CD4 antibodies (150 μL/each; BE0119, Bio X Cell) to deplete CD4^+^ T cells. On the fourth, sixth, and eighth days, the mice were intraperitoneally injected with anti-CD8β antibodies (150 μL/each; BE0223, Bio X Cell) to deplete CD8^+^ T cells. On the fourth, sixth, eighth, and 10th days, the mice were intraperitoneally injected with anti-Asialo-GM1 antibodies (35 μL/each; 146002, BioLegend) to deplete NK cells. On the fourth day (200 μL/each), eighth day (100 μL/each), and 12th day (100 μL/each), the mice were intraperitoneally injected with Clodronate Liposomes (40337ES08, Yeasen) to deplete macrophages. After 5 days of tumor bearing, mice were subjected to intragastric administration of Y-320 (25 mg/kg) daily and weighing. Tumor volume was measured every 2 days. During the dissection, the tumor tissues were retrieved for photography and weight measurement and the draining lymph nodes were extracted for flow cytometry analysis.

### Immunophenotype Analysis

Tumor tissues were digested with hyaluronidase (20426ES60, Yeasen) and collagenase IV (40510ES60, Yeasen) and then passed through a 75 μm nylon mesh (7061011, Dakewe) to prepare single-cell suspensions. The suspensions were treated with red blood cell lysis buffer (40401ES60, Yeasen) to remove the red blood cells. The cells were blocked by 4% FPBS containing antimouse CD16/CD32 (101320, BioLegend) at 4 °C for 30 min and then incubated with surface marker antibody solution at 4 °C for 20 min for surface fluorescence labeling. Then, after permeabilizing the cells with BD cytofix/cytoperm buffer (554714, BD Biosciences), the cells were labeled by the intracellular marker antibody solution at 4 °C for 30 min for intracellular labeling. Flow cytometry analysis was performed using ACEA NovoCyte (Agilent) and data processing was done through NovoExpress software (version 1.6.0).

Draining lymph nodes were ground using a syringe and passed through a 75 μm nylon mesh to make single-cell suspensions. The cells were cultured with RPMI1640 complete medium containing a leukocyte activation cocktail with BD GolgiPlug (550538, BD Pharmingen) and incubated in a cell culture incubator for 6 h. Subsequently, cells were collected and stained with the corresponding antibodies listed in .

### *In Vitro* Macrophage Differentiation Assay

Peritoneal macrophages (PM) were obtained by injecting 1 mL of 3% Brewer’s Thioglycolate medium (LA4590, Solarbio) into the peritoneal cavity of mice, stimulating mice for 72 h, and then collecting the peritoneal lavage fluid from the mice. PM (2–4 × 10^6^ cells/mL) were allowed to stand in complete RPMI1640 medium for 12 h, and then, the supernatant was removed. Subsequently, PM were incubated with M1 inducer containing IFN-γ (20 ng/mL, Z02916–100, GenScript) and LPS (100 ng/mL, L2630–100MG, SIGMA) or M2 inducer containing IL-4 (40 ng/mL, Z02996–50, GenScript), respectively, along with Y-320 (60 nM) for 48 h. Finally, the macrophages were collected for flow cytometry analysis.

Bone marrow macrophages (BMDM) were obtained by collecting the humeral wash from mice and culturing it in complete DMEM medium containing M-CSF (20 ng/mL, Z03275–50, GenScript) for 96 h. After the nonadherent cells were removed from the supernatant, BMDM were treated with M1 inducer with LPS (100 ng/mL), IFN-γ (20 ng/mL), and M-CSF (20 ng/mL), or M2 inducer with IL-4 (40 ng/mL) and M-CSF (20 ng/mL), respectively, and incubated for 48 h with Y-320 (60 nM). Finally, the macrophages were collected for flow cytometry analysis.

### Macrophage Phagocytosis Assay

PM were induced to become M2 cells, and the M2 cells were simultaneously treated with DMSO (PWL064, Meilunbio) or Y-320 (60 nM). The density of the M2 cells was adjusted to 2 × 10^5^ cells/mL. After labeling the CT26 cells with the CFSE reagent (MB2308, Meilunbio), the cell density was adjusted to 1 × 10^5^ cells/mL. The labeled CT26 cells were added to M2 cells and cocultured together for 24 h. Finally, the cocultured cells were collected for flow cytometry analysis.

### T-Cell Killing Assay

T cells were obtained by sorting splenocytes from C57BL/6 mice using an EasySep Mouse T Cell Isolation Kit (19851, STEMCELL). The sorted T cells were transferred to culture plates coated with antimouse CD3ε (1 μg/mL, 100340, BioLegend)/antimouse CD28 (1 μg/mL, 102116, BioLegend) antibodies, and the cells were cultured in complete RPMI1640 medium containing IL-2 (10 ng/mL, Z02764, GenScript) for 24 h. T cells were added in proportion to the Pan-02 tumor cell culture plates that had been prepared 12 h in advance, and cocultured with Y-320 (60 nM) for 24 h. Finally, the cells were collected for apoptosis analysis by flow cytometry.

### Statistics

All *in vitro* experiments were performed independently three times independently. Quantitative data from *in vitro* experiments were shown as mean ± SD; while the data from *in vivo* experiments were shown as mean ± SEM. One-way ANOVA test and Student’s two-tailed *t* test was used for statistical analysis with multigroup and two-group experiments, respectively. Two-way ANOVA test was used for analyzing the statistical change of tumor volume in tumor xenograft experiments. Kaplan–Meier (KM) survival analysis was performed using survival package in R and the P value was assessed by log-rank test. *P* < 0.05 was considered significant statistically.

## Supplementary Material





## Notes

Additional details including the supplementary figures, supplementary tables, synthesis and characterization of compounds, as well as copies of ^1^H NMR, ^13^C NMR, HRMS spectra (PDF)
Transparent Peer Review report available (PDF)

## Notes

All animal experiments were carried out according to the Institutional Ethical Guidelines on animal care and were approved by the Institutional Animal Care and Use Committee, Shanghai Institute of Materia Medica, Chinese Academy of Sciences (2024–03-GLK-55).
