# Supplementary material for: A Potent Oral Sialylation Inhibitor Augments the Immunotherapy in Pancreatic Ductal Adenocarcinoma
Source: ACS Cent Sci. 2025 Sep 3;11(10):1969–83. doi: 10.1021/acscentsci.5c00939 (PMC12550625; doi:10.1021/acscentsci.5c00939)
Supplement: Supplementary file 1 [file oc5c00939_si_001.pdf]

Supporting information

A potent oral sialylation inhibitor augments the immunotherapy in pancreatic ductal adenocarcinoma

Jiahui Mou[1, #], Runqiu Chen[2, 3, #], Zihao Dai[1, #], Hao Yang[5], Feiyashan Suo[4], Yifan Li[4], Yangxu Ye[1], Pengfei Fang[1, 4], Fang Bai[5], Yachen Zhao[4], Rong Zhang[6], Yiru Long[3, *], Likun Gong[3, *], Jing Wang[1, 4, *], Biao Yu[1, 4, *]

Affiliations:

[1] State Key Laboratory of Chemical Biology, Shanghai Institute of Organic Chemistry, Chinese Academy of Sciences, Shanghai 200032, China

[2] Shanghai Institute of Infectious Diseases and Biosecurity, Fudan University, Shanghai 200032, China

[3] State Key Laboratory of Drug Research, Shanghai Institute of Materia Medica, Chinese Academy of Sciences, Shanghai 201203, China

[4] School of Chemistry and Materials Science, Hangzhou Institute for Advanced Study, University of Chinese Academy of Sciences, Hangzhou 310024, China

[5] School of Life Science and Technology, and Shanghai Institute for Advanced Immunochemical Studies, ShanghaiTech University, Shanghai 201210, China

[6] School of Public Health, China Medical University, Shenyang 110122, China

[*] Corresponding author.

Emails: s18-longyiru@simm.ac.cn; lkgong@simm.ac.cn; Jwang@sioc.ac.cn; byu@sioc.ac.cn

[#] These authors contributed equally to this work.

Content

Supporting figures **3**

Supporting tables **16**

Synthesis and characterization of compounds **19**

Supporting figures

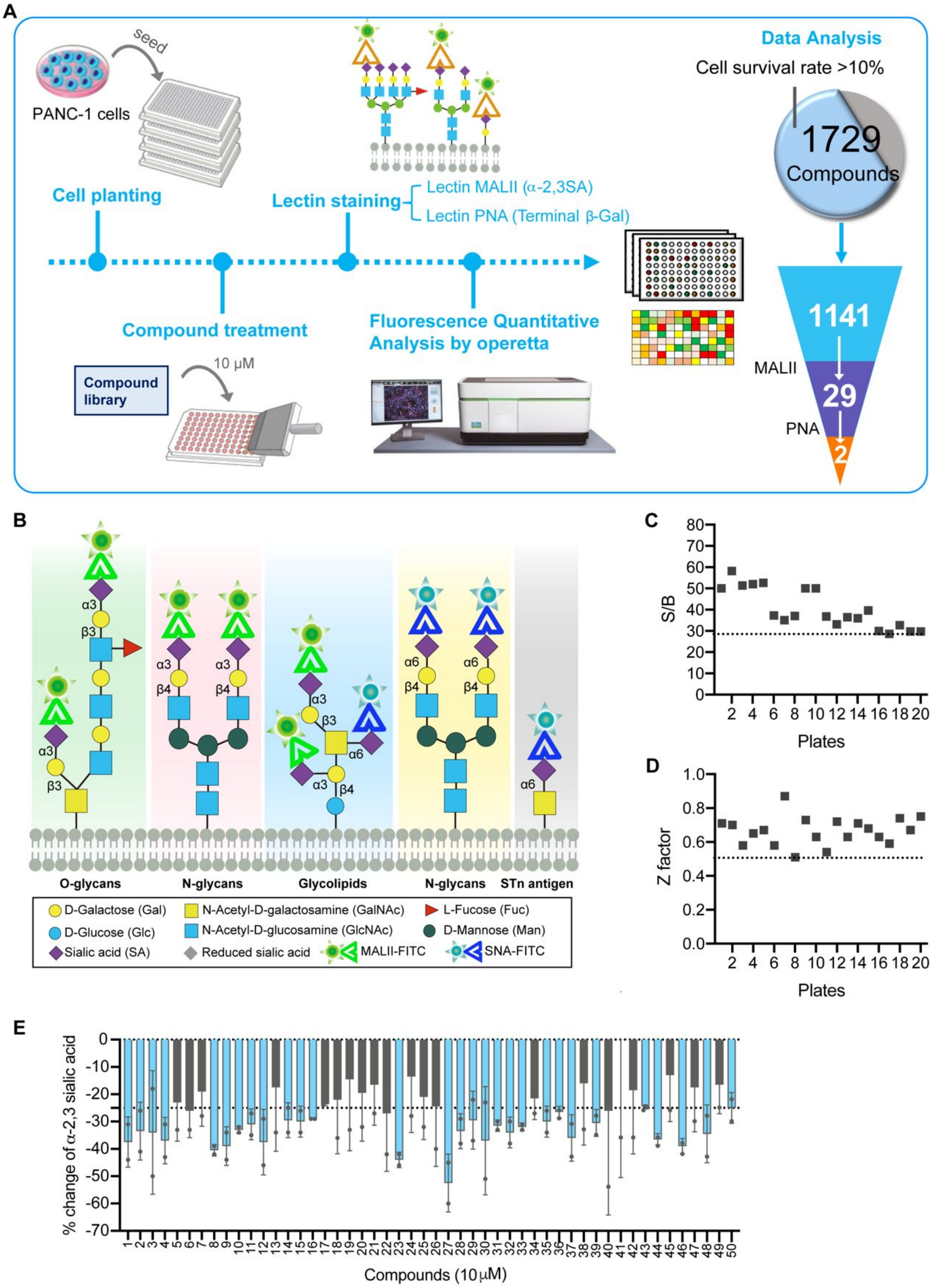

Figure S1. Primary screening for sialylation inhibitors. (A) Screening workflow to identify sialylation inhibitors. **(B)** Glycan structures recognized by lectin MALII and SNA. **(C)** The signal-to-background (S/B) ratio in the screening experiment. **(D)** The

Z factors in the screening experiment. **(E)** Relative α-2,3 sialic acid level in PANC-1 cells measured 72 h after treatment with hits from high-throughput screening at a final drug concentration of 10 μM. Result is the average of three independent experiments and mean ± SD are provided.

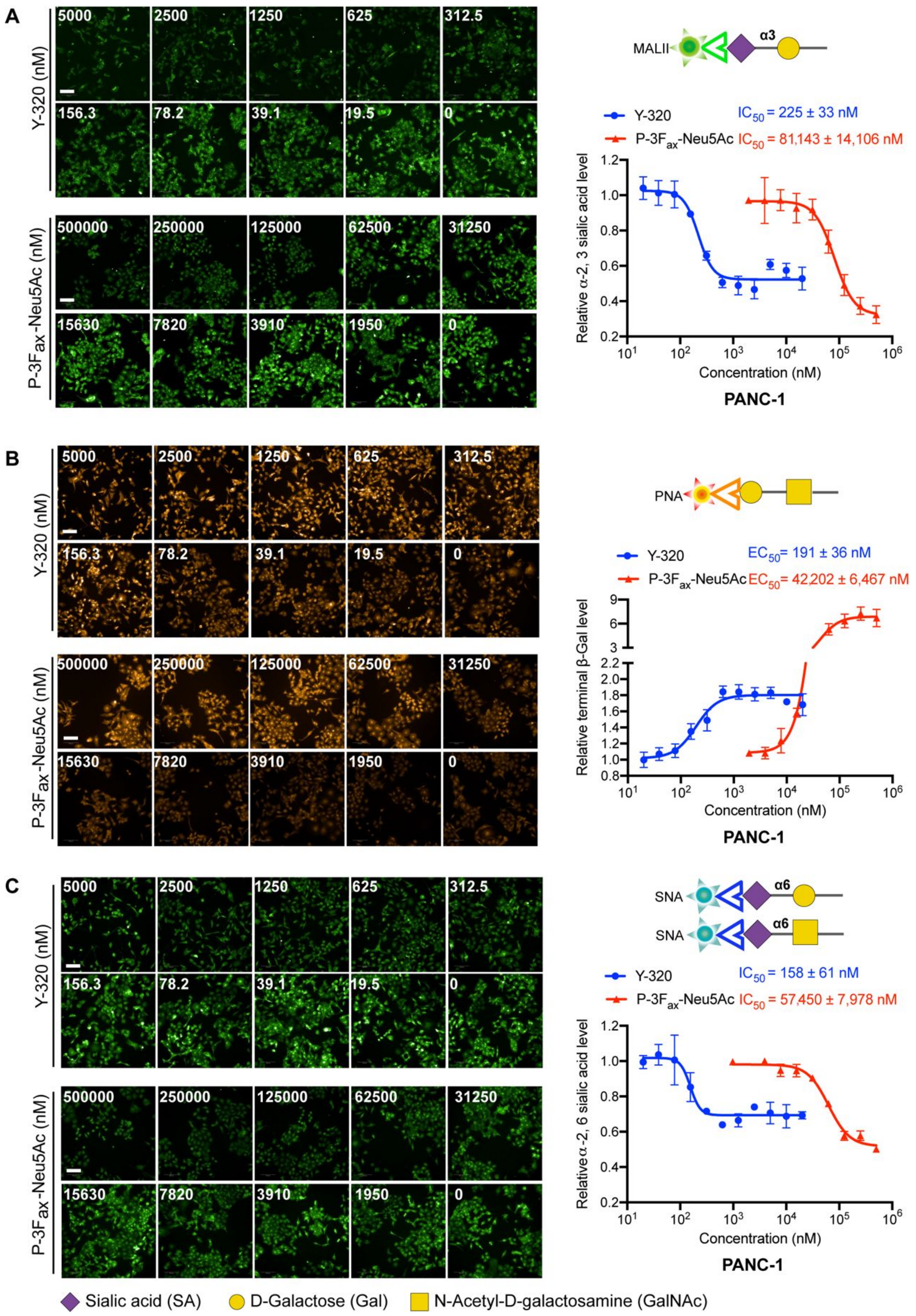

Figure S2. Y-320 and P-3F$_{ax}$-Neu5Ac inhibit the sialylation of PANC-1 cells. (A-C) PANC-1 cells were treated with increasing concentrations of Y-320 (top) or P-3F$_{ax}$-Neu5Ac (bottom), and α-2,3SA (**A**), terminal β-gal levels (**B**) and α-2,6SA (**C**) were assessed using indicated lectin staining. Representative images of membrane-

bound lectins acquired using the Operetta system; scale bar, 100 μm. Result is the average of three independent experiments and mean ± SD are provided.

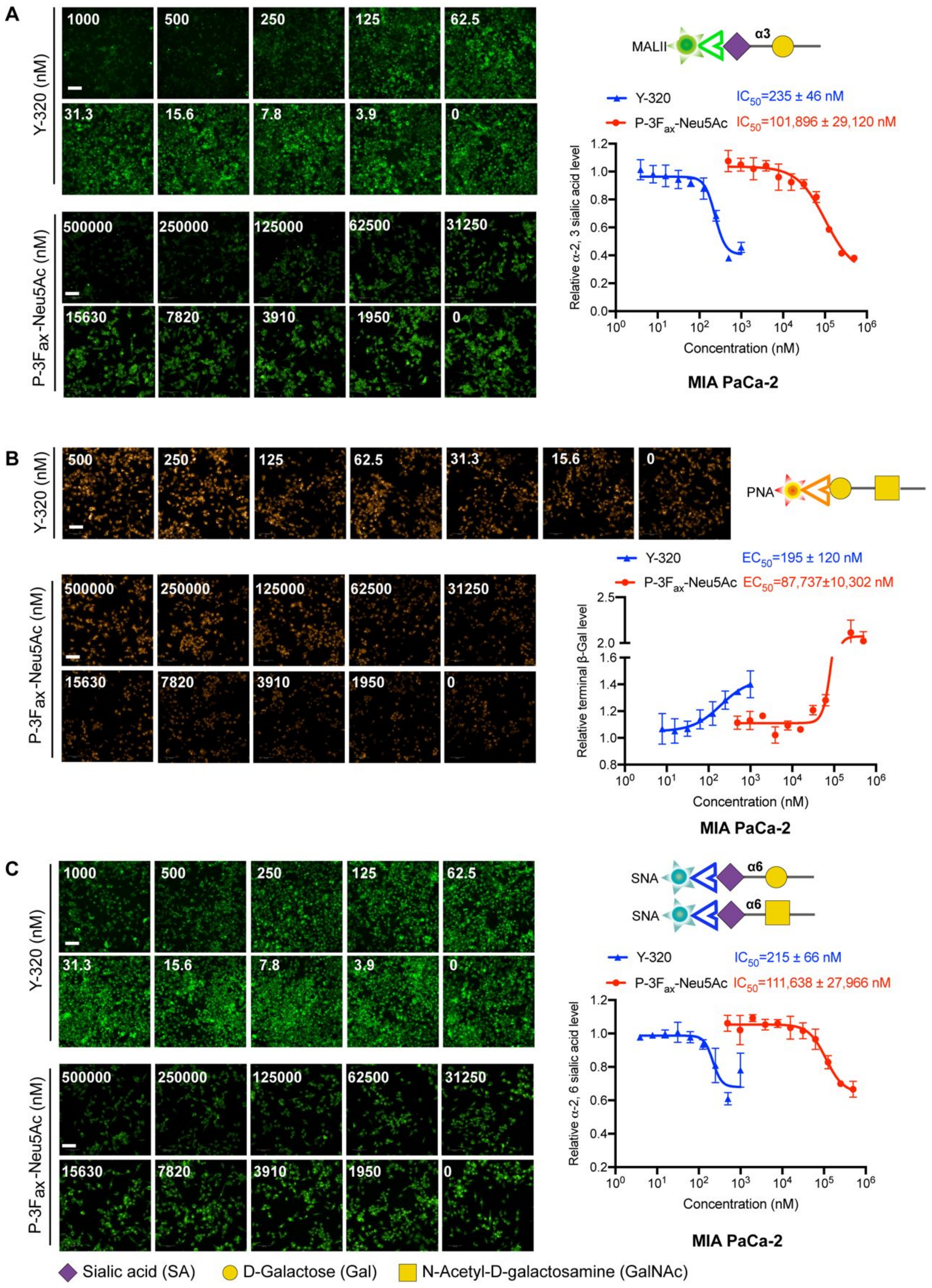

Figure S3. Y-320 and P-3F$_{ax}$-Neu5Ac inhibit the sialylation of MIA PaCa-2 cells. (A-C) MIA PaCa-2 cells were treated with increasing concentrations of Y-320 (top)

or P-3F$_{ax}$-Neu5Ac (bottom), and α-2,3SA (**A**), terminal β-gal levels (**B**) and α-2,6SA (**C**) were assessed using indicated lectin staining. Representative images of membrane-bound lectins acquired using the Operetta system; scale bar, 100 μm. Result is the average of three independent experiments and mean ± SD are provided.

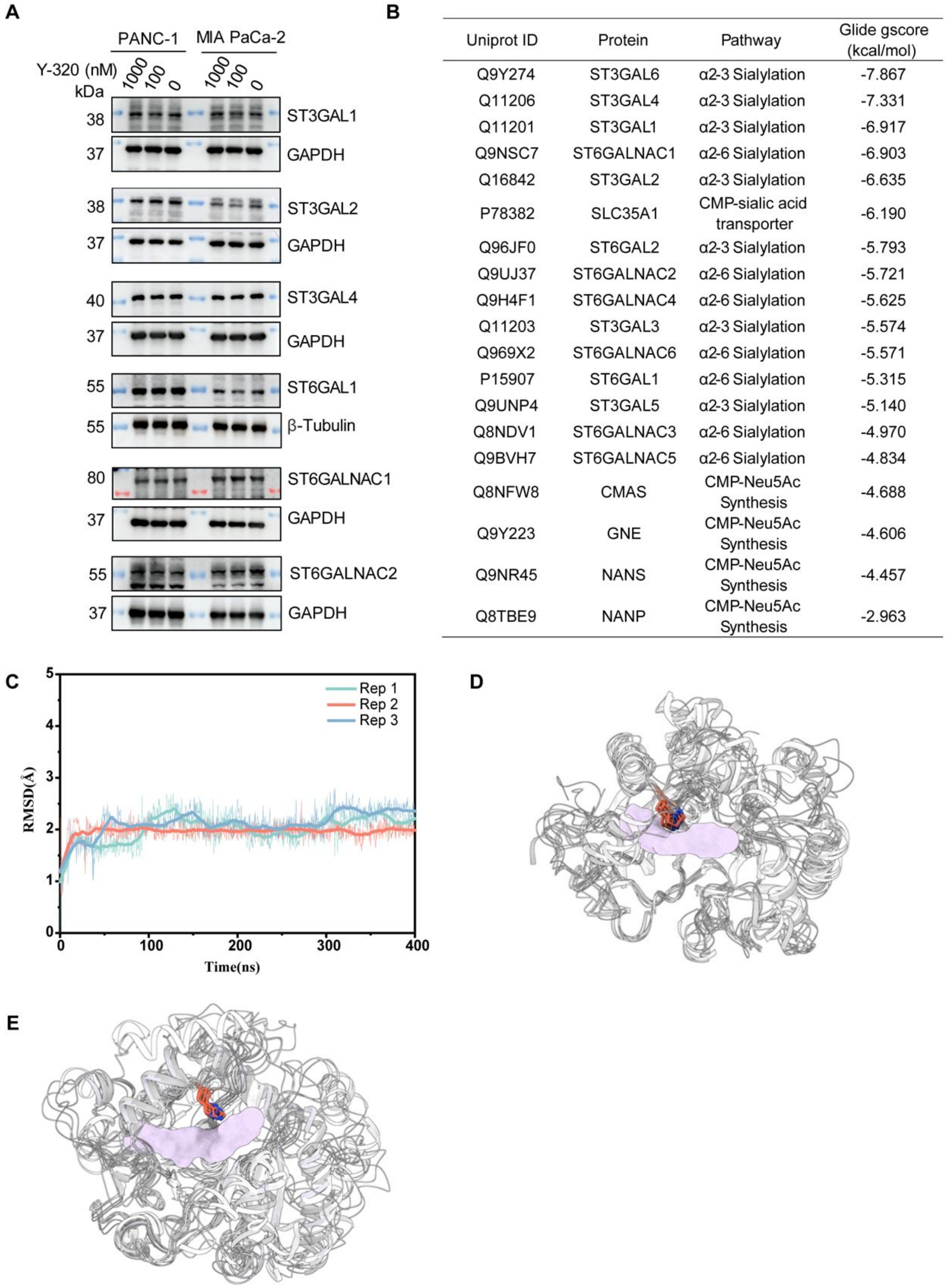

Uniprot ID	Protein	Pathway	Glide gscore (kcal/mol)
Q9Y274	ST3GAL6	α2-3 Sialylation	-7.867
Q11206	ST3GAL4	α2-3 Sialylation	-7.331
Q11201	ST3GAL1	α2-3 Sialylation	-6.917
Q9NSC7	ST6GALNAC1	α2-6 Sialylation	-6.903
Q16842	ST3GAL2	α2-3 Sialylation	-6.635
P78382	SLC35A1	CMP-sialic acid transporter	-6.190
Q96JF0	ST6GAL2	α2-3 Sialylation	-5.793
Q9UJ37	ST6GALNAC2	α2-6 Sialylation	-5.721
Q9H4F1	ST6GALNAC4	α2-6 Sialylation	-5.625
Q11203	ST3GAL3	α2-3 Sialylation	-5.574
Q969X2	ST6GALNAC6	α2-6 Sialylation	-5.571
P15907	ST6GAL1	α2-6 Sialylation	-5.315
Q9UNP4	ST3GAL5	α2-3 Sialylation	-5.140
Q8NDV1	ST6GALNAC3	α2-6 Sialylation	-4.970
Q9BVH7	ST6GALNAC5	α2-6 Sialylation	-4.834
Q8NFW8	CMAS	CMP-Neu5Ac Synthesis	-4.688
Q9Y223	GNE	CMP-Neu5Ac Synthesis	-4.606
Q9NR45	NANS	CMP-Neu5Ac Synthesis	-4.457
Q8TBE9	NANP	CMP-Neu5Ac Synthesis	-2.963

Figure S4. The reverse molecular docking and molecular dynamics simulation to predict the potential binding targets of Y-320 among the enzymes related to sialylation. (A) Immunoblot analysis of ST3Gal1, ST3Gal2, ST3Gal4, ST6Gal1, ST6GalNAc2 in PANC-1 cells treated with or without Y-320. **(B)** Docking scores of Y-320 to sialyltransferases and related enzymes across different sialylation pathways. **(C)** RMSD of Y-320 across four replicate 400 ns MD simulations with ST3GAL4. **(D)** Overlay of docking poses for ST3GAL1-6 (light to dark). The illustrative position of Y-320 is shown in pink, and the conserved histidine is depicted as orange sticks. **(E)** Overlay of docking poses for ST6Gal1-2 and ST6GalNac1-6 (light to dark) with the same color scheme.

A

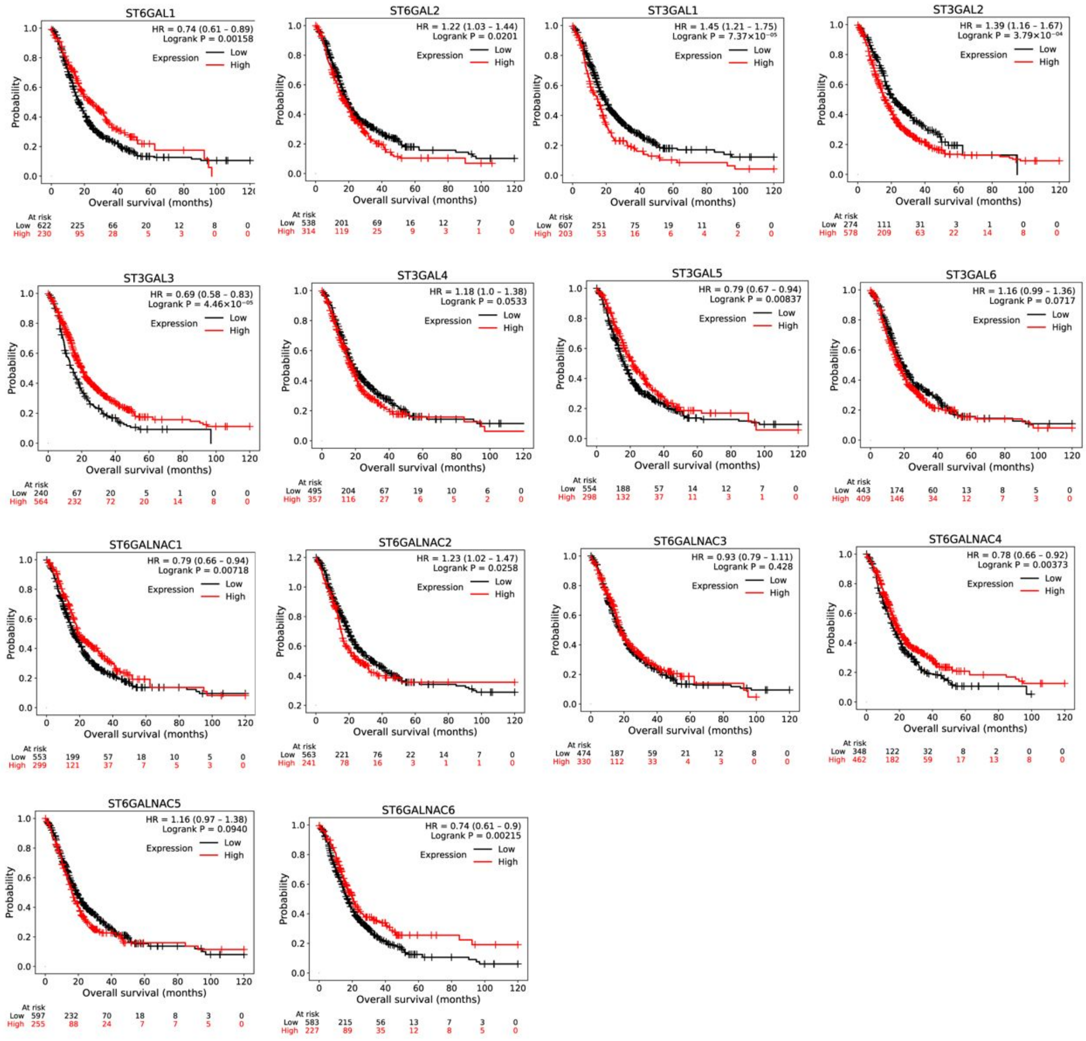

Figure S5. The correlation between sialyltransferase expression and prognosis of PDAC patients. (A) Kaplan-Meier survival analysis of PDAC patients stratified by sialyltransferase expression using data from TCGA.

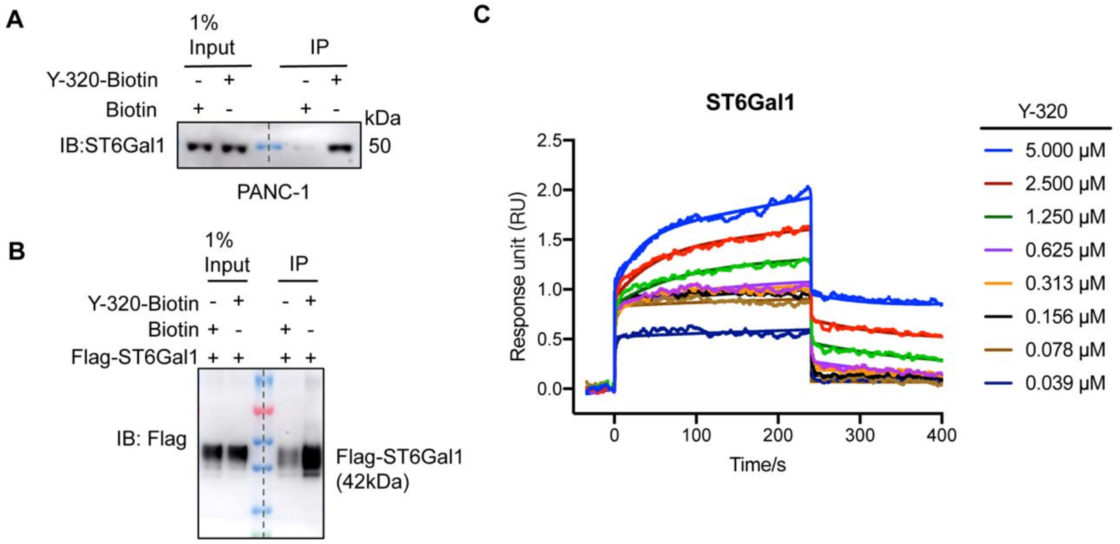

Figure S6. Binding affinity of Y-320 to ST6Gal1 quantified using biotinylated Y-320. (A) Co-immunoprecipitation (Co-IP) of endogenous ST6Gal1 from PANC-1 cell lysates using Y-320-Biotin. IB, immunoblot; IP, immunoprecipitation. **(B)** Pull down analysis of the interactions between Y-320 and his-tagged human ST6Gal1, using Y-320-Biotin immobilized on streptavidin beads. **(C)** Surface plasmon resonance (SPR) analysis of the interaction between Y-320 and human ST6Gal1. Binding kinetics: Ka=6.14×10^{3} $(Ms)^{-1}$, Kd=5.44×10^{-3} s^{-1}, and KD=885 nM.

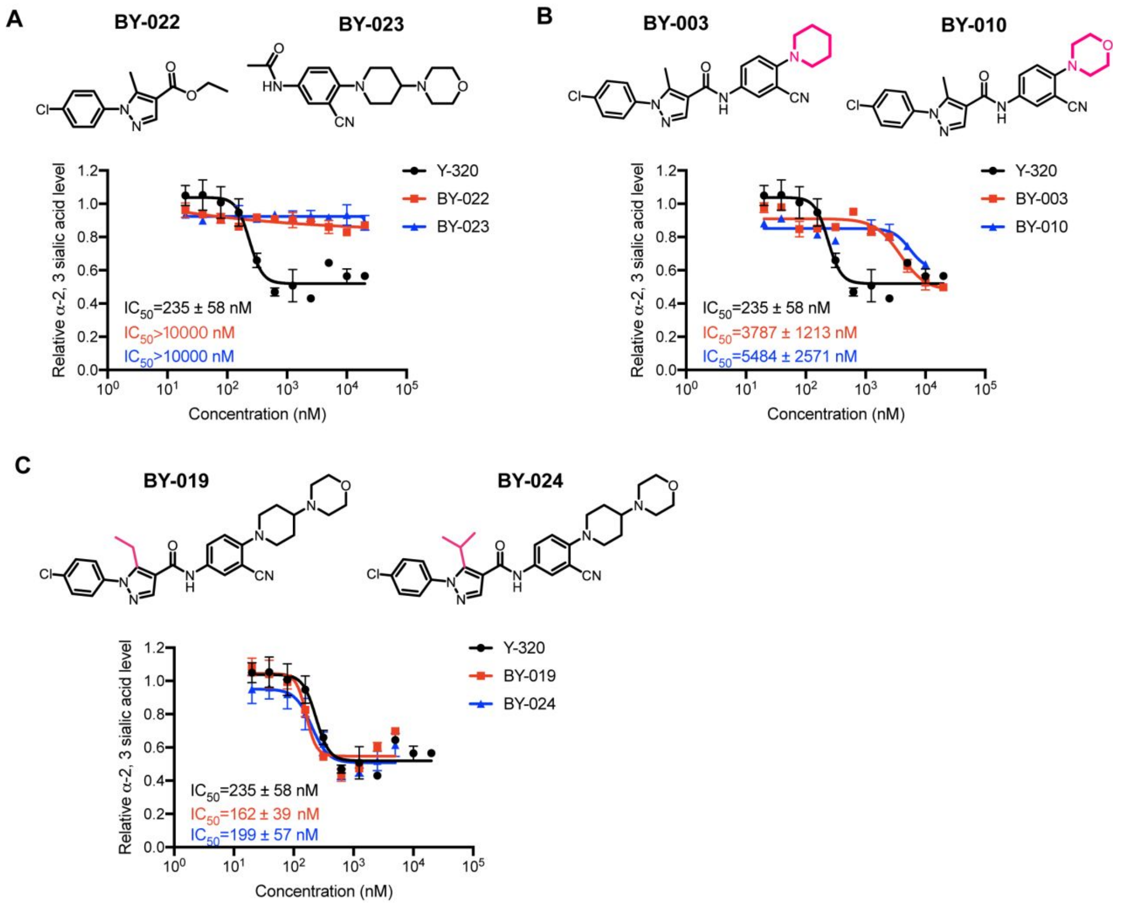

Figure S7. Mechanism of action (MOA) of Y-320 on ST3Gal1. (A-C) Chemical structures and inhibitory activities of additional Y-320 analogs. Inhibition of α-2,3 sialylation was assessed using MALII lectin staining. Result is the average of three independent experiments and mean ± SD are provided.

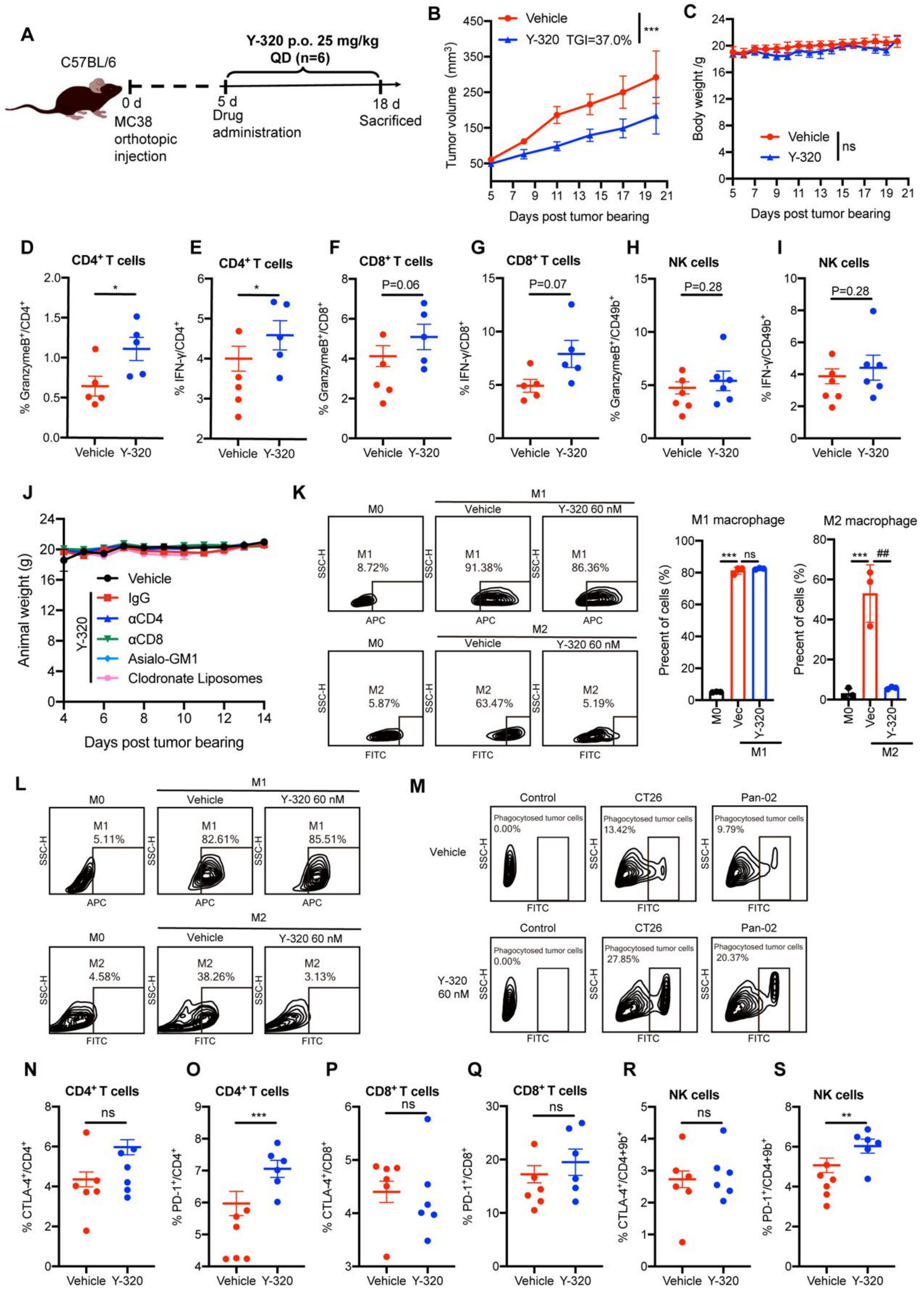

Figure S8. Y-320 enhances antitumor immunity *in vivo*. (A) Experimental design for MC38 tumor treatment. Mice bearing MC38 tumors were orally administered Y-320 (25 mg/kg) daily (n=8 per group). **(B)** Tumor volumes in mice treated with 25 mg/kg Y-320 or solvent control. **(C)** Body weight changes in mice during treatment.

(D-E) Flow cytometry analysis of the frequency of Granzyme B (**D**) or INF-γ^+ (**E**) $CD4^+$ T cells in dLN. **(F-G)** Flow cytometry analysis of the frequency of Granzyme B (**F**) or INF-γ^+ (**G**) $CD8^+$ T cells in dLN. **(H-I)** Flow cytometry analysis of the frequency of Granzyme B (**H**) or INF-γ^+ (**I**) NK cells in dLN. **(J)** CT26 tumor-bearing mice were pretreated with anti-CD4 and anti-CD8 antibodies, anti-Asialo-GM1 antibody, or clodronate liposomes, followed by oral administration of Y-320. Body weight changes in mice during the experiment. **(K-L)** Flow cytometry analysis of bone marrow-derived macrophage (**K**) and peritoneal macrophages (**L**) differentiation *in vitro*, demonstrating the effects of 60 nM Y-320 treatment on macrophage polarization markers. **(M)** Flow cytometry analysis of macrophage phagocytosis against tumor cells. **(N-S)** Mice bearing CT26 tumors were orally administered Y-320 25 mg/kg daily (n=6 per group). Flow cytometry analysis of the frequency of CTLA-4^+ (**N**) or PD-1^+ (**O**) $CD4^+$ T cells in dLN. Flow cytometry analysis of the frequency of CTLA-4^+ (**P**) or PD-1^+ (**Q**) $CD8^+$ T cells in dLN. Flow cytometry analysis of the frequency of CTLA-4^+ (**R**) or PD-1^+ (**S**) NK cells in dLN. Mean ± SEM are provided in **B-G**, **N-S**. Mean ± SD are provided in **K**. Statistical analysis was performed using a two-way ANOVA for **B**, **C**, **J** and a Student's unpaired two-tailed t-test in **D-I**, **N-S**; and a one-way ANOVA for **K**. *, $P < 0.05$; **, $P < 0.01$; ***, $P < 0.001$ and $^{\#\#}$, $P < 0.01$ as indicated.

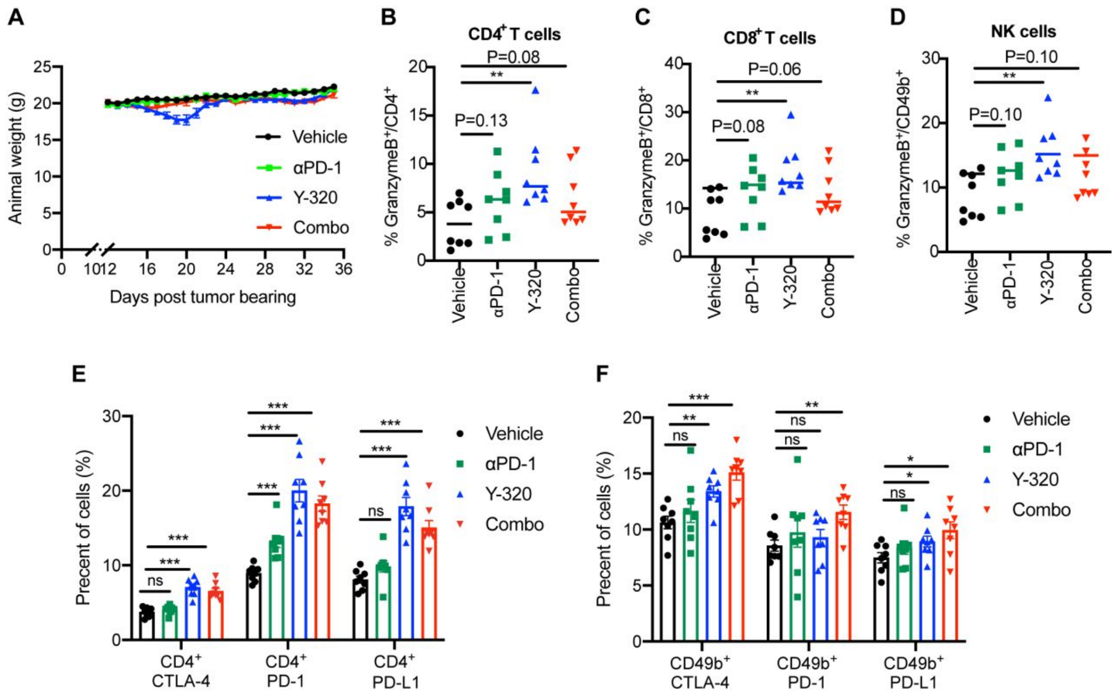

Figure S9. Y-320 enhances the effect of PD-1 antibody in Pan-02 model. (A) Mice bearing Pan-02 tumors were treated with PD-1 antibody (150 μg, intravenously every 4 days) and/or 25 mg/kg Y-320 (orally daily) (n=8 per group). Body weight changes in mice during treatment. **(B-D)** Flow cytometry analysis of the frequency of Granzyme B^+ $CD4^+$ T cells (**B**), Granzyme B^+ $CD8^+$ T cells (**C**) or Granzyme B^+ NK cells (**D**) in dLN. **(E)** Flow cytometry analysis of the frequency of CTLA-4^+, PD-1^+, and PD-$L1^+$ in $CD4^+$T cells in dLN. **(F)** Flow cytometry analysis of the frequency of CTLA-4^+, PD-1^+, and PD-$L1^+$ in NK cells in dLN. Mean ± SEM are provided in **A-F**. Statistical analysis was performed using a multiple t test in **B-F**. *, P < 0.05; **, P < 0.01; *** and P < 0.001 as indicated.

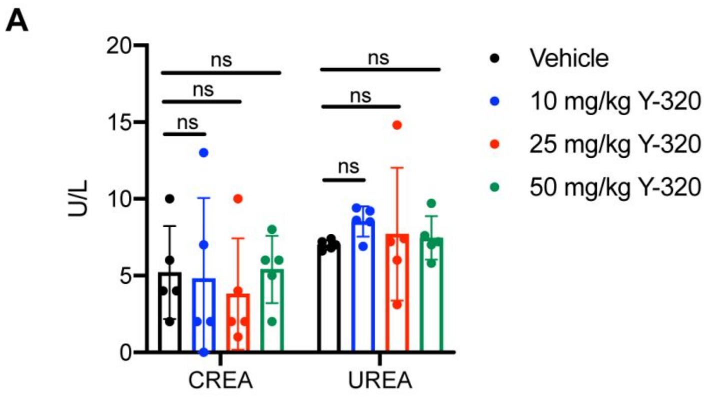

Figure S10. Y-320 did not affect kidney function. (A) The detection of serum kidney injury indicators UREA and CREA after Y-320 administration.

Supporting tables

Table S1. Primer sequences for RT-qPCR analysis

Gene	Direction	Sequence (5’-3’)
GAPDH	Forward	GGAGCGAGATCCCTCCAAAAT
	Reverse	GGCTGTTGTCATACTTCTCATGG
ST3Gal1	Forward	AAGAGGACCCTGAAAGTGCTC
	Reverse	CTCCAGGACCATCTGCTTGG
ST3Gal2	Forward	CGTCTGGACCCGAGAGAAC
	Reverse	GCCAGGCACTATCTGGAACA
ST3Gal3	Forward	TTGACTCCGCTGGACAAACA
	Reverse	AGTTTGCGTACTTGGTGGCT
ST3Gal4	Forward	GGGAAGACAGGTACATCGAGC
	Reverse	AATCCTCAAGCCGCAGGAAG
ST3Gal5	Forward	TGATACTGGGTCTGCGATGC
	Reverse	CCCTGGTTGGTTCTCGAGTC
ST3Gal6	Forward	ACTGCATTGCATATTATGGGGAA
	Reverse	TGGCTTTGATAAACAAGGCTGG
ST6GalNAc1	Forward	AGAAAGGTCTCTACAGTCCCTG
	Reverse	TGTGTGTTGAGGGCATTGTTC
ST6GalNAc2	Forward	ACTTCCGTGGCCTGTTCAAT
	Reverse	CATACGGGGCTTTGTGTTGG
ST6GalNAc3	Forward	AGTTGGTATCTATCCGAATGCCC
	Reverse	TGTACTGTCCTTCCCAGTTTCC
ST6GalNAc4	Forward	TCGTCTCACACACAAGCGTG
	Reverse	TGGTCGCAGTAGGCCATCA
ST6GalNAc5	Forward	CACTGGCTGGTTTACAATGACA
	Reverse	GTCCTCGCTCATGGGAGAG
ST6GalNAc6	Forward	TGATACTGGGTCTGCGATGC
	Reverse	CCCTGGTTGGTTCTCGAGTC
ST6Gal1	Forward	AACTCTCAGTTGGTTACCACAGA
	Reverse	GGTGCAGCTTACGATAAGTCTT
ST6Gal2	Forward	AAGGGGAACGTCTCTTCCAAA
	Reverse	CTTGTTGGCGGTCAGGTAATC
CMAS	Forward	ACCTGGCAGCCCTAATTCTG
	Reverse	TCGAAACCCATACACTCTGGAA
GNE	Forward	GACTTTGACATTAACACCAGGCT
	Reverse	GCTTCAGGCGATTAAGGACATCT
NANP	Forward	AAAGGTGGTGCAGCCAATAGA
	Reverse	TCTGTCTGTCCCCATTCGTTAAT
NANS	Forward	AACACCCGTGCTTCATCATTG
	Reverse	GGATCATGCGCTTGGCTAC

SLC5A1	Forward	CAACCACAGCCGTGTGTATCA
	Reverse	TGCTAAGAGCTAGGAAAGCCAT
NEU1	Forward	GGAGGCTGTAGGGTTTGGG
	Reverse	CACCAGACCGAAGTCGTTCT
NEU2	Forward	ACCCATGCCCCTTGTATGAC
	Reverse	TGCTGTTGCTCCGTGACTTG
NEU3	Forward	AAGTGACAACATGCTCCTTCAA
	Reverse	TCTCCTCGTAGAACGCTTCTC
NEU4	Forward	GGCCACGGGATGACAGTTG
	Reverse	CAGGCGGATACCCATGTGTAG

NOTE. Glyceraldehyde-3-phosphate dehydrogenase (GAPDH) was amplified as an internal control.

Table S2. Antibody information for Western blotting

Antibody	Brand	Catalog Number
Anti-ST3Gal1	Abcam	ab96129
Anti-ST3Gal2	Santa Cruz	sc-100856
Anti-ST3Gal4	ProteinTech	13546-1-AP
Anti-ST6Gal1	Invitrogen	MA5-11900
Anti-ST6GalNAc2	Thermo	PA5-31353
Anti-GAPDH	ProteinTech	60004-1-lg
Anti-β-Tubulin	ProteinTech	66240-1-lg
Anti-His	Santa Cruz	sc-8036
Anti-Flag	Sigma	F3156

Table S3. Antibody information for flow cytometry analysis

Antibody	Fluorescence	Brand	Catalog Number
Anti-mouse CD3	APC-Cy7	BD Pharmingen	557596
Anti-mouse CD4	BV421	BD Pharmingen	562891
Anti-mouse CD8a	BV510	BD Pharmingen	563068
Anti-mouse CD49b	FITC	BD Pharmingen	553857
Anti-mouse CD279	BV786	BD Pharmingen	744548
Anti-mouse CD274	BV650	BD Pharmingen	740614
Anti-mouse CD152	BV605	BioLegend	106323
Anti-mouse IFN-γ	BV786	BD Pharmingen	563773
Anti-mouse TNF-α	BV650	BD Pharmingen	563943
Anti-mouse Granzyme B	PE-Cy7	Invitrogen	25-8898-82
Anti-mouse Perforin	PE	Invitrogen	12-9392-80

Anti-mouse CD45	APC-Cy7	BioLegend	109824
Anti-mouse CD11b	BV421	BioLegend	101235
Anti-mouse CD11c	BV786	BD Pharmingen	563735
Anti-mouse CD86	APC	BD Pharmingen	558703
Anti-mouse CD206	AF488	BD Pharmingen	568806
Anti-mouse F4/80	PE-Cy7	Invitrogen	25-4801-82

Synthesis and characterization of compounds

Reagents were purchased at the highest commercial quality and used without further purification, unless otherwise stated. Ethyl acetate (EA, ACS grade), petroleum ether (PE, ACS grade), methanol (ACS grade) were purchased from Shanghai Titan Scientific and used without further purification. Isopropanol (*i*-PrOH) were dried with activated Linde types 4Å molecular sieves and stored under an argon atmosphere. Anhydrous N,N-dimethylformamide (DMF), methylene chloride (CH_2Cl_2), and tetrahydrofuran (THF) were purchased from J&K Scientific with molecular sieves and were used without further purification. Reactions were monitored by thin layer chromatography (TLC) carried out on MilliporeSigma glass TLC plates (silica gel 60 coated with F254, 250 μm) visualized by UV light or staining with $KMnO_4$, followed by heating with heat-gun. Flash column chromatography was performed over SiliaFlash® P60 silica gel (200-300 mesh). ^{1}H NMR and ^{13}C NMR spectra were recorded on Bruker 400 MHz and 600 MHz, Agilent 500 MHz spectrometers using solvent residual peaks as internal standards ($CDCl_3$, δ = 7.26 ppm (^{1}H) and 77.16 ppm (^{13}C); DMSO-d6, δ = 2.50 ppm (^{1}H) and 39.52 ppm (^{13}C)). The following abbreviations are used to designate multiplicities: s = singlet, d = doublet, t = triplet, q = quartet, m = multiplet, br = broad. Structural assignments were made with additional information from gHMBC experiments. High-resolution mass spectra were recorded with Shimadzu Biotech Axima Performance FTMS, maXis 4G FTMS, Thermo Scientific Q Exactive HF Orbitrap-FTMS, or Agilent-TOF/LC-MS 1260-6230 FTMS. Optical rotations were measured by an Anton Paar MCP 5500 polarimeter using the sodium D lines (589 nm).

Scheme 1：Synthesis of 5 (Y-320)

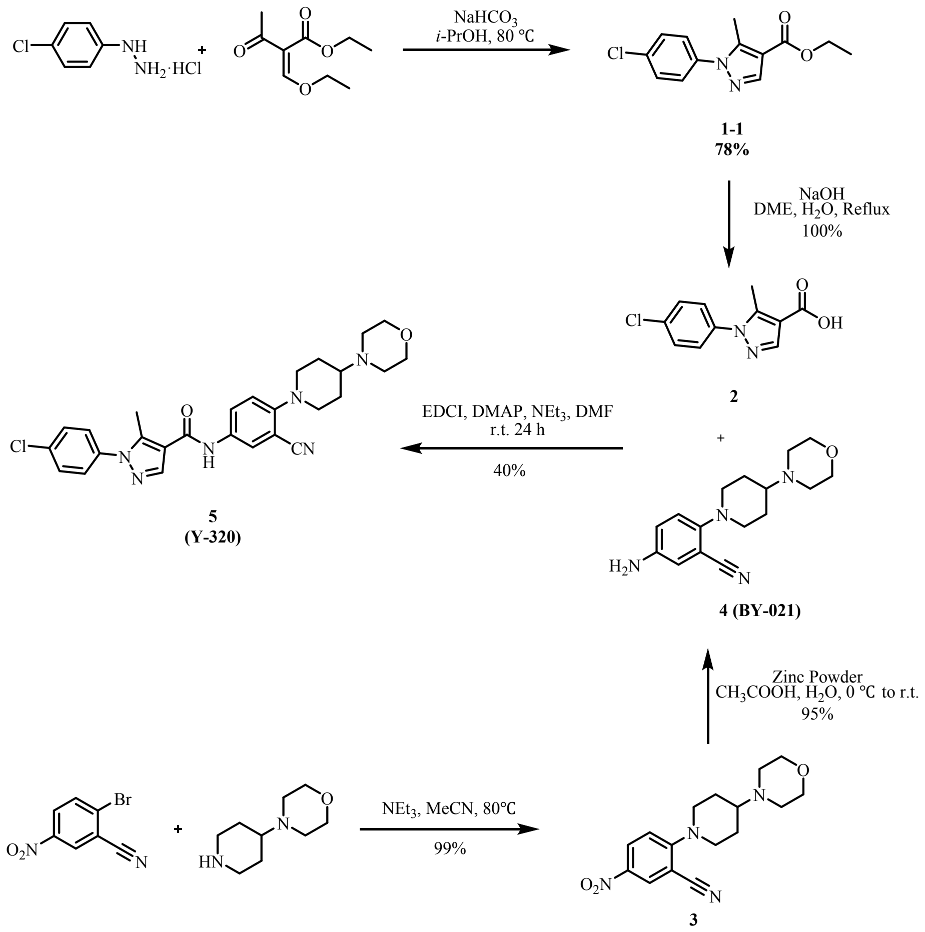

Compound 1 (BY-022):

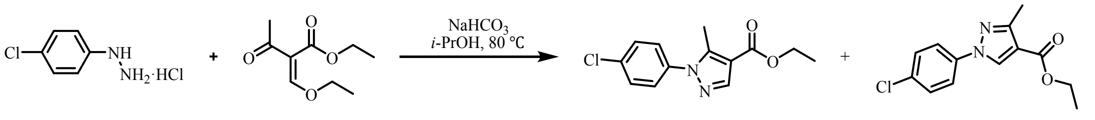

1-1 (BY-022)
78%

1-2
7%

To a 10 mL reaction tube 4-chlorophenylhydrazine hydrochloride (300 mg, 1.68 mmol, 1.0 equiv) and $NaHCO_3$ (140 mg, 1.68 mmol, 1.0 equiv) were added, followed by addition of 3.2 mL *i*-PrOH and 2-ethoxymethylene-3-oxobutanoic acid ethyl ester (320 μL, 1.68 mmol, 1.0 equiv). After stirring at 80 ℃ overnight, TLC indicated the reaction was complete. The mixture was diluted with ethyl acetate, then washed with brine. The organic layer was dried over anhydrous Na_2SO_4, filtered, and concentrated under vacuum. The residue was purified by silica gel column chromatography (petroleum ether/ethyl acetate = 20:1) to afford compound **1-1** (344.8 mg, 78%) as a

yellowish solid and compound **1-2** (29.2 mg, 7%) as a white solid. Compound **1-1**: ^{1}H NMR (500 MHz, DMSO-d6) δ 8.02 (s, 1H), 7.63 (d, *J* = 8.8 Hz, 2H), 7.59 (d, *J* = 8.8 Hz, 2H), 4.25 (q, *J* = 7.1 Hz, 2H), 2.52 (s, 3H), 1.29 (t, *J* = 7.1 Hz, 3H); ^{13}C NMR (151 MHz, DMSO-d6) δ 163.30, 144.10, 141.97, 137.78, 133.62, 129.81, 127.54, 112.92, 60.12, 14.76, 12.02; HRMS (ESI-TOF) m/z $[M+H]^+$ calcd for $C_{13}H_{14}ClN_2O_2$ 265.0738, found 265.0746. Compound **1-2**: ^{1}H NMR (500 MHz, DMSO-d6) δ 9.01 (s, 1H), 7.94 (d, *J* = 8.8 Hz, 2H), 7.56 (t, *J* = 8.9, 6.7 Hz, 2H), 4.25 (q, *J* = 7.1 Hz, 2H), 2.44 (s, 3H), 1.30 (t, *J* = 7.1 Hz, 3H); ^{13}C NMR (151 MHz, DMSO-d6) δ 163.11, 151.86, 138.09, 132.73, 131.55, 129.93, 120.87, 114.34, 60.24, 14.76, 13.84; HRMS (ESI-TOF) m/z $[M+H]^+$ calcd for $C_{13}H_{14}ClN_2O_2$ 265.0738, found 265.0746.

Compound 2 (BY-020):

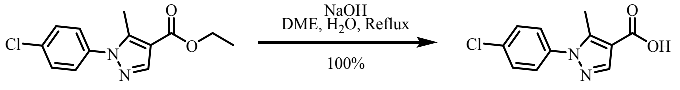

1-1 2 (BY-020)

NaOH (34 mg, 849 μmol, 2.0 equiv) was dissolved in 2.5 mL H_2O, then added to a stirring solution of compound **1-1** (150 mg, 566 μmol, 1.0 equiv) in 2.5 mL DME. The resulting mixture was heated to 80 ℃ until TLC indicated the reaction was complete. The mixture was cool to room temperature, 5 mL petroleum ether was added and stirring continued vigorously for 2 min. The organic phase was separated, water phase was acidified with 1N HCl until pH = 7, white precipitant formed. The water phase was extracted with ethyl acetate, then washed with brine. The organic layer was dried over anhydrous Na_2SO_4, filtered, and concentrated under vacuum to afford compound **2** (133.8 mg, 100%) as a white solid: ^{1}H NMR (500 MHz, DMSO-d6) δ 12.48 (br, 1H), 7.96 (s, 1H), 7.61 (d, *J* = 8.9 Hz, 2H), 7.57 (d, *J* = 8.8 Hz, 2H), 2.49 (s, 3H); ^{13}C NMR (126 MHz, DMSO-d6) δ 164.89, 143.91, 142.33, 137.89, 133.44, 129.76, 127.46, 113.68, 11.94; HRMS (ESI-TOF) m/z $[M+H]^+$ calcd for $C_{11}H_{10}ClN_2O_2$ 237.0425, found 2337.0428.

Compound 3:

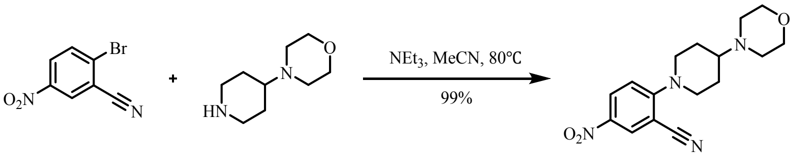

3

2-bromo-5-nitrobenzonitrile (300 mg, 1.32 mmol, 1.0 equiv) and 4-(piperidin-4-yl) morpholine (270 mg, 1.584 mmol, 1.3 equiv) were dissolved with 2.6 mL MeCN. Triethylamine (550 µL, 3.96 mmol, 3 equiv) was then added, the mixture was stirred at 80 ℃ for about 4 h until TLC indicated the reaction was complete. The mixture was diluted with DCM, washed with brine. The organic layer was dried over anhydrous Na_2SO_4, filtered, and concentrated under vacuum. The residue was purified by silica gel column chromatography (ethyl acetate/methanol = 1:0 to 20:1) to afford compound **3** (412.3 mg, 99%) as a light yellow solid: ^{1}H NMR (500 MHz, DMSO-d6) δ 8.50 (d, *J* = 2.8 Hz, 1H), 8.26 (dd, *J* = 9.5, 2.8 Hz, 1H), 7.24 (d, *J* = 9.5 Hz, 1H), 3.95 (d, *J* = 13.0 Hz, 2H), 3.60 – 3.55 (m, 4H), 3.17 – 3.08 (m, 2H), 2.51 – 2.39 (m, 6H), 1.93 (d, *J* = 13.2 Hz, 2H), 1.60 – 1.47 (m, 2H); ^{13}C NMR (151 MHz, DMSO-d6) δ 157.29, 137.62, 130.83, 128.38, 117.72, 116.67, 99.18, 65.95, 59.53, 48.96, 48.84, 27.29; HRMS (ESI-TOF) m/z $[M+H]^+$ calcd for $C_{16}H_{21}ClN_4O_3$ 317.1608, found 317.1614.

Compound 4 (BY-021):

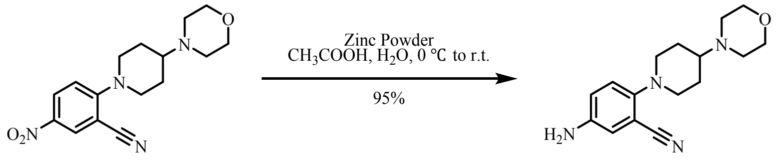

3 **4**

Compound **3** (150 mg, 474 µmol, 1.0 equiv) was dissolved with 6 mL acetic acid and 1.5 mL H_2O. The mixture was cooled with ice-bath, zinc powder (310 mg, 4.74 mmol, 10.0 equiv) was added portion-wise. After adding, the mixture was stirring at 0 ℃ for 5 min, then warm to room temperature slowly, yellow solution faded slowly as reaction went. The mixture was stirring at room temperature for about 30 min until TLC indicated the reaction was complete. The volatile compounds were removed under

vacuum Remain crude was diluted with ethyl acetate, then washed with 2N KOH solution, then washed with brine. The organic layer was dried over anhydrous Na_2SO_4, filtered, and concentrated under vacuum to afford compound 4 (128.7 mg, 95%) as a pink solid, used directly in next step without further purification. Compound **4**: ^{1}H NMR (500 MHz, DMSO-d6) δ 6.96 (d, J = 9.4 Hz, 1H), 6.84 – 6.78 (m, 2H), 5.20 (br, 2H), 3.62 – 3.57 (m, 4H), 3.25 – 3.18 (m, 2H), 2.68 – 2.59 (m, 2H), 2.29 – 2.19 (m, 1H), 1.91 – 1.84 (m, 2H), 1.55 (qd, J = 11.9, 3.8 Hz, 2H); ^{13}C NMR (151 MHz, DMSO-d6) δ 146.27, 144.90, 121.13, 120.01 (d, J = 2.7 Hz), 118.86, 117.68, 107.45, 67.06, 61.27, 52.65, 49.93, 28.83; HRMS (ESI-TOF) m/z $[M+H]^+$ calcd for $C_{16}H_{23}N_4O$ 287.1866, found 287.1873.

Compound 5 (Y-320):

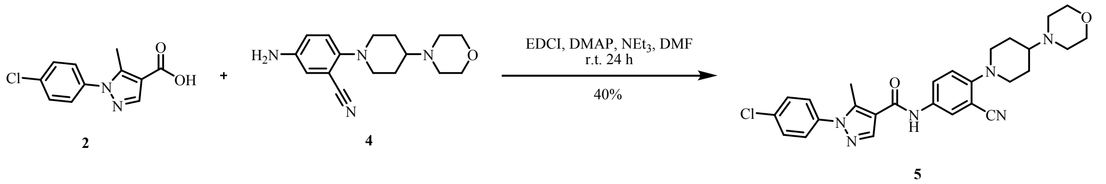

Compound **2** (15 mg, 63.38 μmol, 1.0 equiv), compound **4** (18 mg, 63.38 μmol, 1.0 equiv), EDCI (15 mg, 76 μmol, 1.2 equiv) and DMAP (1.1 mg, 9.5 μmol, 0.15 equiv) was dissolved with 600 μL anhydrous DMF in a 10 mL reaction seal tube, triethylamine (18 μL, 126.7 μmol, 2.0 equiv) was then added. The mixture was stirred at room temperature for 24 h. Then 1 mL methanol, 1 mL 10% K_2CO_3 water solution, 4 mL H_2O were added to the mixture. Pink precipitant was formed, then dispersed with ultrasonic. The precipitant was collected by centrifuging with 3000 rpm for 5 min, washed with 5 mL H_2O once to afford compound **5** (12.8 mg, 40%) as a white solid: ^{1}H NMR (500 MHz, DMSO-d6) δ 9.99 (s, 1H), 8.31 (s, 1H), 8.07 (d, J = 2.6 Hz, 1H), 7.84 (dd, J = 9.0, 2.6 Hz, 1H), 7.64 (d, J = 8.9 Hz, 2H), 7.60 (d, J = 8.7 Hz, 2H), 7.19 (d, J = 9.0 Hz, 1H), 3.59 (t, J = 4.5 Hz, 4H), 3.47 (d, J = 11.7 Hz, 2H), 2.81 – 2.71 (m, 2H), 2.55 (s, 3H), 2.51 (s, 4H), 2.33 – 2.25 (m, 1H), 1.91 (d, J = 12.1 Hz, 2H), 1.62 – 1.52 (m, 2H); ^{13}C NMR (126 MHz, DMSO-d6) δ 161.89, 151.91, 143.22, 139.97,

137.87, 133.82, 133.39, 129.77, 127.45, 126.47, 125.19, 120.13, 118.50, 115.95, 105.37, 67.03, 61.09, 51.77, 49.91, 28.59, 12.01; HRMS (ESI-TOF) m/z $[M+H]^+$ calcd for $C_{27}H_{30}ClN_6O_2$ 505.2113, found 505.2115.

Scheme 2: Synthesis of 11 (Y-320 Biotin)

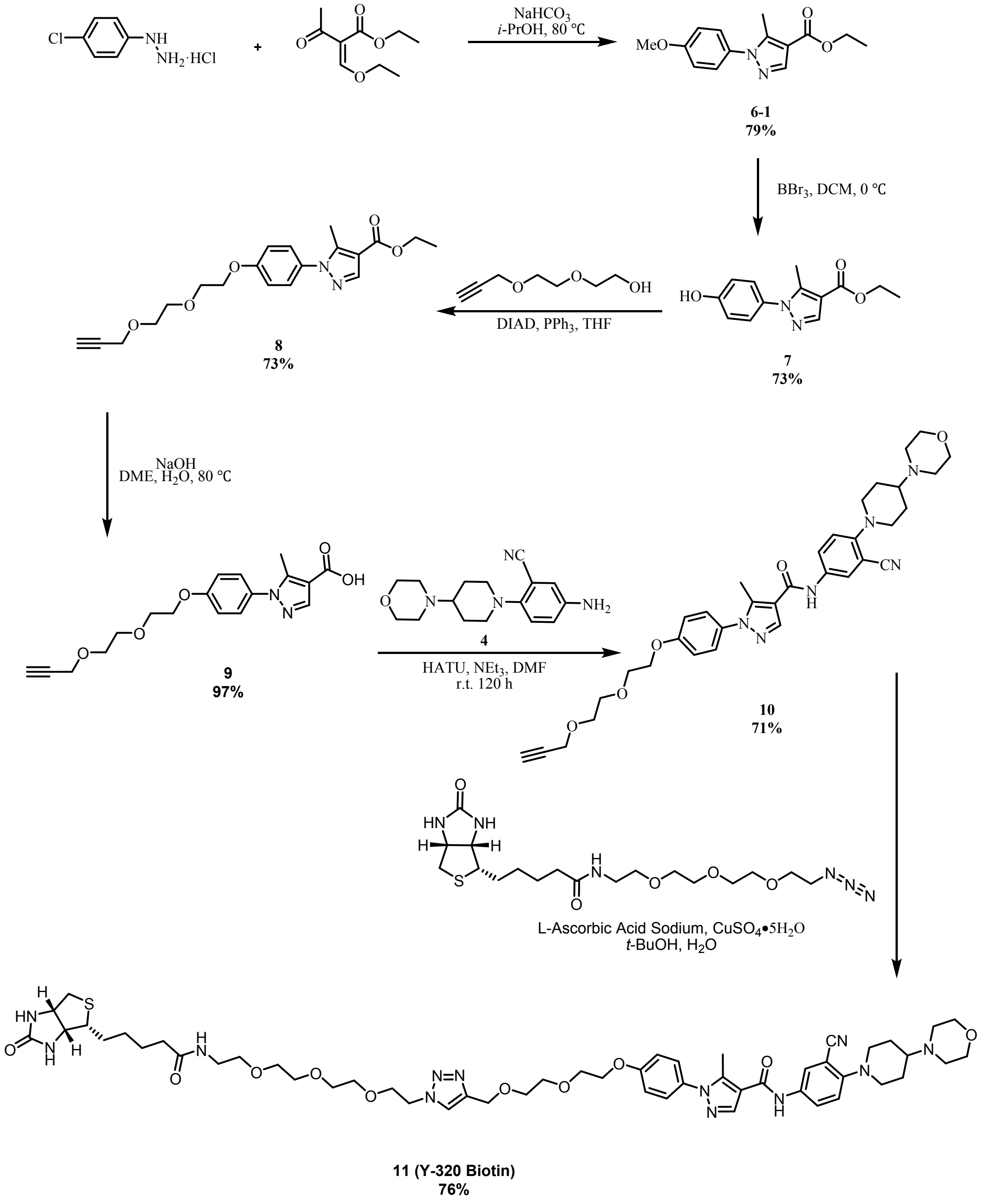

Compound 6:

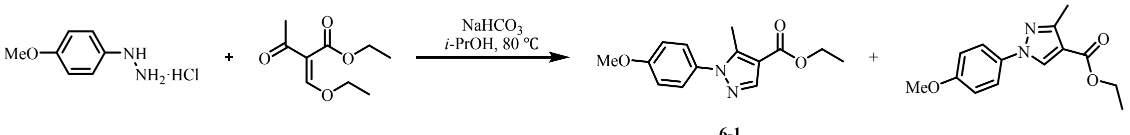
6-1
79%
6-2
5%

To a 20 mL reaction tube 4-methoxyphenylhydrazine hydrochloride (800 mg, 4.58 mmol, 1.0 equiv) and $NaHCO_3$ (384 mg, 4.58 mmol, 1.0 equiv) were added, followed by addition of 8 mL *i*-PrOH and 2-ethoxymethylene-3-oxobutanoic acid ethyl ester (1.05 mL, 5.49 mmol, 1.2 equiv). After stirring at 80 °C overnight, TLC indicated the reaction was complete. The reaction was diluted with ethyl acetate, then washed with brine. The organic layer was dried over anhydrous Na_2SO_4, filtered, and concentrated under vacuum. The residue was purified by silica gel column chromatography (petroleum ether/ethyl acetate = 15:1 to 10:1) to afford compound **6-1** (928.5 mg, 79%) as a yellowish solid, and compound **6-2** (64.5 mg, 5%) as an orange solid. Compound **6-1**: ^{1}H NMR (500 MHz, $CDCl_3$) δ 7.99 (s, 1H), 7.31 (d, J = 8.9 Hz, 2H), 6.98 (d, J = 8.8 Hz, 2H), 4.31 (q, J = 7.1 Hz, 2H), 3.85 (s, 3H), 2.50 (s, 3H), 1.36 (t, J = 7.1 Hz, 3H); ^{13}C NMR (126 MHz, $CDCl_3$) δ 163.88, 159.62, 143.59, 141.59, 131.84, 126.88, 114.33, 112.57, 59.89, 55.56, 14.44, 11.82; HRMS (ESI-TOF) m/z $[M+H]^+$ calcd for $C_{14}H_{17}N_2O_3$ 261.1234, found 261.1239. Compound **6-2**: ^{1}H NMR (400 MHz, $CDCl_3$) δ 8.24 (s, 2H), 7.57 (d, J = 9.0 Hz, 4H), 6.96 (d, J = 8.9 Hz, 4H), 4.31 (q, J = 7.1 Hz, 4H), 3.84 (s, 3H), 2.55 (s, 5H), 1.37 (t, J = 7.1 Hz, 6H); ^{13}C NMR (126 MHz, $CDCl_3$) δ 163.64, 158.67, 152.02, 132.99, 131.06, 120.98, 114.54, 113.80, 60.01, 55.54 (d, J = 1.7 Hz), 14.41, 13.58; HRMS (ESI-TOF) m/z $[M+H]^+$ calcd for $C_{14}H_{17}N_2O_3$ 261.1234, found 261.1238.

Compound 7:

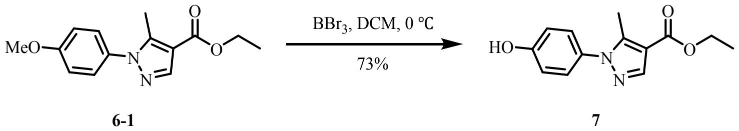
6-1
7

Compound **6** (50 mg, 192 μmol) was dissolved in 200 μL anhydrous CH_2Cl_2 under argon atmosphere. Then the mixture was cooled to -5 ℃, BBr_3 (1 M in DCM, 580 μL, 579 μmol, 3.0 equiv) was added dropwise by syringe. The resulting mixture was stirred at -5 ℃ overnight until TLC indicated the reaction was complete. Quenched with methanol slowly, concentrated under vacuum, then diluted with ethyl acetate, washed with brine. The organic layer was dried over anhydrous Na_2SO_4, filtered, and concentrated under vacuum. The residue was purified by silica gel column chromatography (petroleum ether/ethyl acetate = 2:1) to afford compound **7** (34.4 mg, 73%) as a white solid: ^{1}H NMR (500 MHz, $CDCl_3$) δ 8.01 (s, 1H), 7.21 (d, J = 8.8 Hz, 2H), 6.86 (d, J = 8.7 Hz, 2H), 4.32 (q, J = 7.1 Hz, 2H), 2.50 (s, 3H), 1.37 (t, J = 7.1 Hz, 3H); ^{13}C NMR (126 MHz, $CDCl_3$) δ 163.84, 157.44, 144.26, 141.34, 130.20, 127.17, 116.25, 112.55, 60.21, 14.40, 11.71; HRMS (ESI-TOF) m/z $[M+H]^+$ calcd for $C_{13}H_{15}N_2O_3$ 246.1077, found 247.1081.

Compound 8:

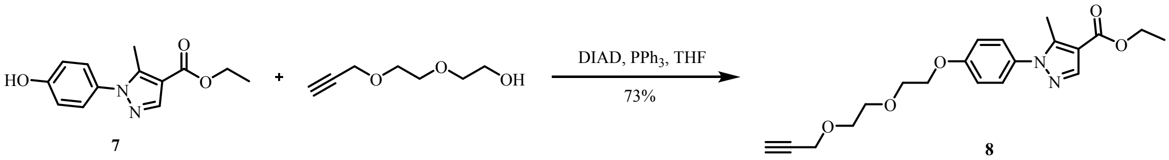

To a solution of compound **7** (333 mg, 134 μmol, 1.0 equiv), 2-(2-(prop-2-ynyloxy) ethoxy) ethanol (27 μL, 201 μmol, 1.5 equiv) and PPh_3 (53 mg, 201 μmol, 1.5 equiv) in 8 mL anhydrous THF, Diisopropyl azodicarboxylate (40 μL, 201 μmol, 1.5 equiv) was added dropwise by syringe under ice-bath. After adding, the mixture was warm to room temperature slowly. The resulting mixture was stirred at room temperature overnight until TLC indicated the reaction was complete. The crude was diluted with ethyl acetate, then washed with brine. The organic layer was dried over anhydrous Na_2SO_4, filtered, and concentrated under vacuum. The residue was purified by silica gel column chromatography (petroleum ether/ethyl acetate = 2:1) to afford compound **8** (36.6 mg, 73%) as a colorless oil: ^{1}H NMR (500 MHz, $CDCl_3$) δ 7.98 (s, 1H), 7.29 (d, J = 8.9 Hz, 2H), 7.00 (d, J = 8.9 Hz, 2H), 4.30 (q, J = 7.1 Hz, 2H), 4.21 (d, J = 2.3

Hz, 2H), 4.18 (dd, J = 5.6, 4.1 Hz, 2H), 3.91 – 3.85 (m, 2H), 3.79 – 3.70 (m, 4H), 2.43 (t, J = 2.4 Hz, 1H), 1.35 (t, J = 7.1 Hz, 3H); ^{13}C NMR (126 MHz, $CDCl_3$) δ 163.86, 158.85, 143.58, 141.59, 132.00, 126.84, 115.05, 112.59, 79.55, 74.61, 70.69, 69.64, 69.12, 67.79, 59.88, 58.45, 14.42, 11.81; HRMS (ESI-TOF) m/z $[M+H]^+$ calcd for $C_{20}H_{25}N_2O_5$ 373.1758, found 373.1763.

Compound 9:

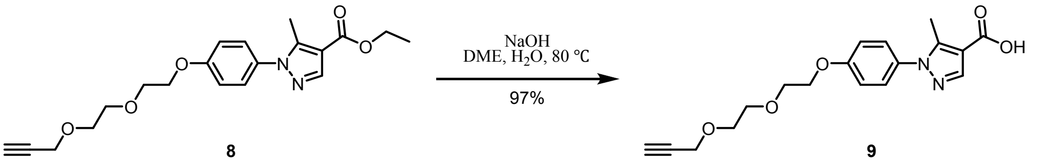

NaOH (6.5 mg, 160 μmol, 2.0 equiv) was dissolved in 0.5 mL water, then added to a stirring solution of compound **8** (30 mg, 80 μmol, 1.0 equiv) in 0.5 mL DME. The resulting mixture was heated to 80 ℃ until TLC indicated the reaction was complete. The mixture was cool to room temperature, 2 mL petroleum ether was added and stirring continued vigorously for 2 min. Organic phase was separated, water phase was acidified with 1N HCl until pH = 7, white precipitant formed. Water phase was extracted with ethyl acetate, then washed with brine. The organic layer was dried over anhydrous Na_2SO_4, filtered, and concentrated under vacuum to afford compound **9** (26.8 mg, 97%) as a white solid: ^{1}H NMR (500 MHz, $CDCl_3$) δ 8.07 (s, 1H), 7.31 (d, J = 8.9 Hz, 2H), 7.02 (d, J = 8.8 Hz, 2H), 4.22 (d, J = 2.4 Hz, 2H), 4.19 (t, J = 4.8 Hz, 2H), 3.89 (t, J = 4.8 Hz, 2H), 3.80 – 3.71 (m, 4H), 2.53 (s, 3H), 2.44 (t, J = 2.4 Hz, 1H); ^{13}C NMR (126 MHz, $CDCl_3$) δ 169.01, 158.98, 144.61, 142.35, 131.79, 126.90, 115.09, 111.88, 79.54, 74.63, 70.69, 69.64, 69.13, 67.80, 11.93; HRMS (ESI-TOF) m/z $[M+H]^+$ calcd for $C_{18}H_{21}N_2O_5$ 345.1445, found 345.1446.

Compound 10:

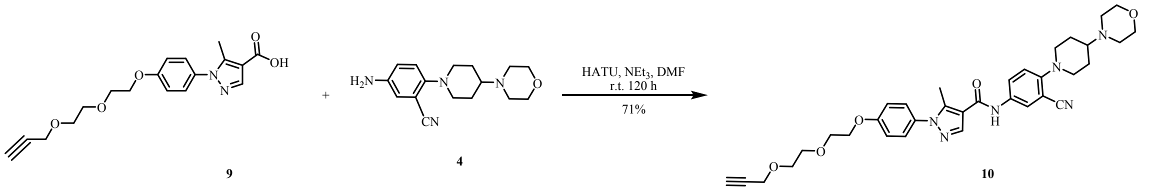

Compound **9** (17 mg, 50 μmol, 1.0 equiv), compound 4 (14 mg, 50 μmol, 1.0 equiv), and HATU (23 mg, 60 μmol, 1.2 equiv) were dissolved with 600 μL anhydrous DMF, in a 10 mL reaction seal tube, triethylamine (18 μL, 126.7 μmol, 2.0 equiv) was then added. The mixture was stirred at room temperature for 120 h. Then 1 mL 10% K_2CO_3 water solution, 4 mL H_2O were added to the mixture. The crude was diluted with ethyl acetate, then washed with brine. The organic layer was dried over anhydrous Na_2SO_4, filtered, and concentrated under vacuum. The residue was purified by silica gel column chromatography (DCM/methanol = 20:1) to afford compound **10** (21.2 mg, 71%) as a white solid: ^{1}H NMR (500 MHz, $CDCl_3$) δ 7.88 (s, 1H), 7.85 (s, 1H), 7.67 (dd, J = 9.0, 2.6 Hz, 1H), 7.43 (s, 1H), 7.34 – 7.28 (m, 2H), 7.04 – 6.99 (m, 3H), 4.22 (d, J = 2.4 Hz, 2H), 4.21 – 4.18 (m, 2H), 3.92 – 3.88 (m, 2H), 3.81 – 3.72 (m, 7H), 3.61 (d, J = 11.7 Hz, 2H), 2.81 (t, J = 11.7 Hz, 2H), 2.69 – 2.57 (m, 3H), 2.56 (s, 3H), 2.44 (t, J = 2.4 Hz, 1H), 2.05 – 1.92 (m, 2H), 1.81 (d, J = 12.6 Hz, 2H), 1.25 (s, 3H); ^{13}C NMR (151 MHz, $CDCl_3$) δ 160.92, 157.97, 151.28, 142.42, 136.81, 131.27, 130.75, 125.83, 125.13, 124.79, 118.41, 116.84, 114.09, 113.92, 105.57, 78.51, 73.63, 69.69, 68.64, 68.12, 66.79, 65.60, 60.99, 57.45, 50.63, 48.41, 26.87, 10.86; HRMS (ESI-TOF) m/z $[M+H]^+$ calcd for $C_{34}H_{41}N_6O_5$ 613.3133, found 613.3140.

Compound 11 (Y320-Biotin):

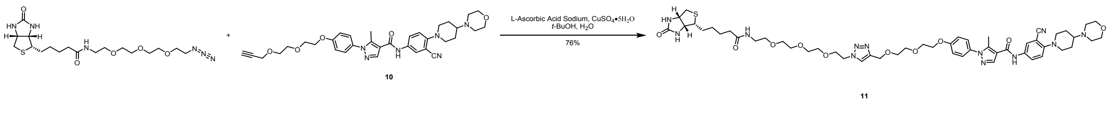

Biotin-PEG3-azide (7.26 mg, 16.32 μmol, 1.0 equiv), compound **10** (10 mg, 16.32 μmol, 1.0 equiv) and L-Ascorbic Acid Sodium Salt (3 mg, 16.32 μmol, 1.0 equiv) were suspended in 100 μL *t*-BuOH. Then $CuSO_4 \cdot 5H_2O$ (0.4 mg, 1.632 μmol, 0.1 equiv) was

dissolved in 100 μL H_2O and added to above suspension. The mixture was stirred at room temperature for 48 h until TLC indicated the reaction was complete. Crude was concentrated under vacuum, then purified by silica gel column chromatography (DCM/methanol = 12:1) to afford compound **11** (13 mg, 76%) as a white solid: $[\alpha]_D^{25}$ = 11.2 (c 0.1, $CHCl_3$); ^1H NMR (500 MHz, $CDCl_3$) δ 8.66 (s, 1H), 8.05 (s, 1H), 7.91 (d, *J* = 2.6 Hz, 1H), 7.81 (dd, *J* = 8.9, 2.7 Hz, 1H), 7.76 (s, 1H), 7.29 (d, *J* = 8.7 Hz, 2H), 7.00 (d, *J* = 8.8 Hz, 2H), 6.98 (d, *J* = 9.1 Hz, 1H), 6.70 (t, *J* = 5.6 Hz, 1H), 6.20 (s, 1H), 5.38 (s, 1H), 4.68 (s, 2H), 4.51 (t, *J* = 5.1 Hz, 2H), 4.46 – 4.41 (m, 1H), 4.29 – 4.23 (m, 1H), 4.17 (t, *J* = 4.6 Hz, 2H), 3.88 – 3.83 (m, 4H), 3.80 – 3.75 (m, 4H), 3.73 (s, 4H), 3.62 – 3.55 (m, 10H), 3.51 (t, *J* = 5.2 Hz, 2H), 3.38 (q, *J* = 5.4 Hz, 2H), 3.13 – 3.05 (m, 1H), 2.85 (dd, *J* = 12.8, 4.9 Hz, 1H), 2.78 (t, *J* = 11.7 Hz, 2H), 2.71 – 2.62 (m, 5H), 2.55 (s, 3H), 2.48 – 2.38 (m, 1H), 2.15 (td, *J* = 7.3, 3.3 Hz, 2H), 1.99 (d, *J* = 12.2 Hz, 2H), 1.85 – 1.73 (m, 2H), 1.63 (dddd, *J* = 25.8, 18.5, 12.8, 7.0 Hz, 4H), 1.39 (td, *J* = 15.8, 13.7, 6.0 Hz, 2H); ^{13}C NMR (151 MHz, $CDCl_3$) δ 173.36, 163.68, 162.23, 158.91, 152.18, 144.83, 143.25, 138.46, 132.85, 131.88, 126.86, 126.16, 125.75, 123.94, 119.31, 118.06, 115.26, 115.13, 106.42, 70.81, 70.49, 70.38, 70.32, 70.06, 69.83, 69.71, 69.63, 69.41, 67.88, 67.02, 64.59, 61.85, 61.76, 60.11, 55.45, 51.80, 50.18, 49.63, 40.51, 39.18, 35.83, 28.20, 28.13, 28.04, 25.50, 11.89; HRMS (ESI-TOF) m/z $[M+H]^+$ calcd for $C_{52}H_{73}N_{12}O_{10}S$ 1057.5288, found 1057.5288.

Compound 12 (BY-023):

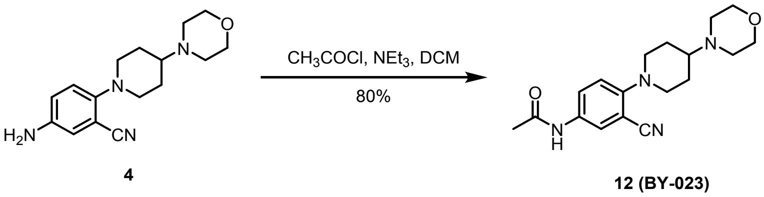

Compound **4** (30 mg, 104.7 μmol, 1.0 equiv) was dissolved in 1 mL anhydrous DCM, triethylamine (30 μL, 209.5 μmol, 2.0 equiv) was added. The mixture was cool to 0 °C, acetyl chloride (10 μL, 141.4 μmol, 1.35 equiv) was added dropwise by syringe, then warm to room temperature slowly. The mixture was stirred at room temperature

for about 2 h until TLC indicated the reaction was complete. The reaction was quenched with H_2O, extracted with ethyl acetate, then washed with brine. The organic layer was dried over anhydrous Na_2SO_4, filtered, and concentrated under vacuum. The residue was purified by silica gel column chromatography (DCM/methanol = 20:1) to afford compound **12** (27.6 mg, 80%) as a pink solid: ^1H NMR (400 MHz, $CDCl_3$) δ 7.95 (s, 1H), 7.71 (d, *J* = 2.6 Hz, 1H), 7.65 (dd, *J* = 8.9, 2.6 Hz, 1H), 6.96 (d, *J* = 8.9 Hz, 1H), 3.75 (t, *J* = 4.6 Hz, 4H), 3.56 (d, *J* = 11.7 Hz, 2H), 2.77 (t, *J* = 11.7 Hz, 2H), 2.61 (t, *J* = 4.6 Hz, 4H), 2.36 (ddd, *J* = 15.2, 7.5, 3.8 Hz, 3H), 2.16 (s, 3H), 1.97 (d, *J* = 12.5 Hz, 2H), 1.76 (qd, *J* = 12.0, 3.8 Hz, 2H); ^{13}C NMR (126 MHz, $CDCl_3$) δ 168.68, 152.54, 132.16, 125.88, 125.41, 119.36, 117.95, 106.23, 67.20, 61.62, 51.73, 49.73, 28.35, 24.19; HRMS (ESI-TOF) m/z $[M+H]^+$ calcd for $C_{18}H_{25}N_4O_2$ 329.1972, found 329.1978.

Scheme 3: Synthesis of 15 (BY-011)

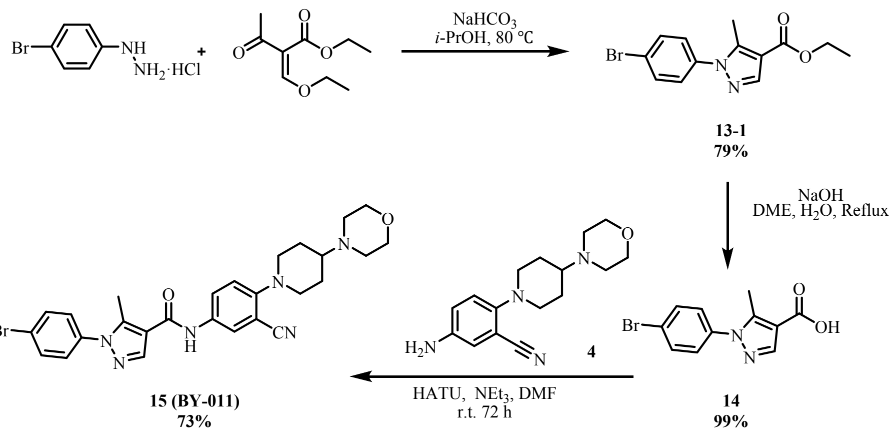

Compound 13:

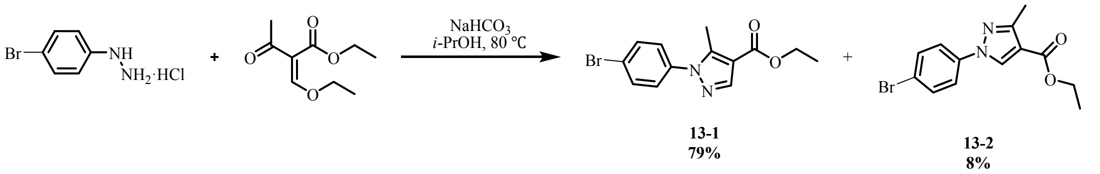

To a 25 mL flask 4-bromophenylhydrazine hydrochloride (1 g, 4.47 mmol, 1.0 equiv) and $NaHCO_3$ (375 mg, 4.47 mmol, 1.0 equiv) were added, followed by addition of 8 mL *i*-PrOH and 2-ethoxymethylene-3-oxobutanoic acid ethyl ester (954 μL, 5.36

mmol, 1.2 equiv). After stirring at 80 ℃ overnight, TLC indicated the reaction was complete. The reaction was diluted with ethyl acetate, then washed with brine. The organic layer was dried over anhydrous Na_2SO_4, filtered, and concentrated under vacuum. The residue was purified by silica gel column chromatography (petroleum ether/ethyl acetate = 25:1 to 20:1) to afford compound **13-1** (1.0994 g, 79%) as a yellowish solid, and compound **13-2** (113.7 mg, 8%) as a white solid. Compound **13-1**: ^{1}H NMR (500 MHz, $CDCl_3$) δ 8.00 (s, 1H), 7.60 (d, *J* = 8.6 Hz, 2H), 7.29 (d, *J* = 8.7 Hz, 3H), 4.29 (q, *J* = 7.2 Hz, 3H), 2.54 (s, 4H), 1.34 (t, *J* = 7.2 Hz, 3H); ^{13}C NMR (126 MHz, $CDCl_3$) δ 163.61, 143.48, 142.15, 137.84, 132.41, 126.90, 122.42, 113.30, 60.03, 14.42, 11.94; HRMS (ESI-TOF) m/z $[M+H]^+$ calcd for $C_{13}H_{14}BrN_2O_2$ 309.0233, found 309.0241. Compound **13-2**: ^{1}H NMR (400 MHz, $CDCl_3$) δ 8.31 (s, 1H), 7.59 – 7.54 (m, 4H), 4.32 (q, *J* = 7.1 Hz, 2H), 2.54 (s, 3H), 1.37 (t, *J* = 7.1 Hz, 3H); ^{13}C NMR (126 MHz, $CDCl_3$) δ 163.32, 152.62, 138.27, 132.54, 130.90, 120.59, 120.38, 114.68, 60.19, 14.40, 13.61; HRMS (ESI-TOF) m/z $[M+H]^+$ calcd for $C_{13}H_{14}BrN_2O_2$ 309.0233, found 309.02337.

Compound 14:

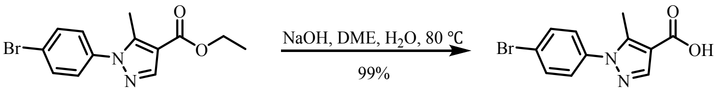

13-1 **14**

NaOH (207 mg, 5.18 mmol, 2.0 equiv) was dissolved in 13 mL H_2O, then added to a stirring solution of compound **13-1** (800 mg, 2.59 mmol, 1.0 equiv) in 13 mL DME. The resulting mixture was heated to 80 ℃ until TLC indicated the reaction was complete. The mixture was cool to room temperature, 5 mL petroleum ether was added and stirring continued vigorously for 2min. Organic phase was separated, water phase was acidified with 1N HCl until pH = 7, white precipitant formed. Extracted with ethyl acetate, then washed with brine. The organic layer was dried over anhydrous Na_2SO_4, filtered, and concentrated under vacuum to afford compound **14** (720.3 mg, 99%) as a white solid: ^{1}H NMR (500 MHz, DMSO-d6) δ 12.48 (s, 1H), 7.96 (s, 1H), 7.74 (d, *J* =

8.6 Hz, 1H), 7.50 (d, J = 8.4 Hz, 2H), 2.50 (s, 3H); ^{13}C NMR (126 MHz, DMSO-d6) δ 164.87, 143.90, 142.37, 138.30, 132.69, 127.71, 121.92, 113.69, 11.94; HRMS (ESI-TOF) m/z $[M+H]^+$ calcd for $C_{11}H_{10}BrN_2O_2$ 280.9920, found 280.9927.

Compound 15 (BY-011):

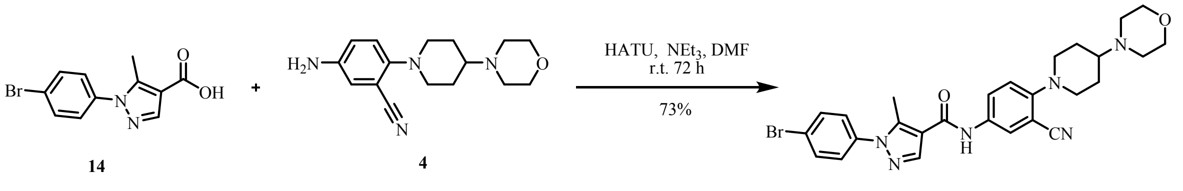

Compound **14** (15 mg, 54 μmol, 1.0 equiv), compound **4** (15 mg, 54 μmol, 1.0 equiv), and HATU (24 mg, 64.8 μmol, 1.2 equiv) were dissolved with 550 μL anhydrous DMF, in a 10 mL reaction seal tube, triethylamine (15 μL, 108 μmol, 2.0 equiv) was then added. The mixture was stirred at room temperature for 72 h. Then 1 mL methanol, 1 mL 10% K_2CO_3 water solution, 4 mL H_2O were added to the mixture. Pink precipitant was formed, then dispersed with ultrasonic. The precipitant was collected by centrifuging with 3000 rpm for 5 min, washed with 5 mL H_2O once to afford compound **15** (21.5 mg, 73%) as a white solid: ^{1}H NMR (600 MHz, DMSO-d6) δ 9.99 (s, 1H), 8.31 (s, 1H), 8.07 (d, J = 2.6 Hz, 1H), 7.85 (dd, J = 9.0, 2.6 Hz, 1H), 7.76 (d, J = 8.6 Hz, 2H), 7.54 (d, J = 8.6 Hz, 2H), 7.19 (d, J = 9.0 Hz, 1H), 3.59 (t, J = 4.6 Hz, 4H), 3.47 (d, J = 11.7 Hz, 2H), 2.77 (td, J = 12.0, 2.2 Hz, 2H), 2.56 (s, 3H), 2.51 (s, 4H), 2.30 (tt, J = 11.0, 3.8 Hz, 1H), 1.91 (d, J = 12.3 Hz, 2H), 1.58 (qd, J = 11.9, 3.8 Hz, 2H); ^{13}C NMR (151 MHz, DMSO-d6) δ 160.82, 150.85, 142.13, 138.93 (d, J = 6.4 Hz), 137.23, 132.76, 131.65, 126.64, 125.42, 124.14, 120.80, 119.07, 117.44, 114.95, 104.31, 65.98, 60.03, 50.71, 48.86, 27.54, 10.95; HRMS (ESI-TOF) m/z $[M+H]^+$ calcd for $C_{27}H_{30}BrN_6O_2$ 549.1608, found 549.1610.

Scheme 4: Synthesis of 18 (BY-014)

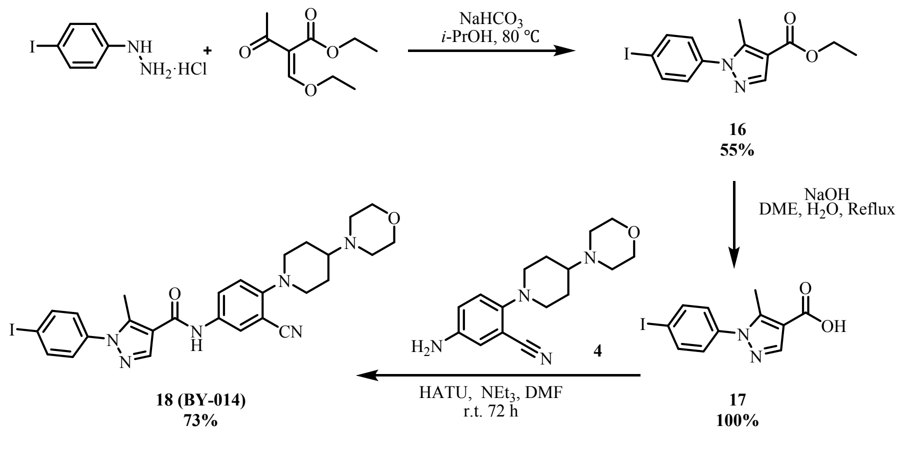

Compound 16:

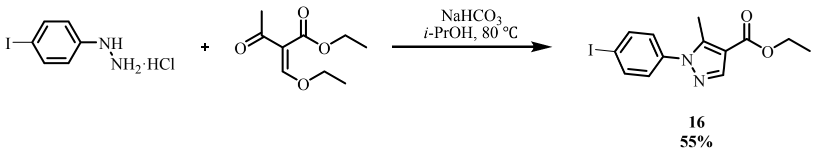

To a 25 mL flask 4-iodophenylhydrazine hydrochloride (500 mg, 1,85 mmol, 1.0 equiv) and $NaHCO_3$ (155 mg, 1.85 mmol, 1.0 equiv) were added, followed by addition of 5.1 mL *i*-PrOH and 2-ethoxymethylene-3-oxobutanoic acid ethyl ester (360 μL, 2.03 mmol, 1.1 equiv). After stirring at 80 ℃ overnight, TLC indicated the reaction was complete. The reaction was diluted with ethyl acetate, then washed with brine. The organic layer was dried over anhydrous Na_2SO_4, filtered, and concentrated under vacuum. The residue was purified by silica gel column chromatography (petroleum ether/ethyl acetate = 20:1) to afford compound **16** (360.8 mg, 55%) as a yellowish oil: ^{1}H NMR (500 MHz, $CDCl_3$) δ 8.01 (s, 1H), 7.81 (d, J = 8.7 Hz, 2H), 7.17 (d, J = 8.6 Hz, 2H), 4.31 (q, J = 7.1 Hz, 3H), 2.55 (s, 3H), 1.36 (t, J = 7.1 Hz, 4H); ^{13}C NMR (126 MHz, $CDCl_3$) δ 163.62, 143.45, 142.20, 138.55, 138.39, 127.06, 113.36, 93.83, 60.03, 14.42, 11.96; HRMS (ESI-TOF) m/z $[M+H]^+$ calcd for $C_{13}H_{14}IN_2O_2$ 357.0094, found 357.0102.

Compound 17:

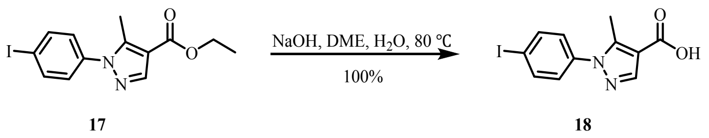

NaOH (44 mg, 1.12 mmol, 2.0 equiv) was dissolved in 3 mL H_2O, then added to a stirring solution of compound **17** (200 mg, 0.56 mmol, 1.0 equiv) in 3 mL DME. The resulting mixture was heated to 80 ℃ until TLC indicated the reaction was complete. The mixture was cool to room temperature, 5 mL petroleum ether was added and stirring continued vigorously for 2min. Organic phase was separated, water phase was acidified with 1N HCl until pH = 7, white precipitant formed. Extracted with ethyl acetate, then washed with brine. The organic layer was dried over anhydrous Na_2SO_4, filtered, and concentrated under vacuum to afford compound **18** (184.3 mg, 100%) as a white solid: ^{1}H NMR (500 MHz, DMSO-d6) δ 12.47 (s, 1H), 7.96 (s, 1H), 7.89 (dd, J = 8.7, 2.6 Hz, 2H), 7.34 (dd, J = 8.6, 2.0 Hz, 2H), 2.49 (s, 3H). 1H NMR (500 MHz, DMSO-d6) δ 12.47 (s, 1H), 7.96 (s, 1H), 7.89 (dd, J = 8.7, 2.6 Hz, 2H), 7.34 (dd, J = 8.6, 2.0 Hz, 2H), 2.49 (s, 3H). ^{13}C NMR (126 MHz, DMSO-d6) δ 164.86, 143.81, 142.37, 138.76, 138.53, 127.69, 113.72, 95.01, 11.96; HRMS (ESI-TOF) m/z $[M+H]^+$ calcd for $C_{11}H_{10}IN_2O_2$ 328.9781, found 328.9787

Compound 18 (BY-014):

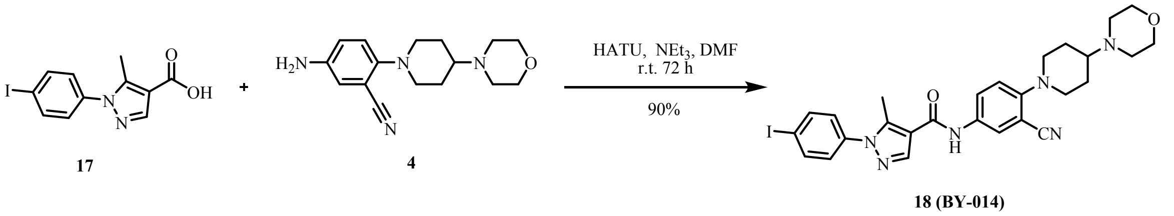

Compound **17** (20 mg, 60 μmol, 1.0 equiv), compound **4** (18 mg, 60 μmol, 1.0 equiv), and HATU (27 mg,72 μmol, 1.2 equiv) were dissolved with 600 μL anhydrous DMF, in a 10 mL reaction seal tube, triethylamine (10 μL, 120 μmol, 2.0 equiv) was then added. The mixture was stirred at room temperature for 72 h. Then 1 mL methanol, 1 mL 10% K_2CO_3 water solution, 4 mL H_2O were added to the mixture. Pink precipitant

was formed, then dispersed with ultrasonic. The precipitant was collected by centrifuging with 3000 rpm for 5 min, washed with 5 mL H_2O once to afford compound **18** (32.7 mg, 90%) as a white solid: ^{1}H NMR (600 MHz, DMSO-d6) δ 10.03 (s, 1H), 8.31 (s, 1H), 8.08 (d, *J* = 2.6 Hz, 1H), 7.93 (d, *J* = 8.6 Hz, 2H), 7.85 (dd, *J* = 8.9, 2.6 Hz, 1H), 7.38 (d, *J* = 8.8 Hz, 2H), 7.19 (d, *J* = 9.0 Hz, 1H), 3.59 (t, *J* = 4.6 Hz, 4H), 3.50 – 3.44 (m, 2H), 2.78 (td, *J* = 12.0, 2.3 Hz, 2H), 2.56 (s, 3H), 2.52 – 2.49 (m, 4H), 2.30 (tt, *J* = 11.1, 3.8 Hz, 1H), 1.95 – 1.88 (m, 2H), 1.58 (qd, *J* = 12.0, 3.8 Hz, 2H); ^{13}C NMR (151 MHz, DMSO-d6) δ 161.96, 151.86, 143.08 (d, *J* = 2.6 Hz), 140.08, 138.78, 138.56, 134.05, 127.69, 126.53, 125.25, 120.13, 118.54, 116.15, 105.40, 94.96, 67.06, 61.12, 51.81, 49.94, 28.62, 12.05; HRMS (ESI-TOF) m/z $[M+H]^+$ calcd for $C_{27}H_{30}IN_6O_2$ 597.1469, found 597.1470.

Scheme 5: Synthesis of 21 (BY-004)

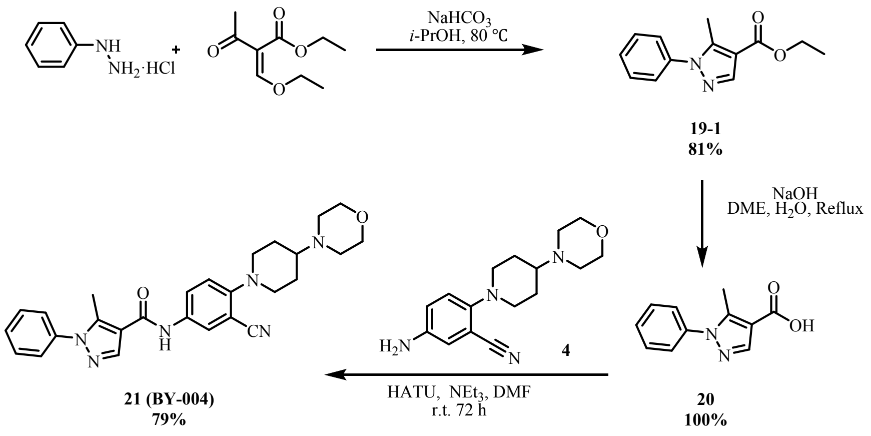

Compound 19:

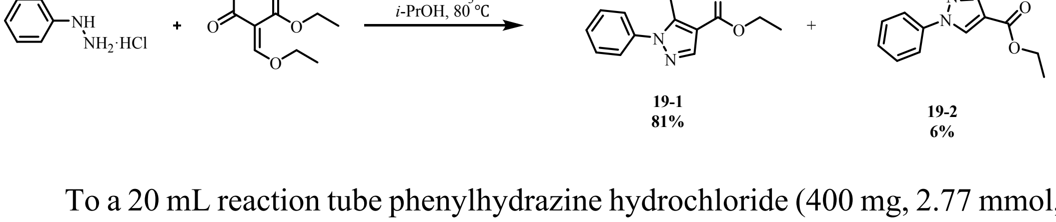

To a 20 mL reaction tube phenylhydrazine hydrochloride (400 mg, 2.77 mmol, 1.0 equiv) and $NaHCO_3$ (232.4 mg, 2.77 mmol, 1.0 equiv) were added, followed by

addition of 5.5 mL *i*-PrOH and 2-ethoxymethylene-3-oxobutanoic acid ethyl ester (540 μL, 3.05 mmol, 1.1 equiv). After stirring at 80 ℃ overnight, TLC indicated the reaction was complete. The reaction was diluted with ethyl acetate, then washed with brine. The organic layer was dried over anhydrous Na_2SO_4, filtered, and concentrated under vacuum. The residue was purified by silica gel column chromatography (petroleum ether/ethyl acetate = 20:1) to afford compound **19-1** (514.4 mg, 81%) as a yellowish oil, and compound **19-2** (38 mg, 6%) as a yellowish oil. Compound **19-1**: ^{1}H NMR (500 MHz, DMSO-d6) δ 8.01 (s, 1H), 7.61 – 7.47 (m, 5H), 4.25 (q, J = 7.1 Hz, 2H), 2.51 (s, 3H), 1.29 (t, J = 7.1 Hz, 3H); ^{13}C NMR (126 MHz, DMSO-d6) δ 163.36, 143.81, 141.68, 138.91, 129.77, 129.11, 125.81, 112.66, 60.03, 14.75, 12.05; HRMS (ESI-TOF) m/z $[M+H]^+$ calcd for $C_{13}H_{15}N_2O_2$ 231.1128, found 231.1135. Compound **19-2**: ^{1}H NMR (500 MHz, DMSO-d6) δ 8.94 (s, 0H), 7.93 – 7.84 (m, 1H), 7.51 – 7.44 (m, 1H), 7.36 – 7.29 (m, 0H), 4.23 (q, J = 7.1 Hz, 1H), 2.42 (s, 1H), 1.29 (t, J = 7.1 Hz, 1H); ^{13}C NMR (126 MHz, DMSO-d6) δ 163.19, 151.55, 139.23, 132.39, 129.98, 127.38, 119.15, 114.00, 60.15, 14.75, 13.85; HRMS (ESI-TOF) m/z $[M+H]^+$ calcd for $C_{13}H_{15}N_2O_2$ 231.1128, found 231.1134.

Compound 20:

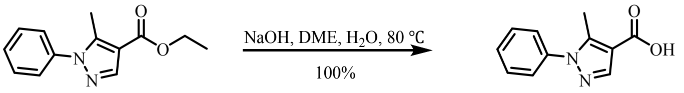

19-1 **20**

NaOH (153 mg, 3.83 mmol, 2.2 equiv) was dissolved in 9 mL H_2O, then added to a stirring solution of compound **19-1** (410 mg, 1.74 mmol, 1.0 equiv) in 6 mL DME. The resulting mixture was heated to 80 ℃ until TLC indicated the reaction was complete. The mixture was cool to room temperature, 5 mL petroleum ether was added and stirring continued vigorously for 2 min. Organic phase was separated, water phase was acidified with 1N HCl until pH = 7, white precipitant formed. Extracted with ethyl acetate, then washed with brine. The organic layer was dried over anhydrous Na_2SO_4, filtered, and concentrated under vacuum to afford compound **20** (358.9 mg, 100%) as

a white solid: ^{1}H NMR (500 MHz, DMSO-d6) δ 12.44 (s, 1H), 7.95 (s, 1H), 7.60 – 7.44 (m, 5H), 2.48 (s, 3H); ^{13}C NMR (126 MHz, DMSO-d6) δ 164.97, 143.67, 142.06, 139.04, 129.74, 128.99, 125.77, 113.40, 11.99; HRMS (ESI-TOF) m/z $[M+H]^+$ calcd for $C_{11}H_{11}N_2O_2$ 203.0815, found 203.0821.

Compound 21 (BY-004):

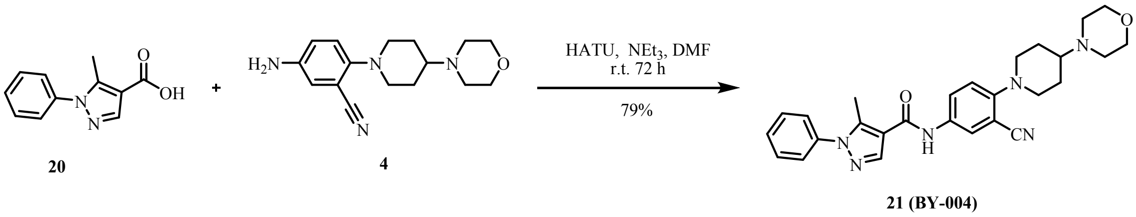

Compound **20** (13 mg, 64.25 μmol, 1.0 equiv), compound **4** (18 mg, 64.25 μmol, 1.0 equiv), and HATU (29 mg, 77.1 μmol, 1.2 equiv) were dissolved with 650 μL anhydrous DMF, in a 10 mL reaction seal tube, triethylamine (18 μL, 128.5 μmol, 2.0 equiv) was then added. The mixture was stirred at room temperature for 72 h. Then 1 mL methanol, 1 mL 10% K_2CO_3 water solution, 4 mL H_2O were added to the mixture. Pink precipitant was formed, then dispersed with ultrasonic. The precipitant was collected by centrifuging with 3000 rpm for 5 min, washed with 5 mL H_2O once to afford compound **21** (23.8 mg, 79%) as a white solid: ^{1}H NMR (600 MHz, DMSO-d6) δ 9.98 (s, 1H), 8.29 (s, 1H), 8.07 (d, *J* = 2.6 Hz, 1H), 7.85 (dd, *J* = 9.0, 2.6 Hz, 1H), 7.60 – 7.53 (m, 4H), 7.53 – 7.48 (m, 1H), 7.19 (d, *J* = 9.0 Hz, 1H), 3.61 – 3.57 (m, 5H), 3.47 (d, *J* = 11.6 Hz, 2H), 2.77 (td, *J* = 12.0, 2.2 Hz, 2H), 2.54 (s, 3H), 2.51 (s, 4H), 2.30 (tt, *J* = 11.2, 3.8 Hz, 1H), 1.91 (d, *J* = 12.2 Hz, 2H), 1.58 (qd, *J* = 11.9, 3.8 Hz, 2H); ^{13}C NMR (151 MHz, DMSO-d6) δ 160.96, 150.82, 141.91, 138.63, 137.98, 132.84, 128.70, 127.90, 125.41, 124.71, 124.13, 119.08, 117.46, 114.69, 104.34, 65.97, 60.05, 50.73, 48.86, 27.54, 11.01; HRMS (ESI-TOF) m/z $[M+H]^+$ calcd for $C_{27}H_{31}N_6O_2$ 471.2503, found 471.2507.

Scheme 6: Synthesis of 24 (BY-001)

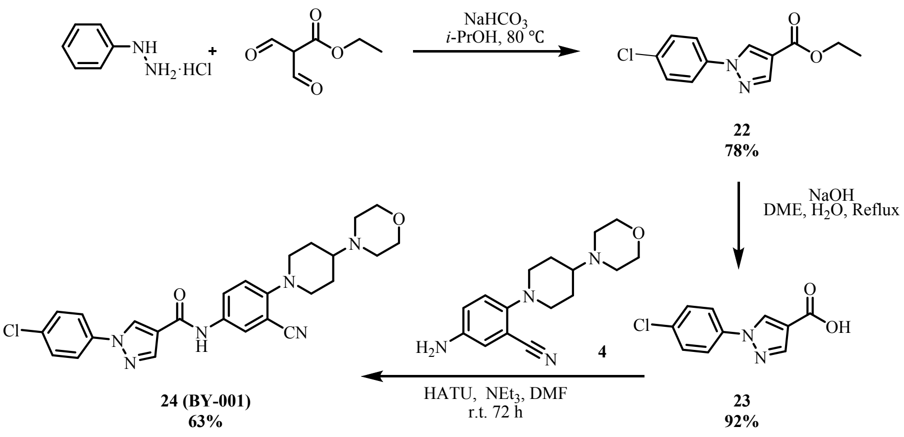

Compound 22:

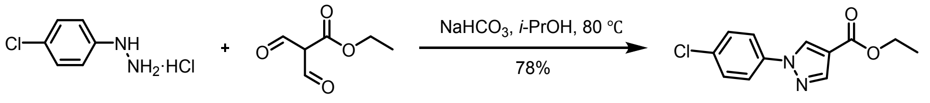

To a 20 mL reaction tube, 4-Chlorophenylhydrazine hydrochloride (400 mg, 2.23 mmol, 1.0 equiv) and $NaHCO_3$ (188 mg, 2.23 mmol, 1.0 equiv) were added, followed by addition of 6 mL *i*-PrOH and ethyl 2-formyl-3-oxopropanoate (281 μL, 2.23 mmol, 1.0 equiv). After stirring at 80 °C overnight, TLC indicated the reaction was complete. The reaction was diluted with ethyl acetate, then washed with brine. The organic layer was dried over anhydrous Na_2SO_4, filtered, and concentrated under vacuum. The residue was purified by silica gel column chromatography (petroleum ether/ethyl acetate = 20:1) to afford compound **22** (438.6 mg, 78%) as a yellowish solid: ^{1}H NMR (500 MHz, DMSO-d6) δ 9.10 (s, 1H), 8.12 (s, 1H), 7.94 (d, J = 8.2 Hz, 2H), 7.55 (d, J = 7.8 Hz, 2H), 4.25 (q, J = 7.2 Hz, 2H), 1.28 (t, J = 7.2 Hz, 3H); ^{13}C NMR (126 MHz, DMSO-d6) δ 162.43, 142.43, 138.18, 131.98, 131.81 (d, J = 1.8 Hz), 129.96 (d, J = 1.6 Hz), 121.17, 116.91, 60.48, 14.73; HRMS (ESI-TOF) m/z $[M+H]^+$ calcd for $C_{12}H_{12}ClN_2O_2$ 251.0582, found 251.0588.

Compound 23:

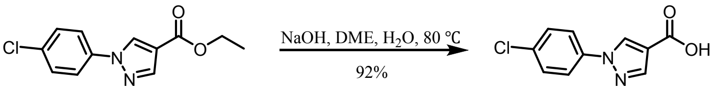

22 23

NaOH (64 mg, 1.6 mmol, 2.0 equiv) was dissolved in 4 mL water, then added to a stirring solution of compound **22** (200 mg, 800 μmol, 1.0 equiv) in 4 mL DME. The resulting mixture was heated to 80 ℃ until TLC indicated the reaction was complete. The mixture was cool to room temperature, 4 mL petroleum was added, stirring continued vigorously for 2 min. Organic phase was separated, water phase was acidified with 1N HCl until pH = 7, white precipitant formed. The water phase was extracted with ethyl acetate, then washed with brine. The organic layer was dried over anhydrous Na_2SO_4, filtered, and concentrated under vacuum to afford compound **23** (162.8 mg, 92%) as a white solid: ^{1}H NMR (500 MHz, DMSO-d6) δ 12.72 (br, 1H), 9.03 (s, 1H), 8.08 (s, 1H), 7.94 (d, *J* = 8.9 Hz, 2H), 7.57 (d, *J* = 8.9 Hz, 2H); ^{13}C NMR (126 MHz, DMSO-d6) δ 164.00, 142.74, 138.31, 131.73, 129.96, 121.10, 118.12; HRMS (ESI-TOF) m/z $[M+H]^+$ calcd for $C_{10}H_8ClN_2O_2$ 223.0269, found 223.0275.

Compound 24 (BY-001):

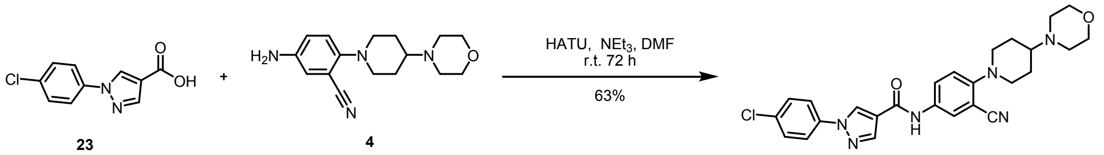

23 4

24 (BY-001)

Compound **23** (13 mg, 58.39 μmol, 1.0 equiv), compound **4** (16.7 mg, 58.39 μmol, 1.0 equiv), and HATU (26.6 mg, 70 μmol, 1.2 equiv) were dissolved with 600 μL anhydrous DMF, in a 10 mL reaction seal tube, triethylamine (12 μL, 116.7 μmol, 2.0 equiv) was then added. The mixture was stirred at room temperature for 72 h. Then 1 mL methanol, 1 mL 10% K_2CO_3 water solution, 4 mL H_2O were added to the mixture. Pink precipitant was formed, then dispersed with ultrasonic. The precipitant was collected by centrifuging with 3000 rpm for 5 min, washed with 5 mL H_2O once to

afford compound **24** (18.2 mg, 63%) as a white solid: ^{1}H NMR (600 MHz, DMSO-d6) δ 10.13 (s, 1H), 9.10 (s, 1H), 8.32 (s, 1H), 8.06 (d, *J* = 2.6 Hz, 1H), 7.95 – 7.91 (m, 2H), 7.84 (dd, *J* = 9.0, 2.6 Hz, 1H), 7.65 – 7.57 (m, 2H), 7.21 (d, *J* = 9.0 Hz, 1H), 3.61 – 3.57 (m, 4H), 3.48 (d, *J* = 11.7 Hz, 2H), 2.78 (td, *J* = 12.1, 2.3 Hz, 2H), 2.51 (s, 4H), 2.35 – 2.28 (m, 1H), 1.94 – 1.89 (m, 2H), 1.58 (qd, *J* = 11.9, 3.8 Hz, 2H); ^{13}C NMR (151 MHz, DMSO-d6) δ 159.34, 150.97, 140.15, 137.29, 132.53, 130.77, 129.37, 129.05, 125.31, 124.03, 119.97, 119.91, 119.20, 117.43, 104.35, 65.94, 60.05, 50.67, 48.85, 27.52; HRMS (ESI-TOF) m/z $[M+H]^+$ calcd for $C_{26}H_{28}ClN_6O_2$ 491.1957, found 491.1949.

Scheme 7: Synthesis of 27 (BY-018)

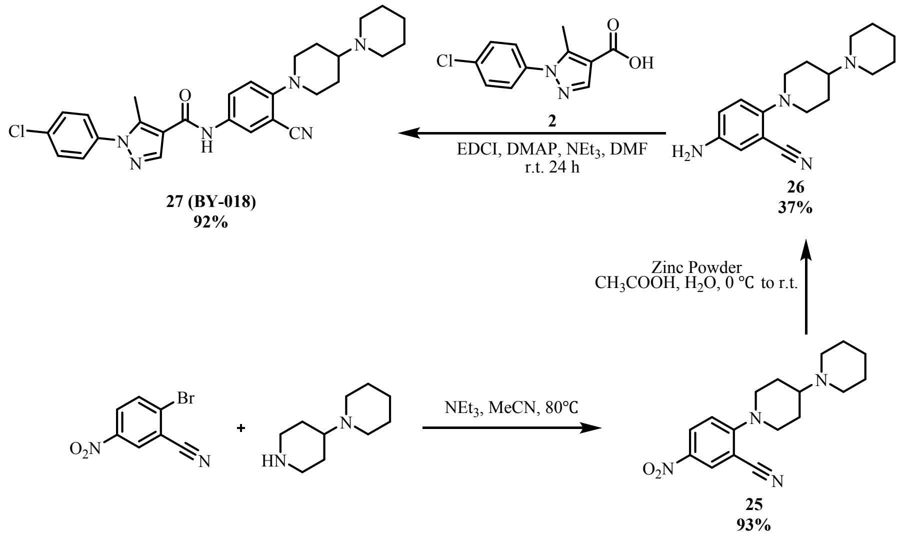

Compound 25:

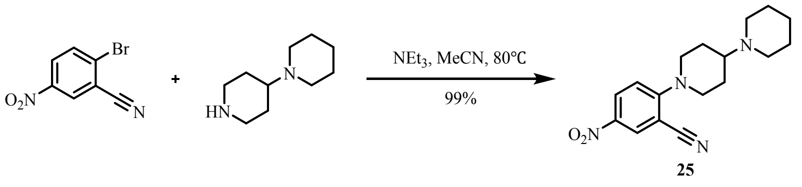

2-bromo-5-nitrobenzonitrile (500 mg, 2.2 mmol, 1.0 equiv) and 4-piperidinopiperidin (444.7 mg,2.64 mmol, 1.2 equiv) were dissolved with 4 mL MeCN. Triethylamine (918 μL, 6.6 mmol, 3 equiv) was added, the mixture was stirred at 80 °C

for about 4 h until TLC indicated the reaction was complete. The mixture was diluted with DCM, washed with brine. The organic layer was dried over anhydrous Na_2SO_4, filtered, and concentrated under vacuum. The residue was purified by silica gel column chromatography (ethyl acetate/methanol = 1:0 to 20:1) to afford compound **25** (645.9 mg, 93%) as a light yellow solid: ^{1}H NMR (500 MHz, $CDCl_3$) δ 8.39 (d, *J* = 4.7 Hz, 1H), 8.21 (dd, *J* = 9.3, 2.8 Hz, 1H), 6.95 (d, *J* = 9.5 Hz, 1H), 4.01 (dt, *J* = 13.1, 2.4 Hz, 2H), 3.08 (td, *J* = 12.5, 2.5 Hz, 2H), 2.56 – 2.47 (m, 5H), 1.98 (d, *J* = 10.7 Hz, 2H), 1.76 (qd, *J* = 12.2, 3.7 Hz, 2H), 1.63 – 1.55 (m, 4H), 1.44 (qd, *J* = 6.6, 4.4, 3.8 Hz, 2H); ^{13}C NMR (126 MHz, $CDCl_3$) δ 158.27, 138.97, 131.52, 128.84, 117.45, 117.15, 101.28, 61.71, 50.68, 50.24, 27.99, 26.34, 24.69; HRMS (ESI-TOF) m/z $[M+H]^+$ calcd for $C_{17}H_{23}N_4O_2$ 315.1816, found 315.1818.

Compound 26:

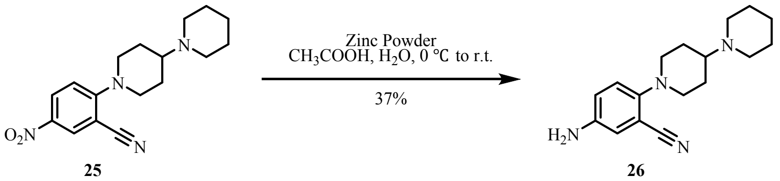

Compound **25** (300 mg, 954 μmol, 1.0 equiv) was dissolved with 5 mL acetic acid and 5 mL H_2O. The mixture was cooled with ice-bath, zinc powder (623 mg, 9.54 mmol, 10.0 equiv) was added portion-wise. After adding, the mixture was stirring at 0 ℃ for 5 min, then warm to room temperature slowly, yellow solution faded slowly as reaction went. The mixture was stirring at room temperature for about 30 min until TLC indicated the reaction was complete. The volatile compounds were removed under vacuum Remain crude was diluted with ethyl acetate, then washed with 2N KOH solution, then washed with brine. The organic layer was dried over anhydrous Na_2SO_4, filtered, and concentrated under vacuum. The residue was purified by silica gel column chromatography (DCM/methanol = 15:1) to afford compound **26** (101.1 mg, 37%) as a light-yellow solid: ^{1}H NMR (500 MHz, $CDCl_3$) δ 6.87 (d, *J* = 8.7 Hz, 1H), 6.85 (d, *J* = 2.9 Hz, 1H), 6.79 (dd, *J* = 8.6, 2.8 Hz, 1H), 3.62 (s, 2H), 3.41 (dq, *J* = 11.4, 2.2 Hz,

2H), 2.67 (td, J = 11.8, 2.5 Hz, 2H), 2.59 – 2.53 (m, 4H), 2.41 (tt, J = 11.5, 3.9 Hz, 1H), 1.92 – 1.86 (m, 2H), 1.81 (qd, J = 11.9, 3.8 Hz, 2H), 1.61 (p, J = 5.6 Hz, 4H), 1.44 (p, J = 6.3 Hz, 2H); ^{13}C NMR (126 MHz, $CDCl_3$) δ 148.71, 141.31, 120.55, 120.37, 119.32, 118.26, 107.93, 62.43, 52.91, 50.10, 28.08, 26.28, 24.73; HRMS (ESI-TOF) m/z $[M+H]^+$ calcd for $C_{17}H_{25}N_4$ 285.2074, found 285.2079.

Compound 27 (BY-018):

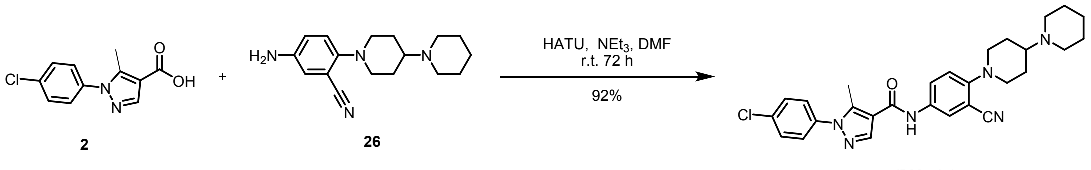

Compound **2** (15 mg, 63.38 μmol, 1.0 equiv), compound **26** (18 mg, 63.38 μmol, 1.0 equiv), and HATU (29 mg, 76.05 μmol, 1.2 equiv) were dissolved with 600 μL anhydrous DMF, in a 10 mL reaction seal tube, triethylamine (18 μL, 126.76 μmol, 2.0 equiv) was then added. The mixture was stirred at room temperature for 72 h. Then 1 mL methanol, 1 mL 10% K_2CO_3 water solution, 4 mL H_2O were added to the mixture. Pink precipitant was formed, then dispersed with ultrasonic. The precipitant was collected by centrifuging with 3000 rpm for 5 min, washed with 5 mL H_2O once to afford compound **27** (30.3 mg, 92%) as a white solid: ^{1}H NMR (600 MHz, DMSO-d6) δ 10.05 (s, 1H), 8.32 (s, 1H), 8.07 (d, J = 2.6 Hz, 1H), 7.85 (dd, J = 9.0, 2.6 Hz, 1H), 7.64 (d, J = 8.9 Hz, 2H), 7.60 (d, J = 8.8 Hz, 2H), 7.18 (d, J = 9.0 Hz, 1H), 3.47 (d, J = 11.8 Hz, 2H), 2.75 (td, J = 12.1, 2.3 Hz, 2H), 2.55 (s, 3H), 2.50 – 2.46 (m, 4H), 2.40 – 2.32 (m, 1H), 1.83 (d, J = 12.0 Hz, 2H), 1.62 (qd, J = 12.2, 3.9 Hz, 2H), 1.53 – 1.47 (m, 4H), 1.42 – 1.35 (m, 2H); ^{13}C NMR (151 MHz, DMSO-d6) δ 161.93, 151.98, 143.21, 140.03, 137.91, 133.85, 133.41, 129.79, 127.47, 126.52, 125.23, 120.12, 118.55, 116.02, 105.34, 61.74, 52.25, 50.21, 28.31, 26.59, 25.07, 12.03. ^{13}C NMR (151 MHz, DMSO-d6) δ 161.93, 151.98, 143.21, 140.03, 137.91, 133.85, 133.41, 129.79, 127.47, 126.52, 125.23, 120.12, 118.55, 116.02, 105.34, 61.74, 52.25, 50.21, 28.31, 26.59, 25.07, 12.03. ^{13}C NMR (151 MHz, DMSO-d6) δ 161.93, 151.98, 143.21, 140.03,

137.91, 133.85, 133.41, 129.79, 127.47, 126.52, 125.23, 120.12, 118.55, 116.02, 105.34, 61.74, 52.25, 50.21, 28.31, 26.59, 25.07, 12.03; HRMS (ESI-TOF) m/z $[M+H]^+$ calcd for $C_{28}H_{32}NClN_6O$ 503.2321, found 503.2314.

Scheme 8: Synthesis of 30 (BY-007)

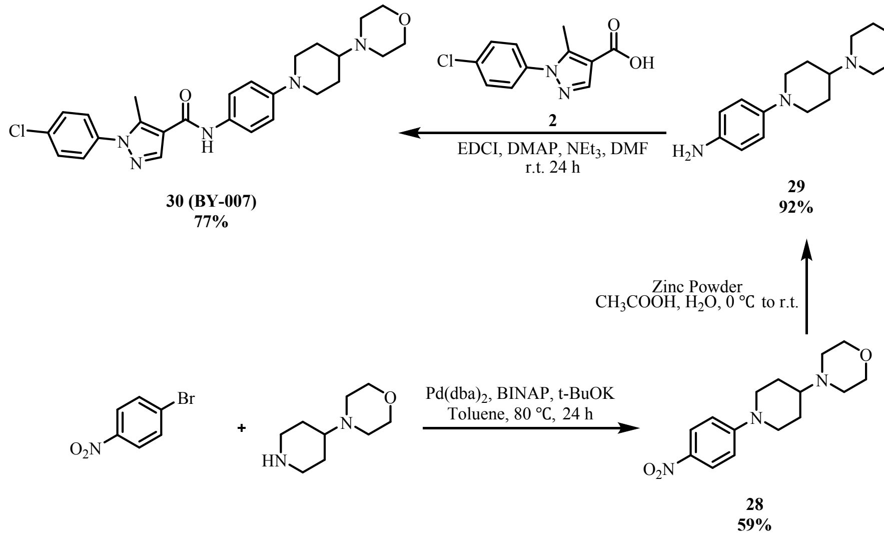

Compound 28:

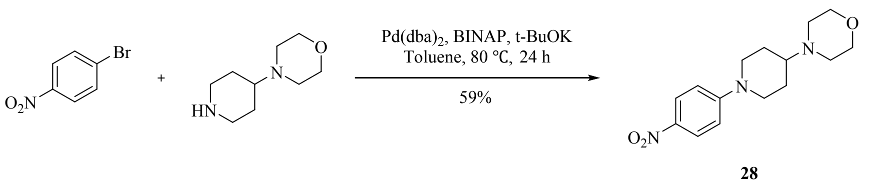

In a 50 mL flask, 4-bromonitrobenzene (500 mg, 2.48 mmol, 1.0 equiv) 4-(piperidin-4-yl) morpholine (505 mg, 2.97 mmol, 1.2 equiv), *t*-BuOK (289 mg, 3.47 mmol, 1.4 equiv) were added in, then recharged with argon three times. 15 mL toluene was added by syringe. After the mixture was bubbled with argon for 15min, $Pd(dba)_2$ (42 mg, 74.4 μmol, 3mol%) and Binap (96 mg, 124 μmol, 5mol%) were added under argon atmosphere. The mixture was stirred at 80 °C for about 24 h. Until TLC indicated the reaction was complete, the resulting mixture was cooled to room temperature and filtered through a pad of celite and washed with ethyl acetate three times. Combine organic phase, then washed with brine. The organic layer was dried over anhydrous

Na_2SO_4, filtered, and concentrated under vacuum. The residue was purified by silica gel column chromatography (ethyl acetate/methanol = 1:0 to 20:1) to afford compound **28** (427.2 mg, 59%) as a yellow solid: ^1H NMR (500 MHz, DMSO-d6) δ 8.01 (d, *J* = 9.7 Hz, 2H), 6.99 (d, *J* = 9.7 Hz, 2H), 4.05 – 3.98 (m, 2H), 3.53 (t, *J* = 4.6 Hz, 4H), 3.02 – 2.91 (m, 2H), 2.46 – 2.37 (m, 5H), 1.87 – 1.80 (m, 2H), 1.38 (qd, *J* = 12.1, 4.0 Hz, 2H); ^{13}C NMR (101 MHz, DMSO-d6) δ 154.81, 136.72, 126.36, 112.95, 66.98, 61.12, 49.86, 46.31, 27.76; HRMS (ESI-TOF) m/z $[M+H]^+$ calcd for $C_{15}H_{22}N_3O_3$ 292.1656, found 292.1658.

Compound 29:

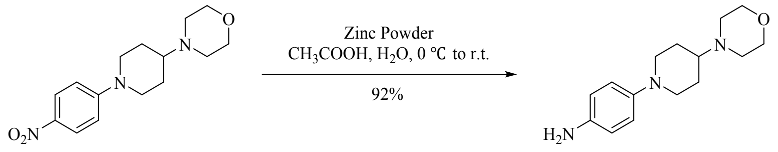

28 29

Compound **28** (250 mg, 857 μmol, 1.0 equiv), was dissolved with 4 mL acetic acid and 8 mL 1N HCl. The mixture was cooled with ice-bath, zinc powder (561 mg, 8.57 mmol, 10.0 equiv) was added portion-wise. After adding, the mixture was stirring at 0 ℃ for 5 min, then warm to room temperature slowly, yellow solution faded slowly as reaction went. The mixture was stirring at room temperature for about 30 min until TLC indicated the reaction was complete. The volatile compounds were removed under vacuum Remain crude was diluted with ethyl acetate, then washed with 2N KOH solution, then washed with brine. The organic layer was dried over anhydrous Na_2SO_4, filtered, and concentrated under vacuum to afford compound **29** (205.7 mg, 92%) as yellowish solid, used directly in next step without further purification. Compound **29**: ^1H NMR (500 MHz, DMSO-d6) δ 6.66 (d, *J* = 8.8 Hz, 2H), 6.45 (d, *J* = 8.7 Hz, 2H), 4.53 (br, 2H), 3.57 – 3.52 (m, 4H), 3.38 – 3.32 (m, 2H), 2.47 – 2.39 (m, 6H), 2.14 (tt, *J* = 11.2, 3.7 Hz, 1H), 1.84 – 1.77 (m, 2H), 1.45 (qd, *J* = 12.0, 3.9 Hz, 2H); ^{13}C NMR (126 MHz, DMSO-d6) δ 143.10, 142.52, 118.96, 115.13, 67.01, 61.65, 50.94, 49.95, 28.53; HRMS (ESI-TOF) m/z $[M+H]^+$ calcd for $C_{15}H_{24}N_3O$ 262.1914, found 262.1914.

Compound 30 (BY-007):

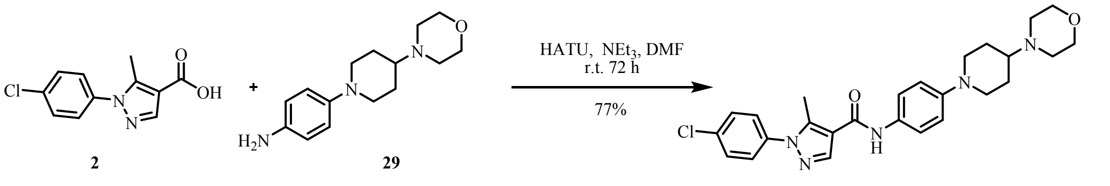

30 (BY-007)

Compound 2 (15 mg, 63.38 μmol, 1.0 equiv), compound 33 (17 mg, 63.38 μmol, 1.0 equiv), and HATU (29 mg, 76 μmol, 1.2 equiv) were dissolved with 600 μL anhydrous DMF, in a 10 mL reaction seal tube, triethylamine (13 μL, 126.76 μmol, 2.0 equiv) was then added. The mixture was stirred at room temperature for 72 h. Then 1 mL methanol, 1 mL 10% K_2CO_3 water solution, 4 mL H_2O were added to the mixture. Pink precipitant was formed, then dispersed with ultrasonic. The precipitant was collected by centrifuging with 3000 rpm for 5 min, washed with 5 mL H_2O once to afford compound 35 (23.4 mg, 77%) as white solid: ^{1}H NMR (500 MHz, DMSO-d6) δ 9.67 (s, 1H), 8.28 (s, 1H), 7.61 (d, *J* = 8.8 Hz, 2H), 7.57 (d, *J* = 8.8 Hz, 2H), 7.51 (d, *J* = 8.9 Hz, 2H), 6.90 (d, *J* = 8.9 Hz, 2H), 3.64 (d, *J* = 12.1 Hz, 2H), 3.55 (t, *J* = 4.6 Hz, 4H), 2.60 (dd, *J* = 12.9, 10.5 Hz, 2H), 2.53 (s, 3H), 2.48 – 2.43 (m, 4H), 2.28 – 2.18 (m, 1H), 1.84 (d, *J* = 12.4 Hz, 2H), 1.47 (qd, *J* = 11.8, 3.9 Hz, 2H); ^{13}C NMR (151 MHz, DMSO-d6) δ 160.38, 146.75, 141.57, 138.87, 136.94, 132.18, 130.13, 128.67, 126.32, 120.78, 115.50, 115.34, 65.94, 60.46, 48.87, 47.91, 27.03, 10.91; HRMS (ESI-TOF) m/z $[M+H]^+$ calcd for $C_{26}H_{31}ClN_5O_2$ 480.2161, found 480.2161.

Scheme 9: Synthesis of 33 (BY-003)

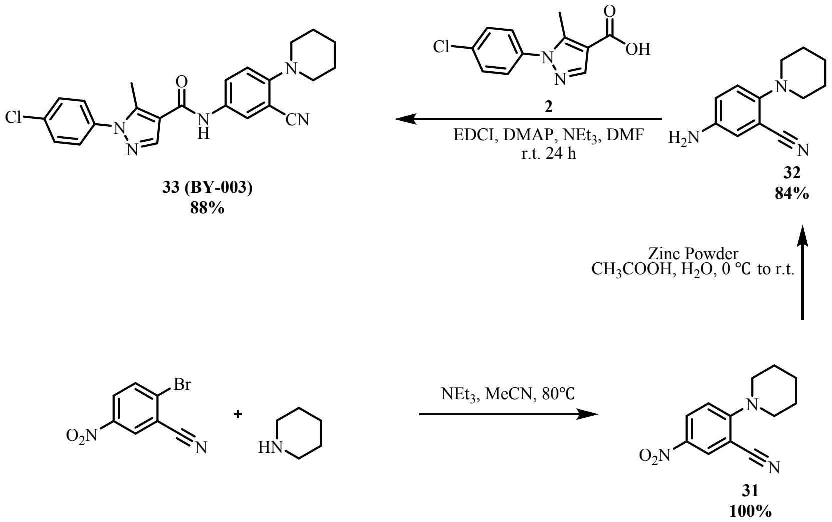

Compound 31:

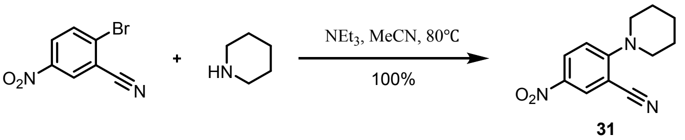

2-bromo-5-nitrobenzonitrile (400 mg, 1.76 mmol, 1.0 equiv) and piperidin (208 μL, 1.584 mmol, 1.2 equiv), were dissolved with 3.5 mL MeCN. Triethylamine (735 μL, 4.752 mmol, 3 equiv) was then added, the mixture was stirred at 80 °C for about 4 h until TLC indicated the reaction was complete. The mixture was diluted with DCM, washed with brine. The organic layer was dried over anhydrous Na_2SO_4, filtered, and concentrated under vacuum. The residue was purified by silica gel column chromatography (petroleum ether/ethyl acetate = 5:1) to afford compound **31** (406.9 mg, 100%) as a light-yellow solid: ^{1}H NMR (500 MHz, DMSO-d6) δ 8.46 (d, *J* = 2.9 Hz, 1H), 8.22 (dd, *J* = 9.5, 2.9 Hz, 1H), 7.19 (d, *J* = 9.4 Hz, 1H), 3.50 – 3.44 (m, 4H), 1.69 – 1.57 (m, 6H); ^{13}C NMR (126 MHz, DMSO-d6) δ 158.69, 138.44, 131.93, 129.40, 118.65, 117.73, 100.08, 51.71, 25.89, 23.69; HRMS (ESI-TOF) m/z $[M+H]^+$ calcd for $C_{12}H_{14}N_3O_2$ 232.1081, found 232.1086.

Compound 32:

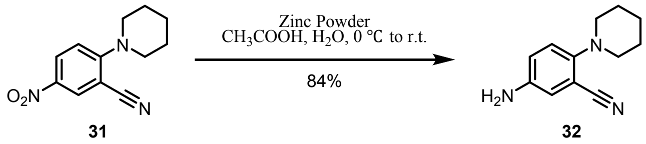

Compound **31** (150 mg, 648 μmol, 1.0 equiv), was dissolved with 3 mL acetic acid and 4 mL H_2O. The mixture was cooled with ice-bath, zinc powder (424 mg, 6.48 mmol, 10.0 equiv) was added portion-wise. After adding, the mixture was stirring at 0 ℃ for 5 min, then warm to room temperature slowly, yellow solution faded slowly as reaction went. The mixture was stirring at room temperature for about 30 min until TLC indicated the reaction was complete. The volatile compounds were removed under vacuum Remain crude was diluted with ethyl acetate, then washed with 2N KOH solution, then washed with brine. The organic layer was dried over anhydrous Na_2SO_4, filtered, and concentrated under vacuum to afford compound **32** (109.7 mg, 84%) as white solid, used directly in next step without further purification. Compound **32**: ^{1}H NMR (500 MHz, DMSO-d6) δ 6.92 (dd, J = 6.3, 2.9 Hz, 1H), 6.81 – 6.75 (m, 2H), 5.14 (s, 2H), 2.85 – 2.80 (m, 4H), 1.65 – 1.57 (m, 4H), 1.50 – 1.42 (m, 2H); ^{13}C NMR (126 MHz, DMSO-d6) δ 147.04, 144.77, 121.14, 120.04, 118.88, 117.64, 107.50, 54.14, 26.42, 23.97; HRMS (ESI-TOF) m/z $[M+H]^+$ calcd for $C_{12}H_{16}N_3$ 202.1339, found 202.1347.

Compound 33 (BY-003):

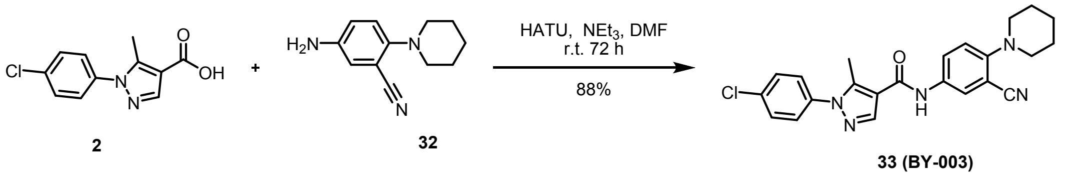

Compound **2** (15 mg, 63.38 μmol, 1.0 equiv), compound **32** (12.76 mg, 63.38 μmol, 1.0 equiv), and HATU (29 mg, 76 μmol, 1.2 equiv) were dissolved with 650 μL anhydrous DMF, in a 10 mL reaction seal tube, triethylamine (18 μL, 126.7 μmol, 2.0 equiv) was then added. The mixture was stirred at room temperature for 72 h. Then 1

mL methanol, 1 mL 10% K_2CO_3 water solution, 4 mL H_2O were added to the mixture. Pink precipitant was formed, then dispersed with ultrasonic. The precipitant was collected by centrifuging with 3000 rpm for 5 min, washed with 5 mL H_2O once to afford compound **33** (23.2 mg, 88%) as white solid: ^{1}H NMR (600 MHz, DMSO-d6) δ 9.98 (s, 1H), 8.30 (s, 1H), 8.06 (d, *J* = 2.6 Hz, 1H), 7.84 (dd, *J* = 9.0, 2.6 Hz, 1H), 7.65 – 7.62 (m, 2H), 7.62 – 7.58 (m, 2H), 7.18 (d, *J* = 9.0 Hz, 1H), 3.06 (t, *J* = 5.3 Hz, 4H), 2.55 (s, 3H), 1.68 (p, *J* = 5.7 Hz, 4H), 1.55 (tt, *J* = 9.0, 4.4 Hz, 2H); ^{13}C NMR (151 MHz, DMSO-d6) δ 160.83, 151.59, 142.15, 138.92, 136.83, 132.67, 132.35, 128.72, 126.40, 125.46, 124.17, 119.07, 117.47, 114.93, 104.42, 52.29, 25.14, 22.86, 10.95; HRMS (ESI-TOF) m/z $[M+H]^+$ calcd for $C_{23}H_{23}ClN_5O$ 420.1586, found 420.1591.

Scheme 10: Synthesis of 36 (BY-010)

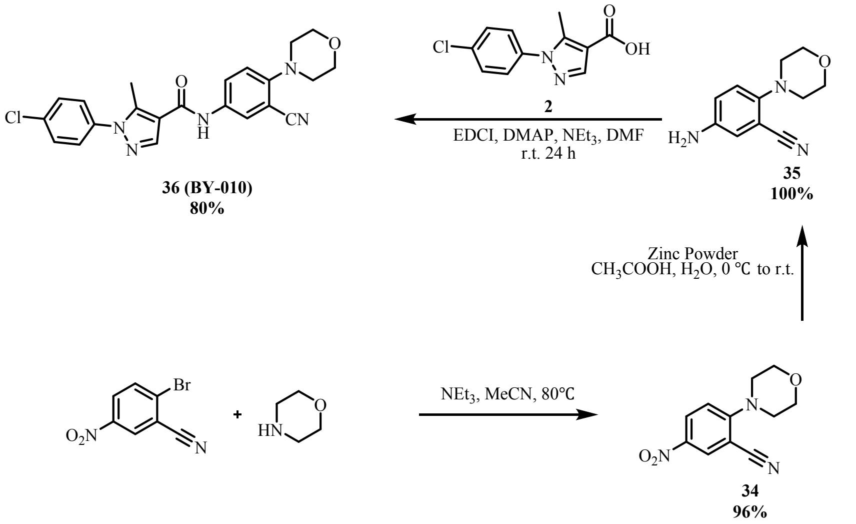

Compound 34:

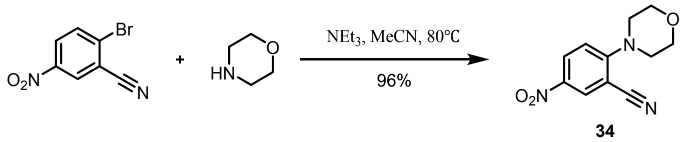

2-Bromo-5-nitrobenzonitrile (500 mg, 2.20 mmol, 1.0 equiv) and morpholine (231 μL, 2.64 mmol, 1.2 equiv), were dissolved with 4 mL MeCN. Triethylamine (918 μL, 6.6 mmol, 3 equiv) was then added, the mixture was stirred at 80 °C for about 4 h until TLC indicated the reaction was complete. The mixture was diluted with DCM, washed

with brine. The organic layer was dried over anhydrous Na_2SO_4, filtered, and concentrated under vacuum. The residue was purified by silica gel column chromatography (petroleum ether/ethyl acetate = 5:1 to 3:1) to afford compound **34** (493.3 mg, 96%) as light-yellow solid: 1H NMR (500 MHz, $CDCl_3$) δ 8.43 (d, *J* = 2.7 Hz, 1H), 8.29 (dd, *J* = 9.3, 2.7 Hz, 1H), 7.00 (d, *J* = 9.3 Hz, 1H), 3.93 – 3.87 (m, 4H), 3.53 – 3.46 (m, 4H); ^{13}C NMR (126 MHz, $CDCl_3$) δ 158.48, 140.10, 131.22, 129.09, 117.64, 116.80, 102.64, 66.45, 50.78; HRMS (ESI-TOF) m/z $[M+Na]^+$ calcd for $C_{11}H_{11}N_3NaO_3$ 256.0693, found 256.0698.

Compound 35:

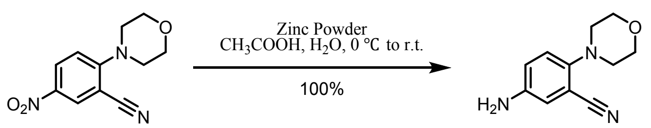

34
35

Compound **34** (200 mg, 857 μmol, 1.0 equiv), was dissolved with 4 mL acetic acid and 4 mL H_2O. The mixture was cooled with ice-bath, zinc powder (560 mg, 8.57 mmol, 10.0 equiv) was added portion-wise. After adding, the mixture was stirring at 0 ℃ for 5 min, then warm to room temperature slowly, yellow solution faded slowly as reaction went. The mixture was stirring at room temperature for about 30 min until TLC indicated the reaction was complete. The volatile compounds were removed under vacuum Remain crude was diluted with ethyl acetate, then washed with 2N KOH solution, then washed with brine. The organic layer was dried over anhydrous Na_2SO_4, filtered, and concentrated under vacuum to afford compound **35** (174 mg, 100%) as yellowish solid, used directly in next step without further purification. Compound **35**: ^1H NMR (500 MHz, $CDCl_3$) δ 6.91 – 6.88 (m, 2H), 6.84 (dd, *J* = 8.6, 2.8 Hz, 1H), 3.89 – 3.85 (m, 4H), 3.66 (br, 2H), 3.05 – 3.01 (m, 4H); ^{13}C NMR (126 MHz, $CDCl_3$) δ 147.80, 141.93, 120.58, 120.34, 119.40, 118.05, 108.16, 67.12, 52.63; HRMS (ESI-TOF) m/z $[M+H]^+$ calcd for $C_{11}H_{14}N_3O$ 204.1131, found 204.1136.

Compound 36 (BY-010):

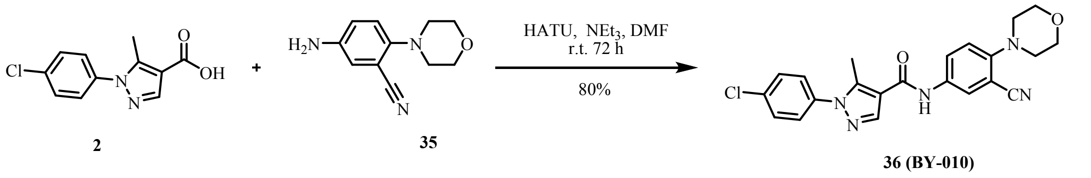

Compound **2** (15 mg, 63.38 μmol, 1.0 equiv), compound **35** (13 mg, 63.38 μmol, 1.0 equiv), and HATU (29 mg, 76 μmol, 1.2 equiv) were dissolved with 630 μL anhydrous DMF, in a 10 mL reaction seal tube, triethylamine (18 μL, 126.7 μmol, 2.0 equiv) was then added. The mixture was stirred at room temperature for 72 h. Then 1 mL methanol, 1 mL 10% K_2CO_3 water solution, 4 mL H_2O were added to the mixture. Pink precipitant was formed, then dispersed with ultrasonic. The precipitant was collected by centrifuging with 3000 rpm for 5 min, washed with 5 mL H_2O once to afford compound **36** (22.4 mg, 80%) as white solid: ^{1}H NMR (600 MHz, DMSO-d6) δ 10.04 (s, 1H), 8.32 (s, 1H), 8.11 (d, *J* = 2.6 Hz, 1H), 7.89 (dd, *J* = 8.9, 2.6 Hz, 1H), 7.64 (d, *J* = 8.8 Hz, 2H), 7.60 (d, *J* = 8.8 Hz, 2H), 7.22 (d, *J* = 9.0 Hz, 1H), 3.80 – 3.75 (m, 4H), 3.12 – 3.08 (m, 4H), 2.56 (s, 3H); ^{13}C NMR (151 MHz, DMSO-d6) δ 160.87, 150.18, 142.19, 138.92, 136.79, 133.29, 132.34, 128.70, 126.38, 125.49, 124.22, 119.06, 117.32, 114.86, 104.34, 65.58, 51.12, 10.94; HRMS (ESI-TOF) m/z $[M+H]^+$ calcd for $C_{22}H_{21}ClN_5O_2$ 422.1378, found 422.1377.

Scheme 11: Synthesis of 39 (BY-019)

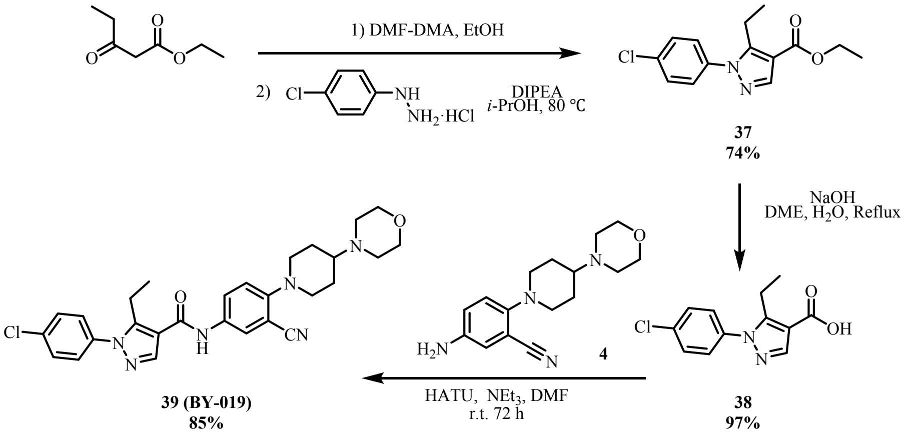

Compound 37:

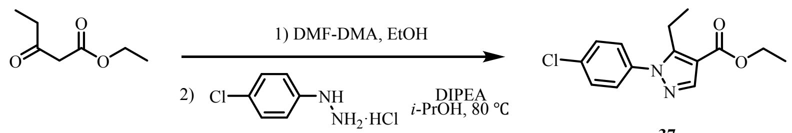

37
74%

To a 20 mL reaction tube, ethyl propanoylacetate (500 μL, 3.51 mM, 1.0 equiv） and DMF-DMA (513 μL, 3.86 mM, 1.1 equiv) were dissolved with 1.75 mL EtOH. The mixture was stirred at 80 °C for about 4 h, then concentrated under vacuum. Followed by addition of 4-chlorophenylhydrazine hydrochloride (550 mg, 3.07 mM, 0.9 equiv) and N,N-Diisopropylethylamine (588 μL, 3.51 mM, 1.0 equiv) in 3.1 mL EtOH. The mixture was stirred at 80 °C overnight. TLC indicated the reaction was complete. The reaction was diluted with ethyl acetate, then washed with brine. The organic layer was dried over anhydrous Na_2SO_4, filtered, and concentrated under vacuum. The residue was purified by silica gel column chromatography (petroleum ether/ethyl acetate = 10:1) to afford compound **37** (549.3 mg, 64%) as a yellowish oil: ^{1}H NMR (400 MHz, $CDCl_3$) δ 8.02 (s, 1H), 7.49 (dd, J = 23.4, 8.8 Hz, 2H), 7.36 (d, J = 8.8 Hz, 2H), 4.33 (q, J = 7.1 Hz, 2H), 2.95 (q, J = 7.5 Hz, 2H), 1.38 (t, J = 7.1 Hz, 3H), 1.18 (t, J = 7.5 Hz, 3H); ^{13}C NMR (101 MHz, $CDCl_3$) δ 163.43, 149.51, 142.33, 137.48, 134.87, 129.55, 127.13, 112.34, 60.05, 18.64, 14.42, 13.67; HRMS (ESI-TOF) m/z $[M+H]^+$ calcd for $C_{14}H_{16}ClN_2O_2$ 279.0895, found 279.0898.

Compound 38:

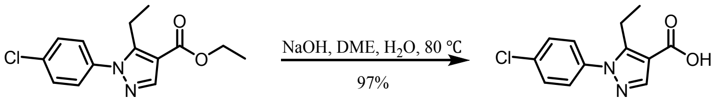

37 38

NaOH (58 mg, 1.43 mmol, 2.0 equiv) was dissolved in 3.5 mL water, then added to a stirring solution of compound **37** (200 mg, 715 μmol, 1.0 equiv) in 3.5 mL DME. The resulting mixture was heated to 80 °C until TLC indicated the reaction was complete. The mixture was cool to room temperature, 4 mL petroleum was added, stirring continued vigorously for 2 min. Organic phase was separated, water phase was

acidified with 1N HCl until pH = 7, white precipitant formed. The water phase was extracted with ethyl acetate, then washed with brine. The organic layer was dried over anhydrous Na_2SO_4, filtered, and concentrated under vacuum to afford compound **38** (173.9 mg, 97%) as a white solid: ^1H NMR (500 MHz, $CDCl_3$) δ 8.09 (s, 1H), 7.50 (d, *J* = 8.7 Hz, 2H), 7.37 (dd, *J* = 8.4, 3.2 Hz, 2H), 2.96 (q, *J* = 7.5 Hz, 2H), 1.19 (t, *J* = 7.4 Hz, 4H); ^{13}C NMR (126 MHz, $CDCl_3$) δ 150.53, 143.01, 137.23, 135.09, 129.59, 127.16, 111.49, 18.65, 13.65; HRMS (ESI-TOF) m/z $[M+H]^+$ calcd for $C_{12}H_{12}ClN_2O_2$ 251.0582, found 251.0590.

Compound 39 (BY-019):

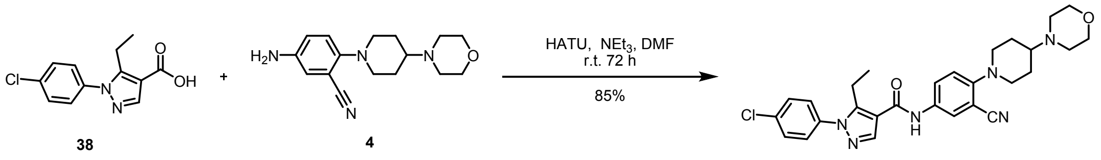

Compound **38** (15 mg, 60 μmol, 1.0 equiv), compound **4** (17 mg, 60 μmol, 1.0 equiv), and HATU (27 mg, 72 μmol, 1.2 equiv) were dissolved with 600 μL anhydrous DMF, in a 10 mL reaction seal tube, triethylamine (17 μL, 120 μmol, 2.0 equiv) was then added. The mixture was stirred at room temperature for 72 h. Then 1 mL methanol, 1 mL 10% K_2CO_3 water solution, 4 mL H_2O were added to the mixture. Pink precipitant was formed, then dispersed with ultrasonic. The precipitant was collected by centrifuging with 3000 rpm for 5 min, washed with 5 mL H_2O once to afford compound **39** (26.5 mg, 85%) as white solid: ^1H NMR (600 MHz, DMSO-d6) δ 10.06 (s, 1H), 8.32 (s, 1H), 8.09 (d, *J* = 2.6 Hz, 1H), 7.86 (dd, *J* = 9.0, 2.6 Hz, 1H), 7.66 (d, *J* = 8.9 Hz, 2H), 7.55 (d, *J* = 9.0 Hz, 2H), 7.19 (d, *J* = 9.0 Hz, 1H), 3.59 (t, *J* = 4.6 Hz, 5H), 3.51 – 3.44 (m, 2H), 2.96 (q, *J* = 7.4 Hz, 2H), 2.78 (td, *J* = 12.0, 2.2 Hz, 2H), 2.52 – 2.48 (m, 4H), 2.30 (tt, *J* = 11.1, 3.8 Hz, 1H), 1.94 – 1.89 (m, 2H), 1.58 (qd, *J* = 12.0, 3.8 Hz, 2H); ^{13}C NMR (151 MHz, DMSO-d6) δ 161.72, 151.92, 148.81, 140.03, 137.99, 133.90, 133.89, 129.95, 128.07, 126.53, 125.24, 120.12, 118.53, 115.11, 105.39, 67.05, 61.12,

51.80, 49.94, 28.61, 18.37, 13.79; HRMS (ESI-TOF) m/z $[M+H]^+$ calcd for $C_{28}H_{32}ClN_6O_2$ 519.2270, found 519.2275.

Scheme 12: Synthesis of 42 (BY-024)

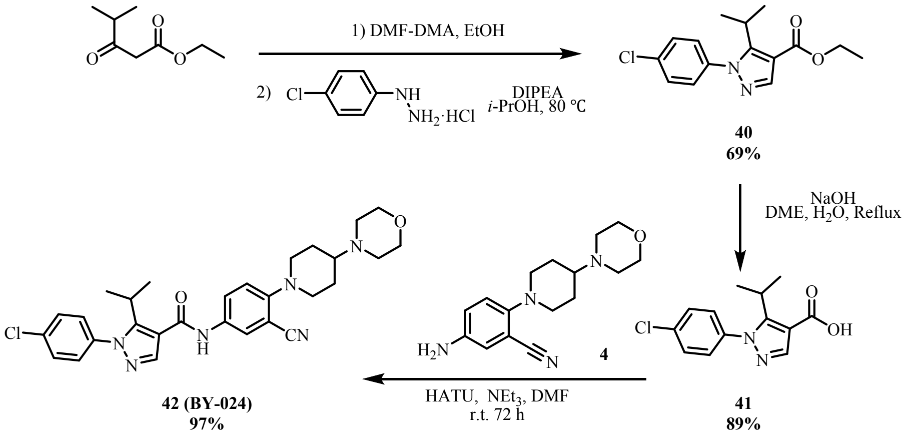

Compound 40:

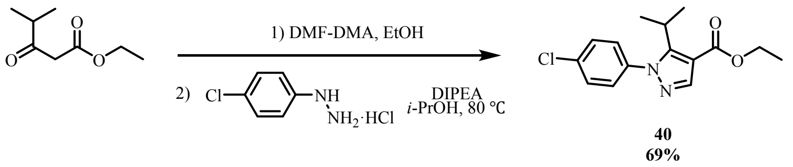

To a 20 mL reaction tube, ethyl 4-methyl-3-oxo-pentanoate (500 μL, 3.1 mM, 1.0 equiv） and DMF-DMA (452 μL, 3.41 mM, 1.1 equiv) were dissolved with 1.6 mL EtOH. The mixture was stirred at 80 ℃ for about 4 h, then concentrated under vacuum. Followed by addition of 4-chlorophenylhydrazine hydrochloride (530 mg, 2.96 mM, 0.9 equiv) and N,N-Diisopropylethylamine (567 μL, 2.96 mM, 1.0 equiv) in 3 mL EtOH. The mixture was stirred at 80 ℃ overnight. TLC indicated the reaction was complete. The reaction was diluted with ethyl acetate, then washed with brine. The organic layer was dried over anhydrous Na_2SO_4, filtered, and concentrated under vacuum. The residue was purified by silica gel column chromatography (petroleum ether/ethyl acetate = 10:1) to afford compound **40** (541.3 mg, 69%) as a yellowish oil: ^1H NMR (400 MHz, $CDCl_3$) δ 8.02 (s, 1H), 7.48 (d, J = 8.5 Hz, 2H), 7.31 (d, J = 8.5 Hz, 2H), 4.32 (q, J = 7.1 Hz, 2H), 3.24 (hept, J = 7.2 Hz, 1H), 1.38 (dd, J = 14.9, 7.1 Hz, 9H); ^{13}C NMR (101 MHz, $CDCl_3$) δ 163.25, 152.92, 143.28, 137.97, 135.15,

129.47, 127.91, 111.88, 60.12, 26.43, 20.11, 14.43; HRMS (ESI-TOF) m/z $[M+H]^+$ calcd for $C_{15}H_{18}ClN_2O_2$ 293.1051, found 293.1055.

Compound 41:

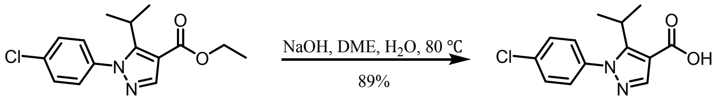

40 41

NaOH (58 mg, 1.43 mmol, 2.0 equiv) was dissolved in 3.5 mL water, then added to a stirring solution of compound **40** (200 mg, 715 μmol, 1.0 equiv) in 3.5 mL DME. The resulting mixture was heated to 80 ℃ until TLC indicated the reaction was complete. The mixture was cool to room temperature, 4 mL petroleum was added, stirring continued vigorously for 2 min. Organic phase was separated, water phase was acidified with 1N HCl until pH = 7, white precipitant formed. The water phase was extracted with ethyl acetate, then washed with brine. The organic layer was dried over anhydrous Na_2SO_4, filtered, and concentrated under vacuum to afford compound **41** (161.4 mg, 90%) as a white solid: ^1H NMR (500 MHz, $CDCl_3$) δ 8.11 (d, J = 1.3 Hz, 1H), 7.49 (d, J = 8.8 Hz, 2H), 7.32 (d, J = 8.7 Hz, 2H), 3.26 (hept, J = 7.0 Hz, 1H), 1.37 (s, 4H), 1.36 (s, 3H); ^{13}C NMR (126 MHz, $CDCl_3$) δ 153.92, 144.02, 137.73, 135.35, 129.51, 127.90, 26.48, 20.01; HRMS (ESI-TOF) m/z $[M+H]^+$ calcd for $C_{13}H_{14}ClN_2O_2$ 265.0738, found265.0744.

Compound 42 (BY-024):

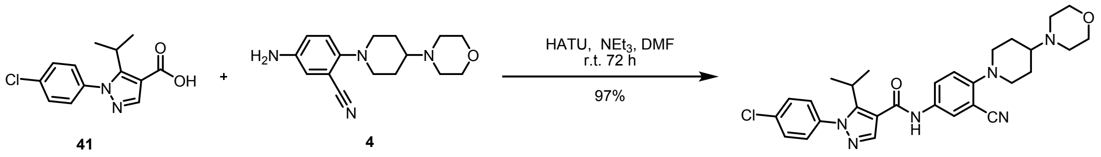

41 **4** **42 (BY-024)**

Compound **41** (15 mg, 56 μmol, 1.0 equiv), compound **4** (16 mg, 56 μmol, 1.0 equiv), and HATU (26 mg, 67.2 μmol, 1.2 equiv) were dissolved with 600 μL anhydrous DMF, in a 10 mL reaction seal tube, triethylamine (16 μL, 112 μmol, 2.0

equiv) was then added. The mixture was stirred at room temperature for 72 h. Then 1 mL methanol, 1 mL 10% K_2CO_3 water solution, 4 mL H_2O were added to the mixture. Pink precipitant was formed, then dispersed with ultrasonic. The precipitant was collected by centrifuging with 3000 rpm for 5 min, washed with 5 mL H_2O once to afford compound **42** (29.3 mg, 97%) as white solid: ^{1}H NMR (600 MHz, DMSO-d6) δ 10.11 (s, 1H), 8.21 (s, 1H), 8.07 (d, *J* = 2.6 Hz, 1H), 7.86 (dd, *J* = 8.9, 2.6 Hz, 1H), 7.66 (d, *J* = 8.8 Hz, 2H), 7.49 (d, *J* = 8.7 Hz, 2H), 7.19 (d, *J* = 9.0 Hz, 1H), 3.59 (t, *J* = 4.6 Hz, 5H), 3.50 – 3.45 (m, 2H), 3.19 – 3.12 (m, 1H), 2.78 (td, *J* = 12.0, 2.3 Hz, 2H), 2.52 – 2.48 (m, 4H), 2.34 – 2.26 (m, 1H), 1.95 – 1.88 (m, 2H), 1.58 (qd, *J* = 12.0, 3.8 Hz, 2H), 1.30 (d, *J* = 7.0 Hz, 7H); ^{13}C NMR (151 MHz, DMSO-d6) δ 162.04, 151.93, 151.51, 140.74, 138.45, 134.26, 134.02, 129.89, 128.95, 126.50, 125.15, 120.13, 118.54, 115.51, 105.41, 67.05, 61.12, 51.82, 49.93, 28.60, 26.47, 20.72; HRMS (ESI-TOF) m/z $[M+H]^+$ calcd for $C_{29}H_{34}ClN_6O_2$ 533.2426, found 533.2434.

^1H and ^{13}C NMR Spectra of Compounds

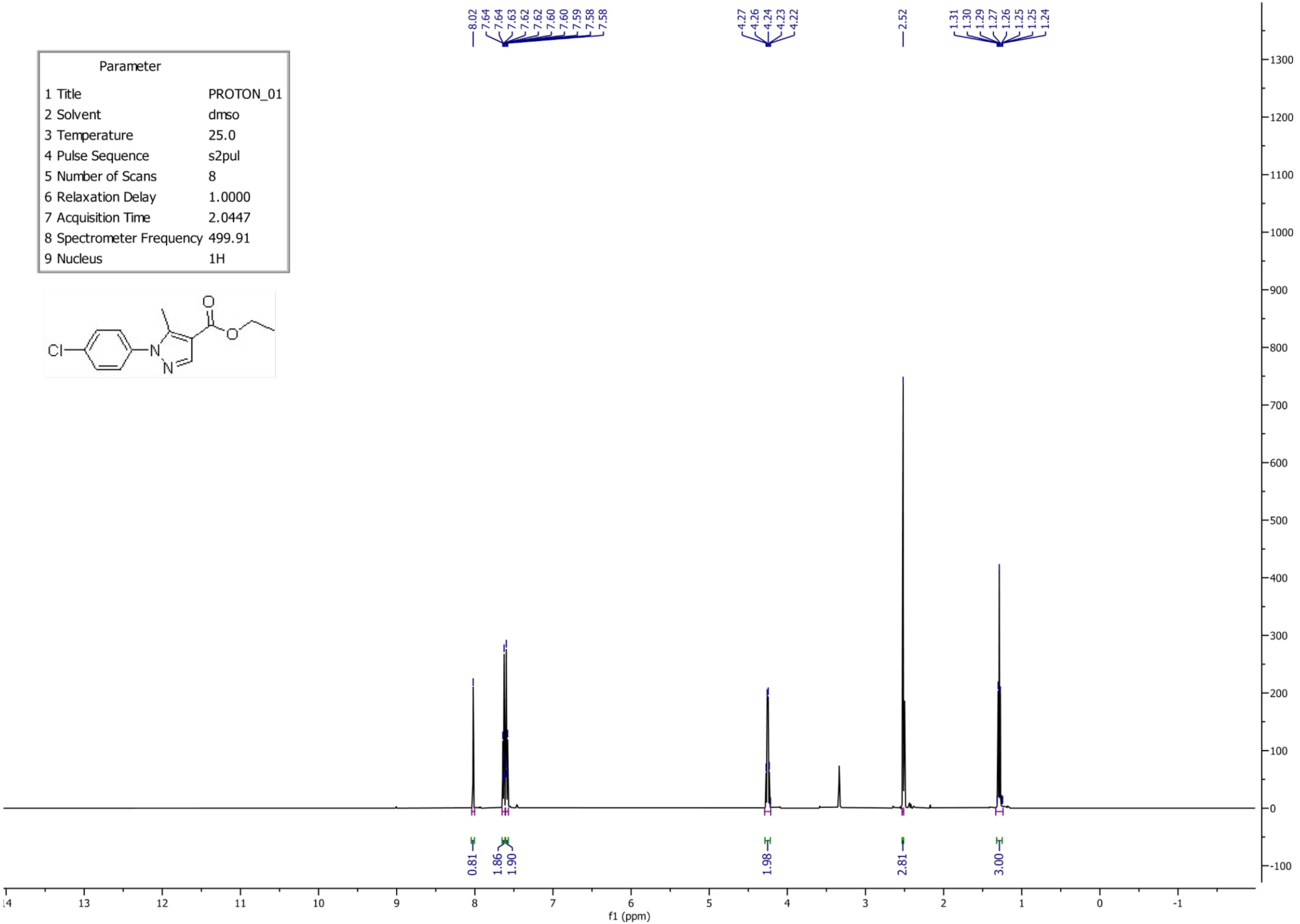

Figure S1 ^{1}H NMR spectrum of compound **1-1 (BY-022)** (DMSO-d6, 500 MHz)

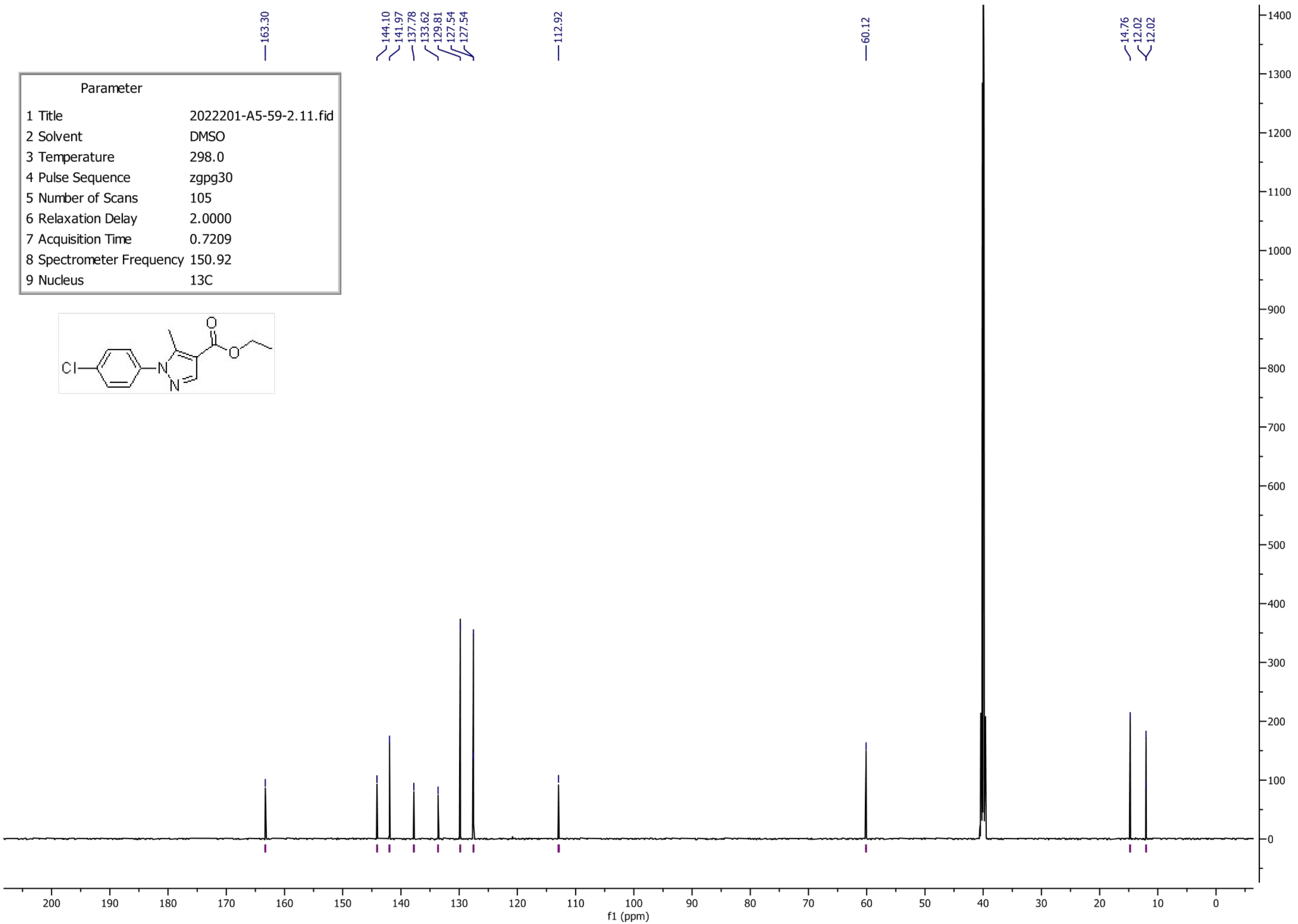

Figure S2 ^{13}C NMR spectrum of compound **1-1 (BY-022)** (DMSO-d6, 151 MHz)

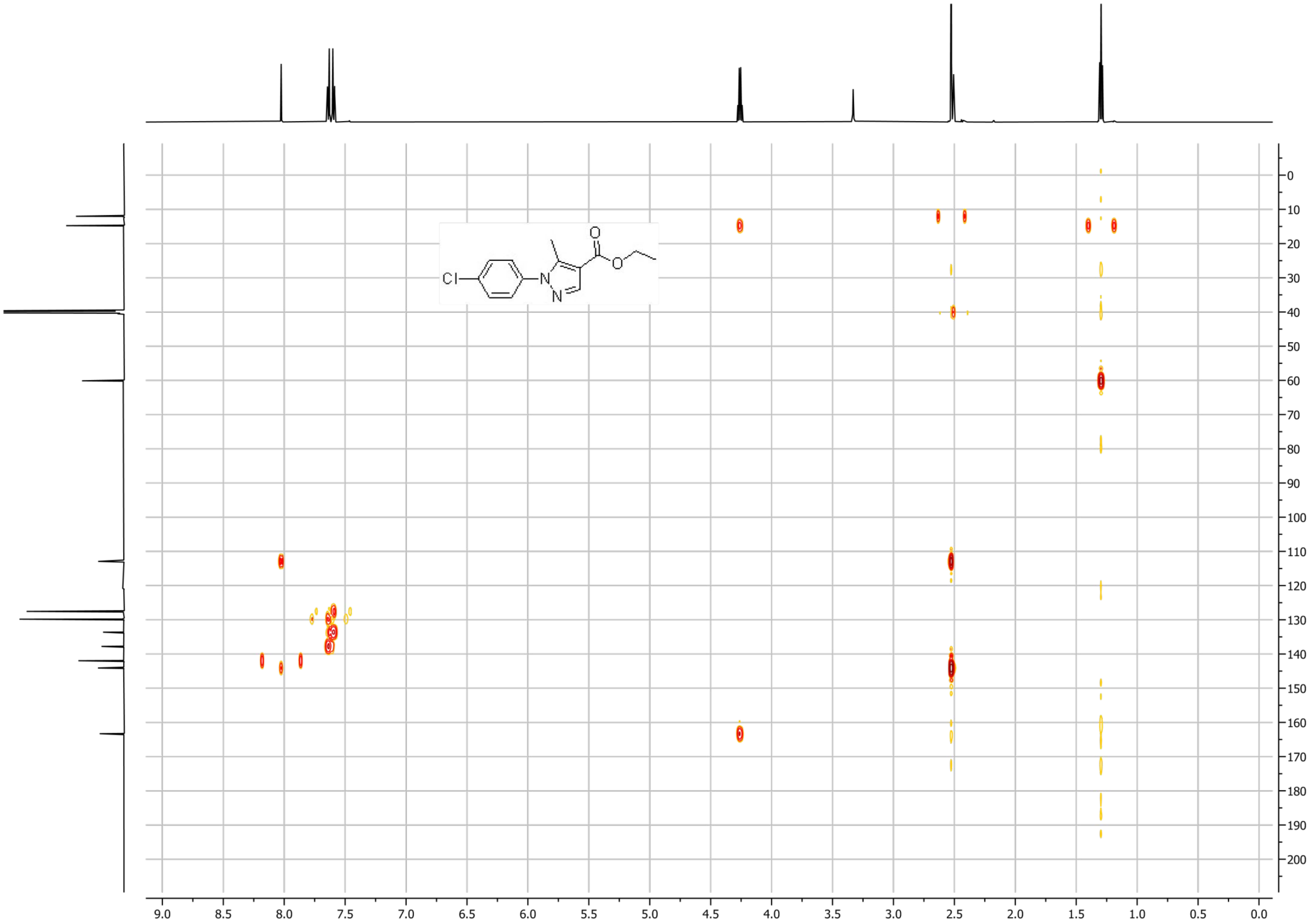

Figure S3 HMBC spectrum of compound **1-1 (BY-022)** (DMSO-d6, 600 MHz)

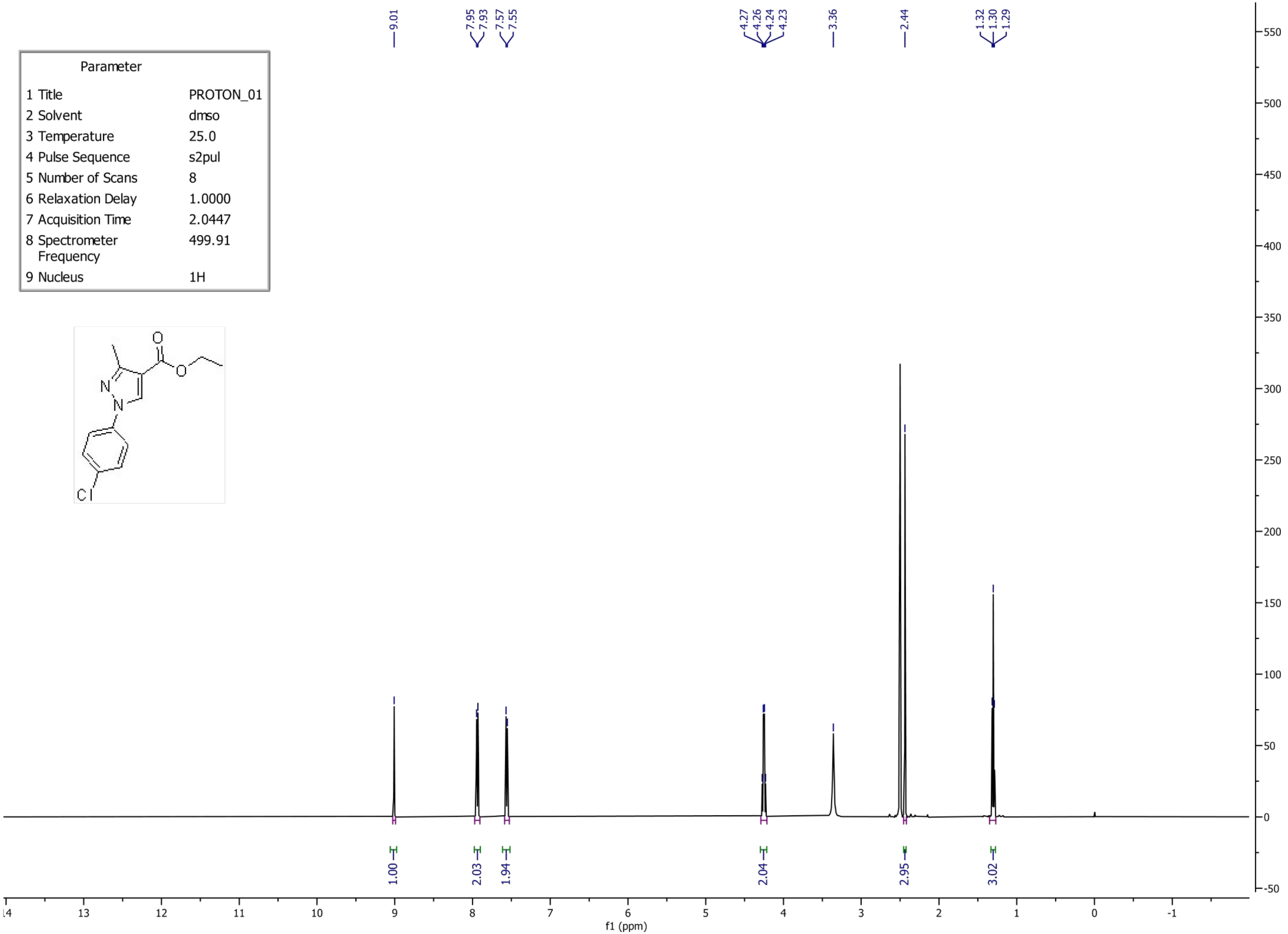

Figure S4 ^{1}H NMR spectrum of compound **1-2** (DMSO-d6, 500 MHz)

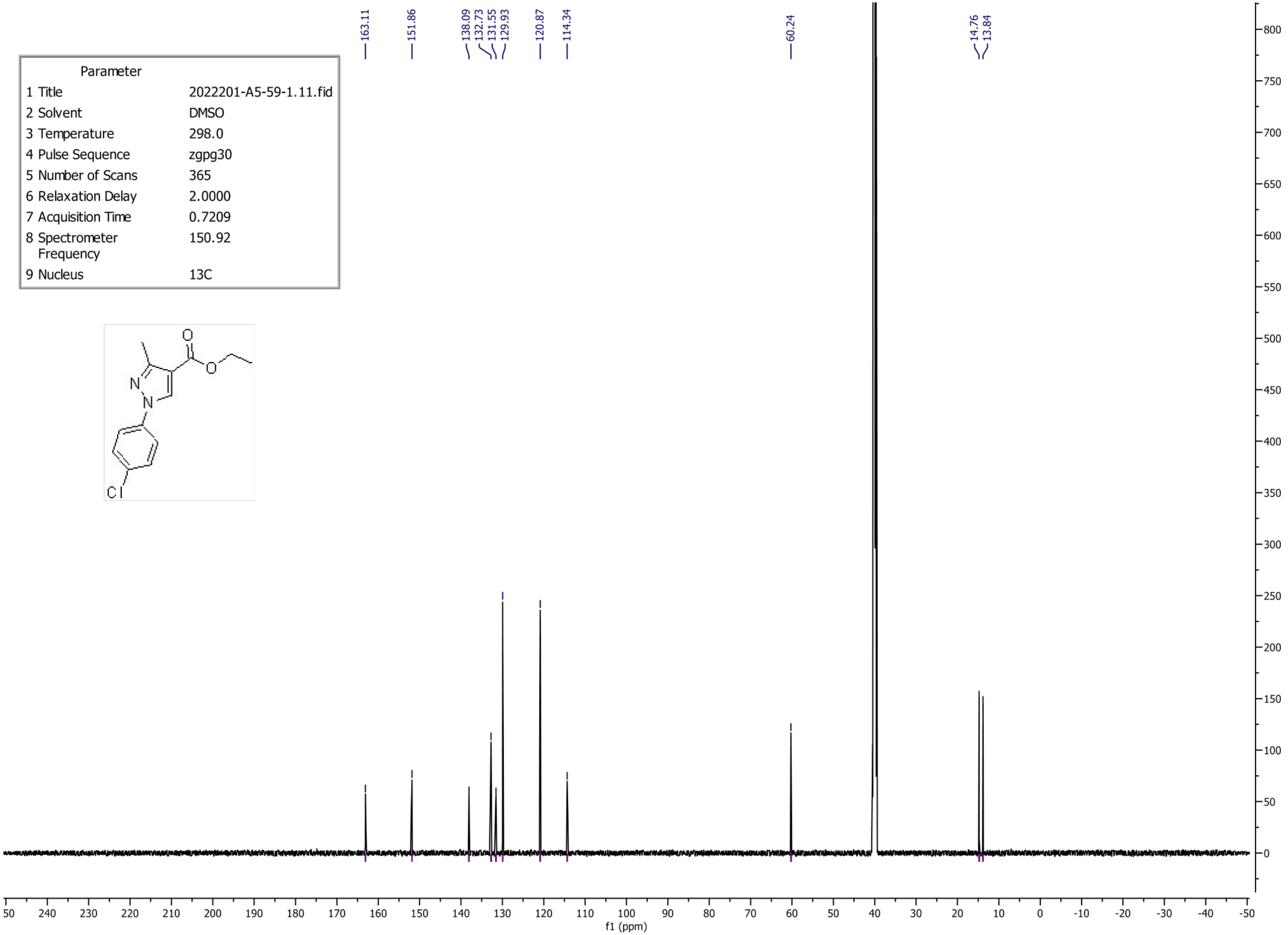

Figure S5 ^{13}C NMR spectrum of compound **1-2** (DMSO-d6, 151 MHz)

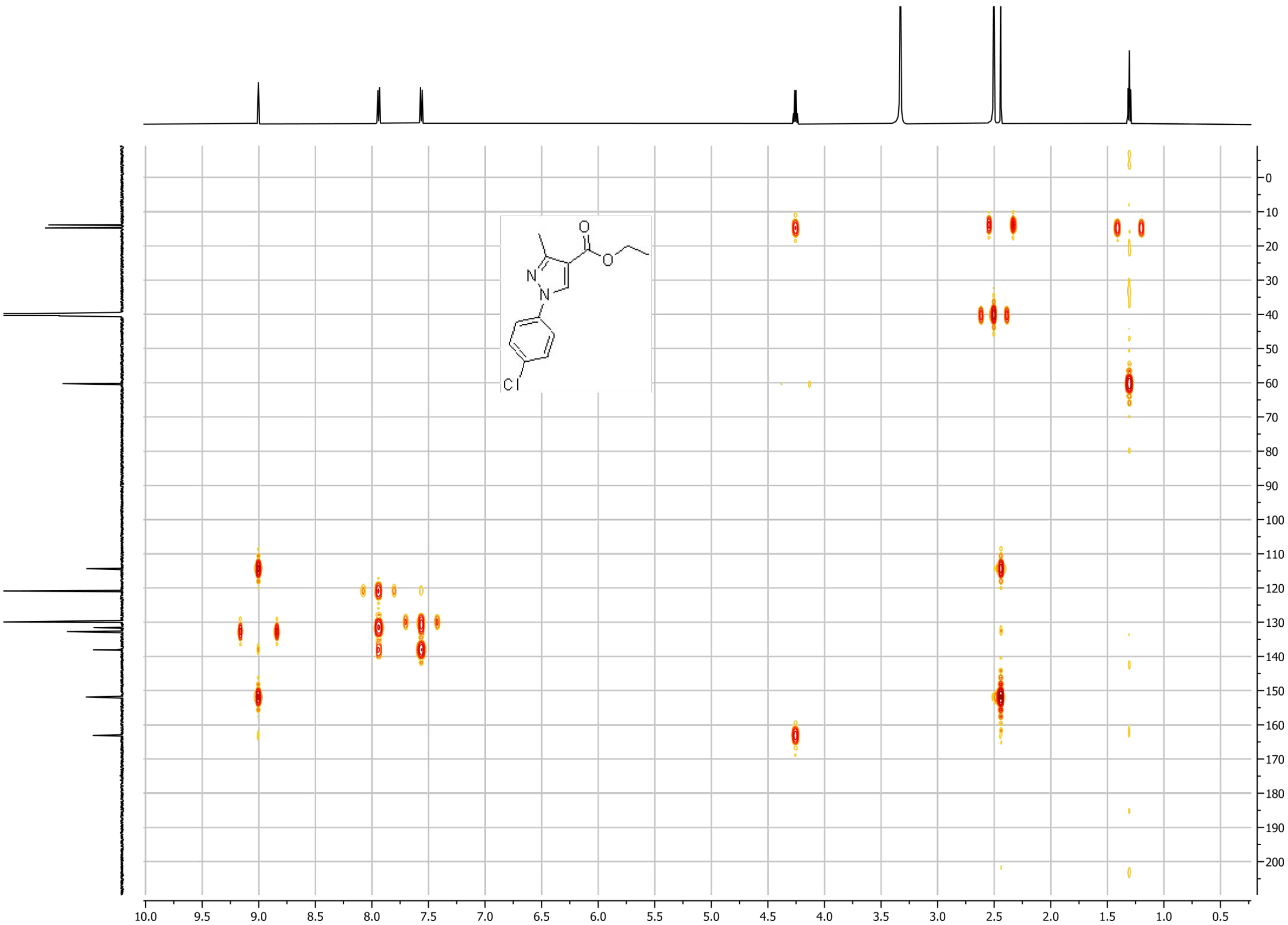

Figure S6 HMBC spectrum of compound **1-2** (DMSO-d6, 600 MHz)

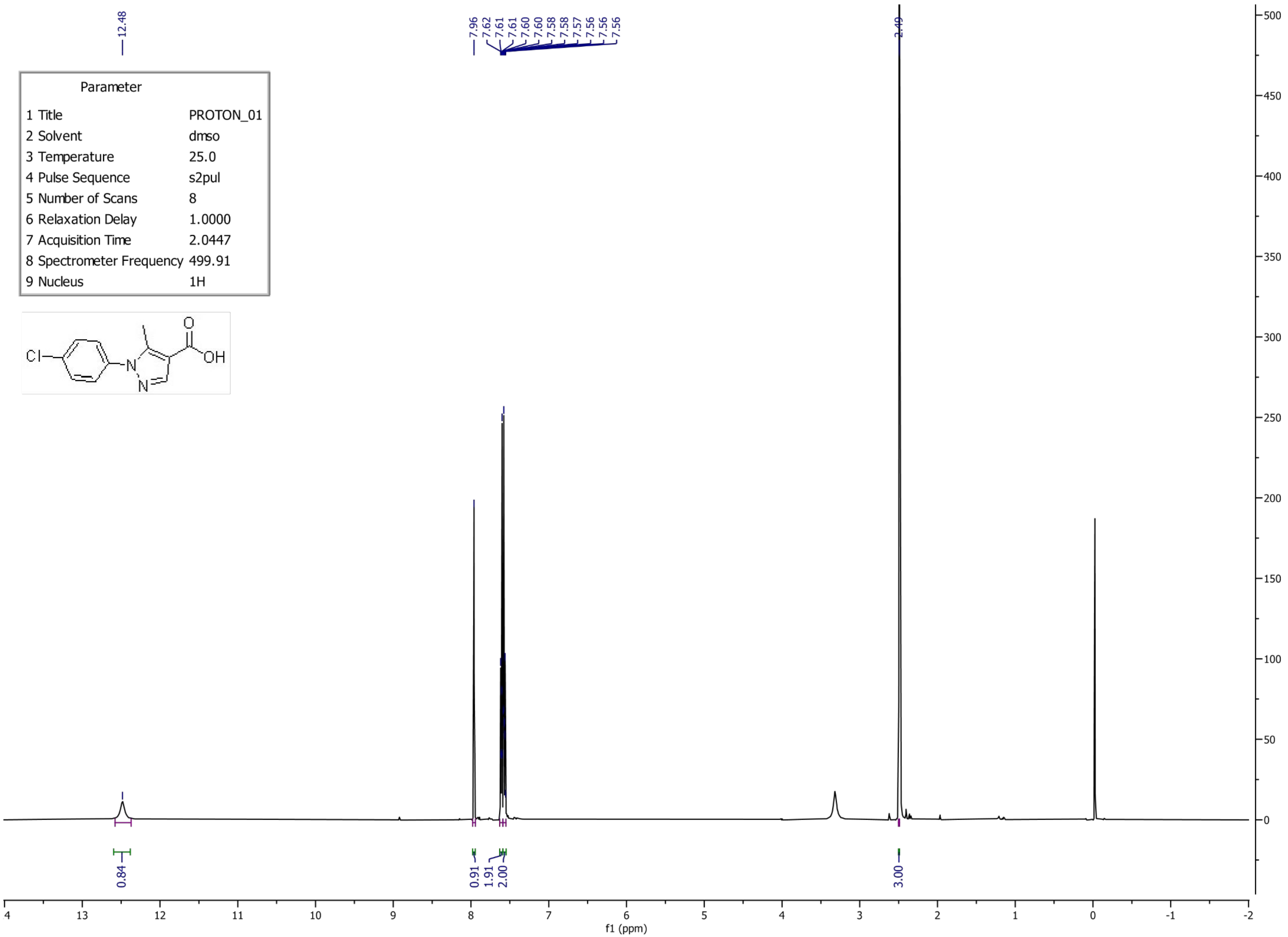

Figure S7 ^{1}H NMR spectrum of compound **2 (BY-020)** (DMSO-d6, 500 MHz)

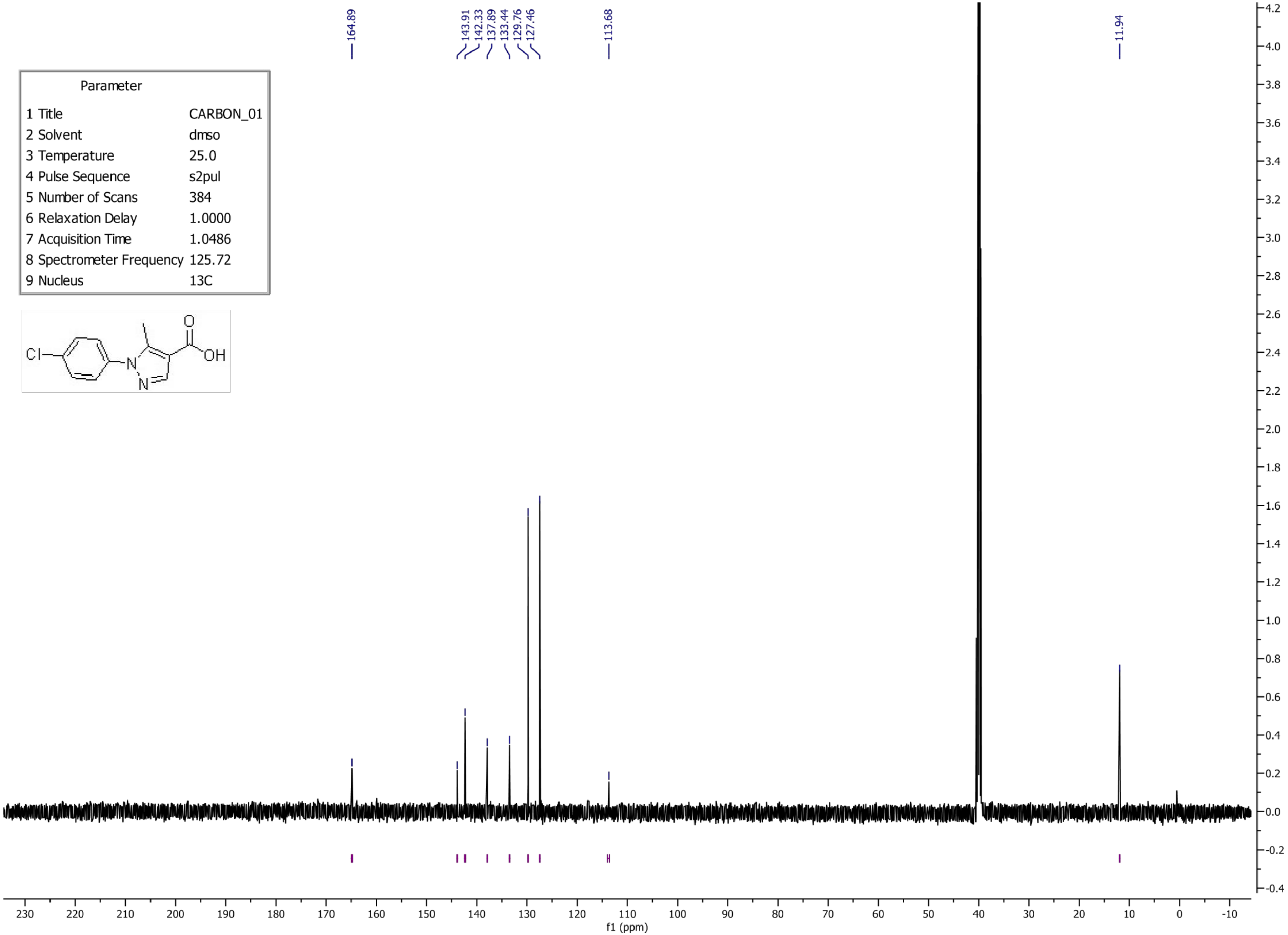

Figure S8 ^{13}C NMR spectrum of compound **2 (BY-020)** (DMSO-d6, 126 MHz)

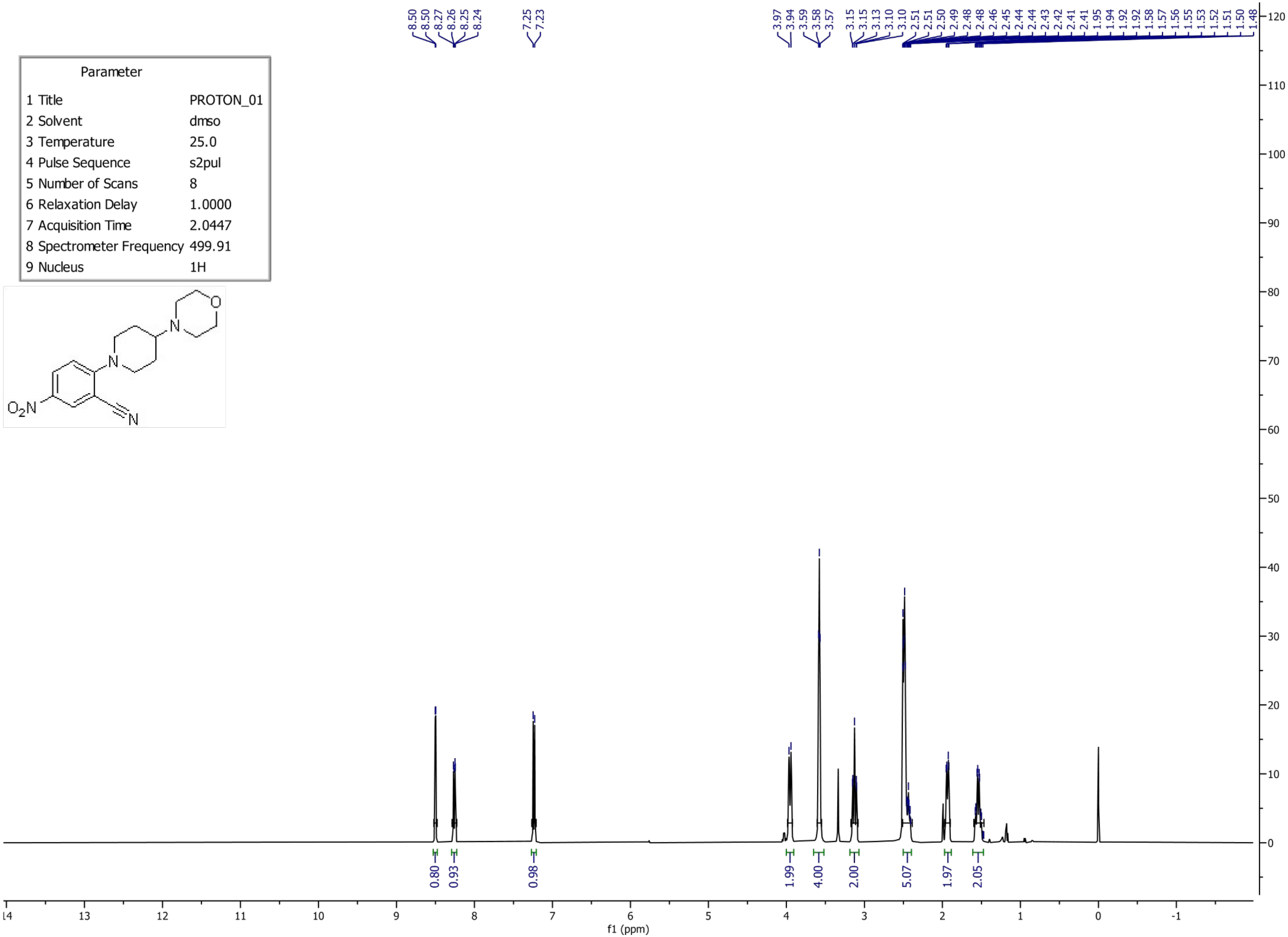

Figure S9 ^{1}H NMR spectrum of compound **3** (DMSO-d6, 500 MHz)

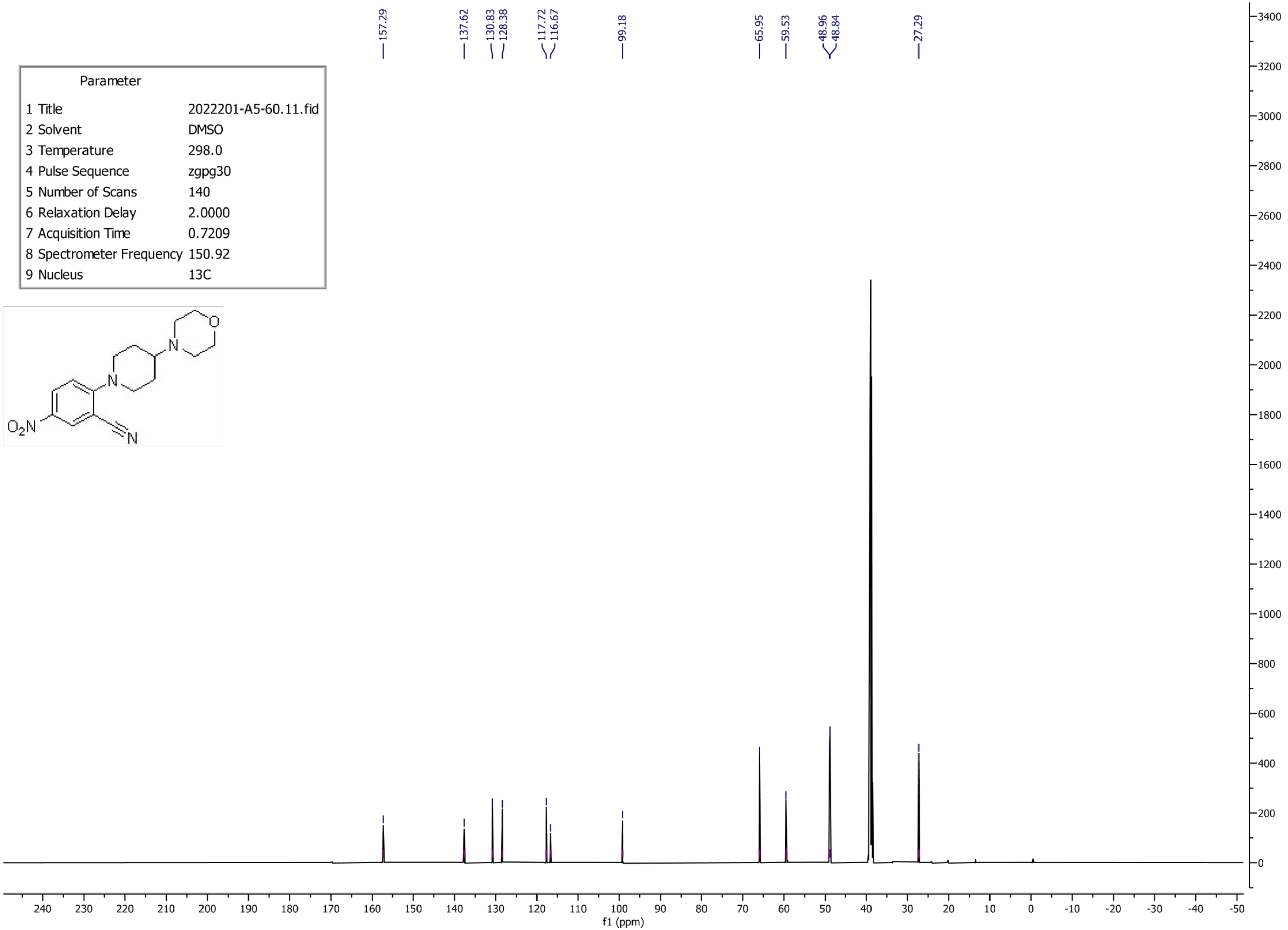

Figure S10 ^{13}C NMR spectrum of compound **3** (DMSO-d6, 151 MHz)

Parameter	
1 Title	PROTON_01
2 Solvent	dmso
3 Temperature	25.0
4 Pulse Sequence	s2pul
5 Number of Scans	8
6 Relaxation Delay	1.0000
7 Acquisition Time	2.0447
8 Spectrometer Frequency	499.91
9 Nucleus	1H

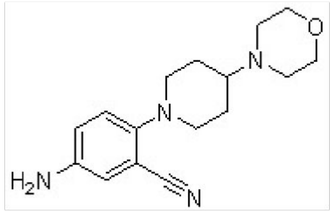

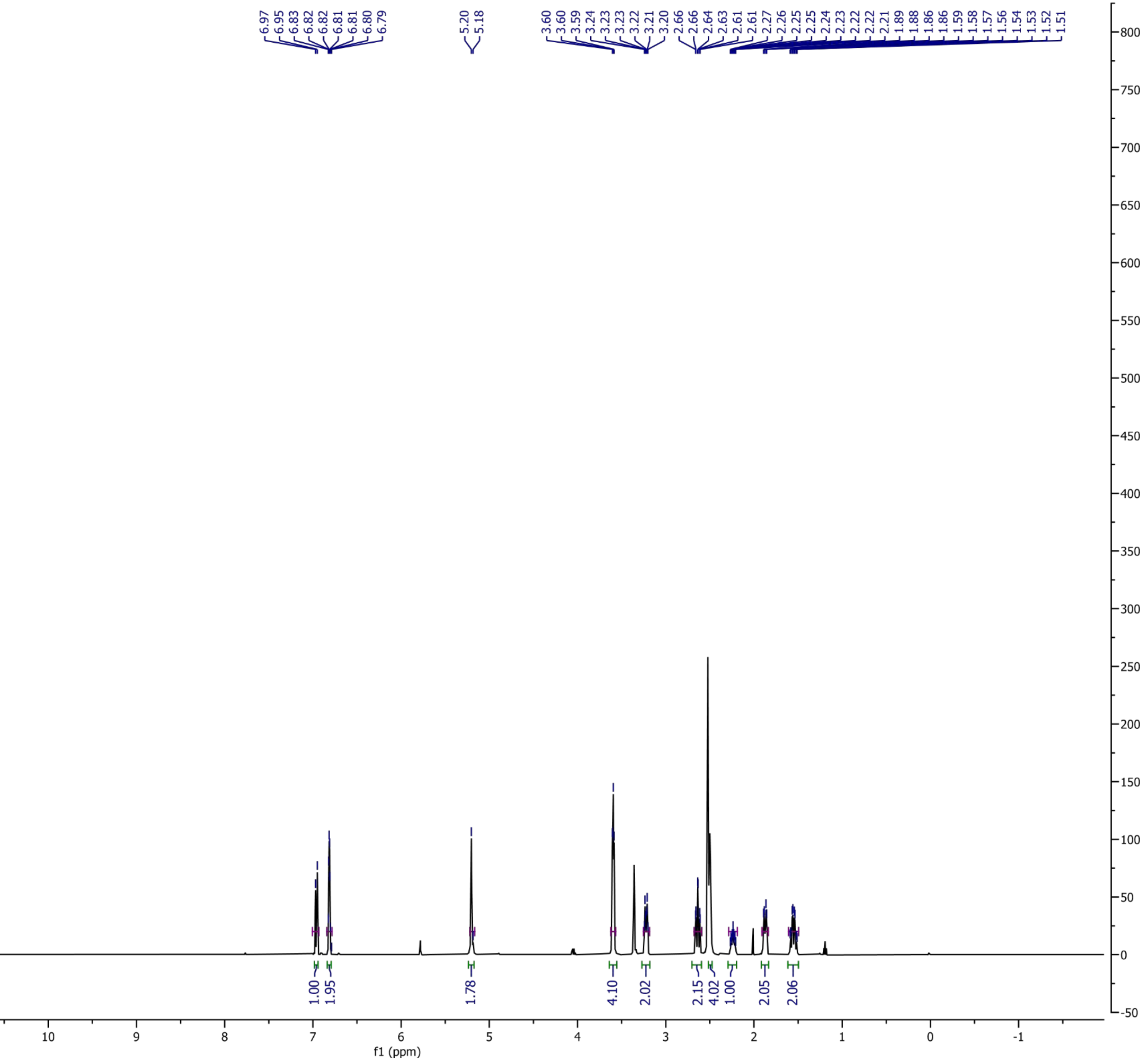

Figure S11 ^{1}H NMR spectrum of compound **4 (BY-021)** (DMSO-d6, 500 MHz)

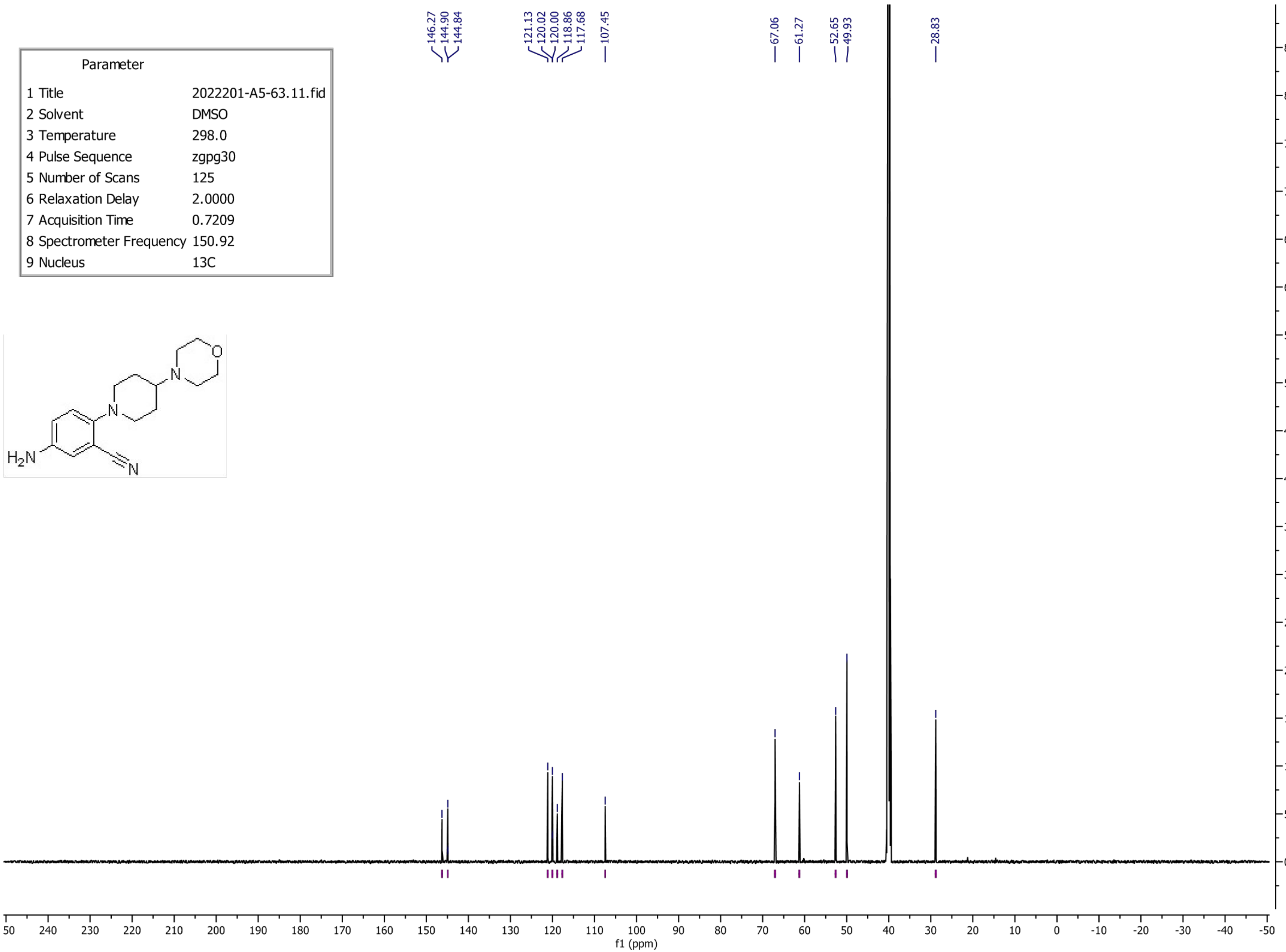

Figure S12 ^{13}C NMR spectrum of compound **4 (BY-021)** (DMSO-d6, 151 MHz)

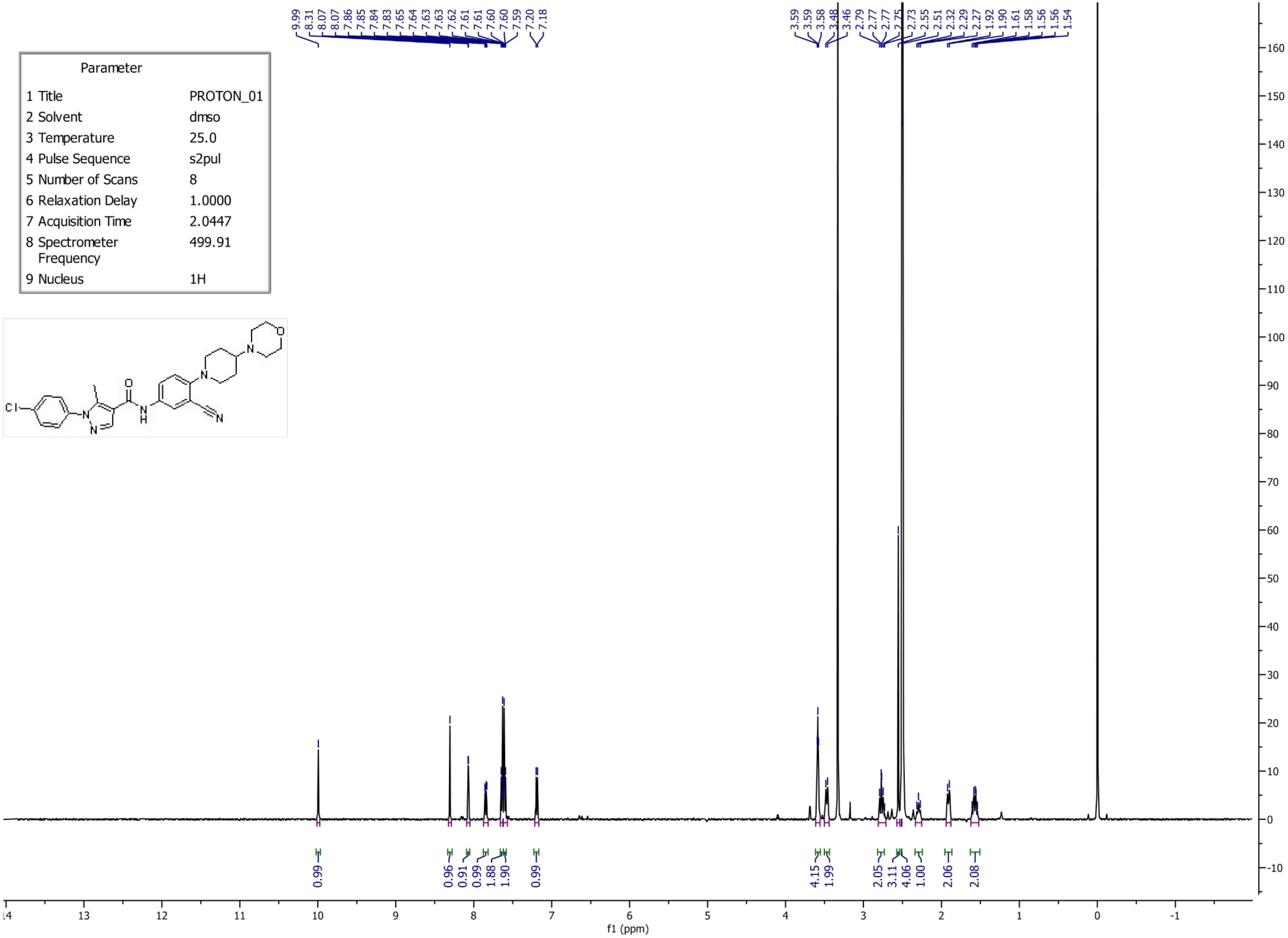

Figure S13 ^{1}H NMR spectrum of compound **5 (Y-320)** (DMSO-d6, 500 MHz)

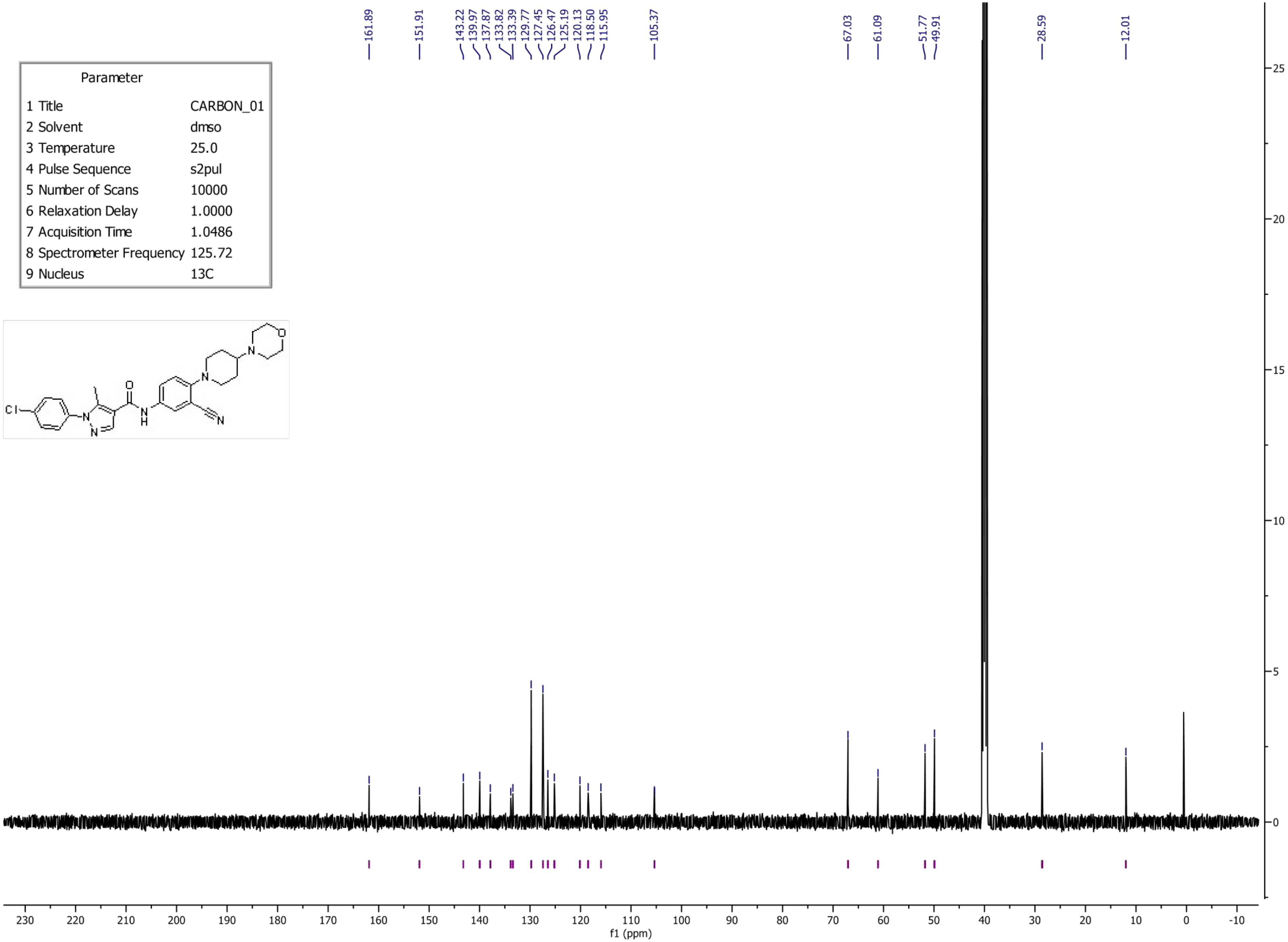

Figure S14 ^{13}C NMR spectrum of compound **5 (Y-320)** (DMSO-d6, 126 MHz)

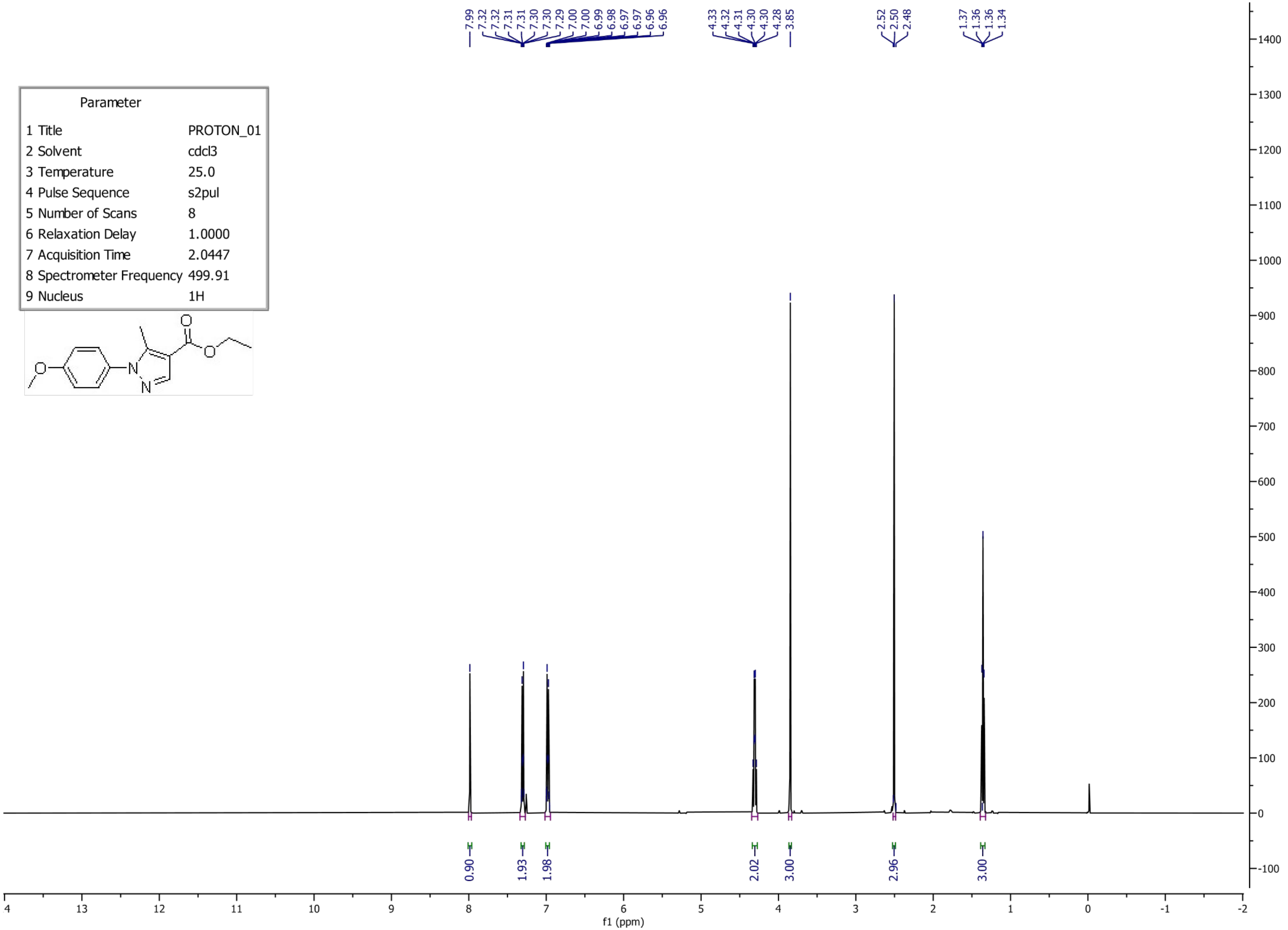

Figure S15 ^{1}H NMR spectrum of compound **6-1** ($CDCl_3$, 500 MHz)

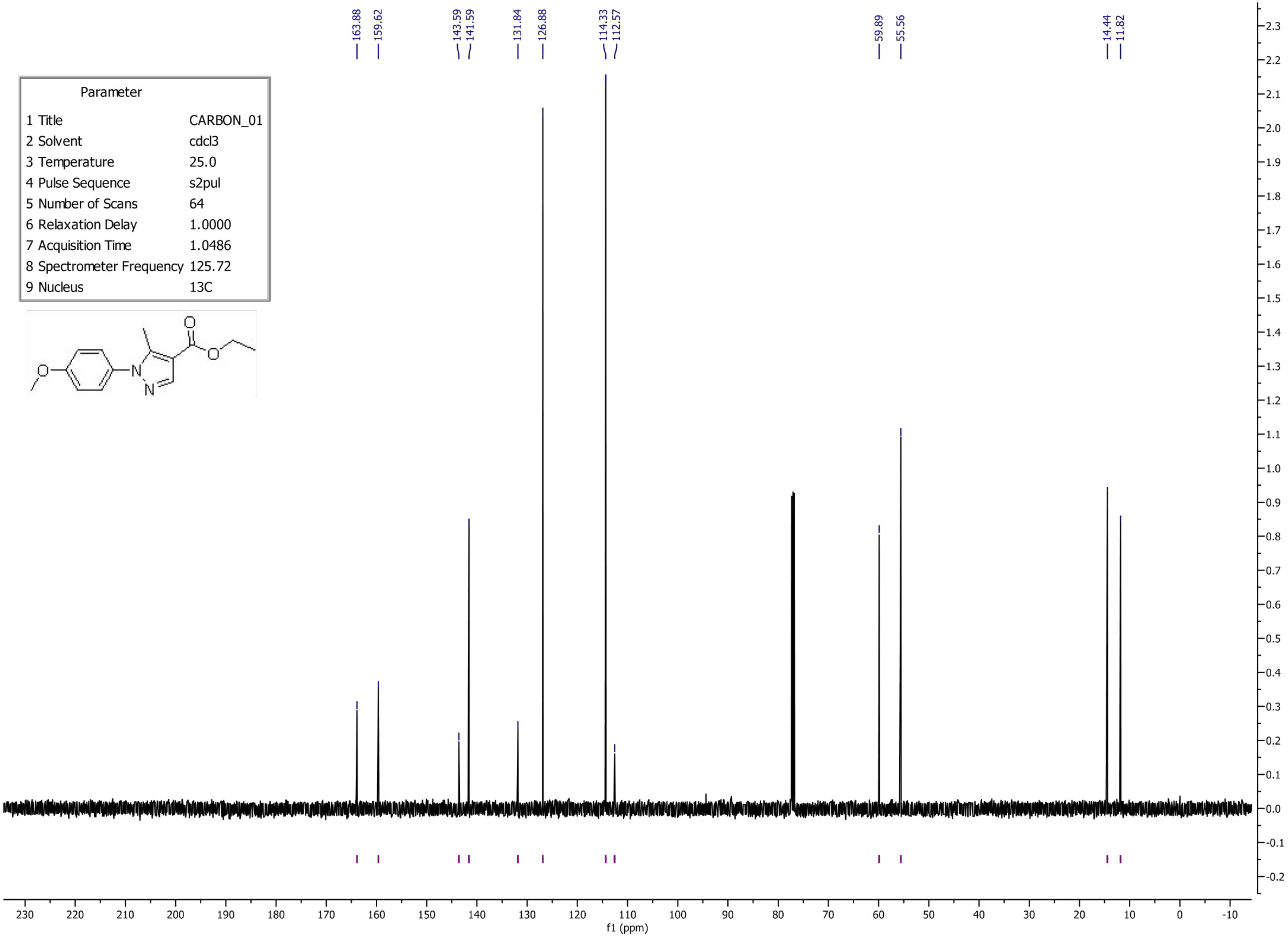

Figure S16 ^{13}C NMR spectrum of compound **6-1** ($CDCl_3$, 126 MHz)

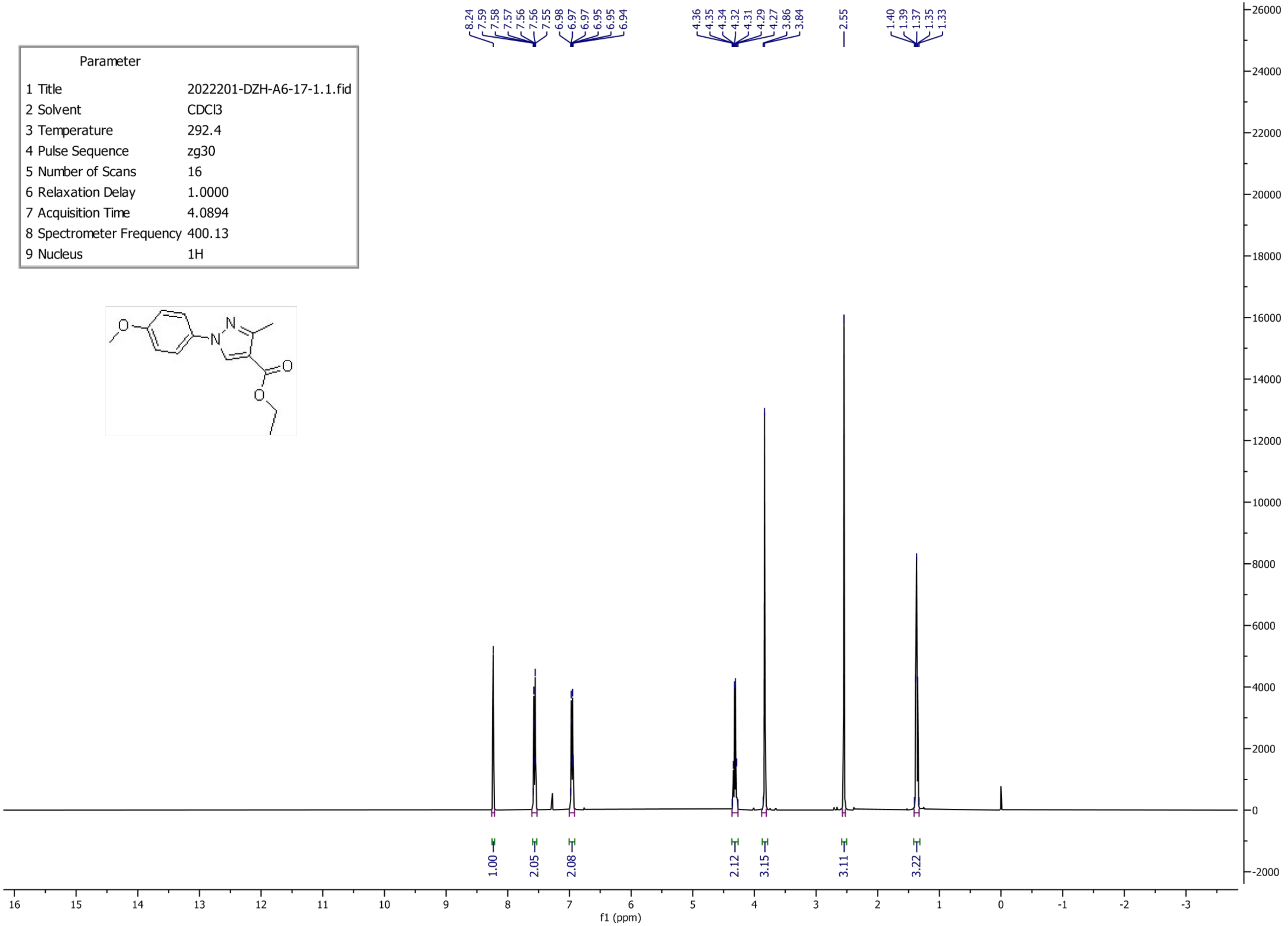

Figure S17 ^{1}H NMR spectrum of compound **6-2** ($CDCl_3$, 400 MHz)

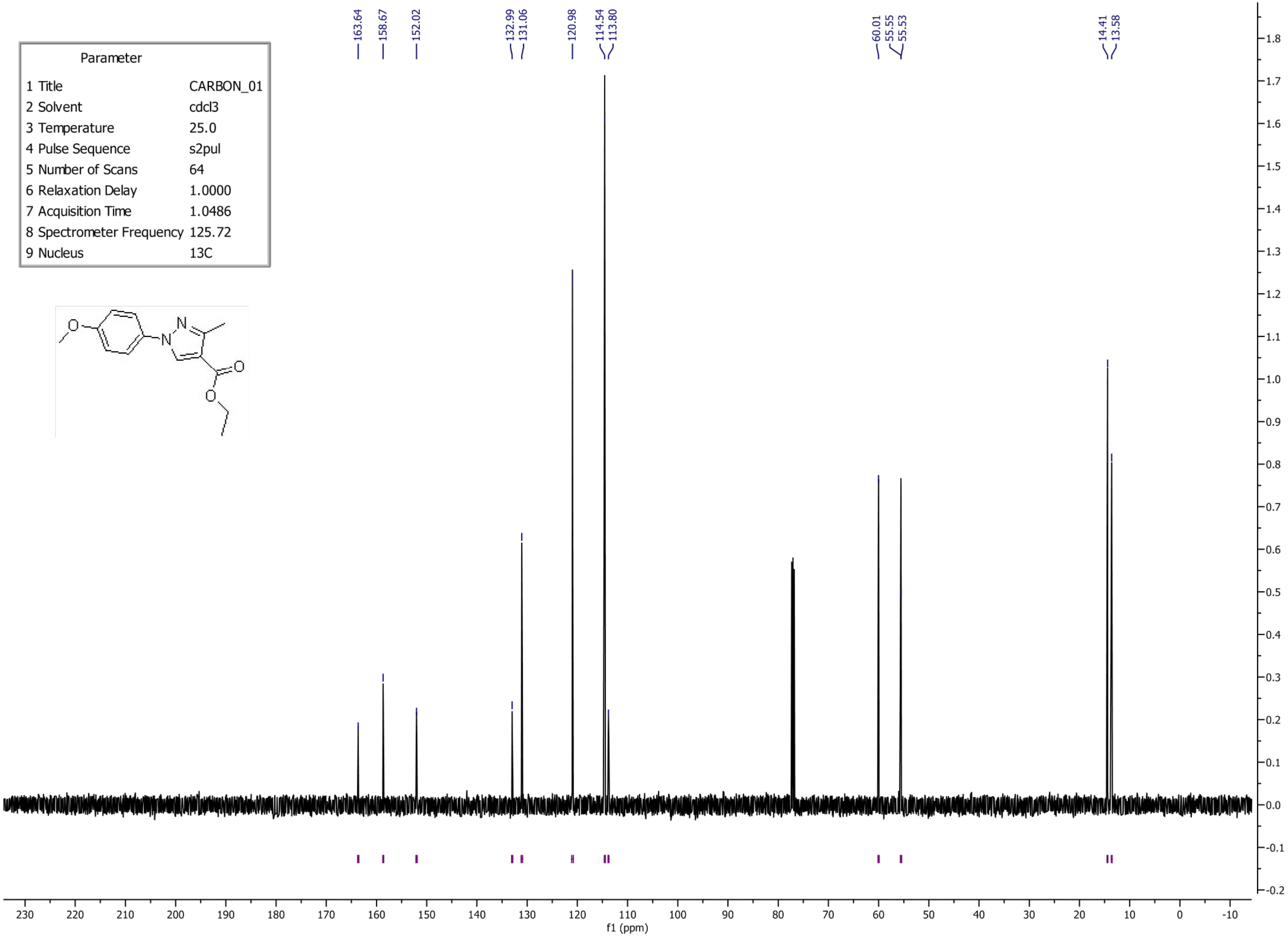

Figure S18 ^{13}C NMR spectrum of compound **6-2** ($CDCl_3$, 126 MHz)

Parameter	
1 Title	PROTON_01
2 Solvent	cdcl3
3 Temperature	25.0
4 Pulse Sequence	s2pul
5 Number of Scans	8
6 Relaxation Delay	1.0000
7 Acquisition Time	2.0447
8 Spectrometer Frequency	499.91
9 Nucleus	1H

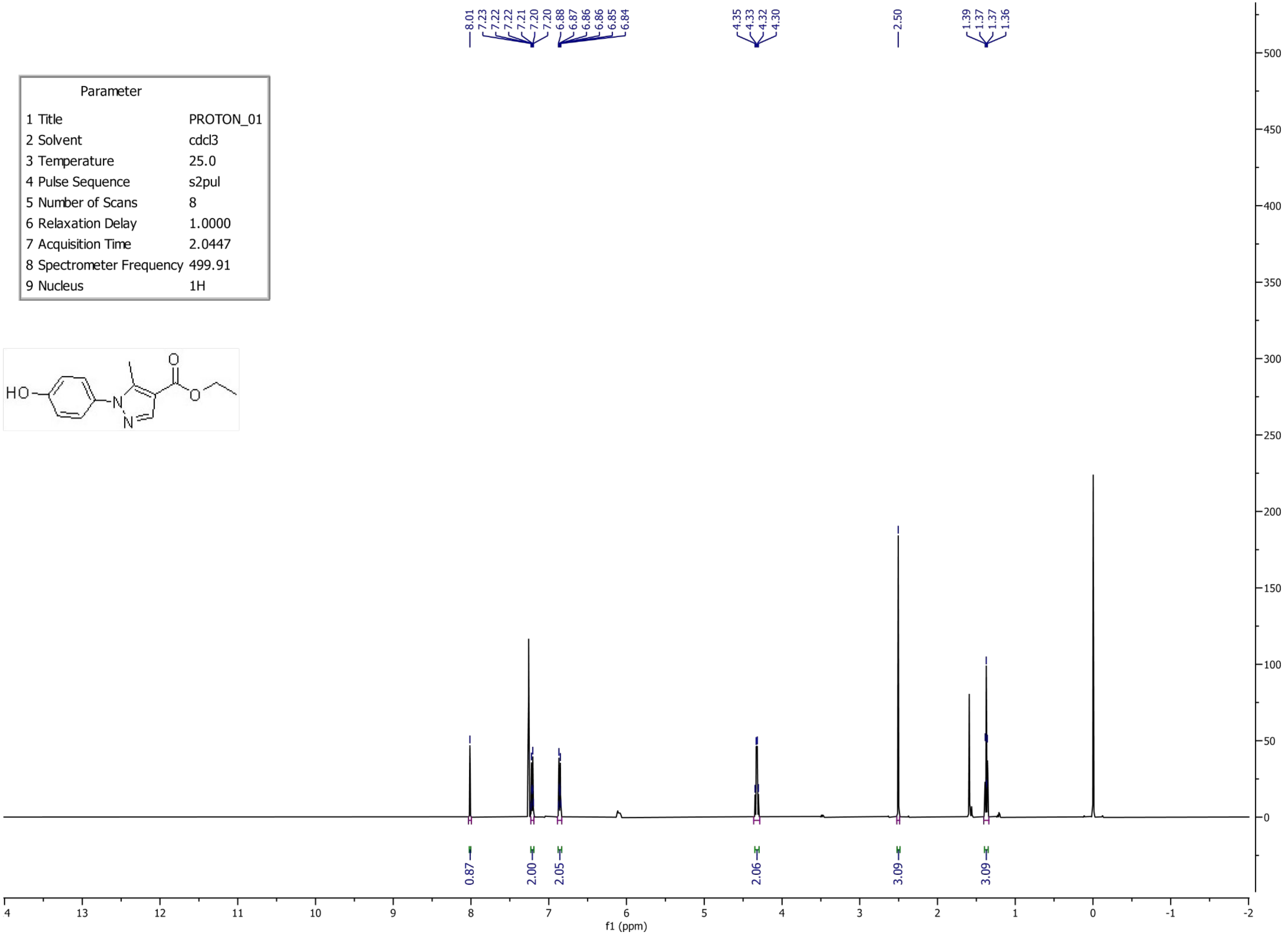

Figure S19 ^{1}H NMR spectrum of compound **7** ($CDCl_3$, 500 MHz)

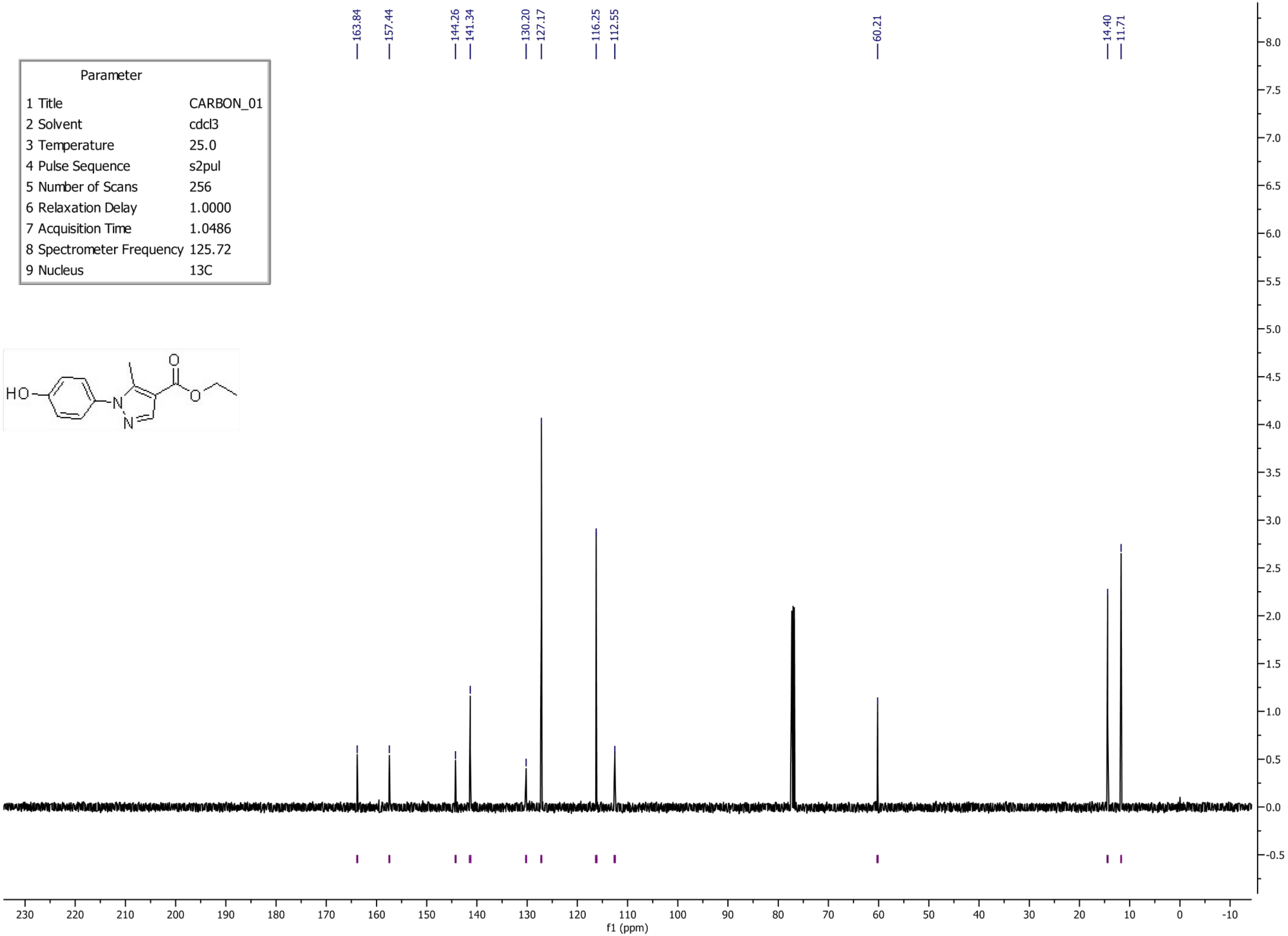

Figure S20 ^{13}C NMR spectrum of compound **7** ($CDCl_3$, 126 MHz)

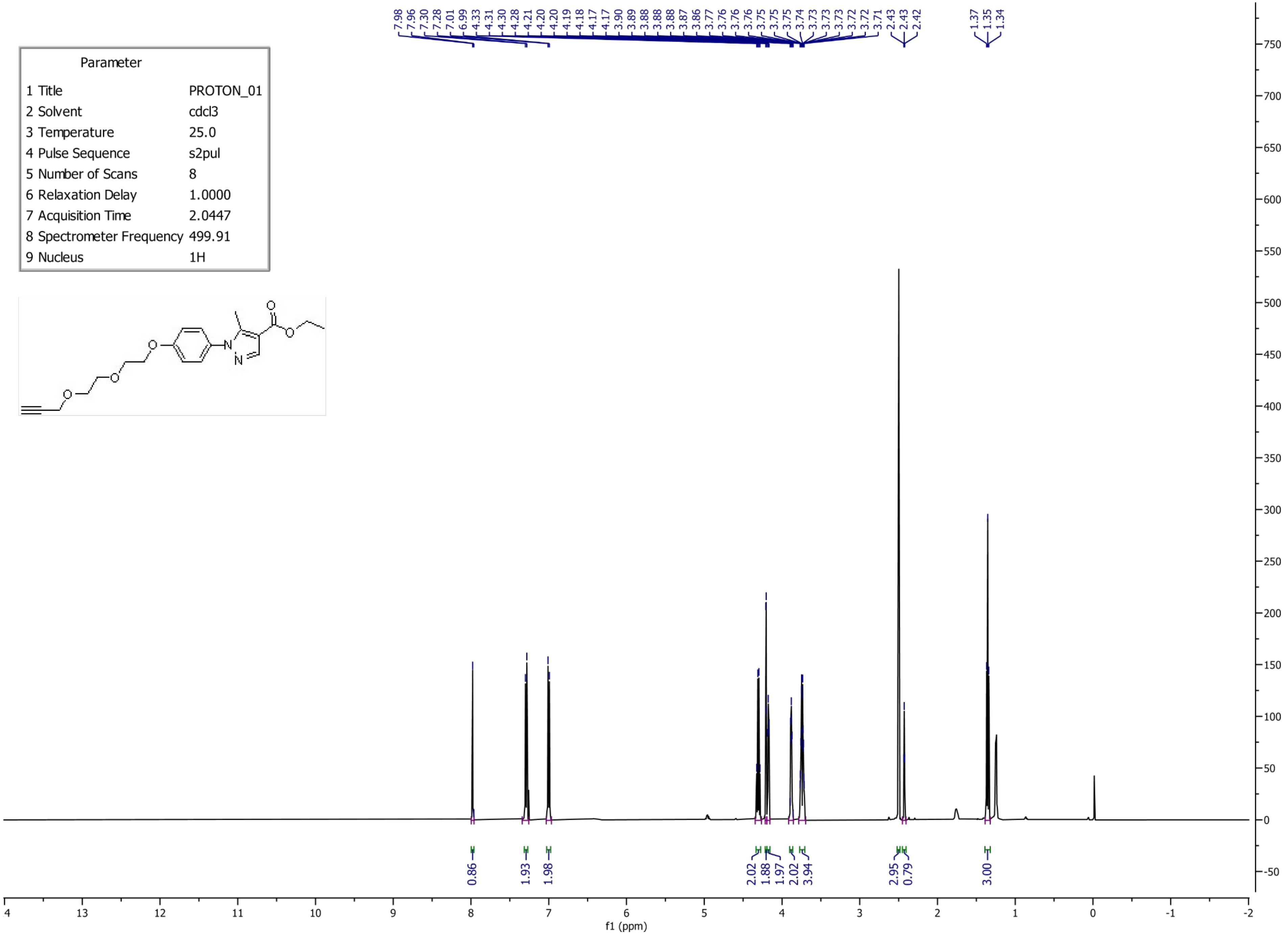

Figure S21 ^{1}H NMR spectrum of compound **8** ($CDCl_3$, 500 MHz)

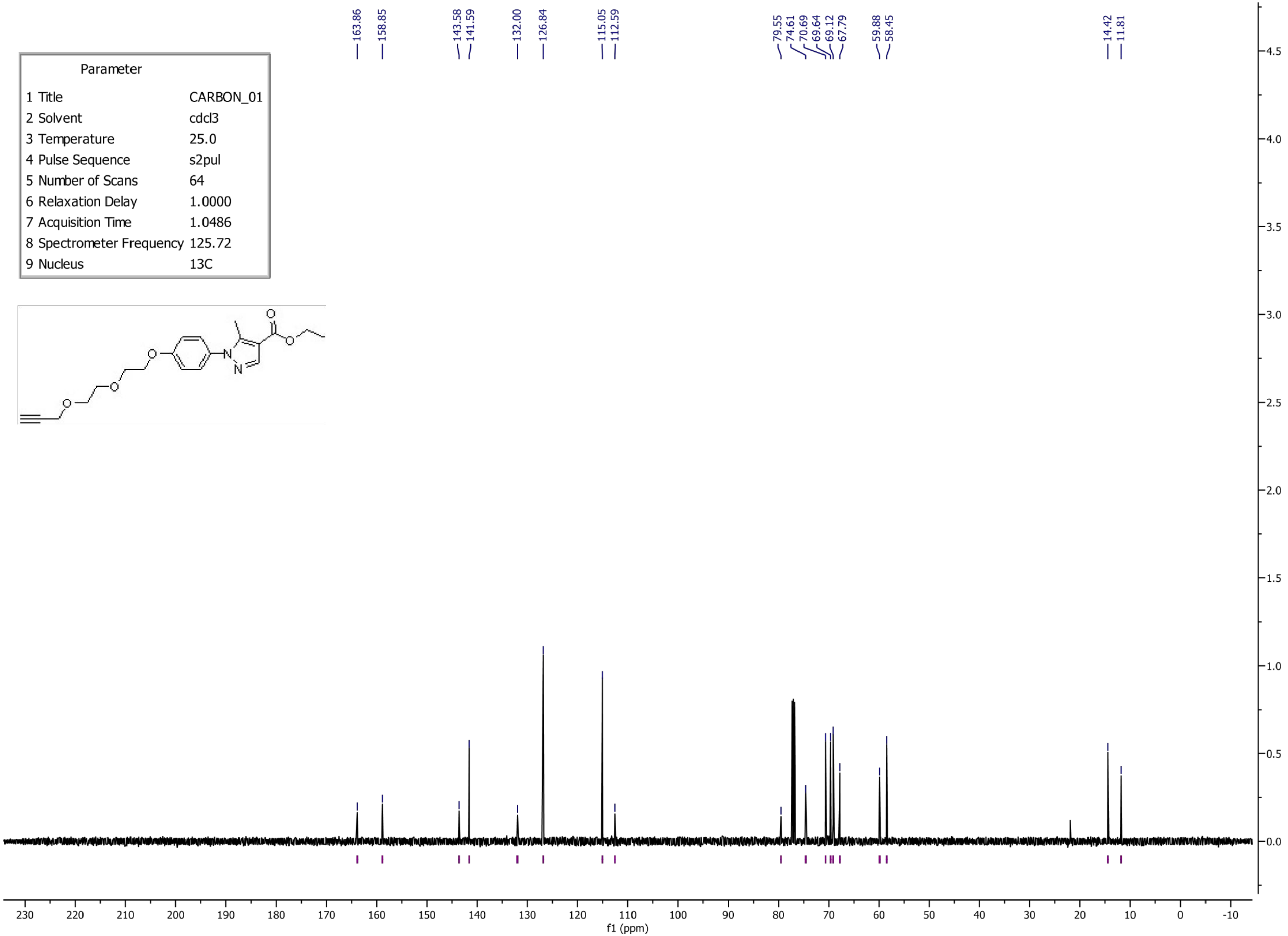

Figure S22 ^{13}C NMR spectrum of compound **8** ($CDCl_3$, 126 MHz)

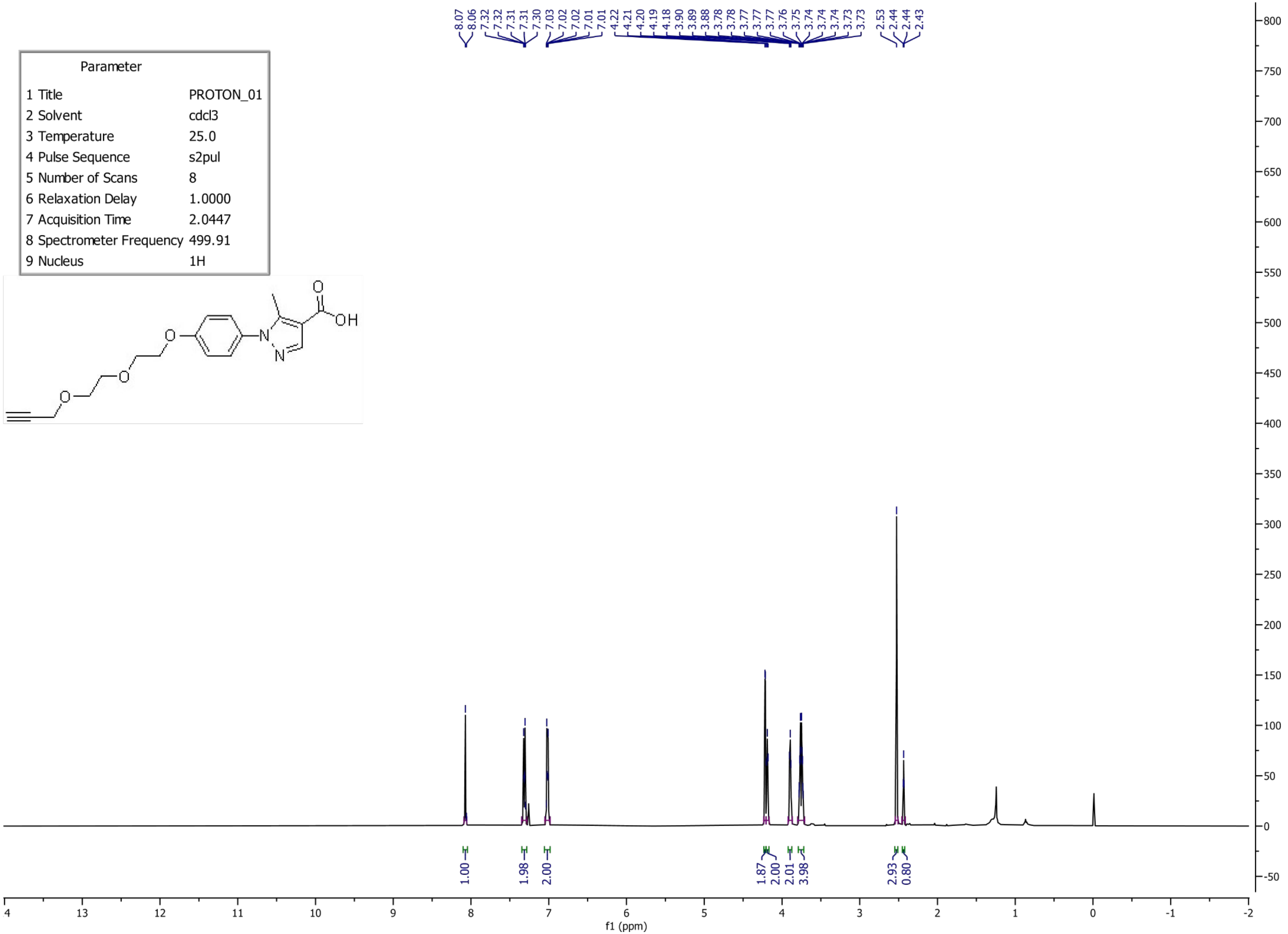

Figure S23 ^{1}H NMR spectrum of compound **9** ($CDCl_3$, 500 MHz)

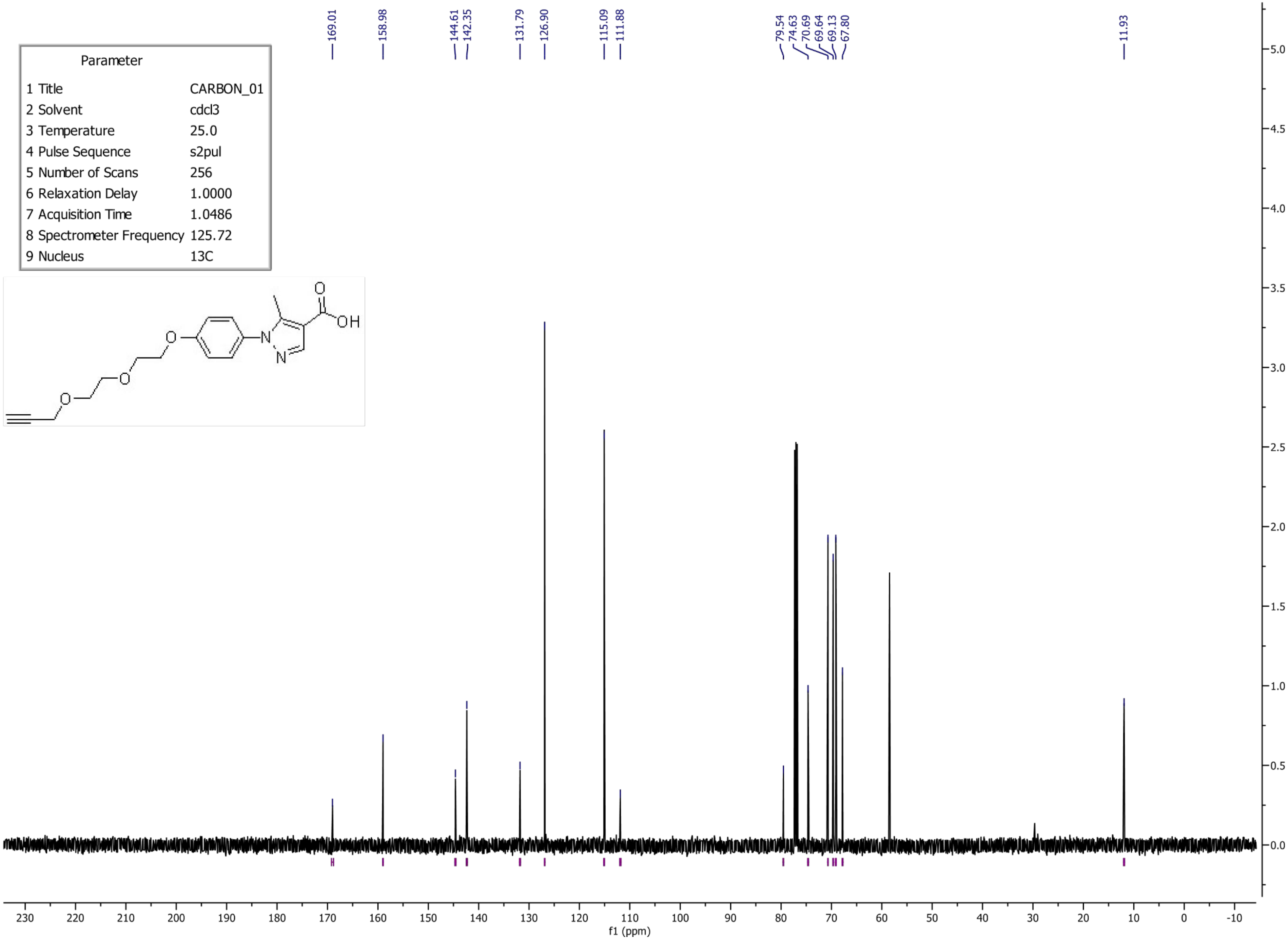

Figure S24 ^{13}C NMR spectrum of compound **9** ($CDCl_3$, 126 MHz)

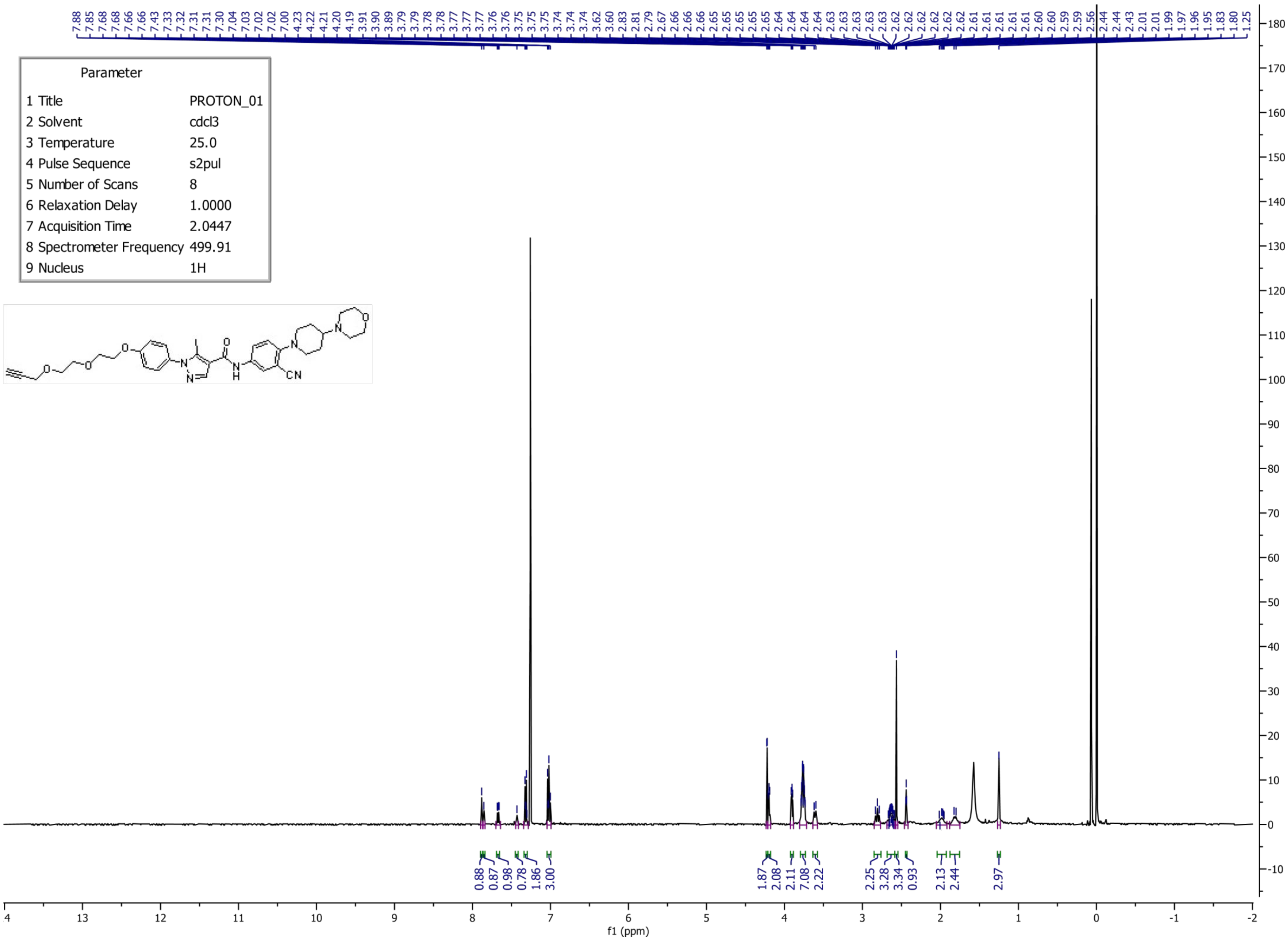

Figure S25 ^{1}H NMR spectrum of compound **10** ($CDCl_3$, 500 MHz)

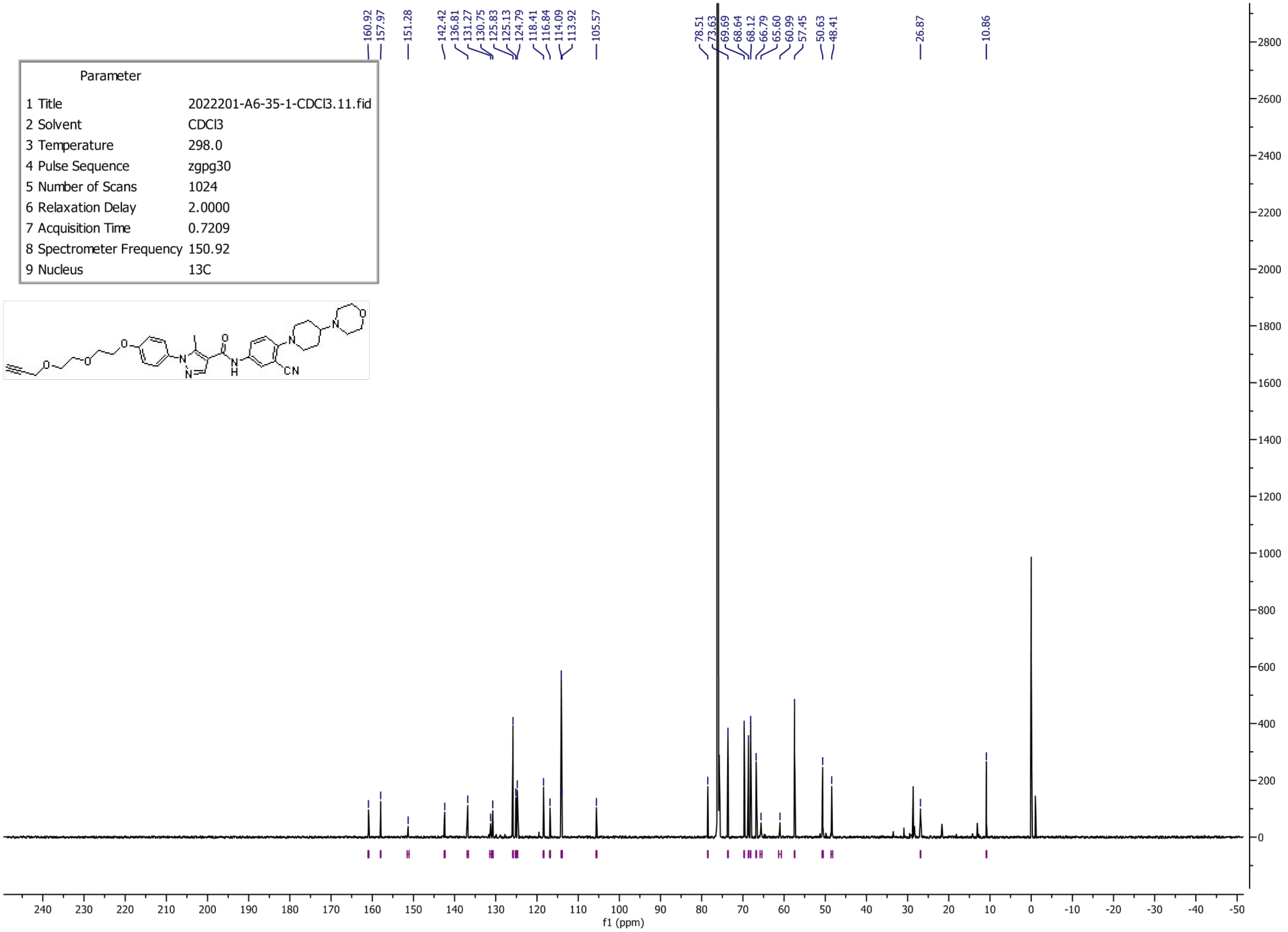

Figure S26 ^{13}C NMR spectrum of compound **10** ($CDCl_3$, 151 MHz)

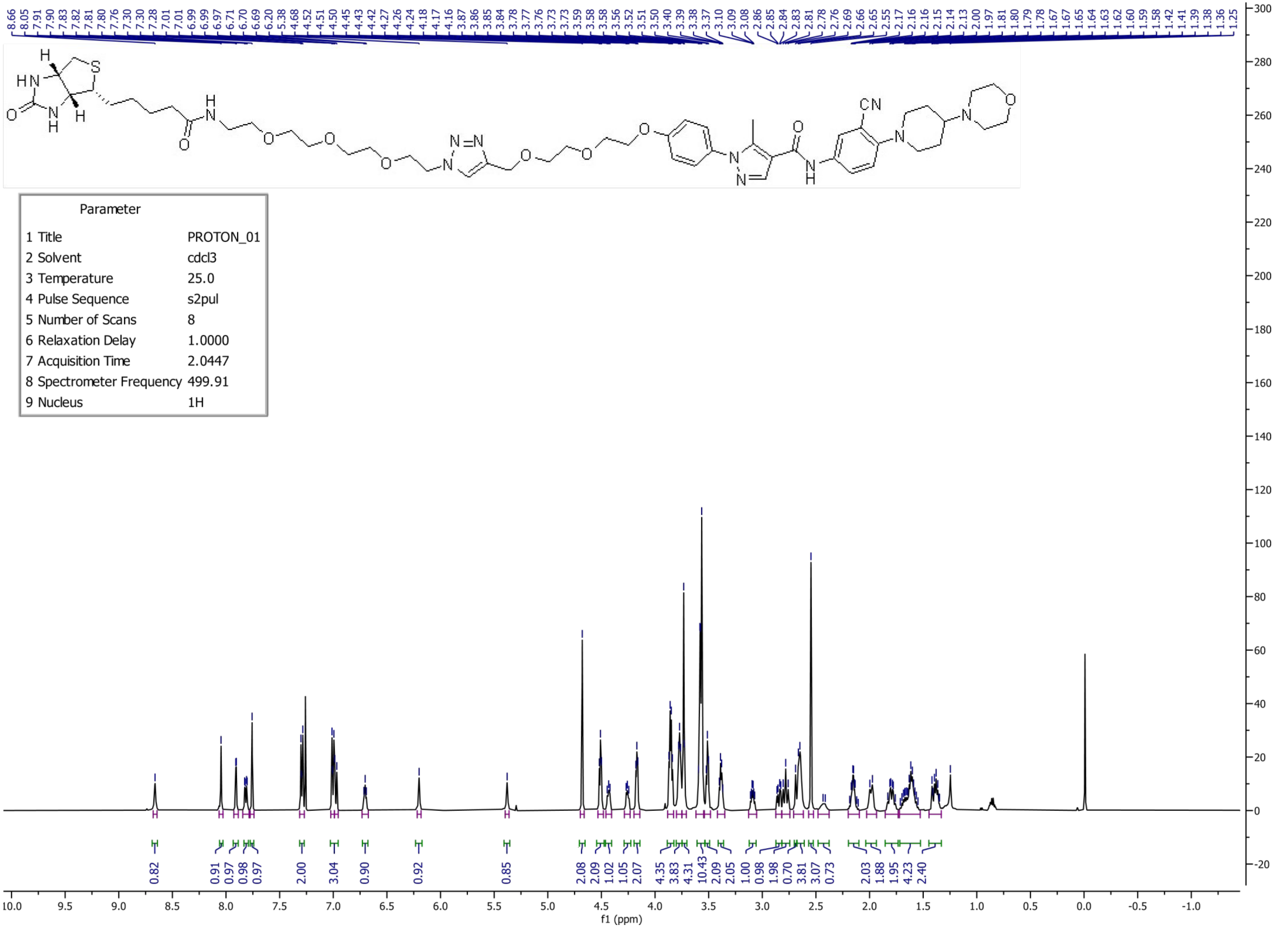

Figure S27 ^{1}H NMR spectrum of compound **11 (Y-320-Biotin)** ($CDCl_3$, 500 MHz)

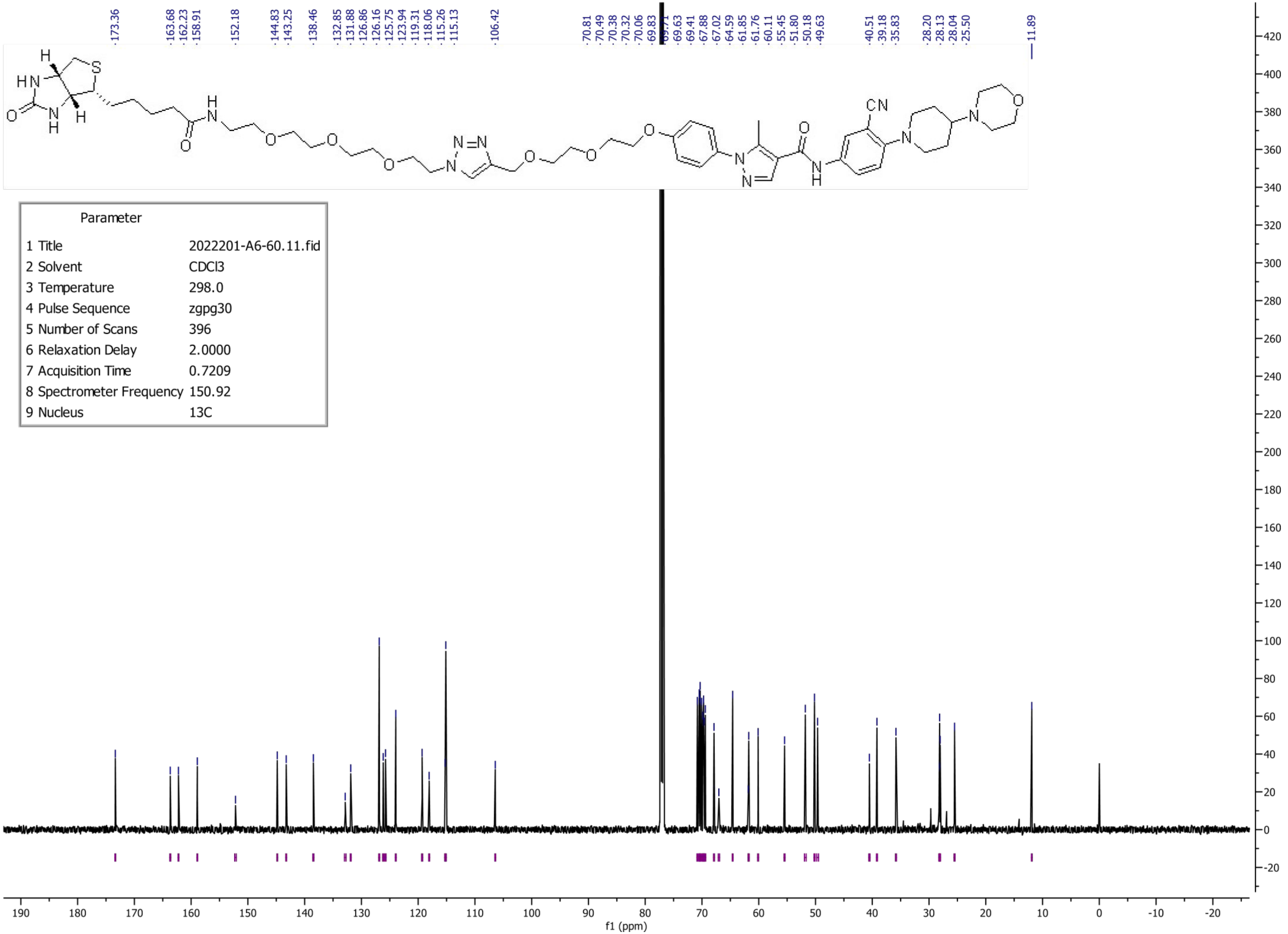

Figure S28 ^{13}C NMR spectrum of compound **11 (Y-320-Biotin)** ($CDCl_3$, 151 MHz)

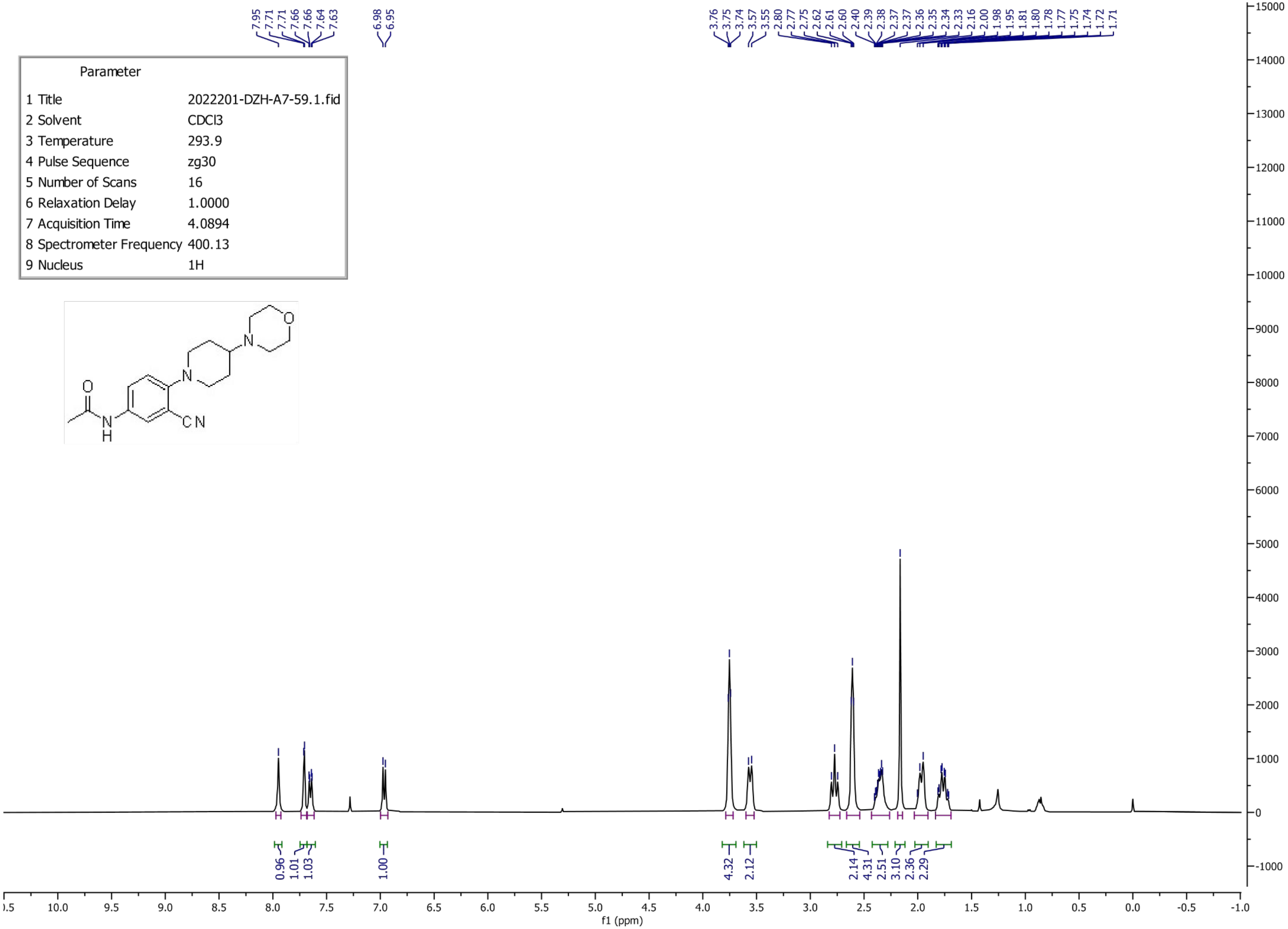

Figure S29 ^{1}H NMR spectrum of compound **12 (BY-023)** ($CDCl_3$, 400 MHz)

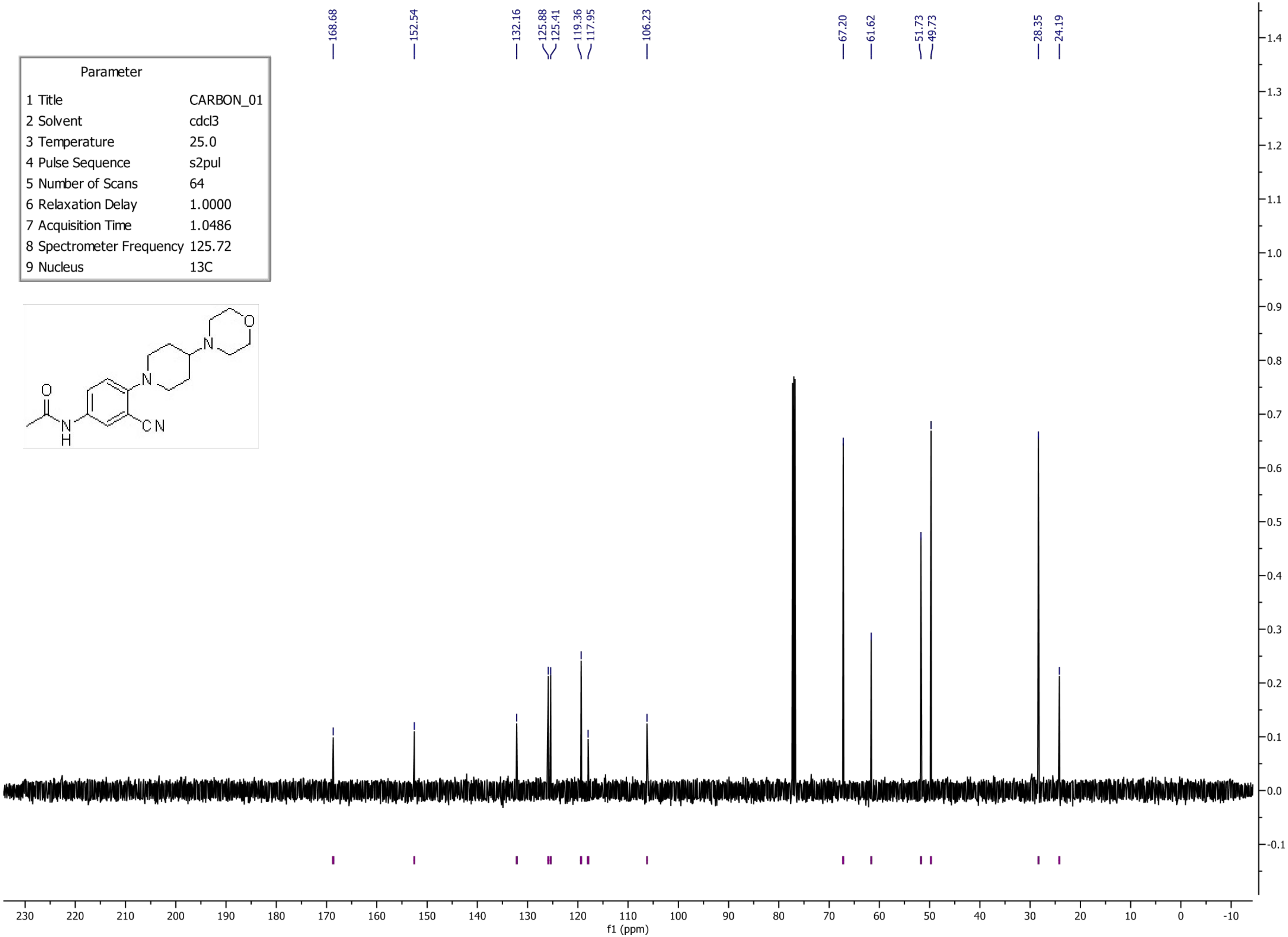

Figure S30 ^{13}C NMR spectrum of compound **12 (BY-023)** ($CDCl_3$, 126 MHz)

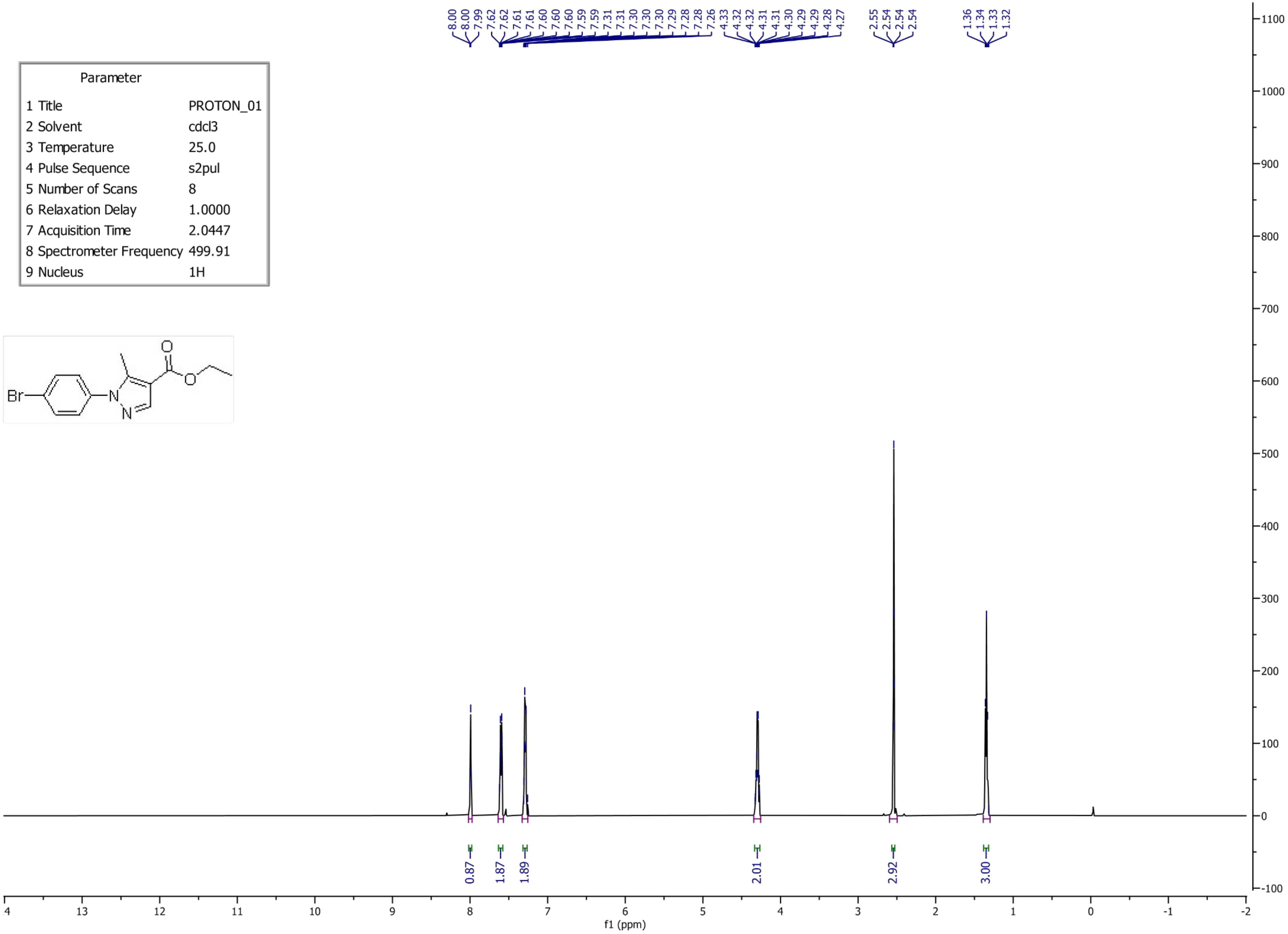

Parameter	
1 Title	PROTON_01
2 Solvent	cdcl3
3 Temperature	25.0
4 Pulse Sequence	s2pul
5 Number of Scans	8
6 Relaxation Delay	1.0000
7 Acquisition Time	2.0447
8 Spectrometer Frequency	499.91
9 Nucleus	1H

Figure S31 ^{1}H NMR spectrum of compound **13-1** ($CDCl_3$, 500 MHz)

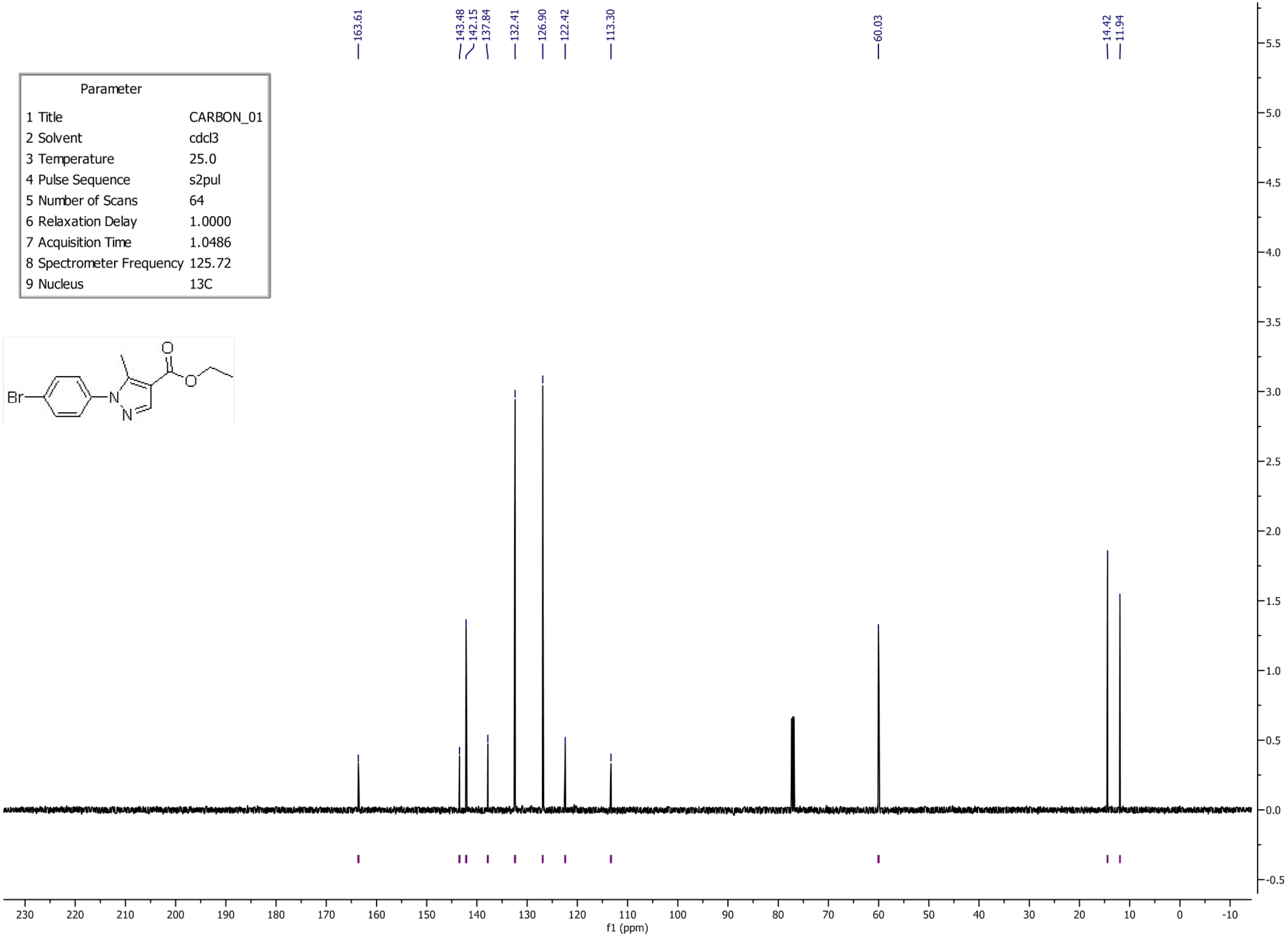

Parameter	
1 Title	CARBON_01
2 Solvent	cdcl3
3 Temperature	25.0
4 Pulse Sequence	s2pul
5 Number of Scans	64
6 Relaxation Delay	1.0000
7 Acquisition Time	1.0486
8 Spectrometer Frequency	125.72
9 Nucleus	13C

Figure S32 ^{13}C NMR spectrum of compound **13-1** ($CDCl_3$ 126 MHz)

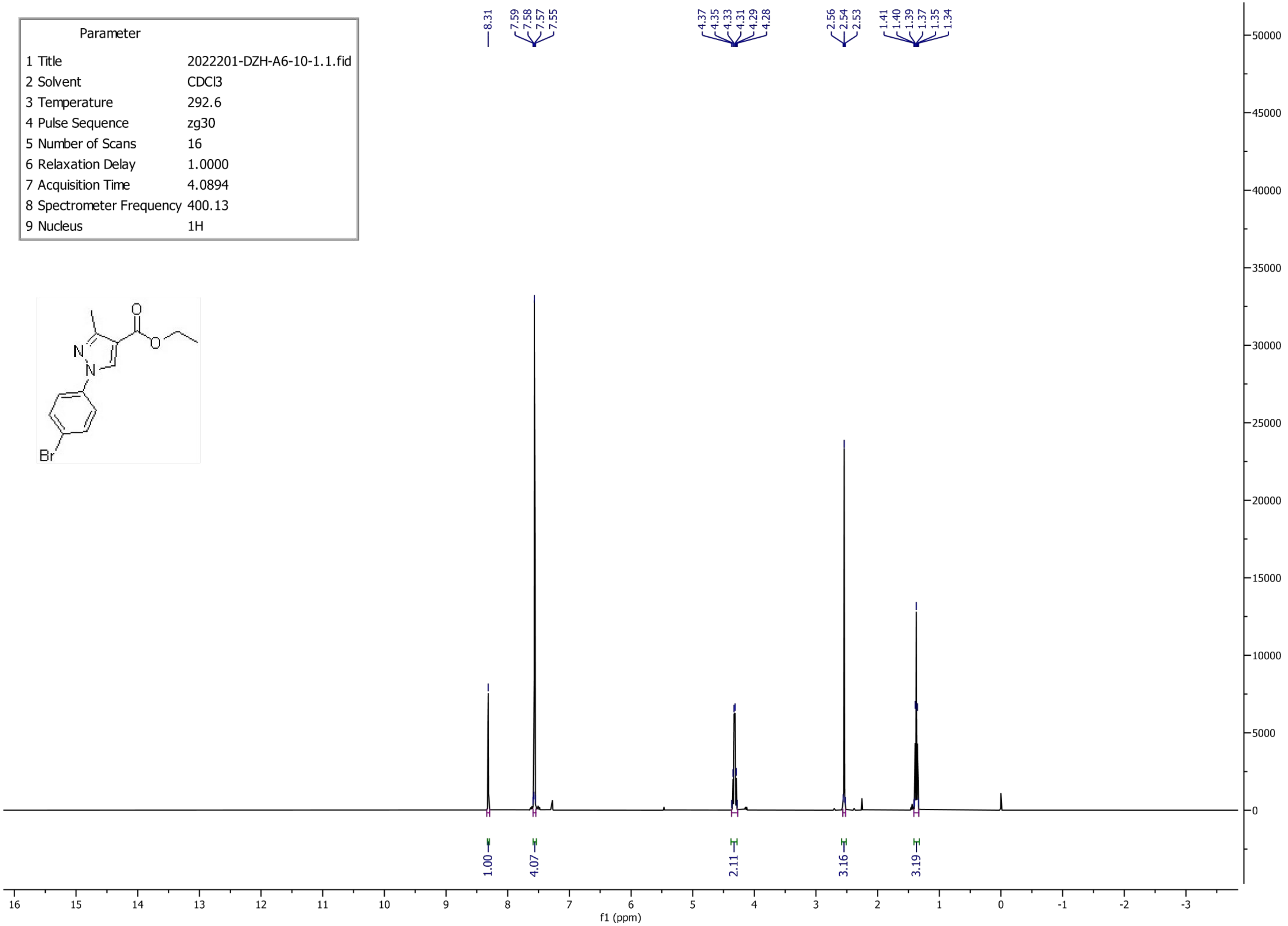

Figure S33 ^{1}H NMR spectrum of compound **13-2** ($CDCl_3$, 500 MHz)

Parameter	
1 Title	CARBON_01
2 Solvent	cdcl3
3 Temperature	25.0
4 Pulse Sequence	s2pul
5 Number of Scans	64
6 Relaxation Delay	1.0000
7 Acquisition Time	1.0486
8 Spectrometer Frequency	125.72
9 Nucleus	13C

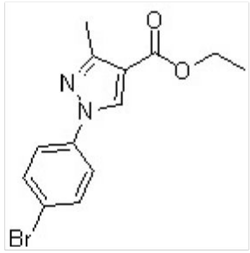

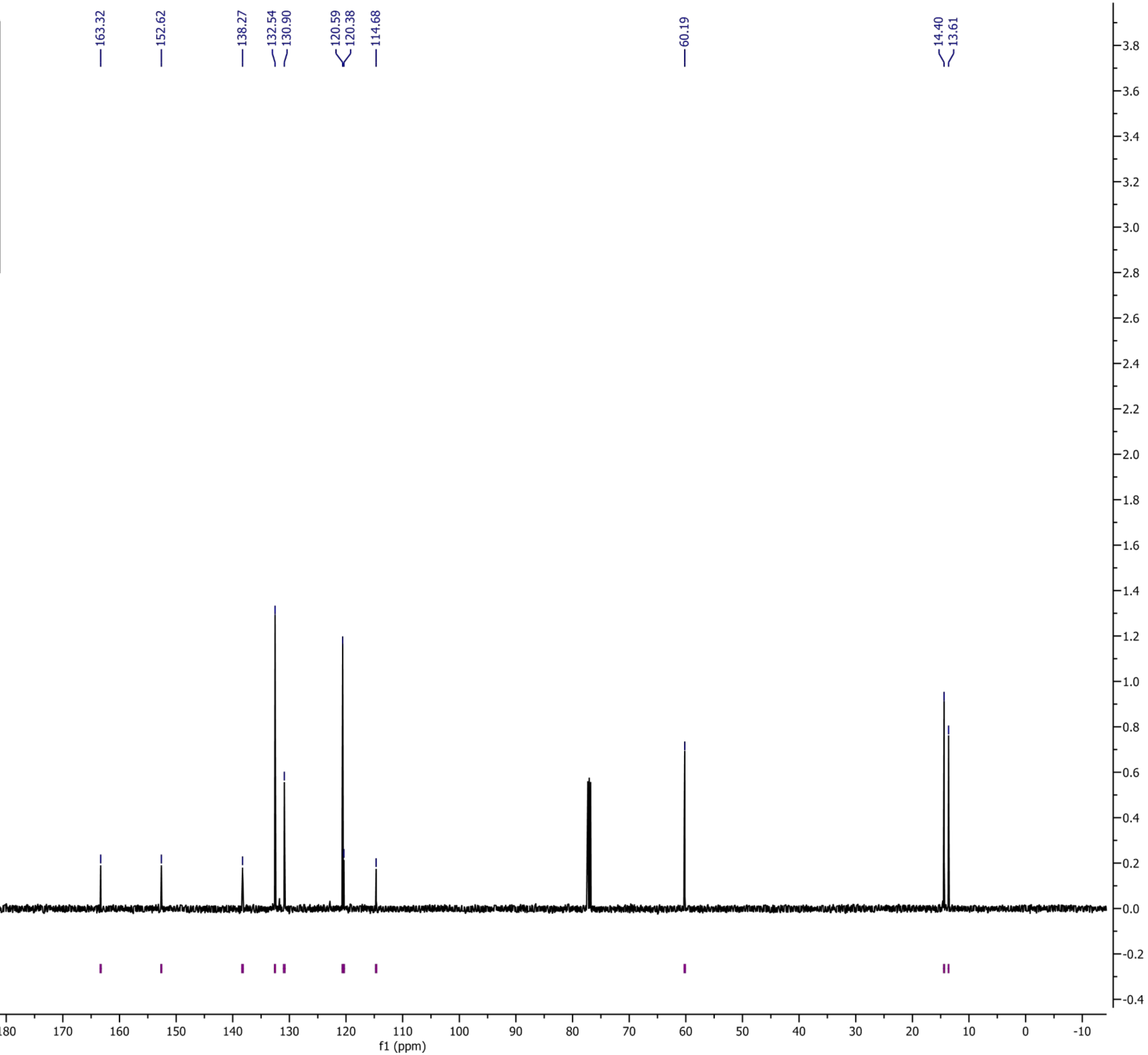

Figure S34 ^{13}C NMR spectrum of compound **13-2** ($CDCl_3$, 126 MHz)

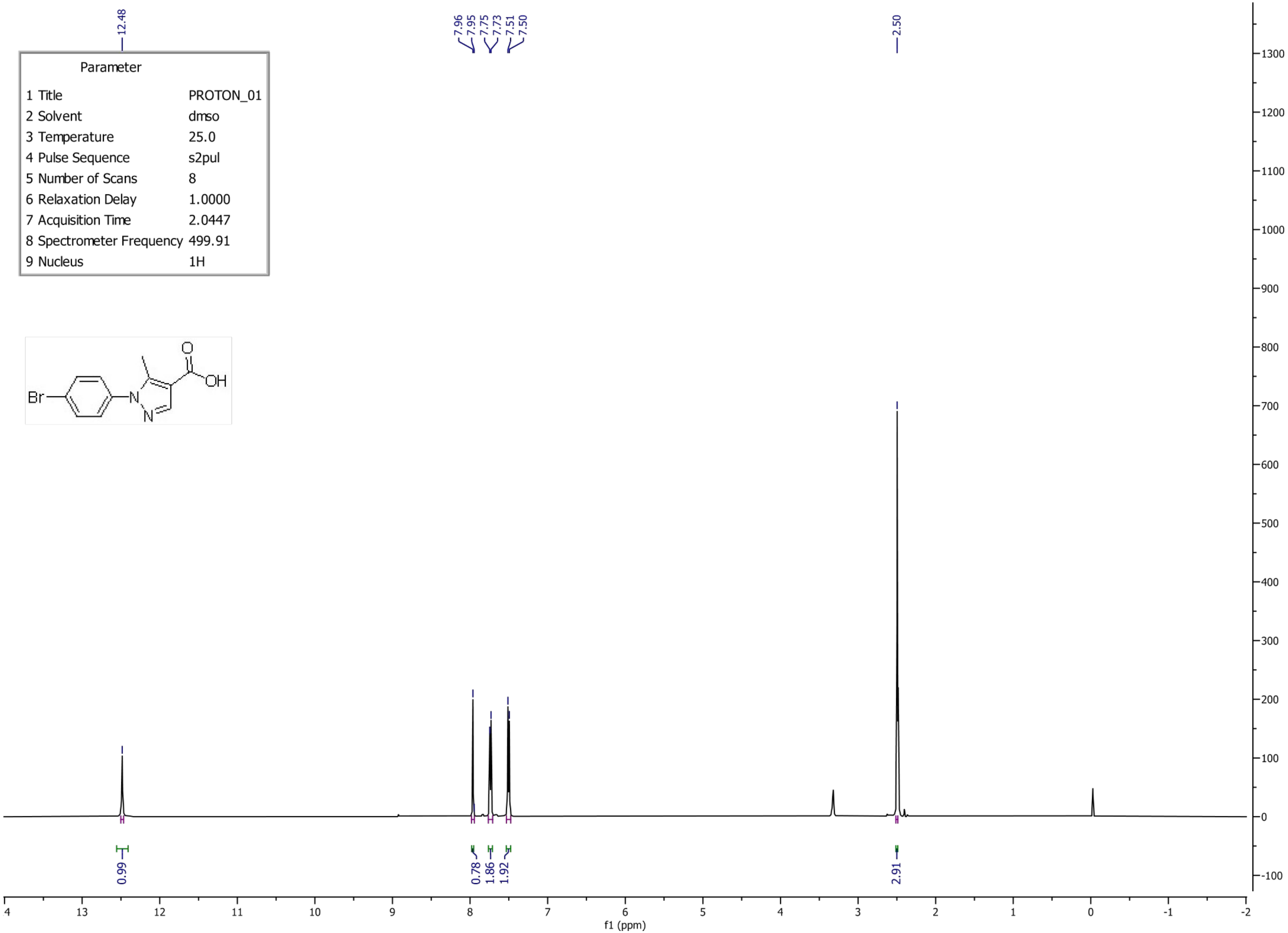

Figure S35 ^{1}H NMR spectrum of compound **14** (DMSO-d6,500 MHz)

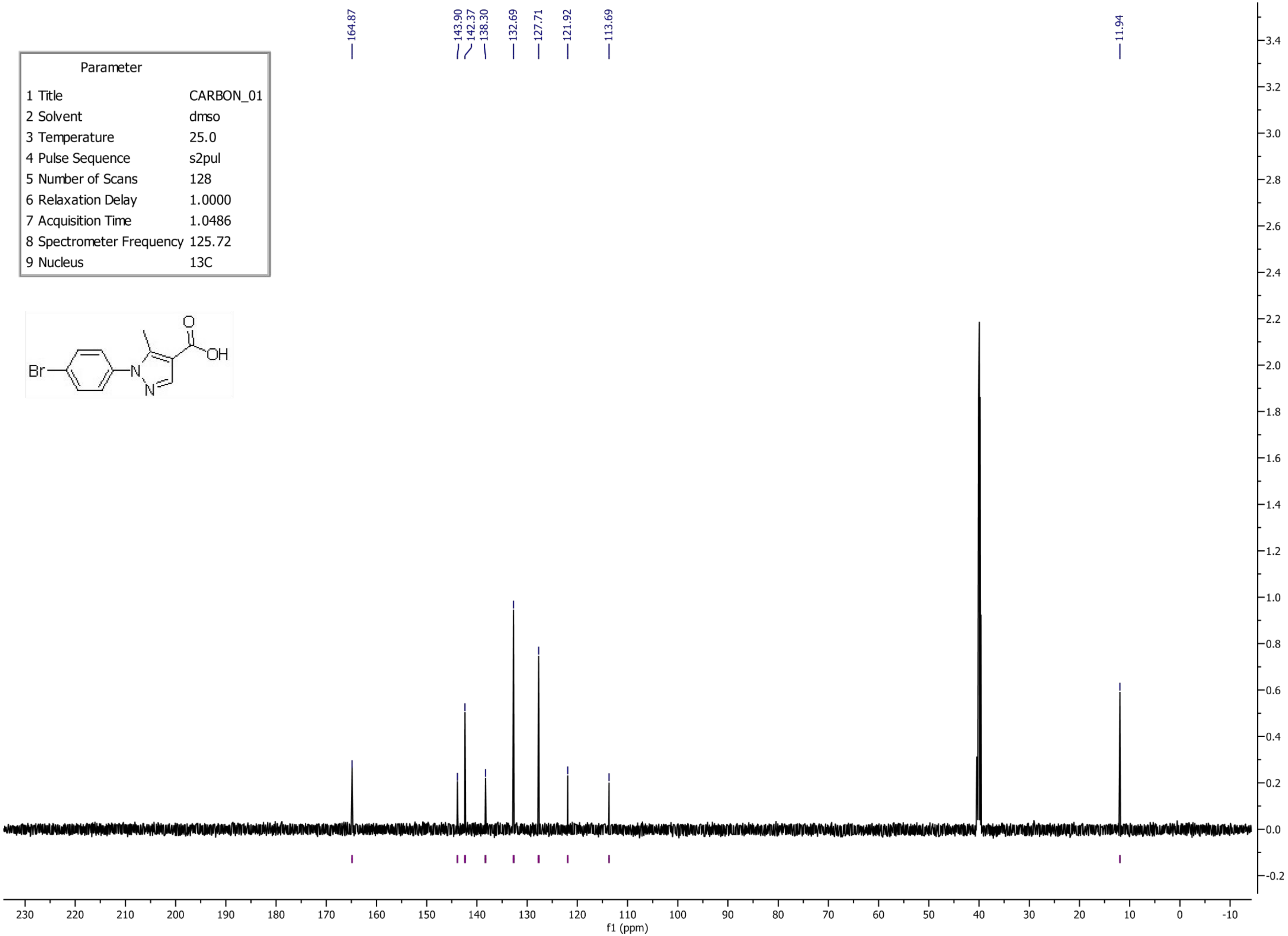

Figure S36 ^{13}C NMR spectrum of compound **14** (DMSO-d6, 126 MHz)

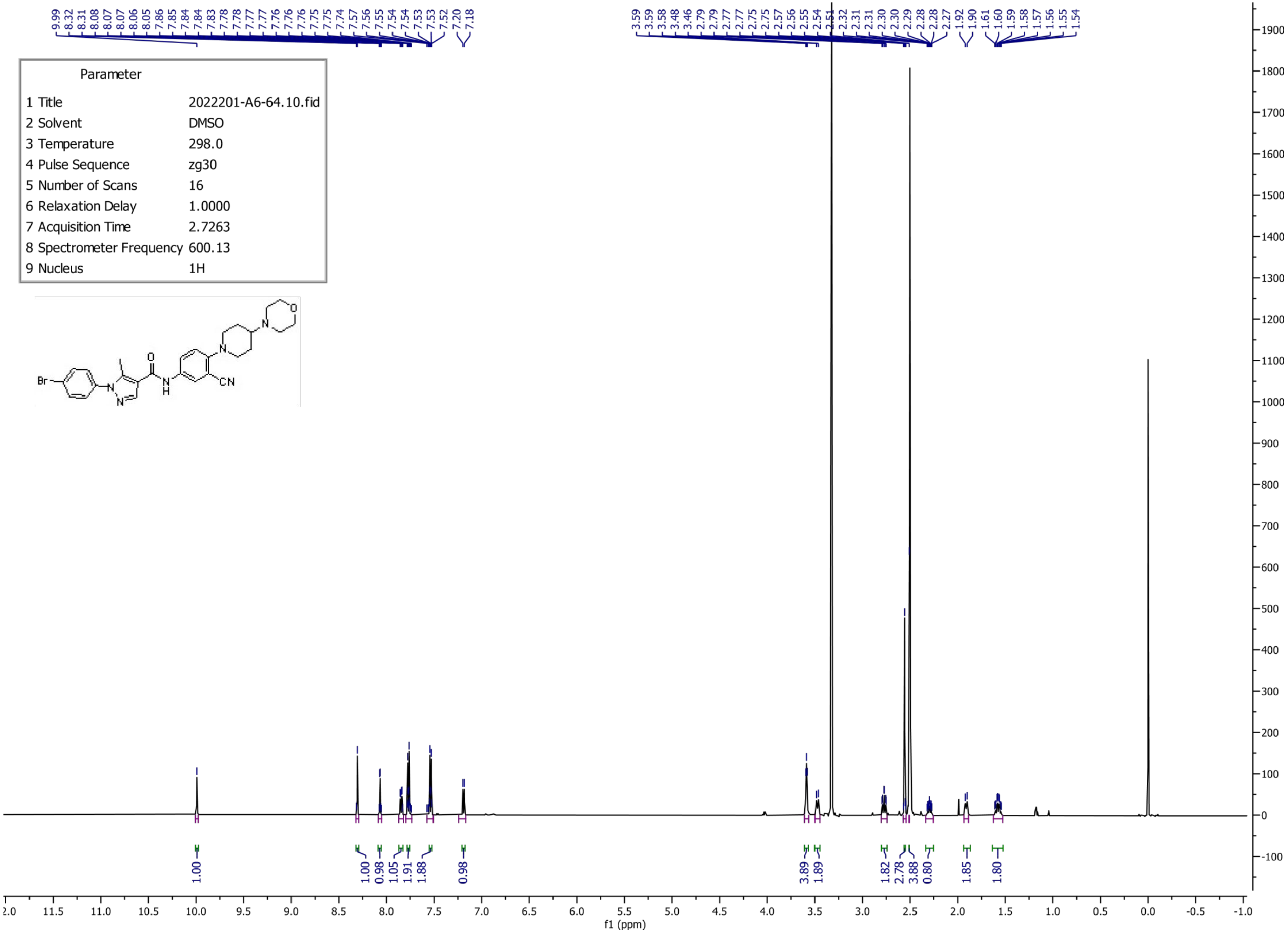

Figure S37 ^{1}H NMR spectrum of compound **15** (DMSO-d6, 600 MHz)

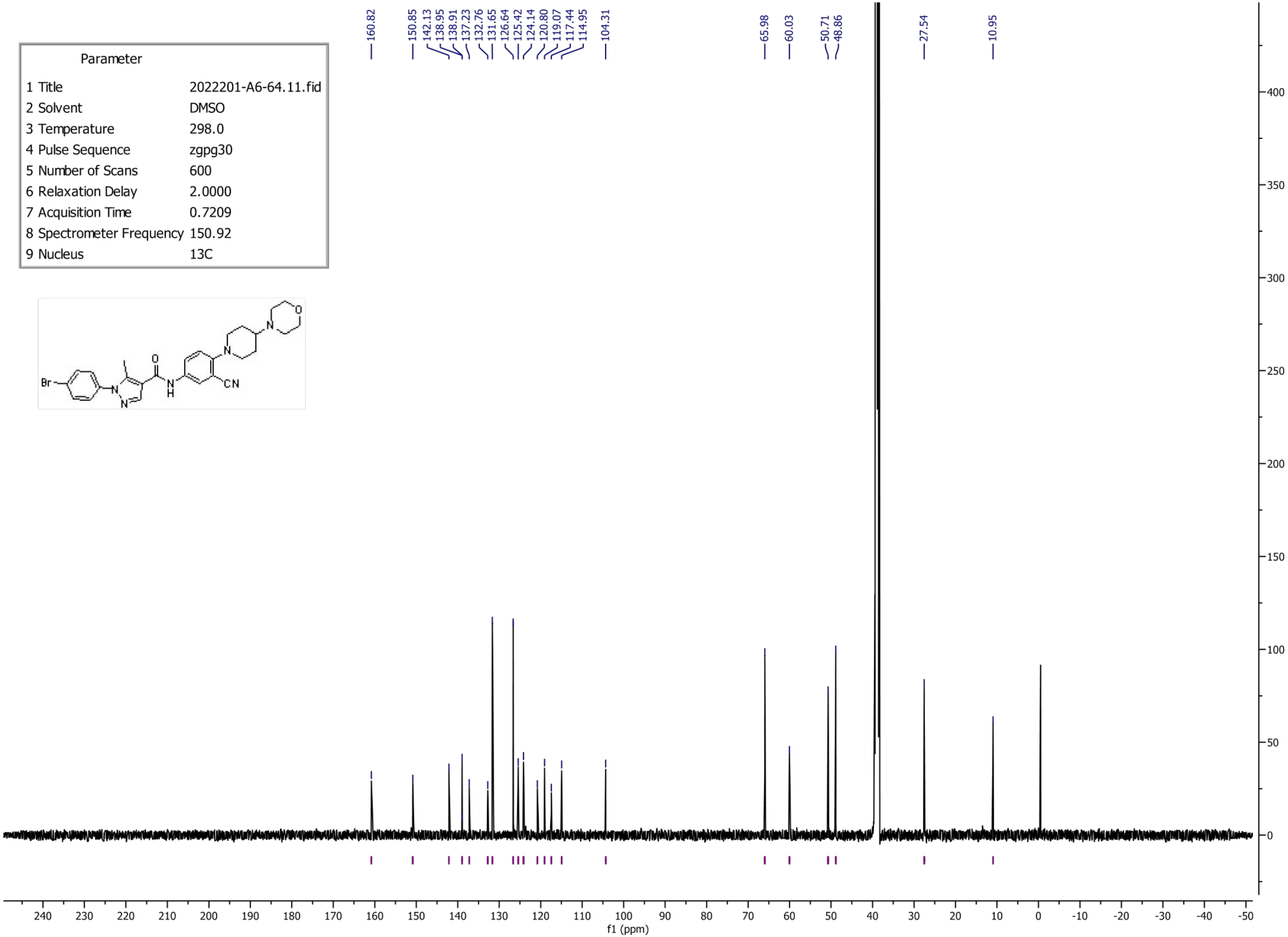

Figure S38 ^{13}C NMR spectrum of compound **15** (DMSO-d6, 126 MHz)

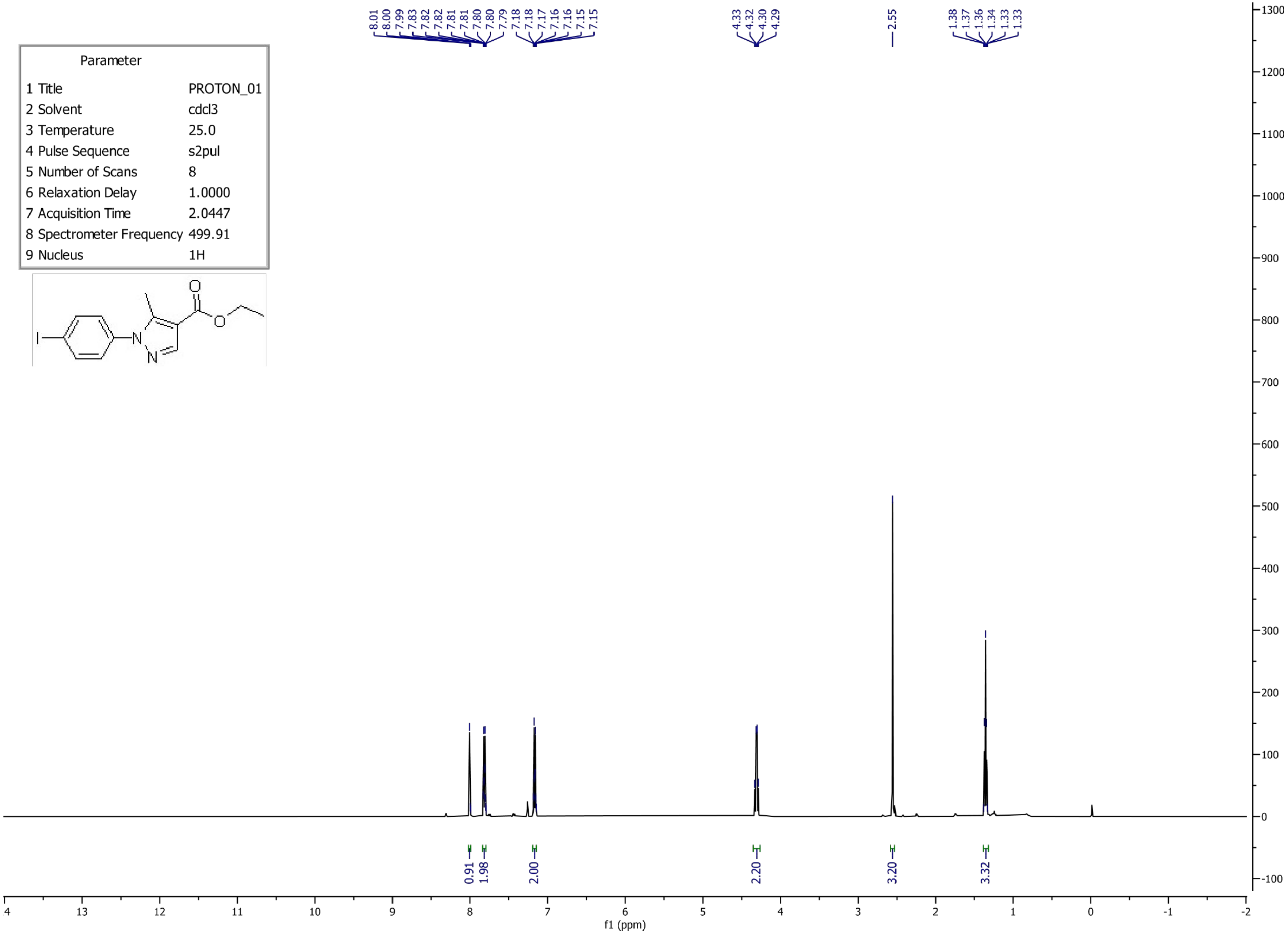

Figure S39 ^{1}H NMR spectrum of compound **16** ($CDCl_3$, 500 MHz)

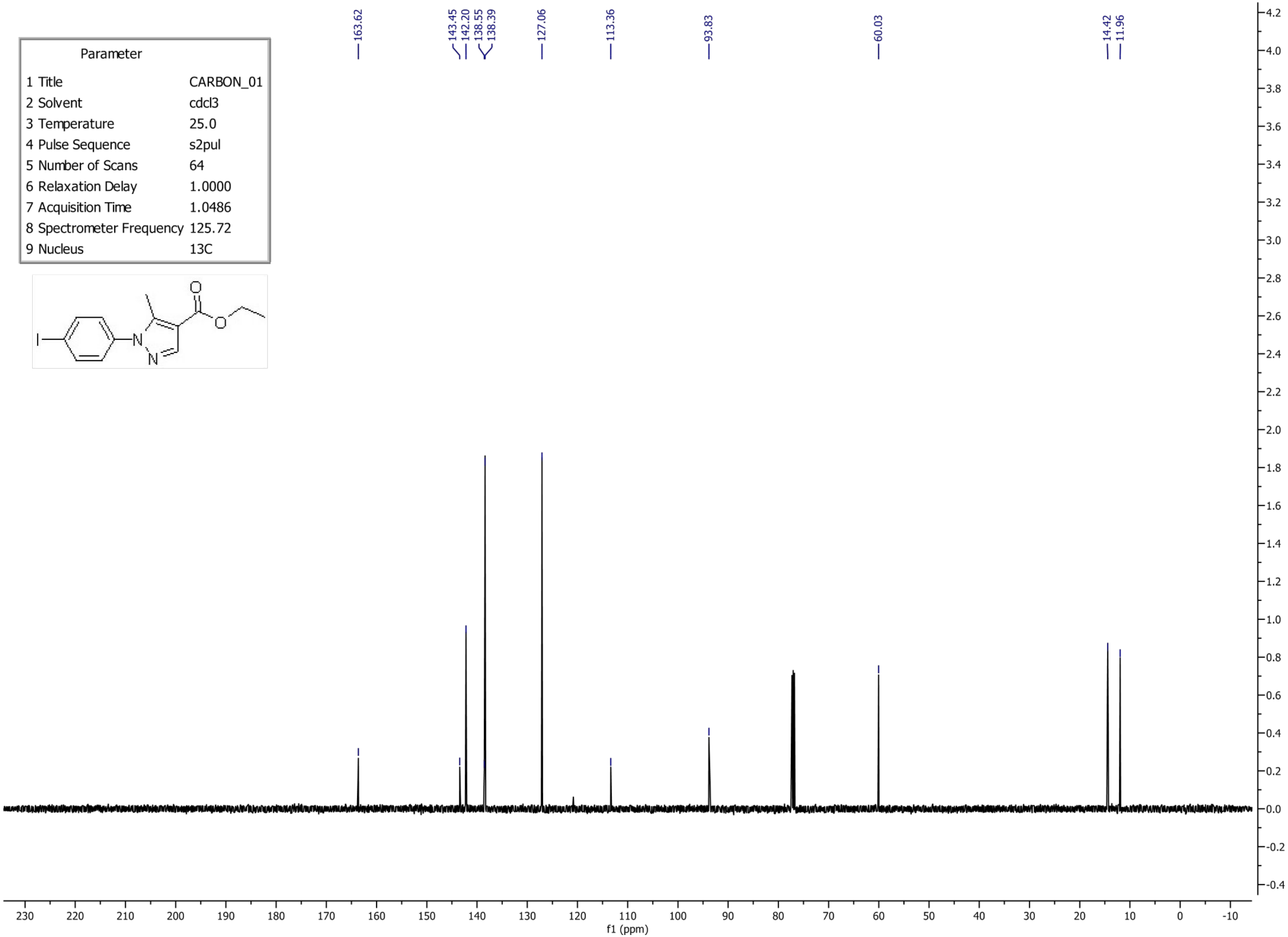

Figure S40 ^{13}C NMR spectrum of compound **16** ($CDCl_3$, 126 MHz)

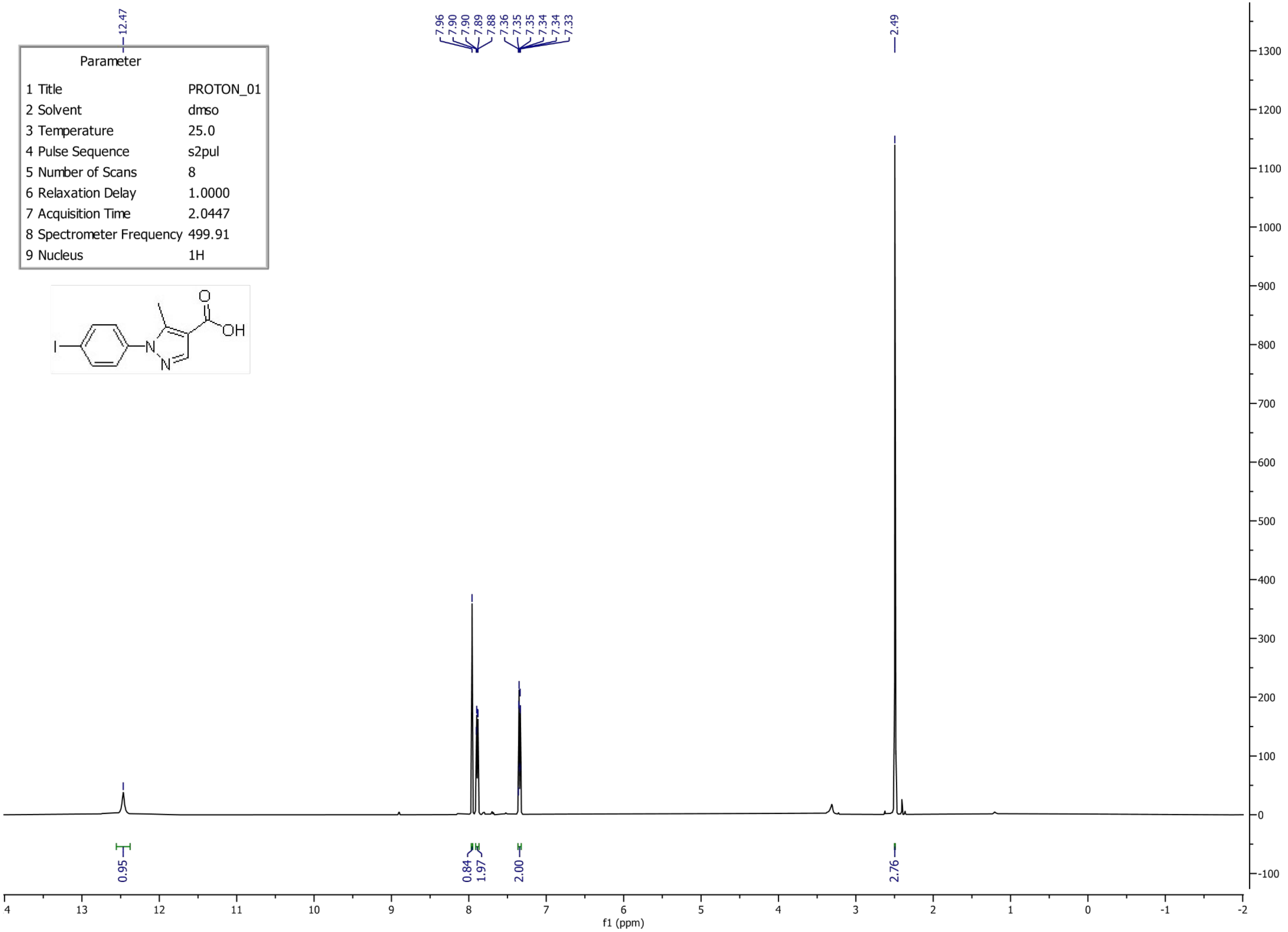

Figure S41 ¹H NMR spectrum of compound **17** (DMSO-d6, 500 MHz)

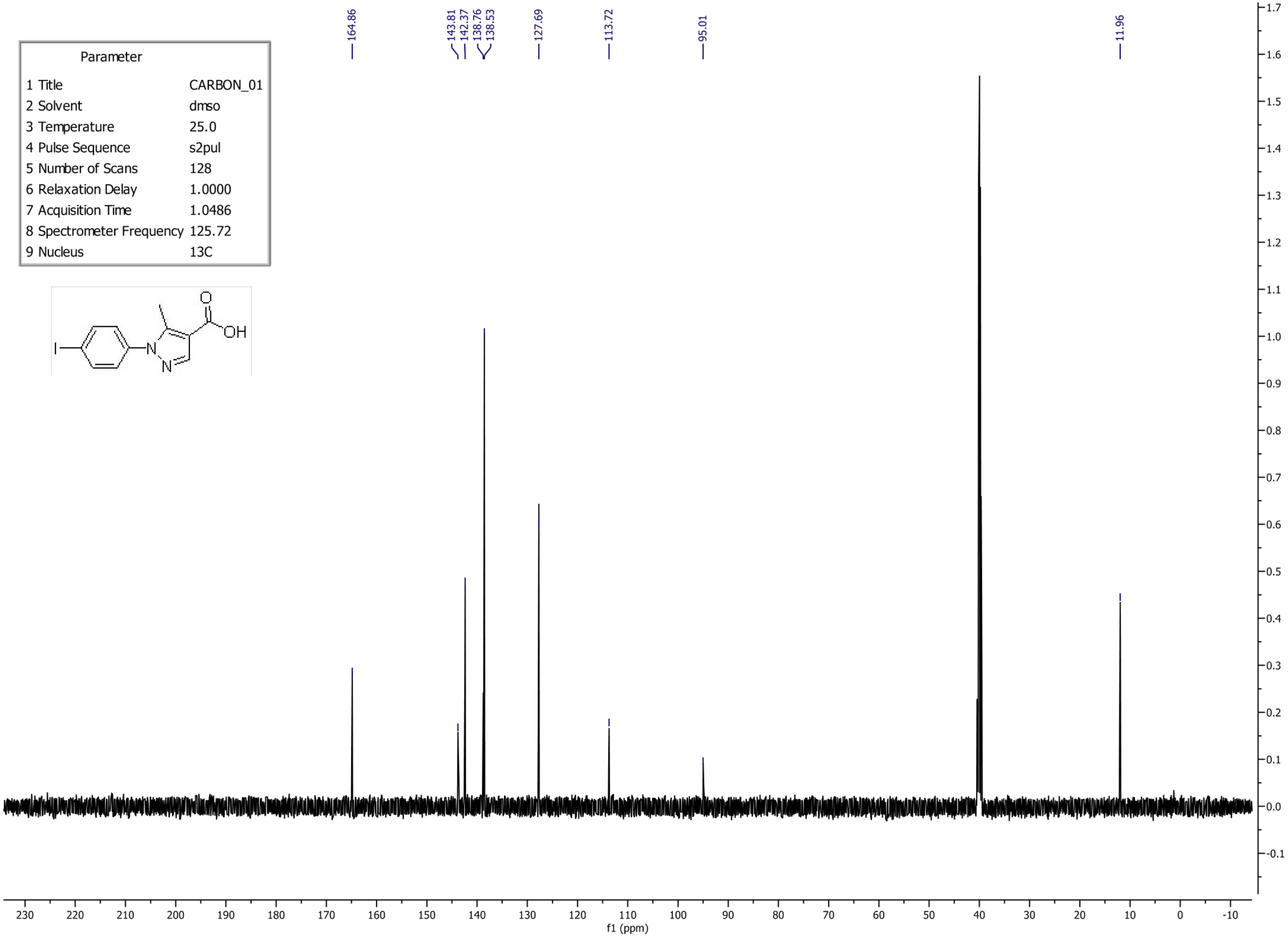

Figure S42 ^{13}C NMR spectrum of compound **17** (DMSO-d6, 126 MHz)

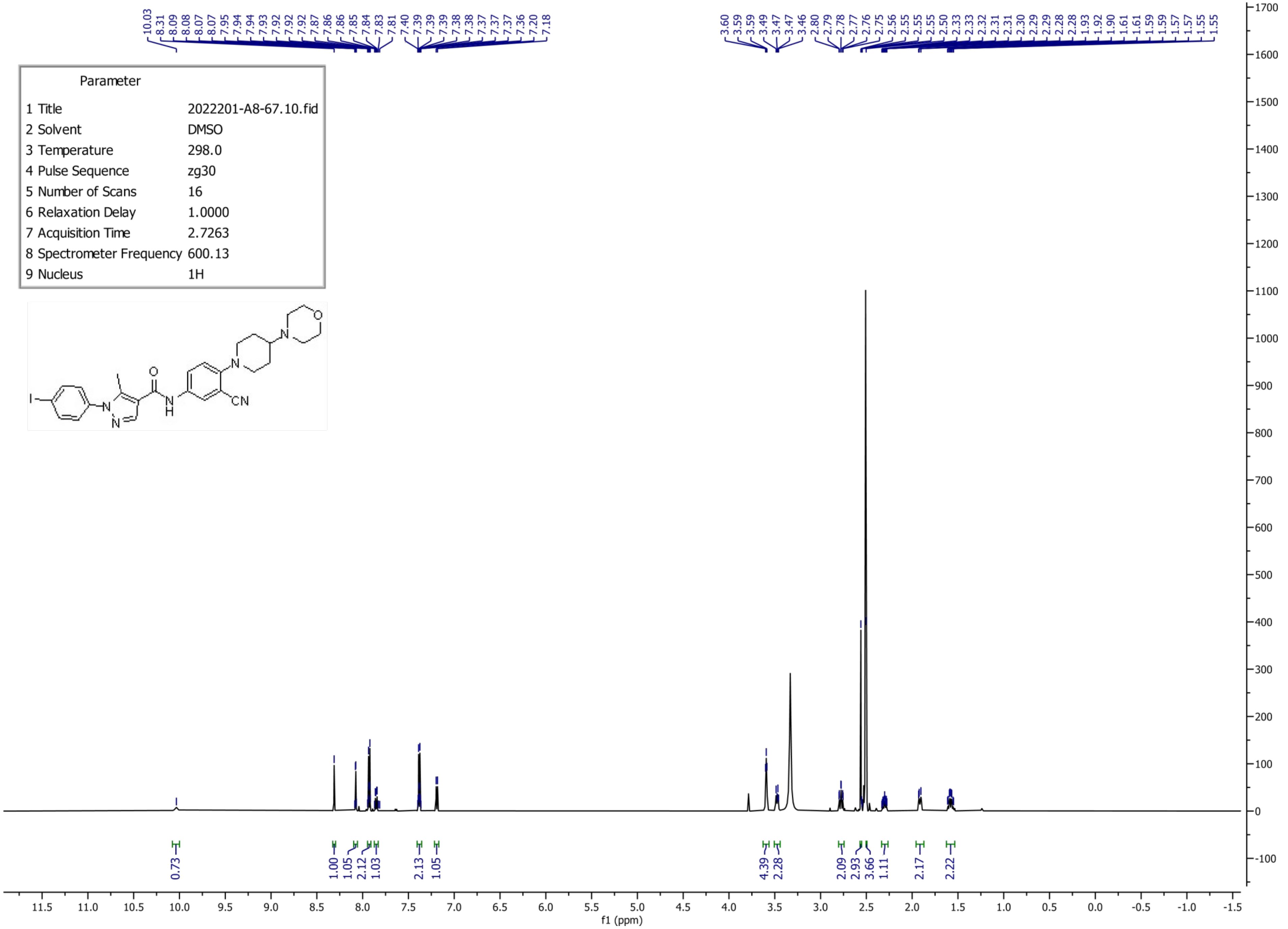

Figure S43 ¹H NMR spectrum of compound **18 (BY-014)** (DMSO-d6, 600 MHz)

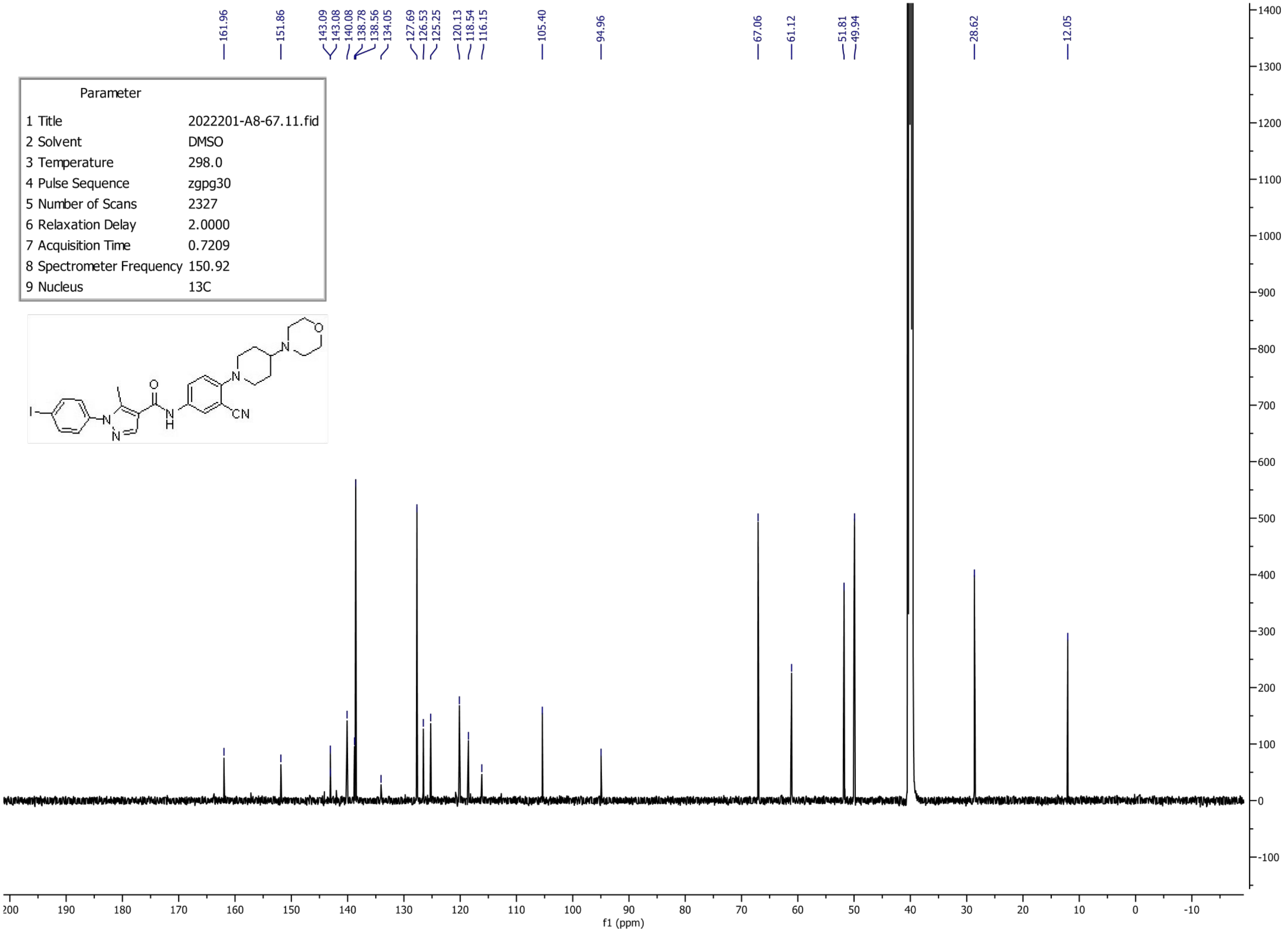

Parameter	
1 Title	2022201-A8-67.11.fid
2 Solvent	DMSO
3 Temperature	298.0
4 Pulse Sequence	zgpg30
5 Number of Scans	2327
6 Relaxation Delay	2.0000
7 Acquisition Time	0.7209
8 Spectrometer Frequency	150.92
9 Nucleus	13C

Figure S44 ^{13}C NMR spectrum of compound **18 (BY-014)** (DMSO-d6, 151 MHz)

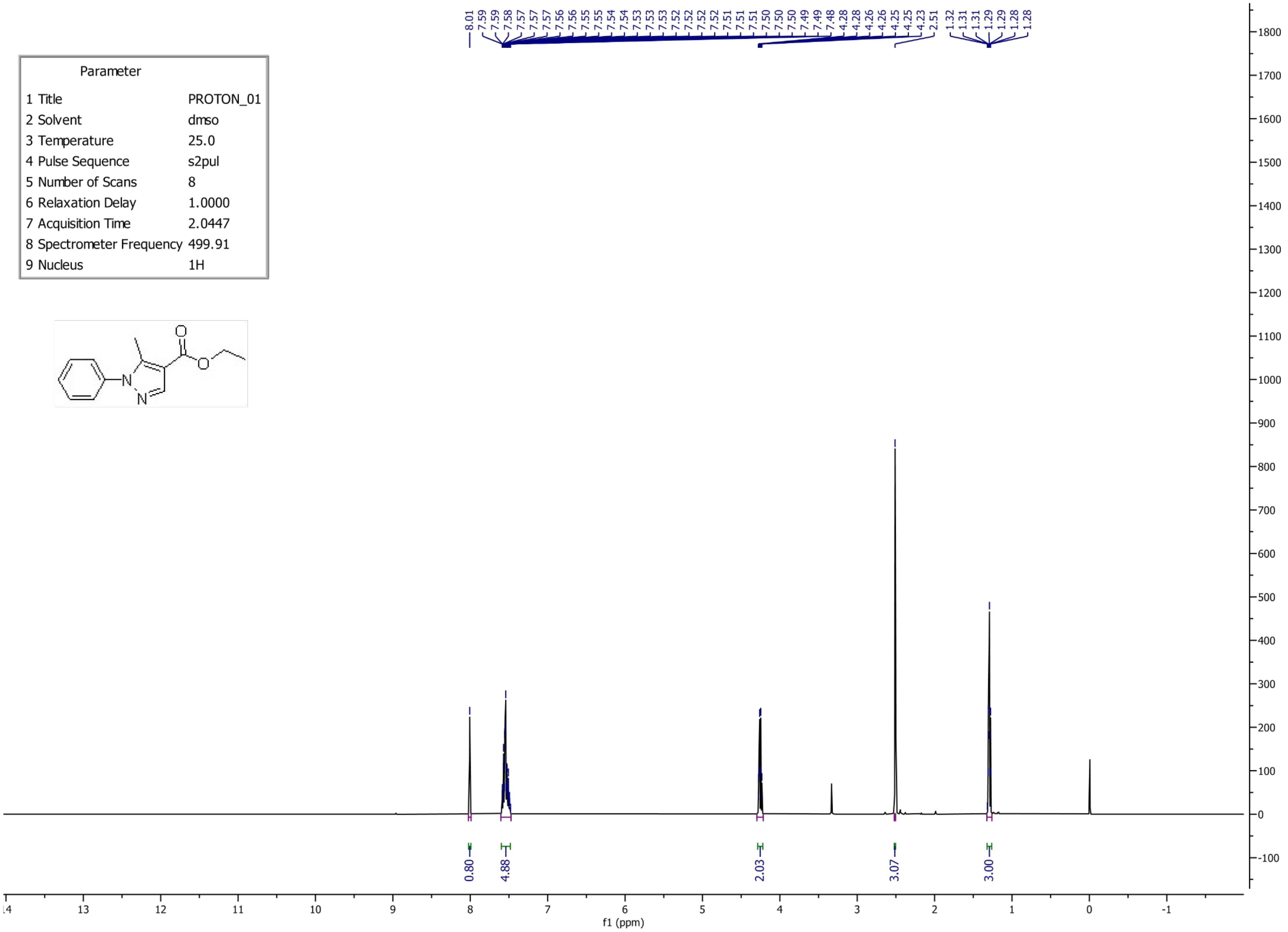

Figure S45 ^{1}H NMR spectrum of compound **19-1** (DMSO-d6, 500 MHz)

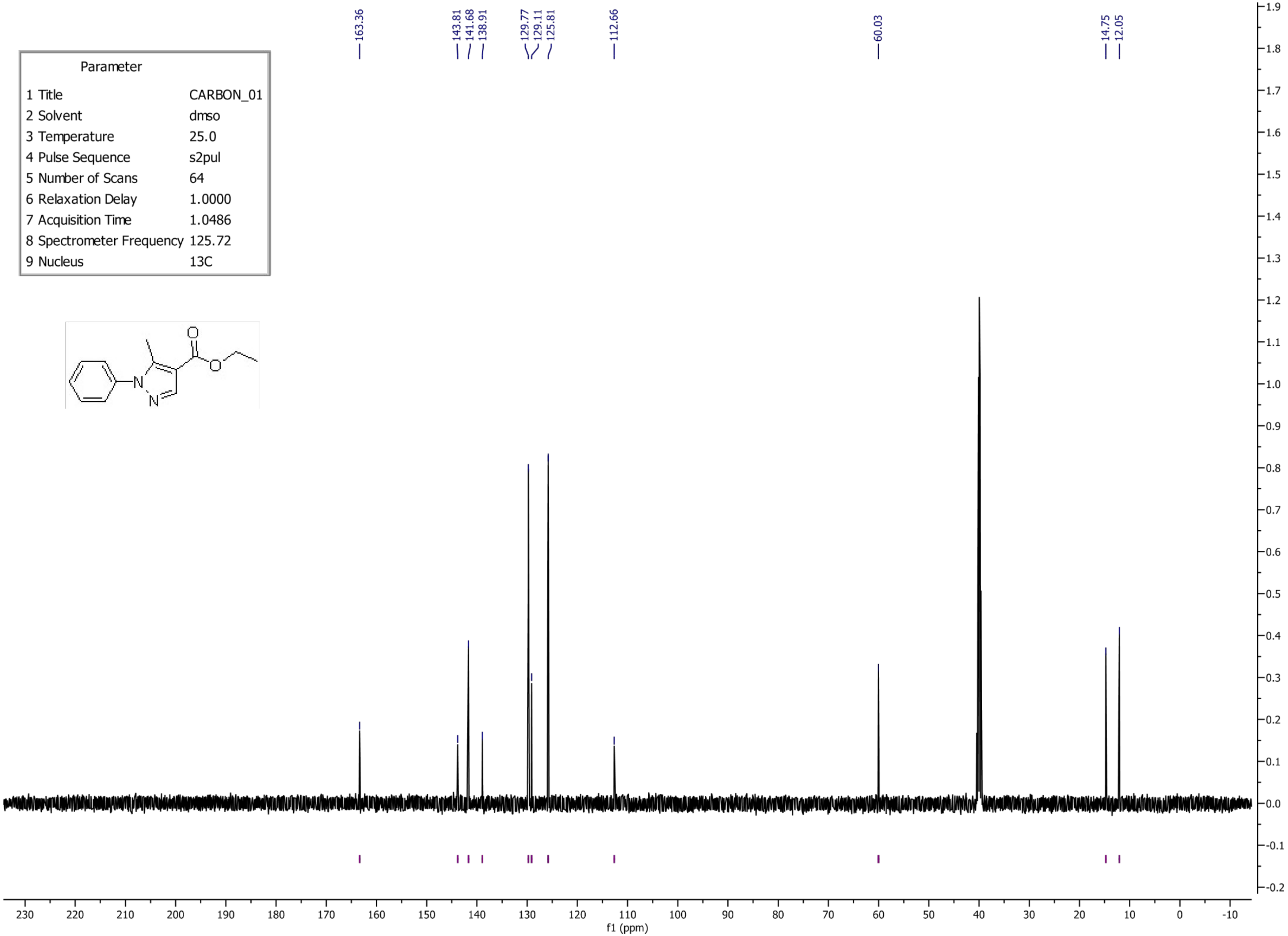

Figure S46 ^{13}C NMR spectrum of compound **19-1** (DMSO-d6, 126 MHz)

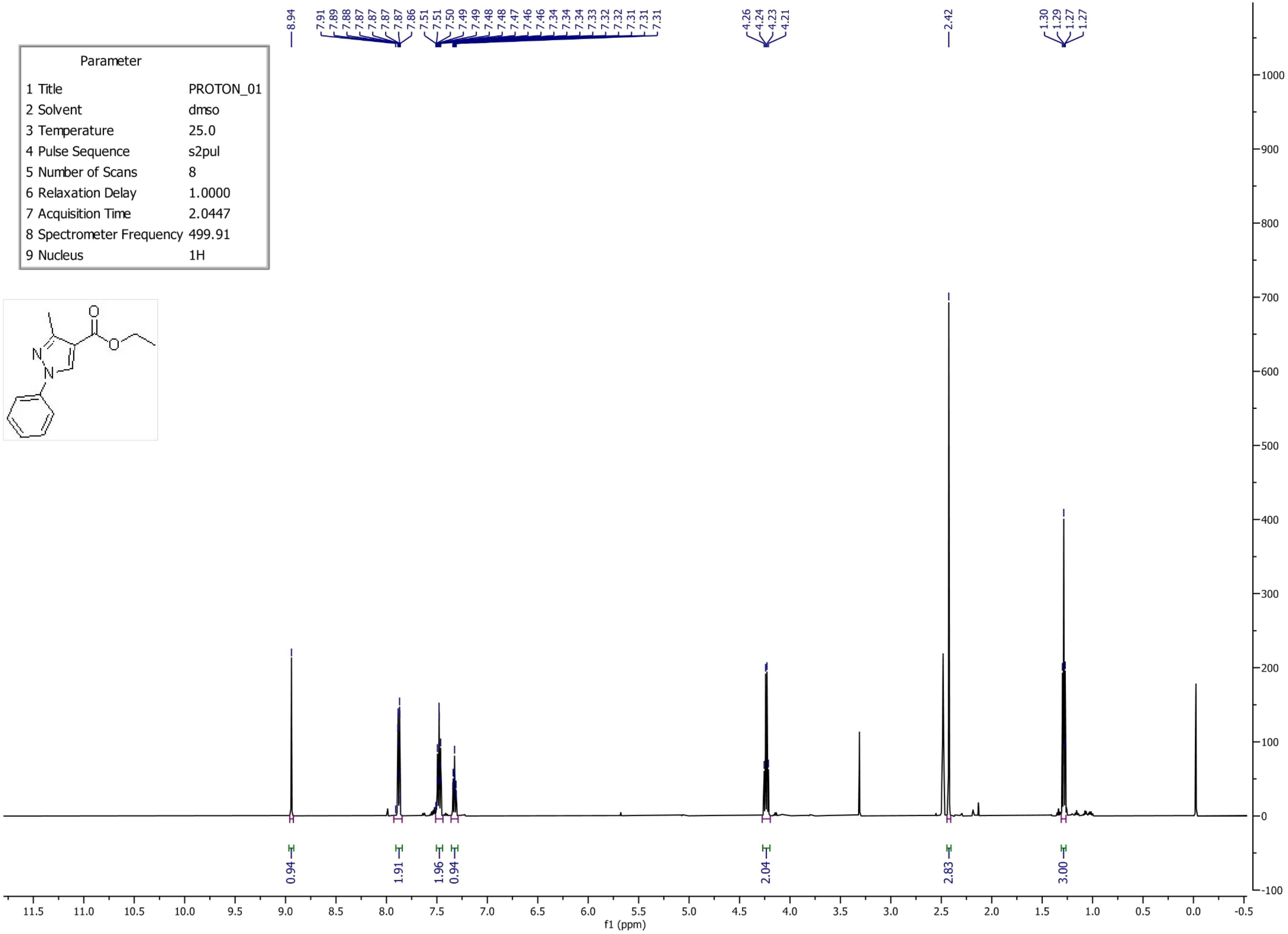

Figure S47 ^{1}H NMR spectrum of compound **19-2** (DMSO-d6, 600 MHz)

Parameter	
1 Title	CARBON_01
2 Solvent	dmso
3 Temperature	25.0
4 Pulse Sequence	s2pul
5 Number of Scans	192
6 Relaxation Delay	1.0000
7 Acquisition Time	1.0486
8 Spectrometer Frequency	125.72
9 Nucleus	13C

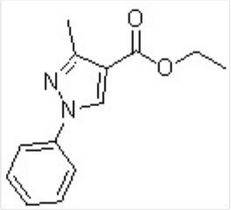

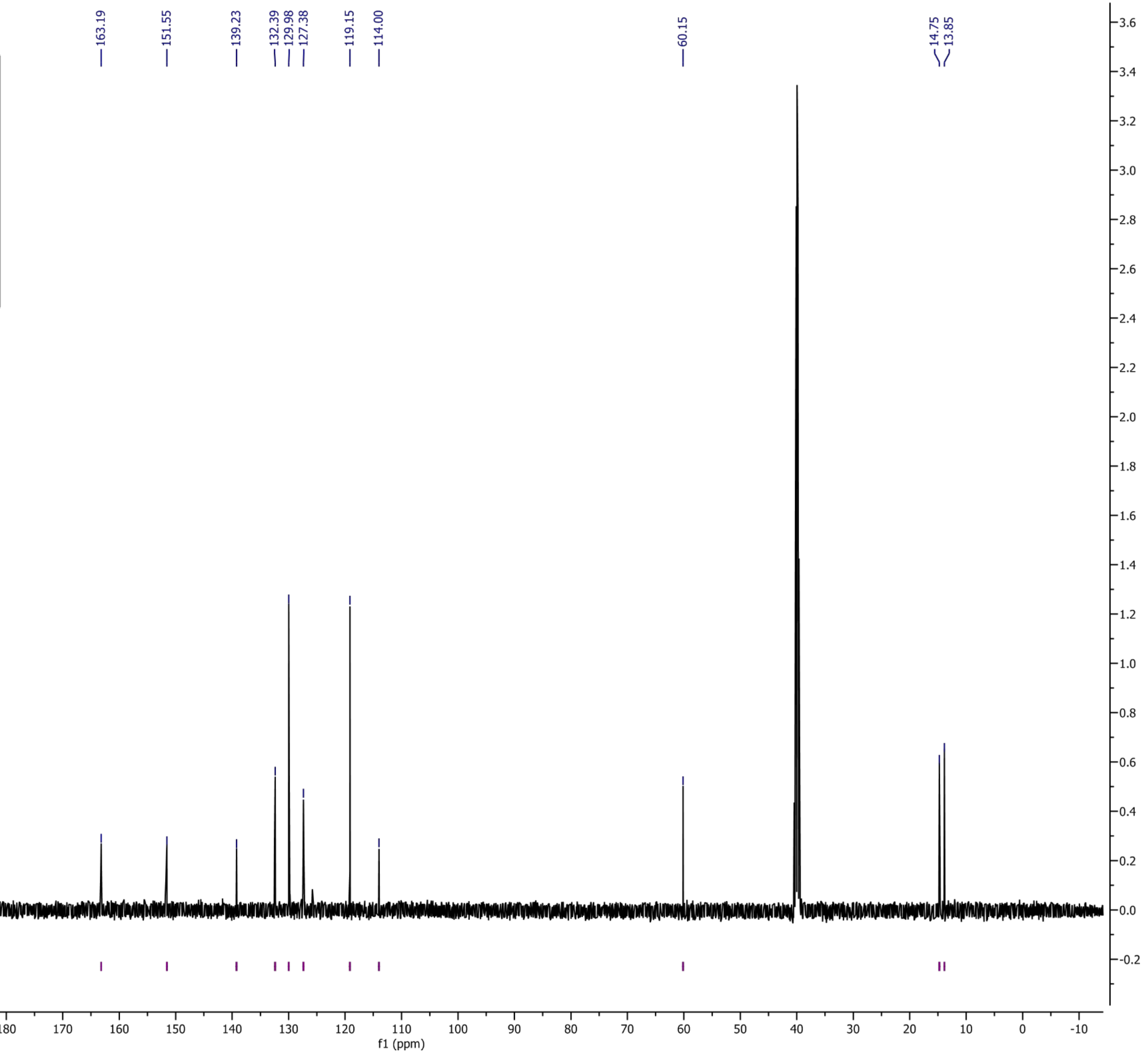

Figure S48 ^{13}C NMR spectrum of compound **19-2** (DMSO-d6, 151 MHz)

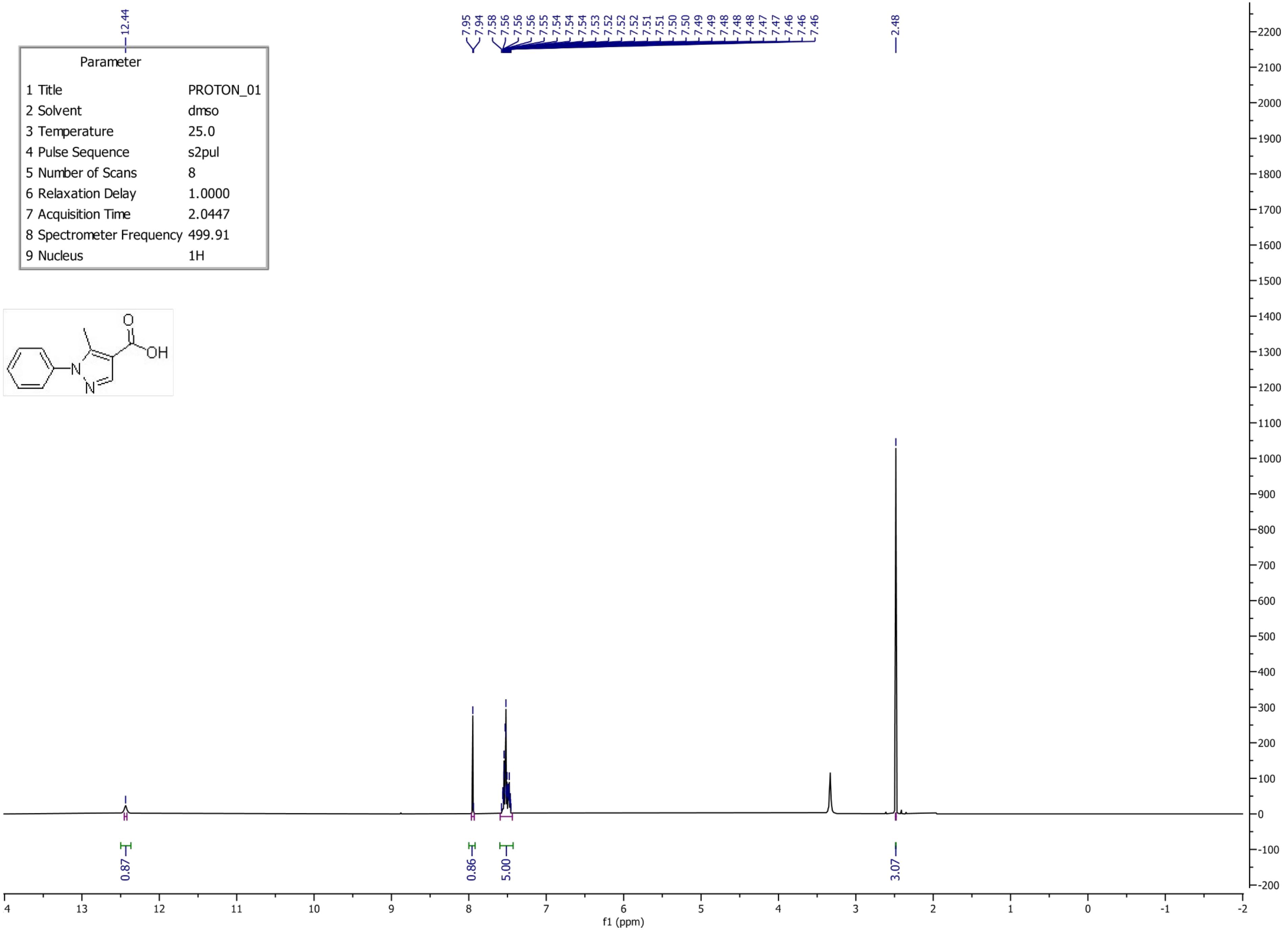

Figure S49 ^{1}H NMR spectrum of compound **20** (DMSO-d6, 500 MHz)

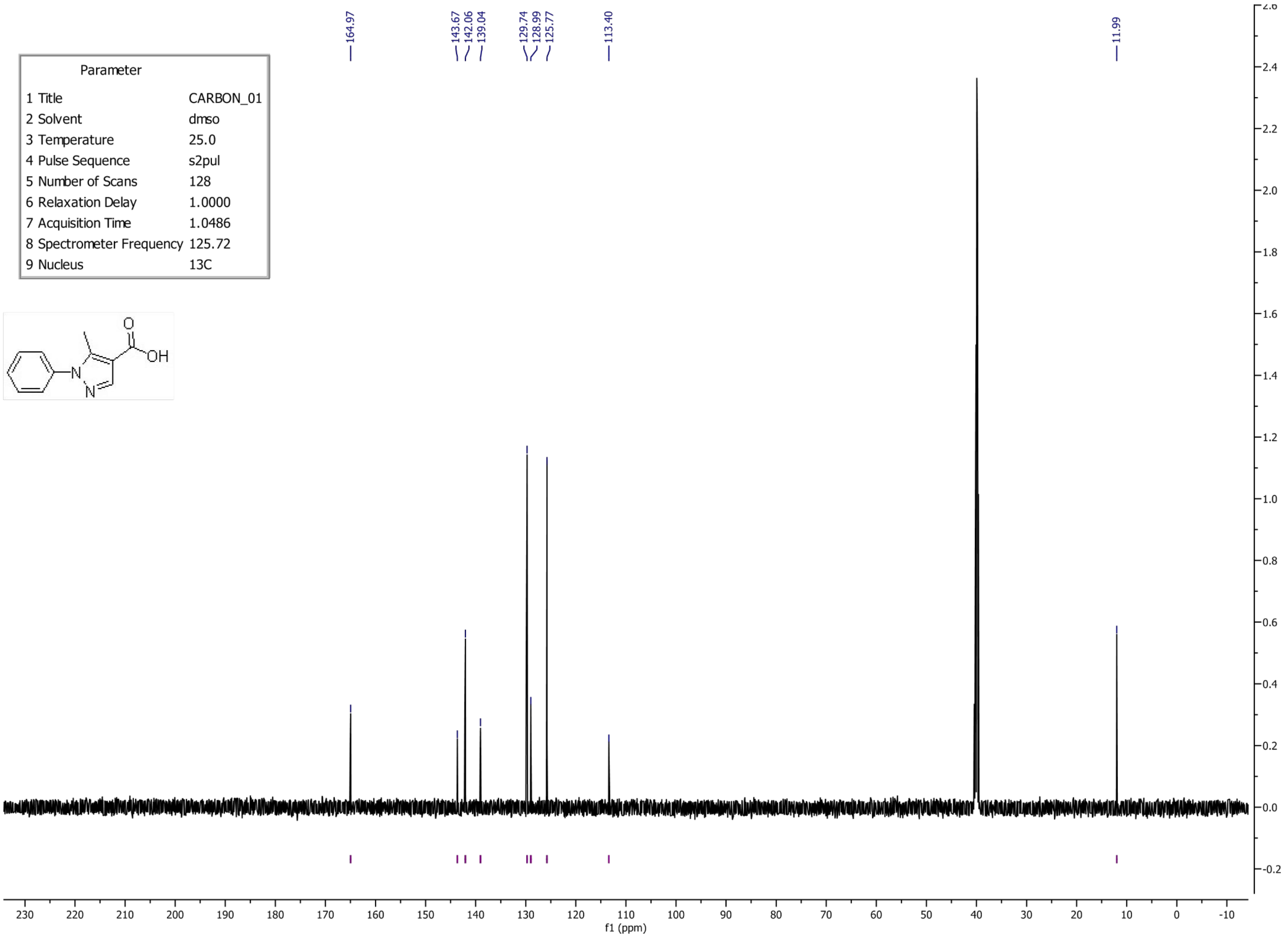

Figure S50 ^{13}C NMR spectrum of compound **20** (DMSO-d6, 126 MHz)

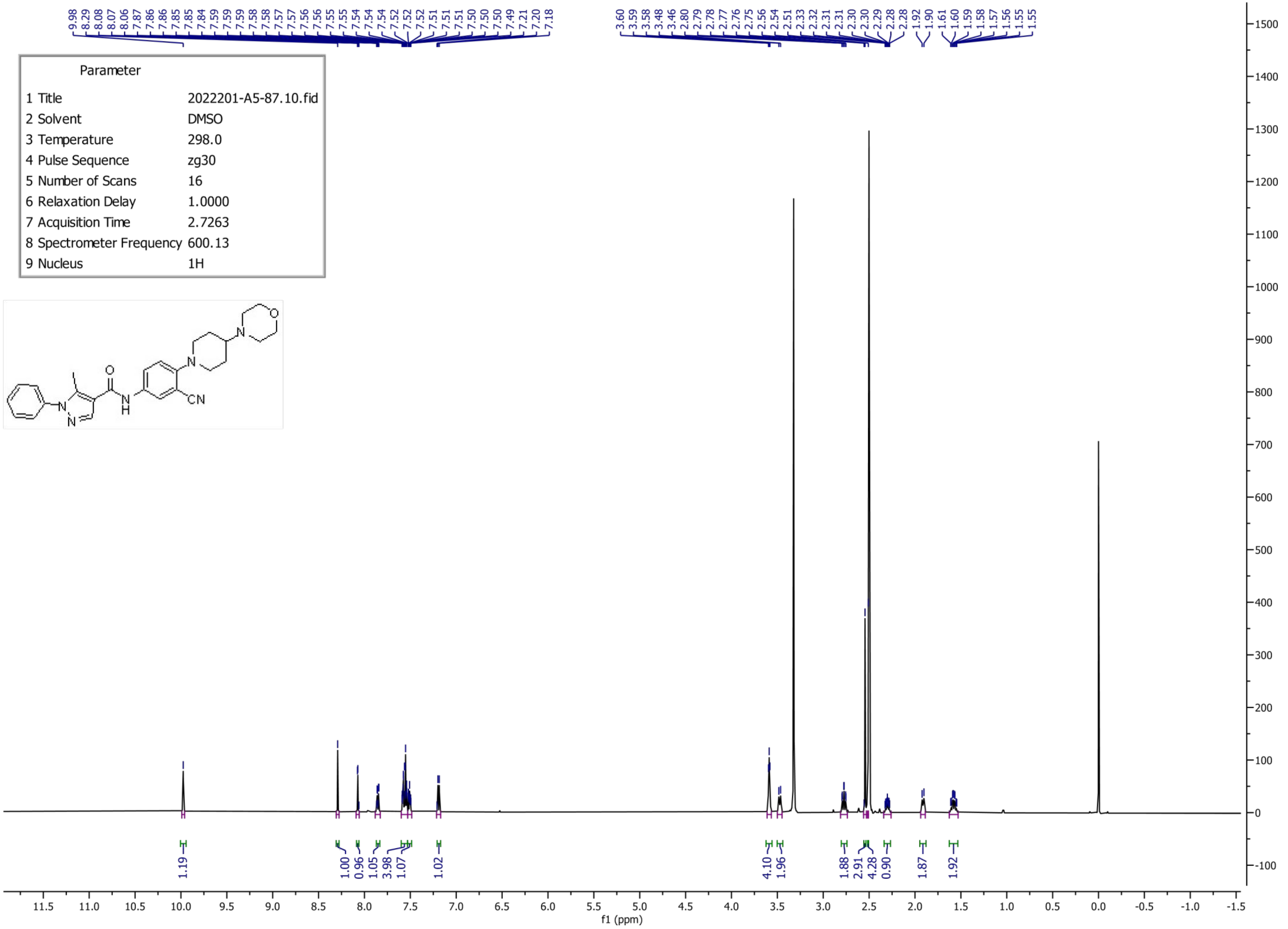

Figure S51 ^{1}H NMR spectrum of compound **21 (BY-004)** (DMSO-d6, 600 MHz)

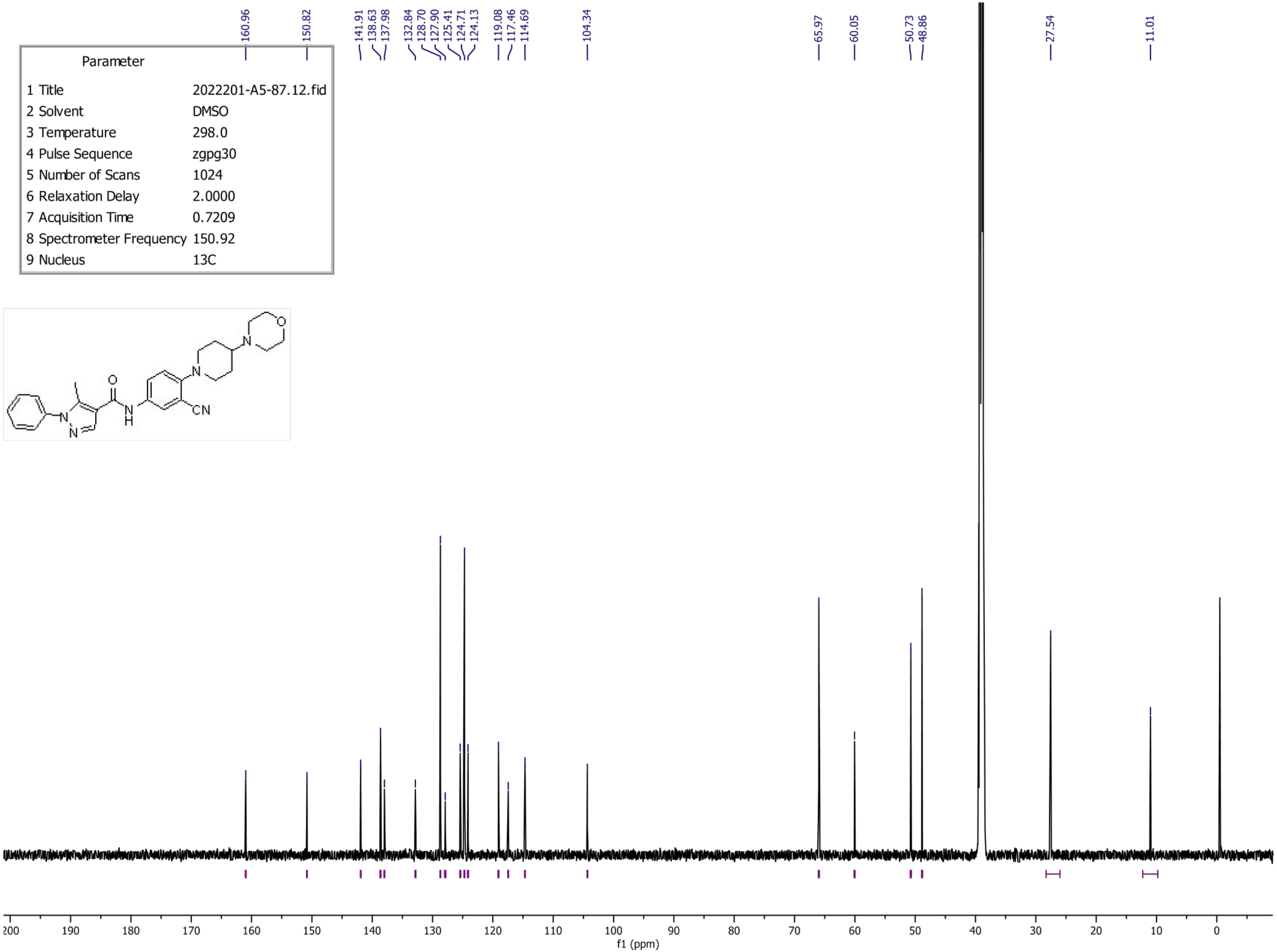

Figure S52 ^{13}C NMR spectrum of compound **21 (BY-004)** (DMSO-d6, 151 MHz)

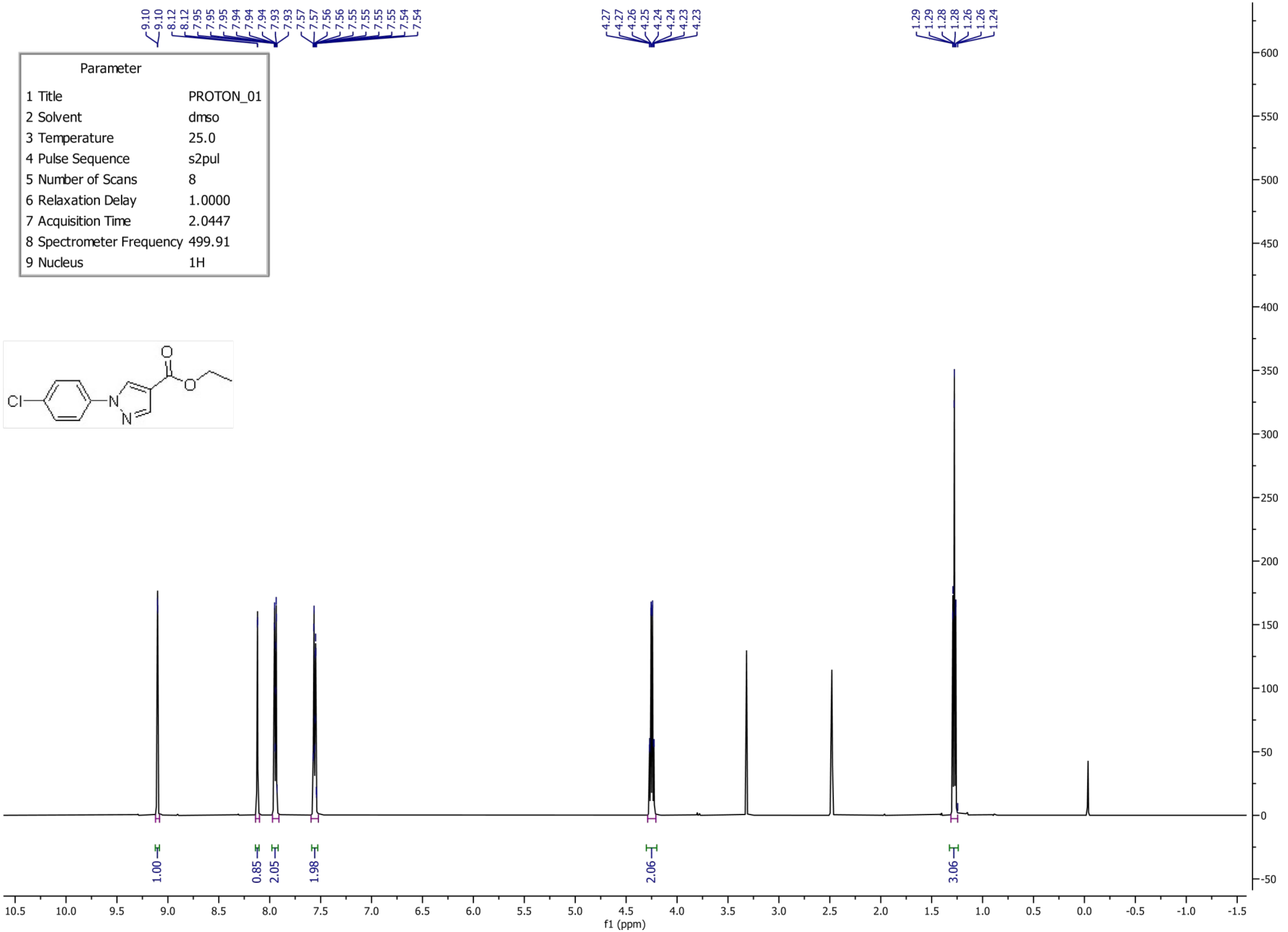

Figure S53 ^{1}H NMR spectrum of compound **22** (DMSO-d6, 500 MHz)

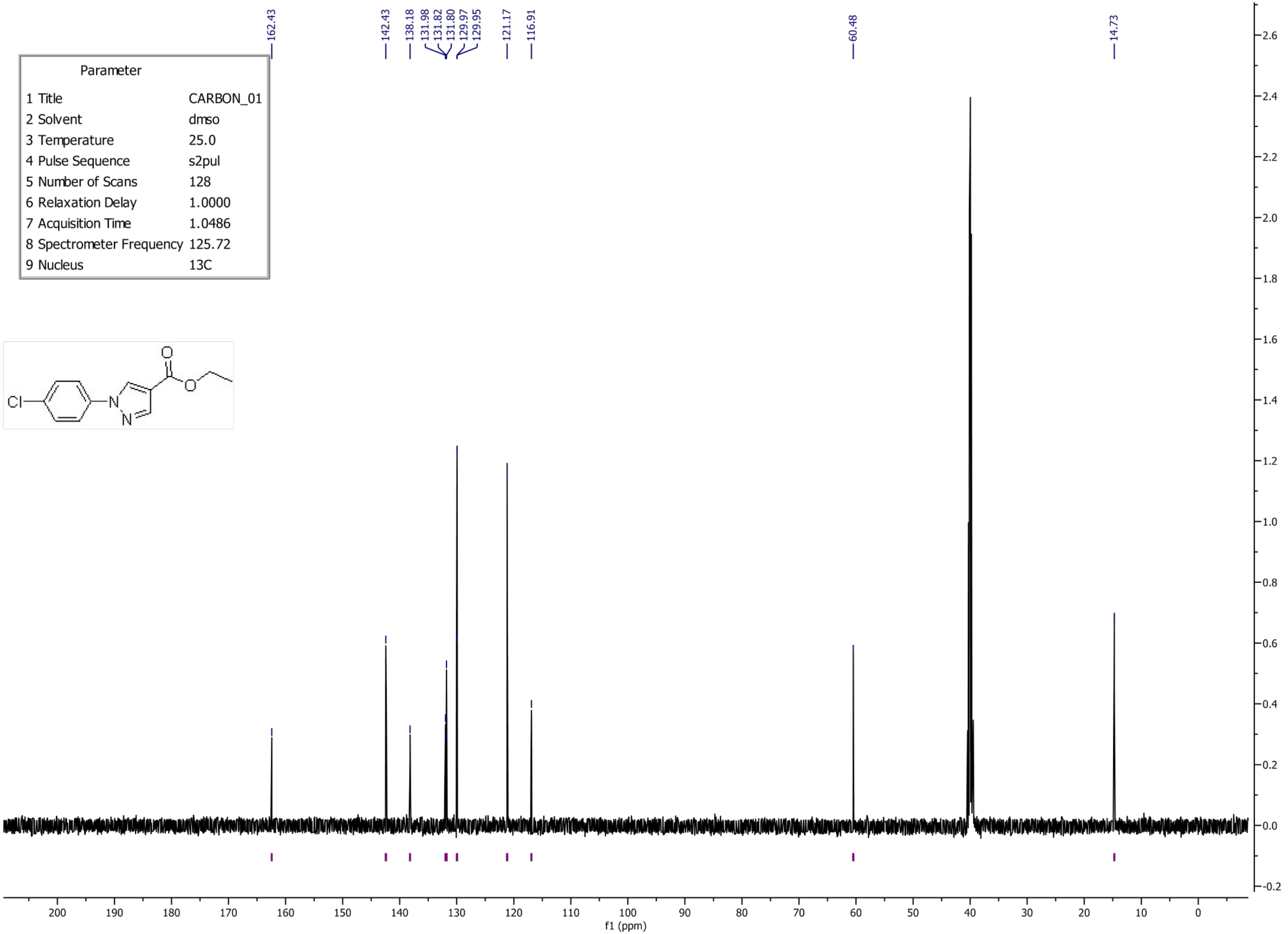

Figure S54 ^{13}C NMR spectrum of compound **22** (DMSO-d6, 126 MHz)

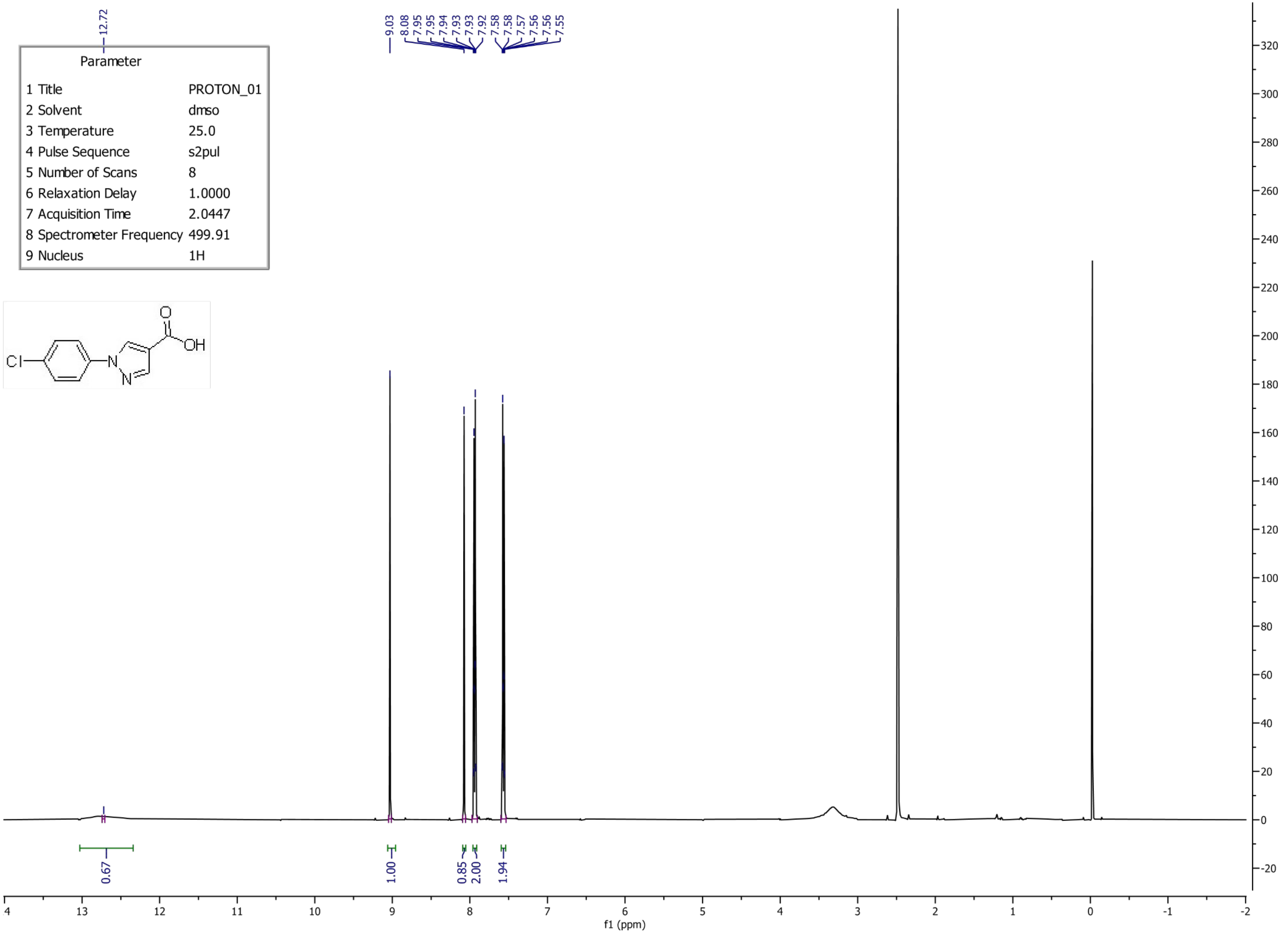

Figure S55 ^{1}H NMR spectrum of compound **23** (DMSO-d6, 500 MHz)

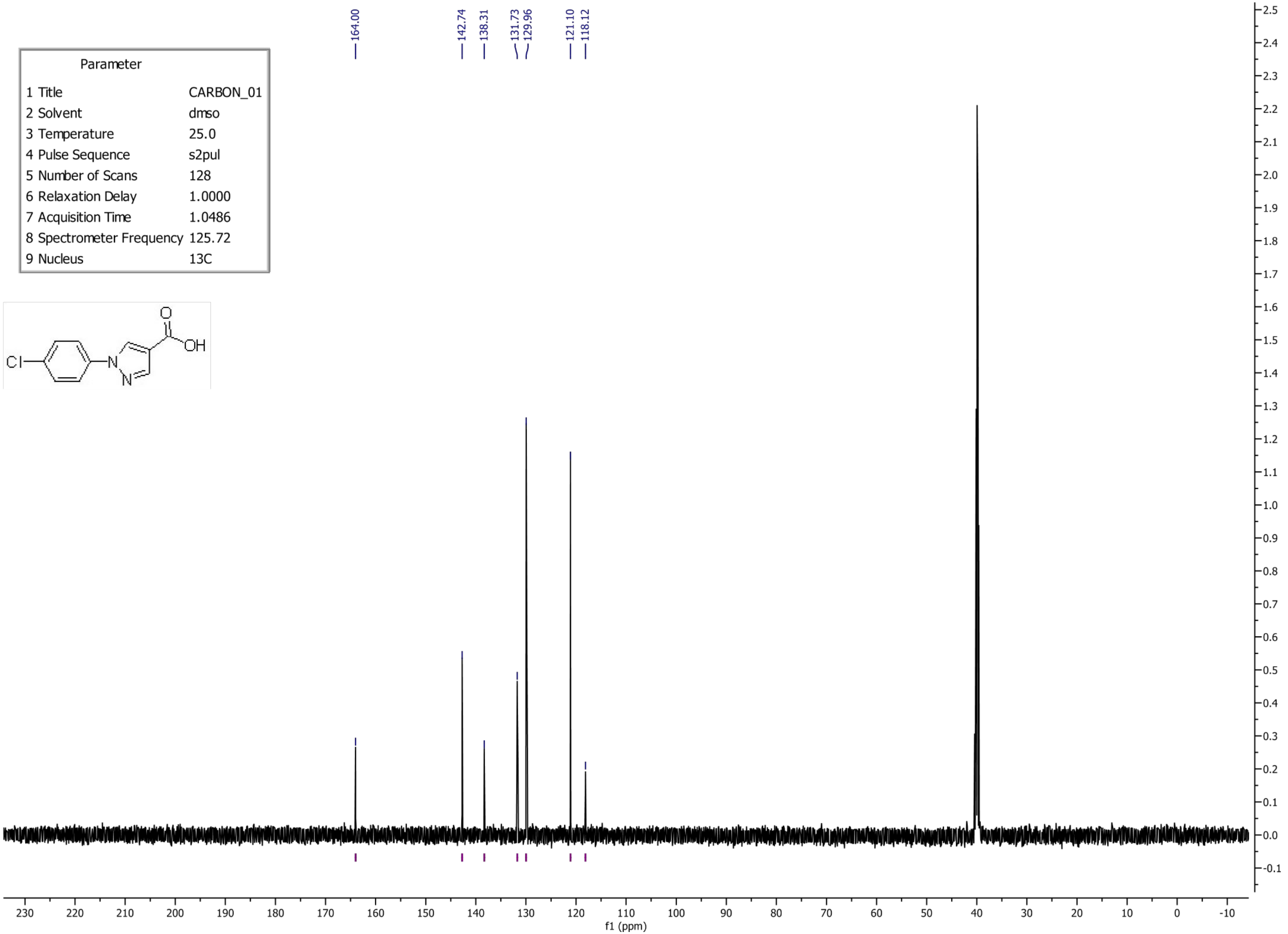

Figure S56 ^{13}C NMR spectrum of compound **23** (DMSO-d6, 126 MHz)

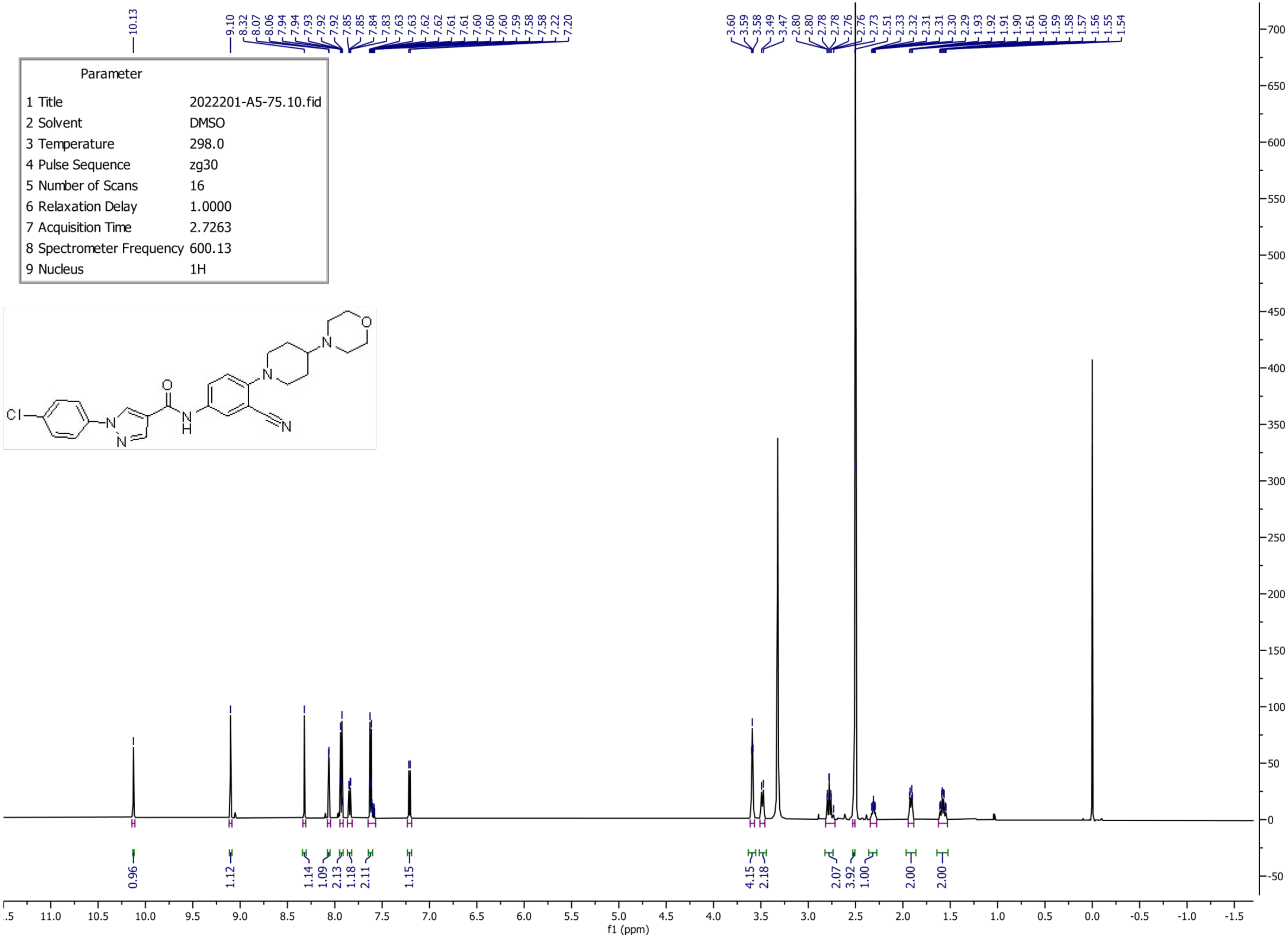

Figure S55 ^{1}H NMR spectrum of compound **24 (BY-001)** (DMSO-d6, 600 MHz)

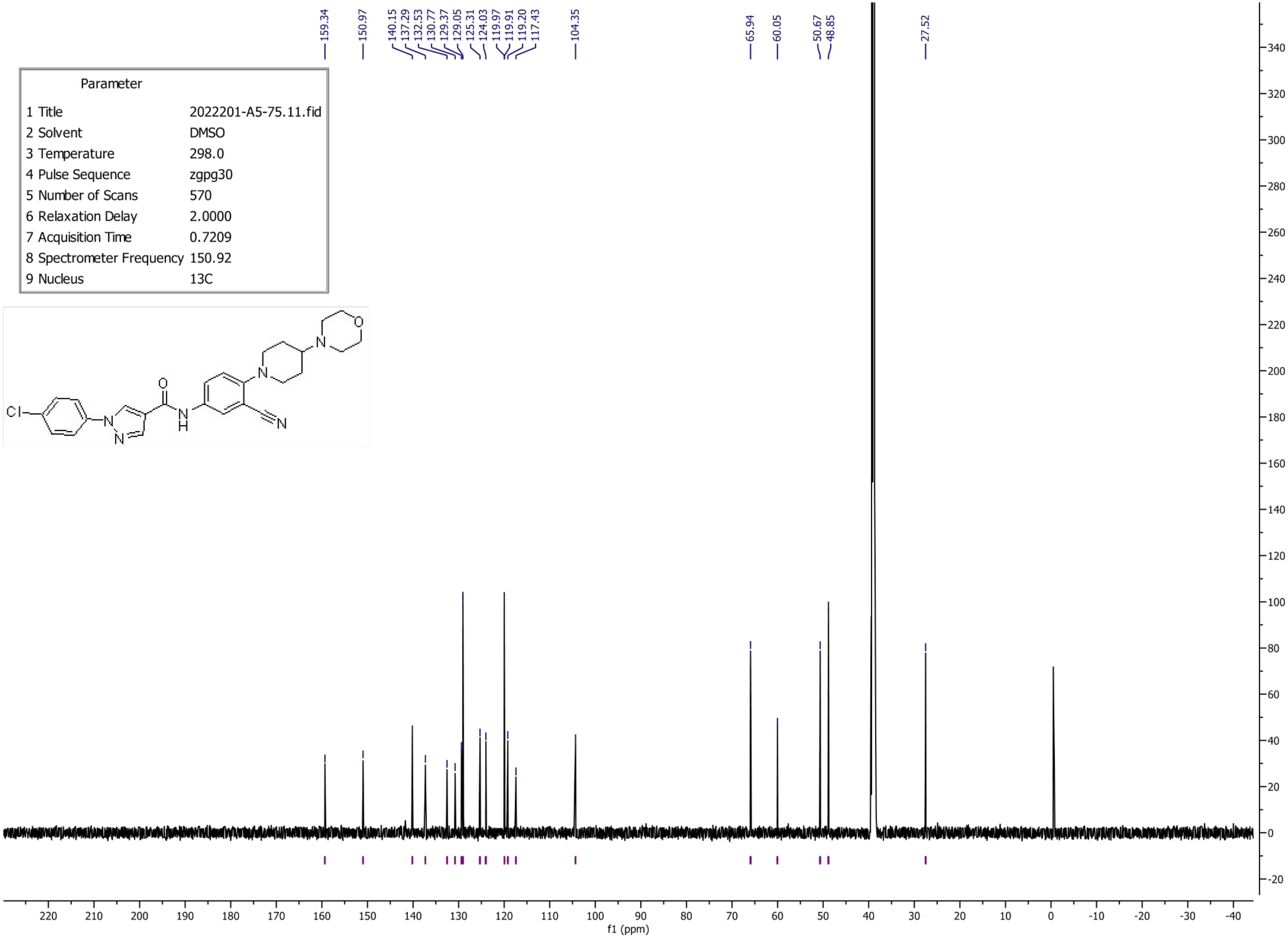

Parameter	
1 Title	2022201-A5-75.11.fid
2 Solvent	DMSO
3 Temperature	298.0
4 Pulse Sequence	zgpg30
5 Number of Scans	570
6 Relaxation Delay	2.0000
7 Acquisition Time	0.7209
8 Spectrometer Frequency	150.92
9 Nucleus	13C

Figure S56 ^{13}C NMR spectrum of compound **24 (BY-001)** (DMSO-d6, 151 MHz)

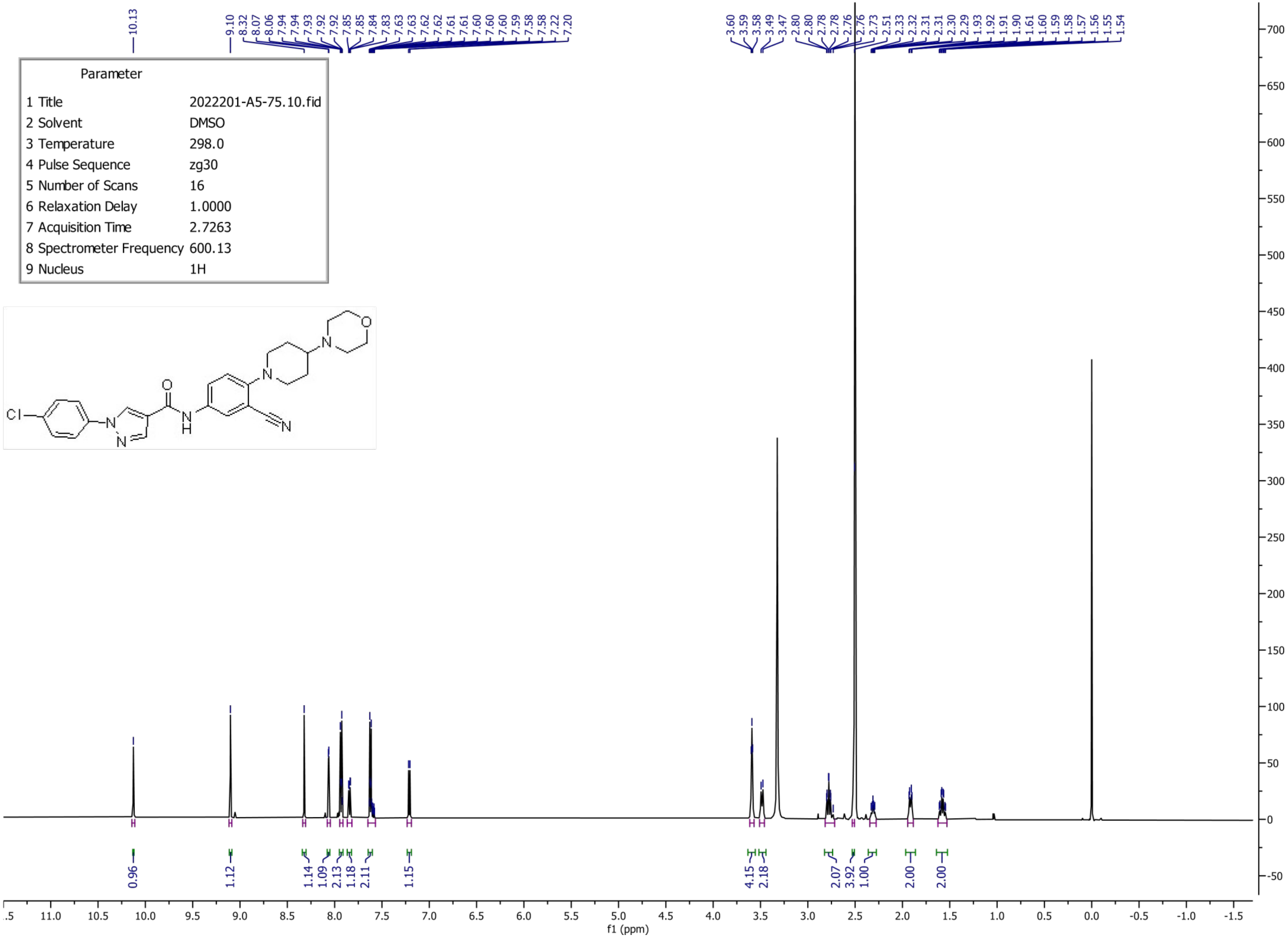

Figure S57 ^{1}H NMR spectrum of compound **24 (BY-001)** (DMSO-d6, 600 MHz)

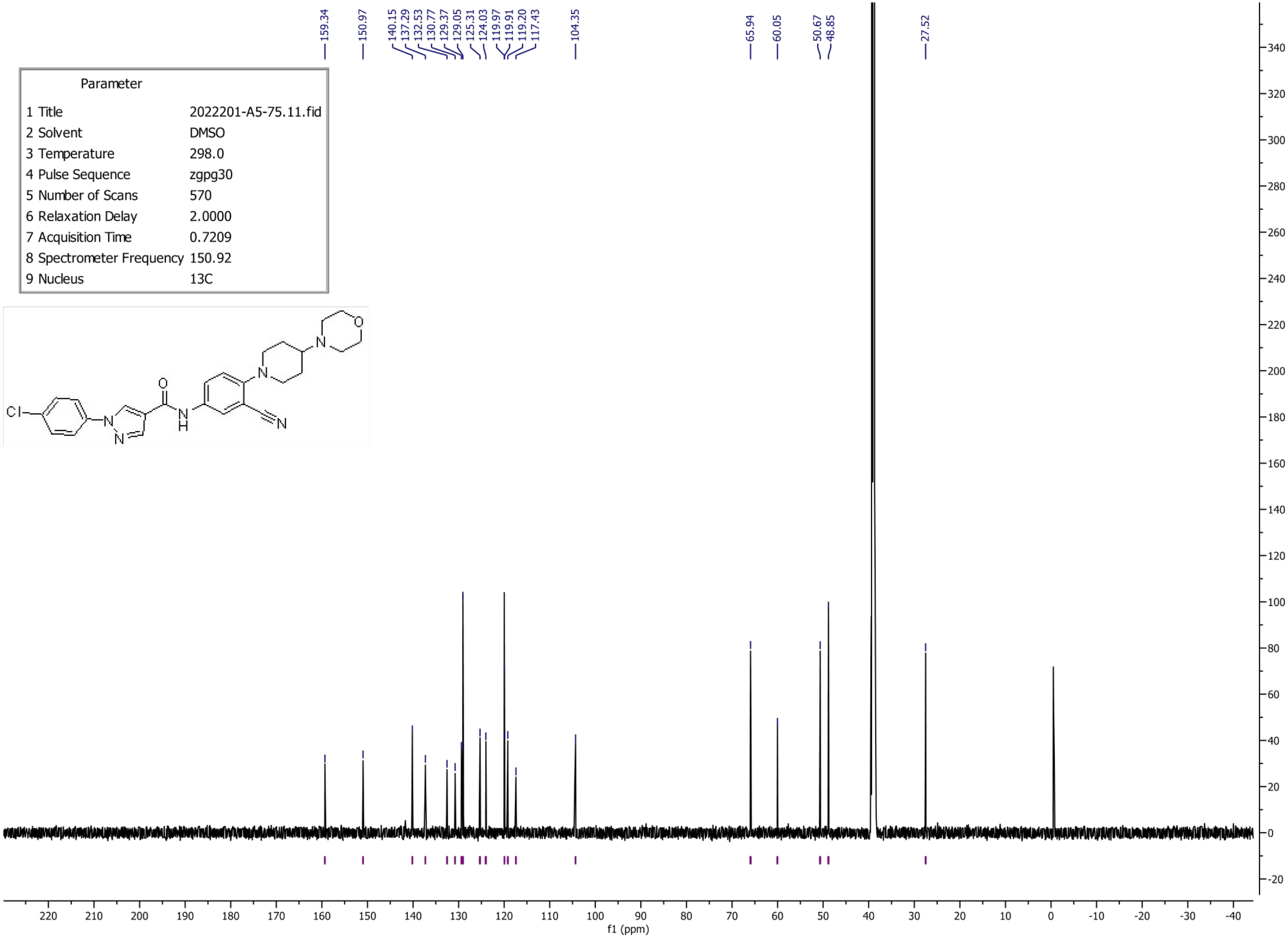

Figure S58 ^{13}C NMR spectrum of compound **24 (BY-001)** (DMSO-d6, 151 MHz)

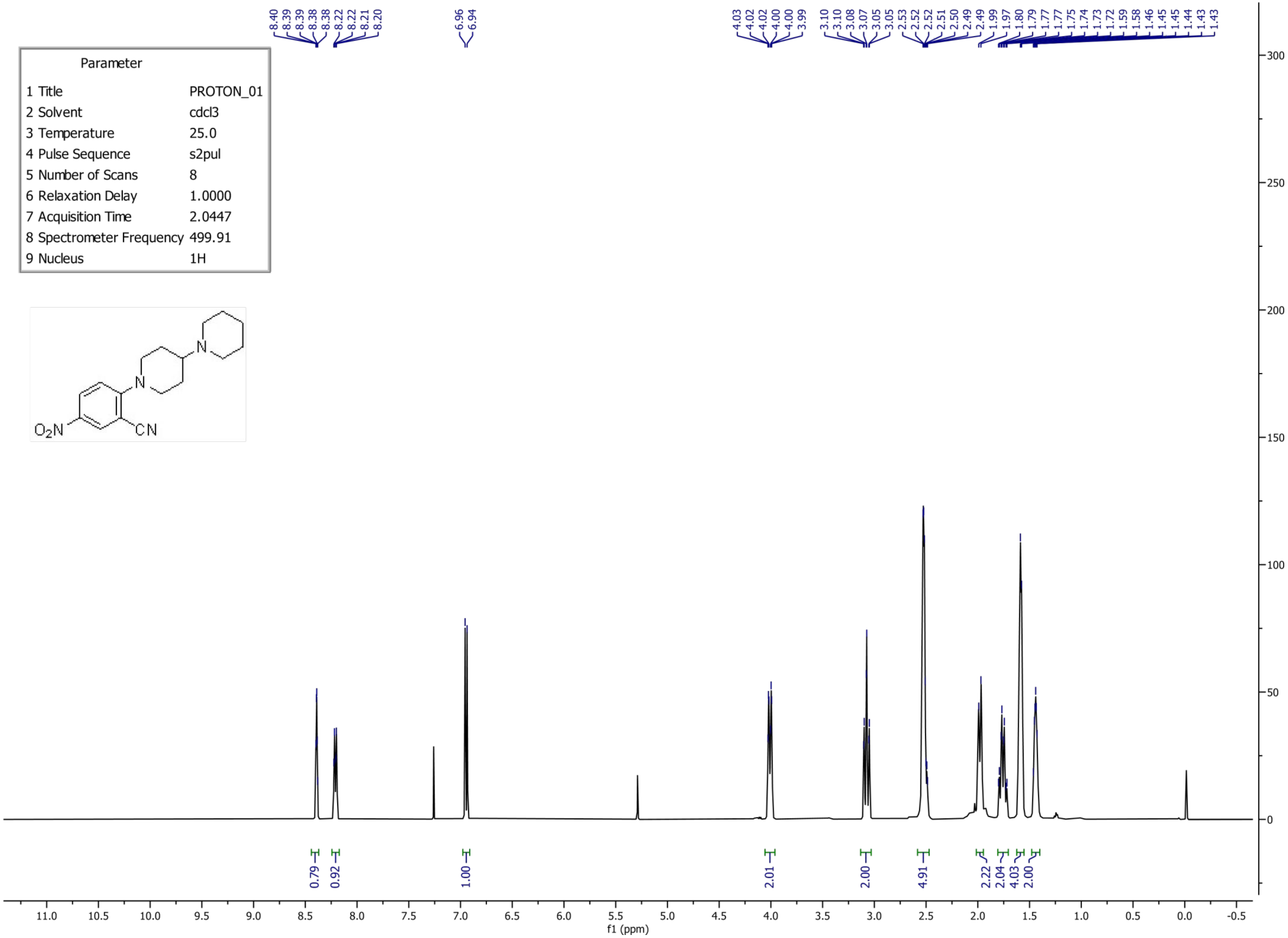

Figure S59 ^{1}H NMR spectrum of compound **25** (CDCl$_3$ 500 MHz)

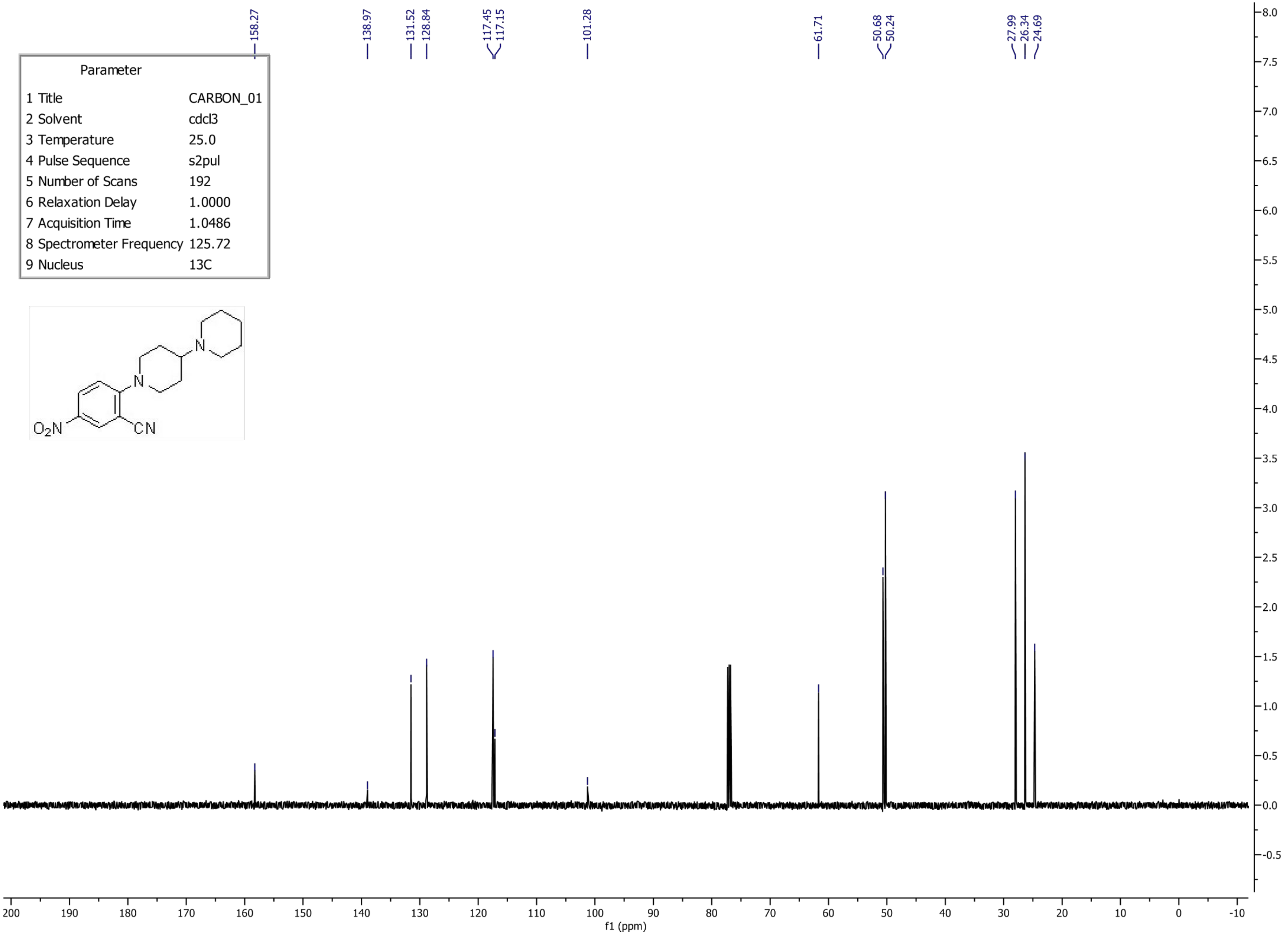

Figure S58 ^{13}C NMR spectrum of compound **25** **(**$CDCl_3$, 126 MHz)

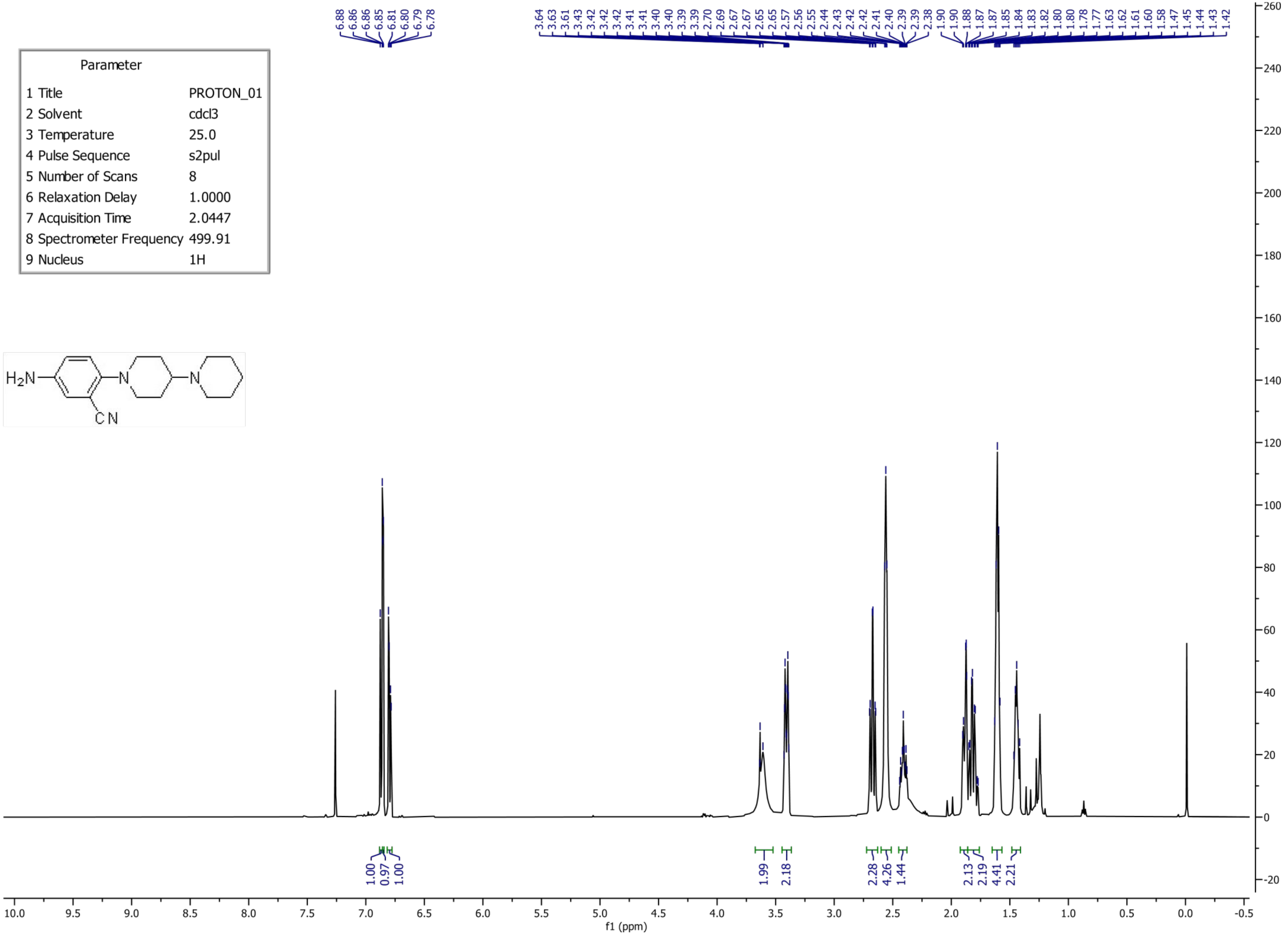

Figure S61 ^{1}H NMR spectrum of compound **26** ($CDCl_3$ 500 MHz)

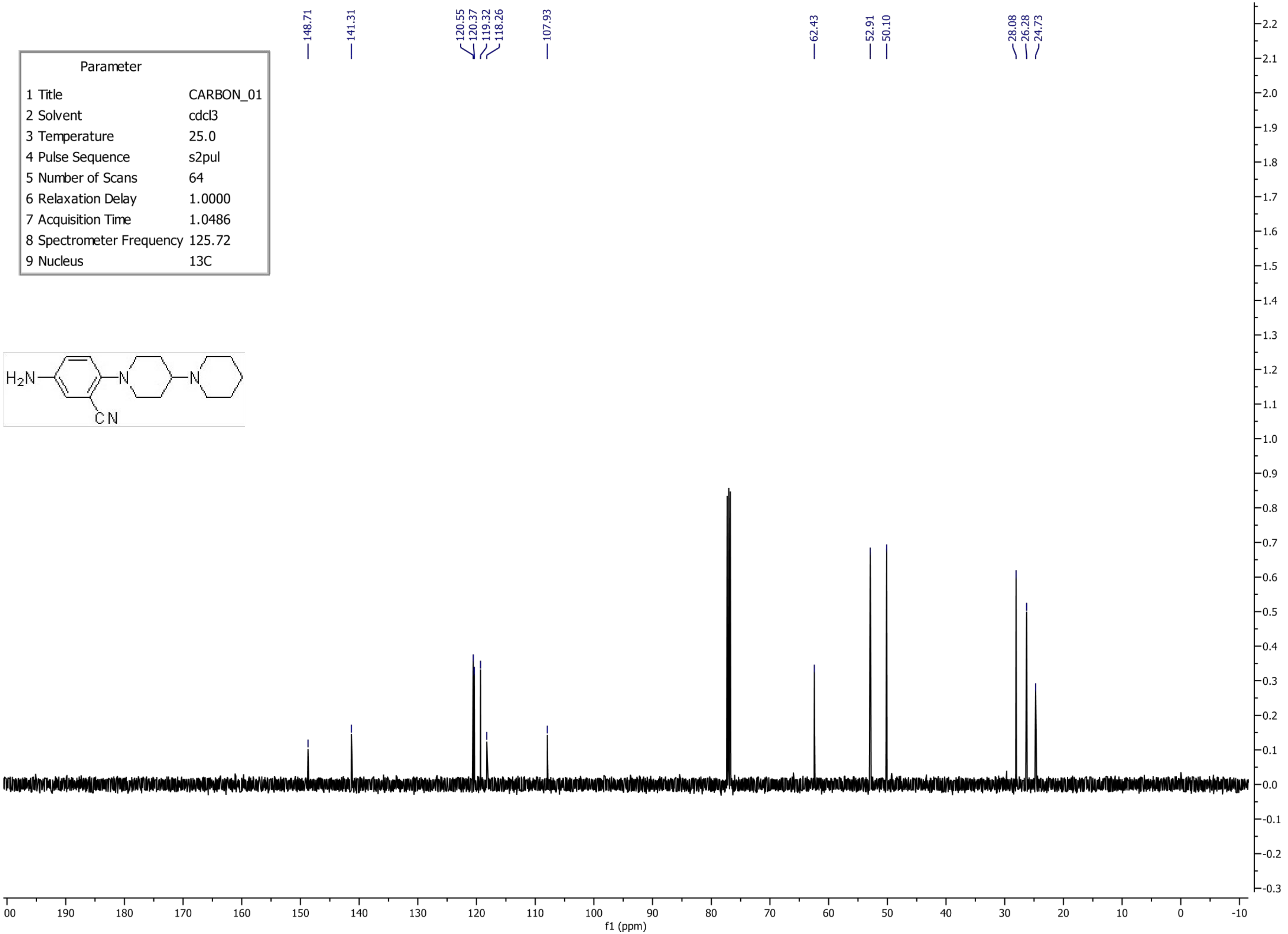

Figure S60 ^{13}C NMR spectrum of compound **26** **(**$CDCl_3$, 126 MHz)

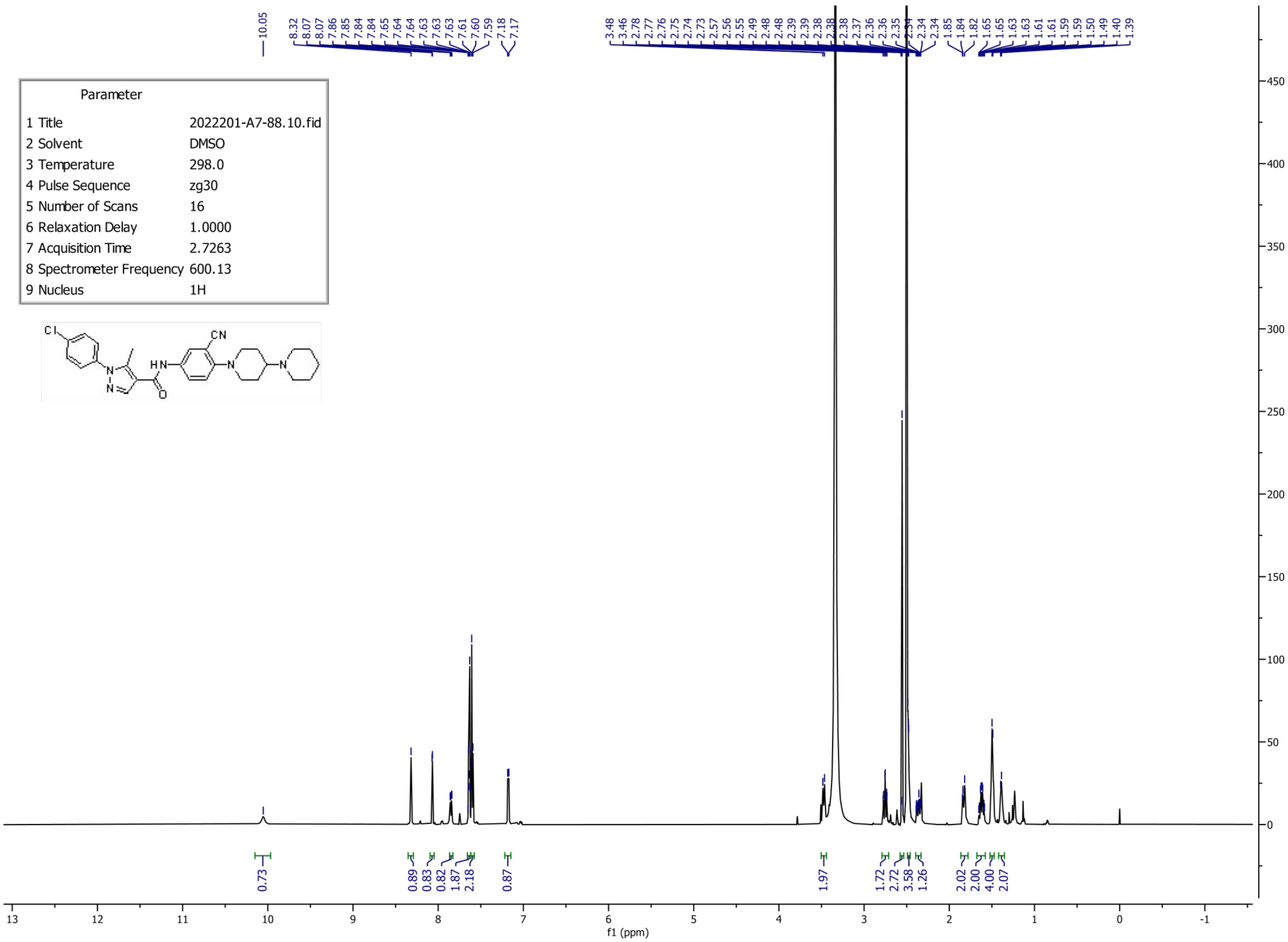

Figure S63 ^{1}H NMR spectrum of compound **27 (BY-018) (**DMSO-d6 600 MHz)

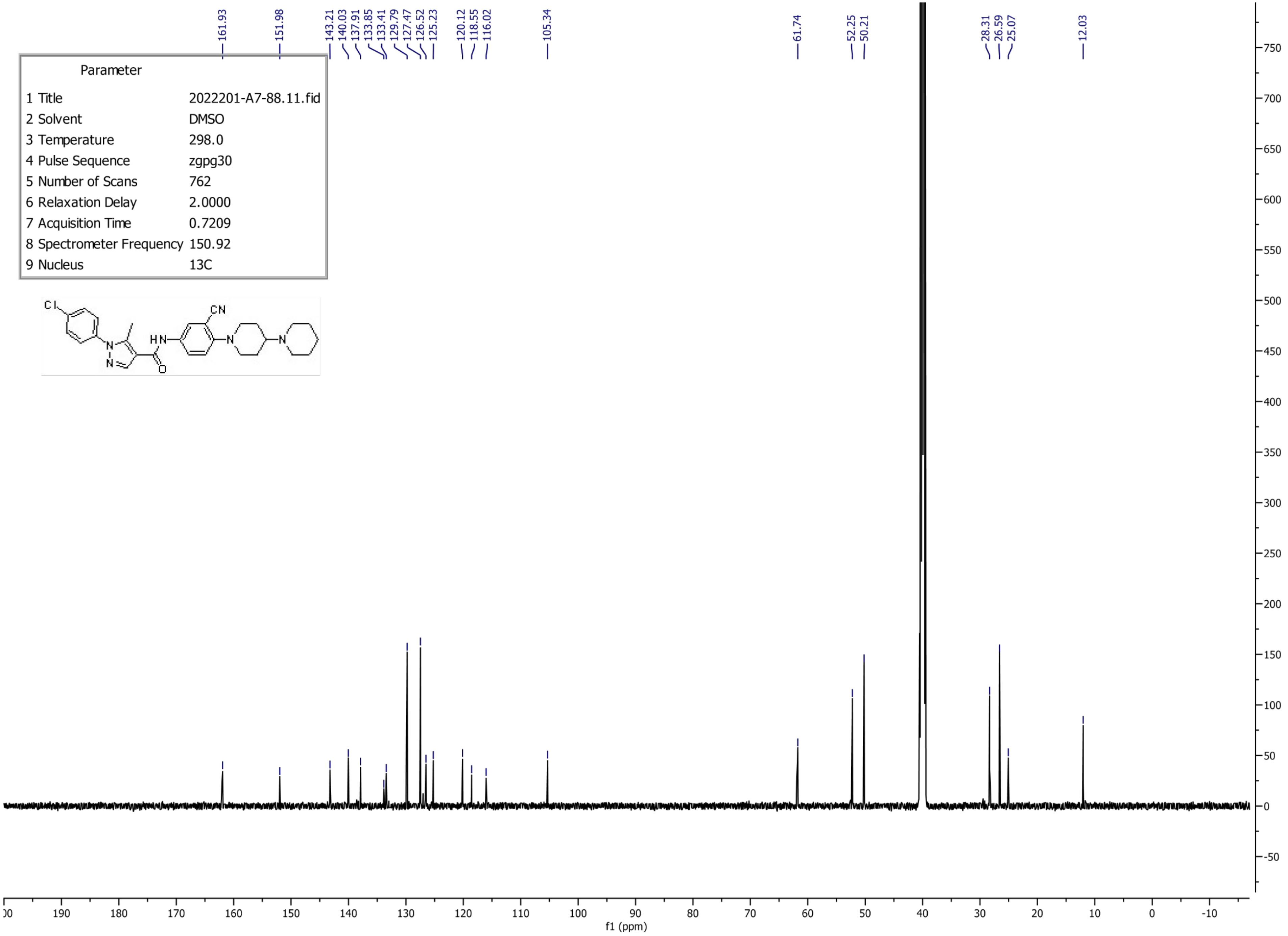

Figure S64 ^{13}C NMR spectrum of compound **27 (BY-018) (**DMSO-d6 151 MHz)

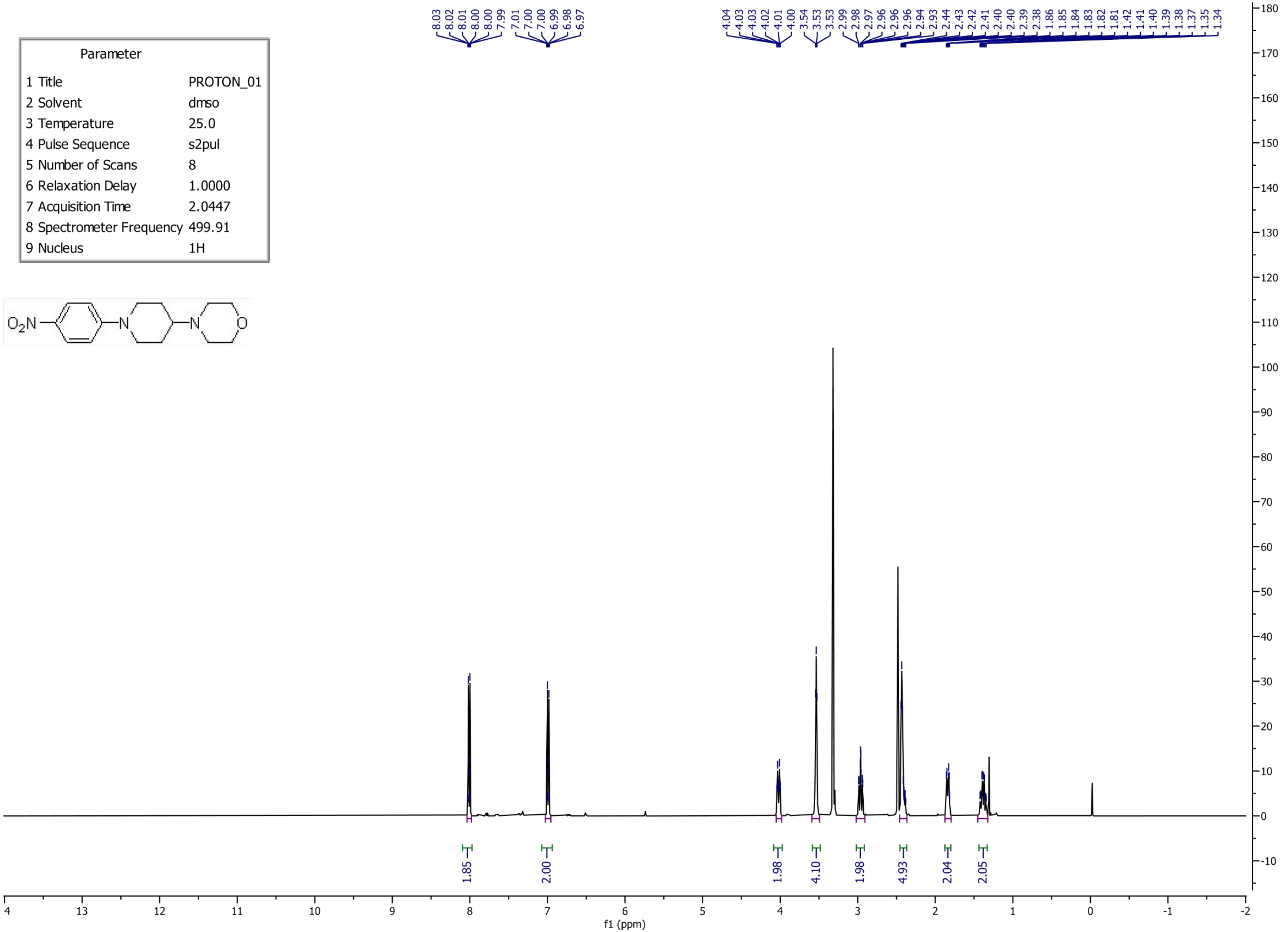

Figure S65 ^{1}H NMR spectrum of compound **28** (DMSO-d6, 500 MHz)

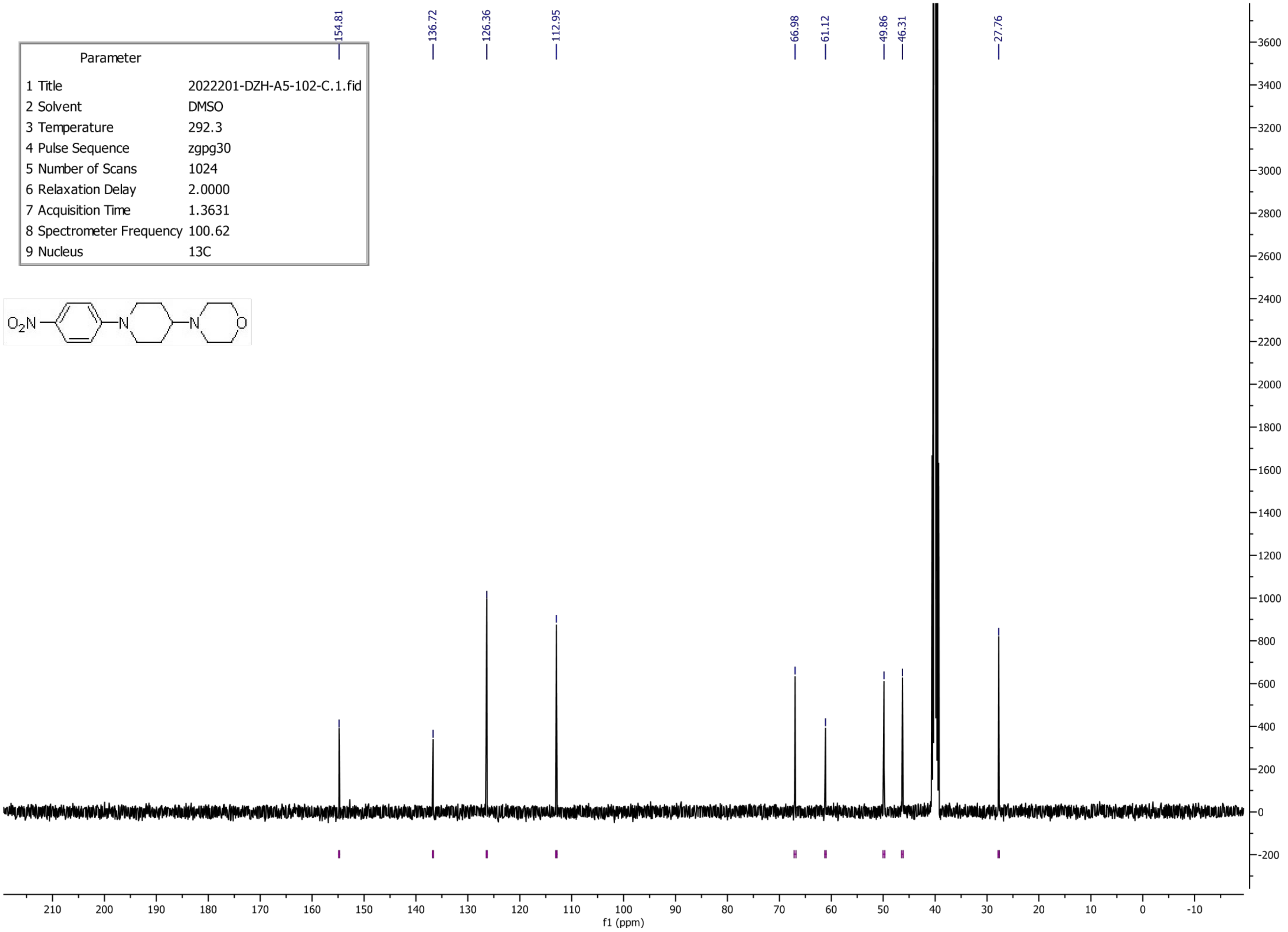

Figure S66 ^{13}C NMR spectrum of compound **28** (DMSO-d6, 100 MHz)

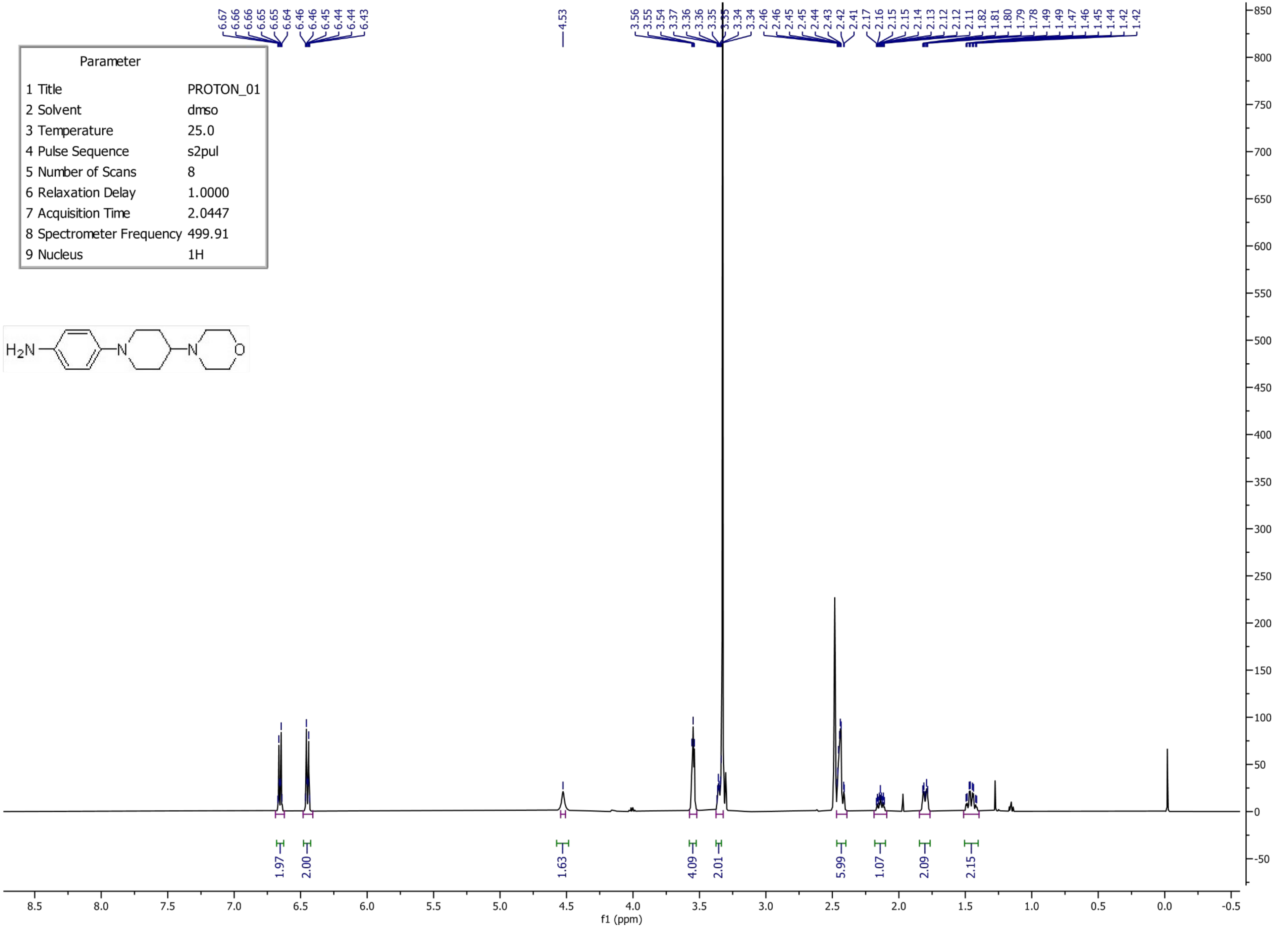

Figure S67 ^{1}H NMR spectrum of compound **29** (DMSO-d6, 500 MHz)

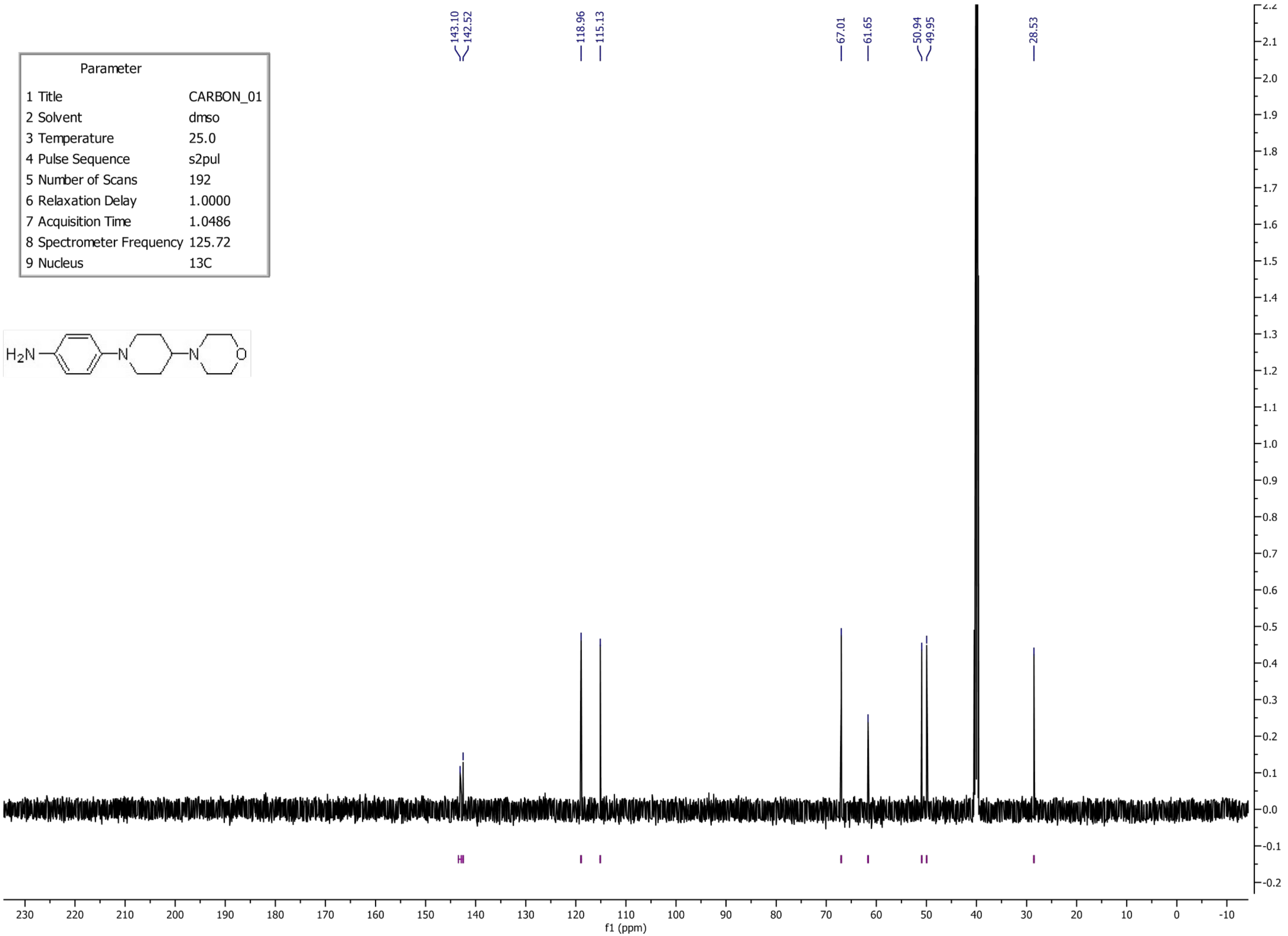

Figure S68 ^{13}C NMR spectrum of compound **29** (DMSO-d6, 126 MHz)

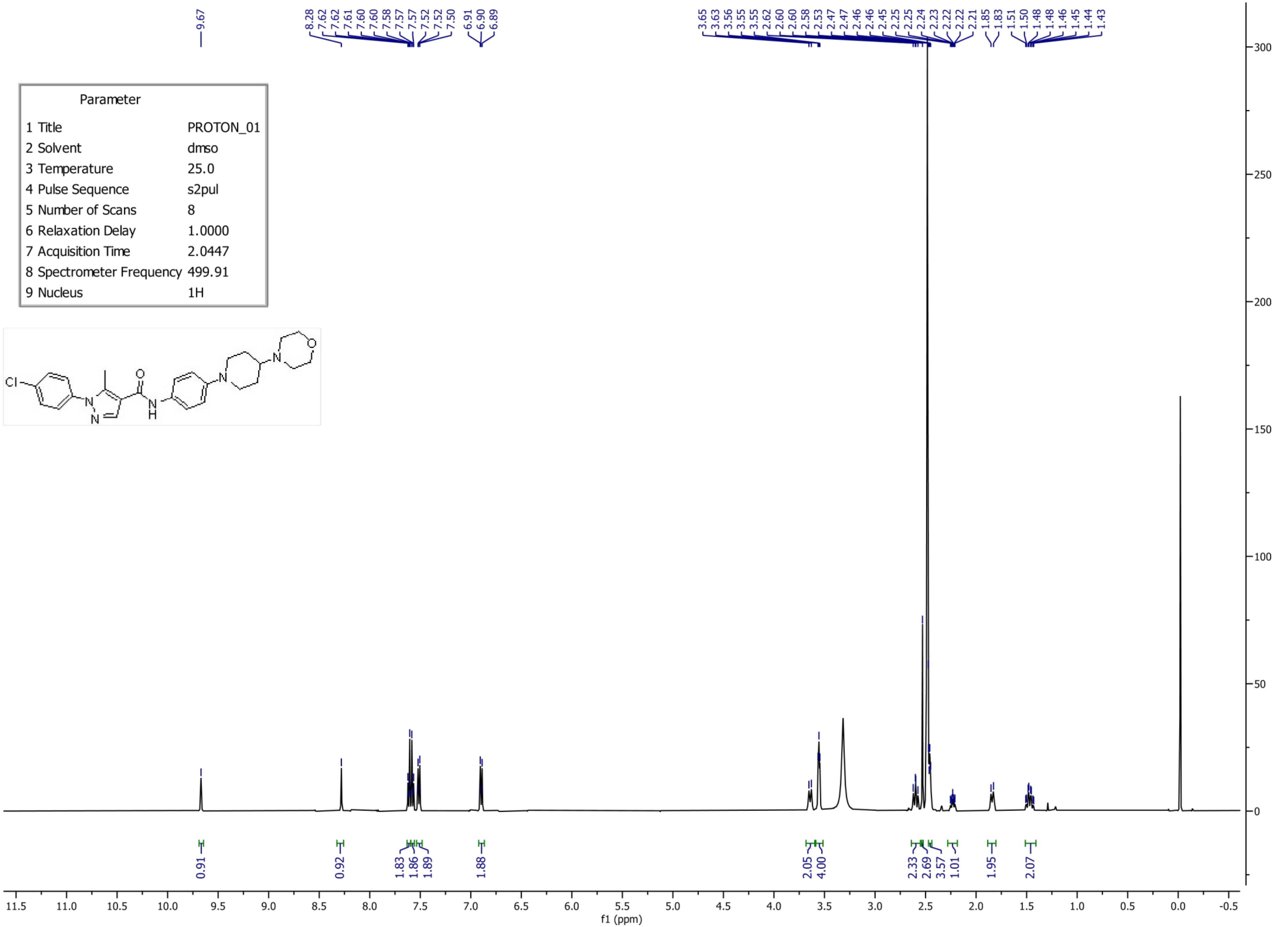

Figure S69 ^{1}H NMR spectrum of compound **30 (BY-007)** (DMSO-d6, 500 MHz)

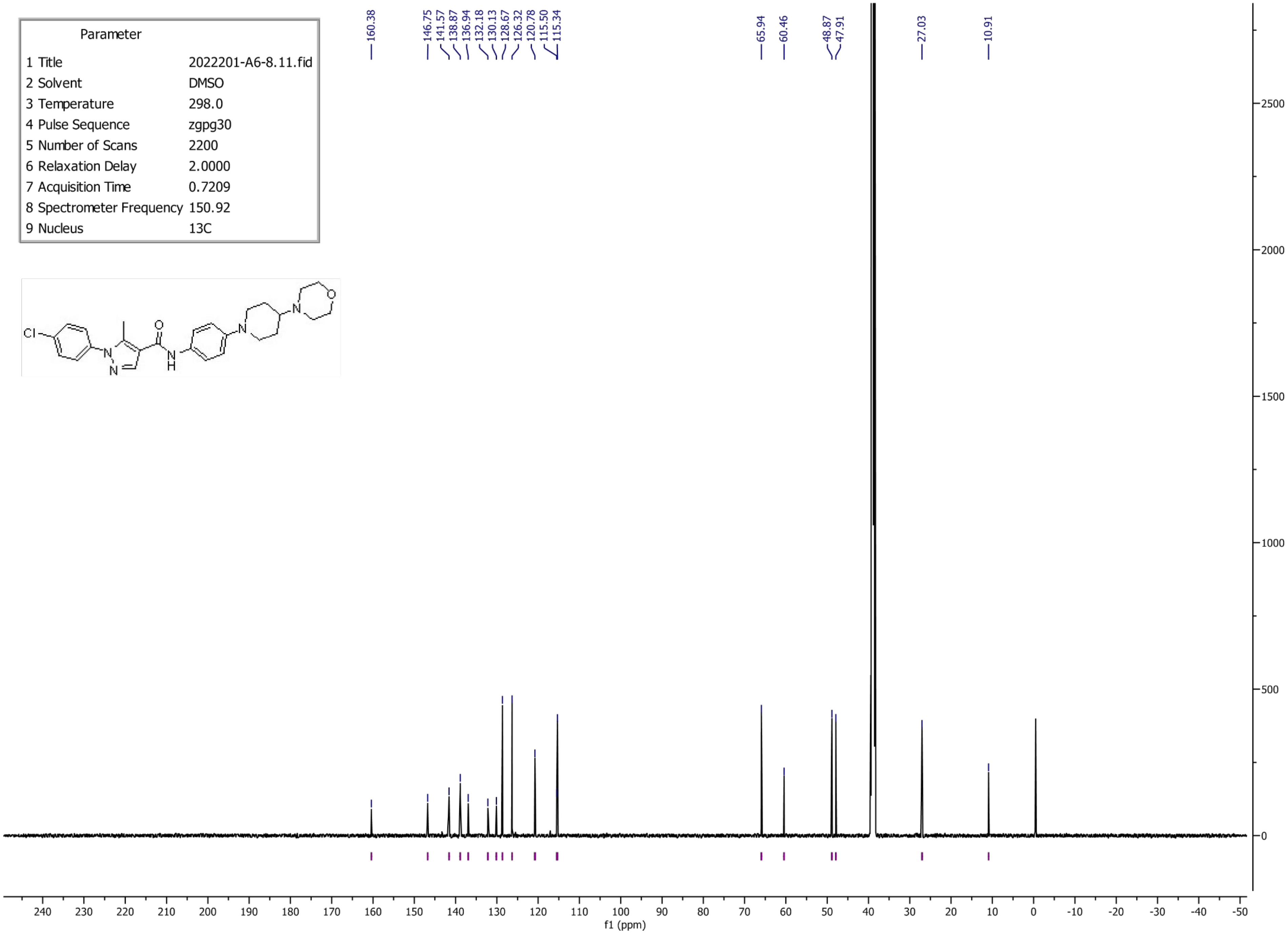

Figure S70 ^{13}C NMR spectrum of compound **30 (BY-007)** (DMSO-d6, 151 MHz)

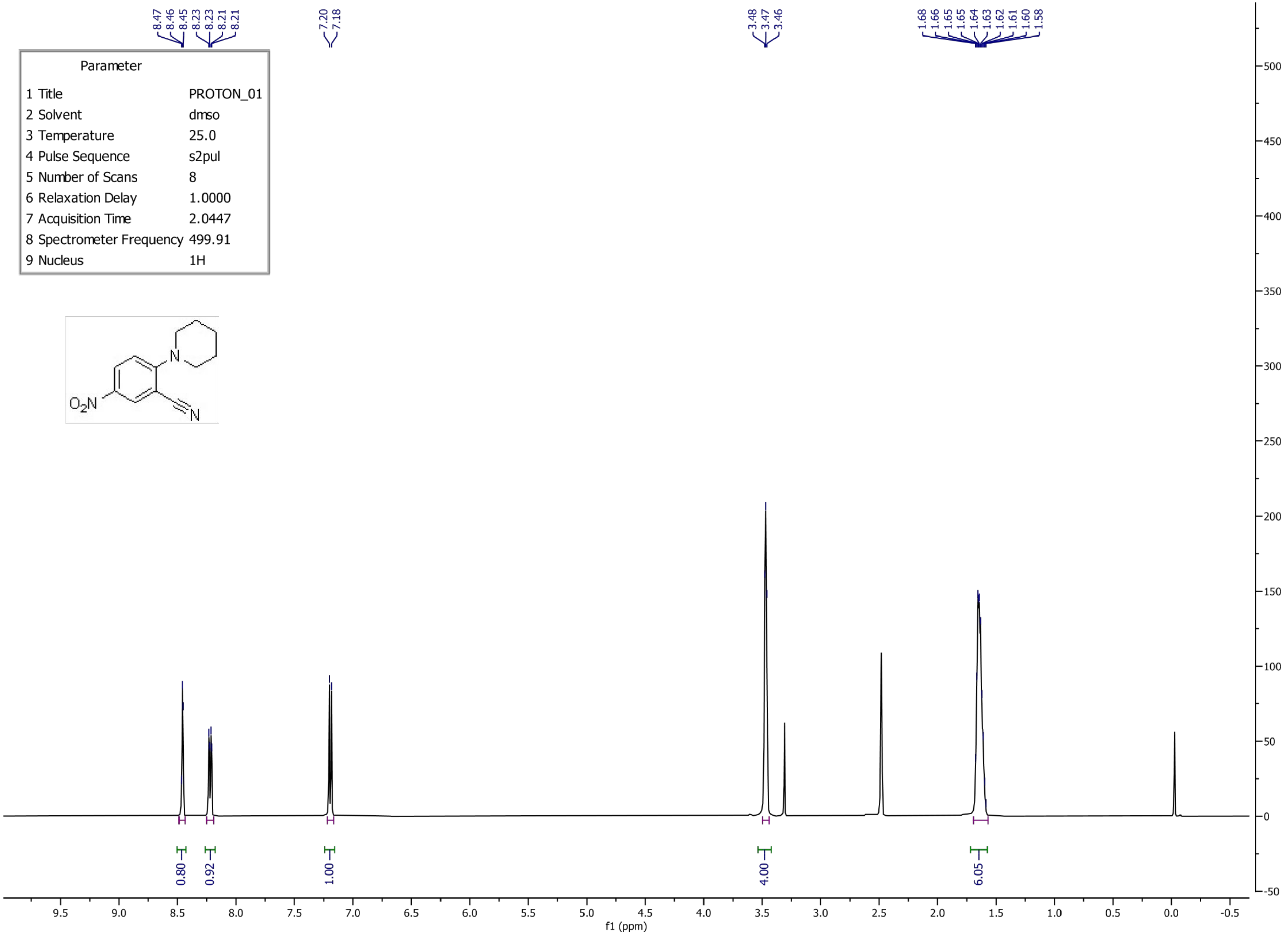

Figure S71 ^{1}H NMR spectrum of compound **31** (DMSO-d6, 500 MHz)

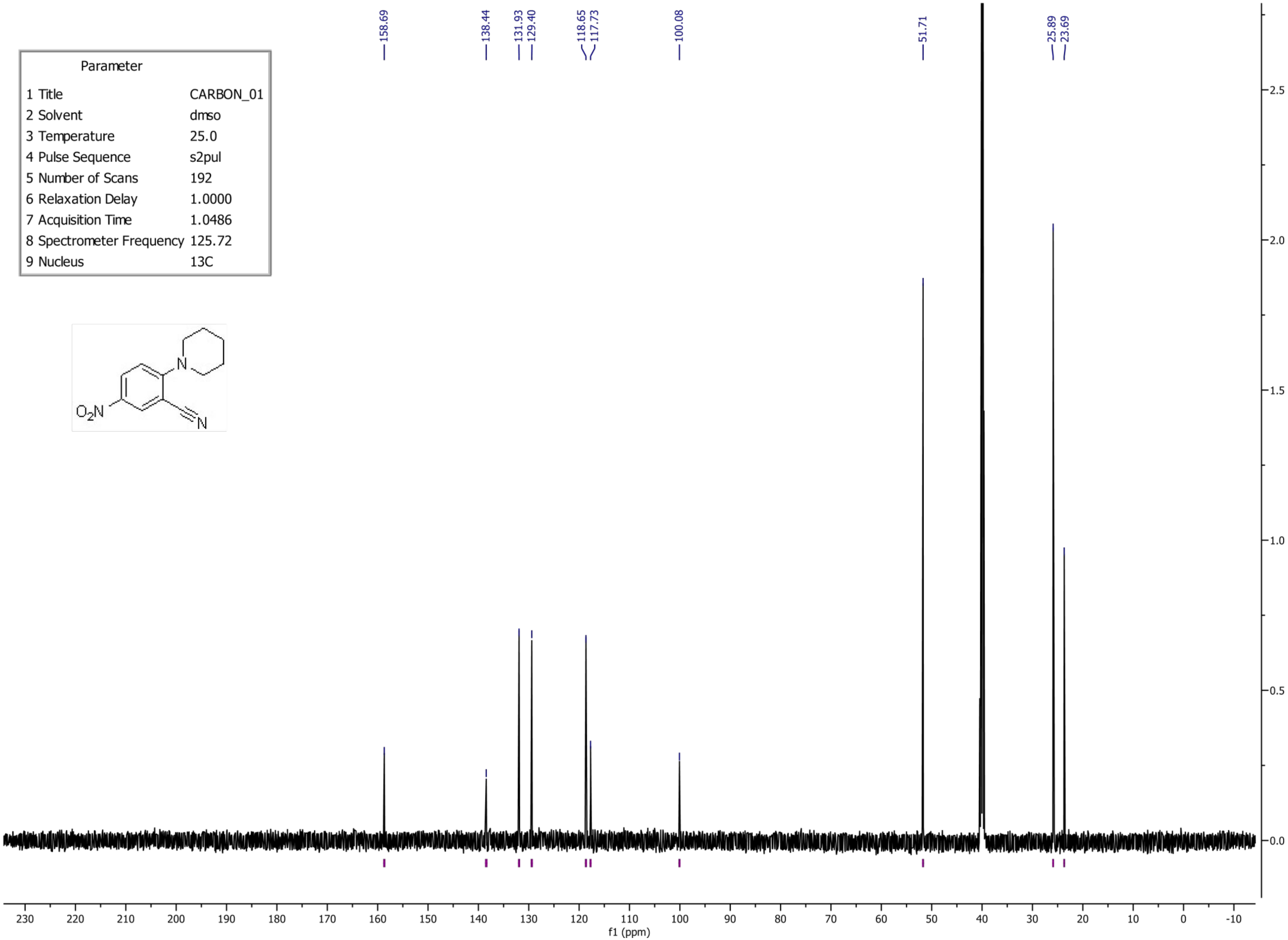

Figure S72 ^{13}C NMR spectrum of compound **31** (DMSO-d6, 126 MHz)

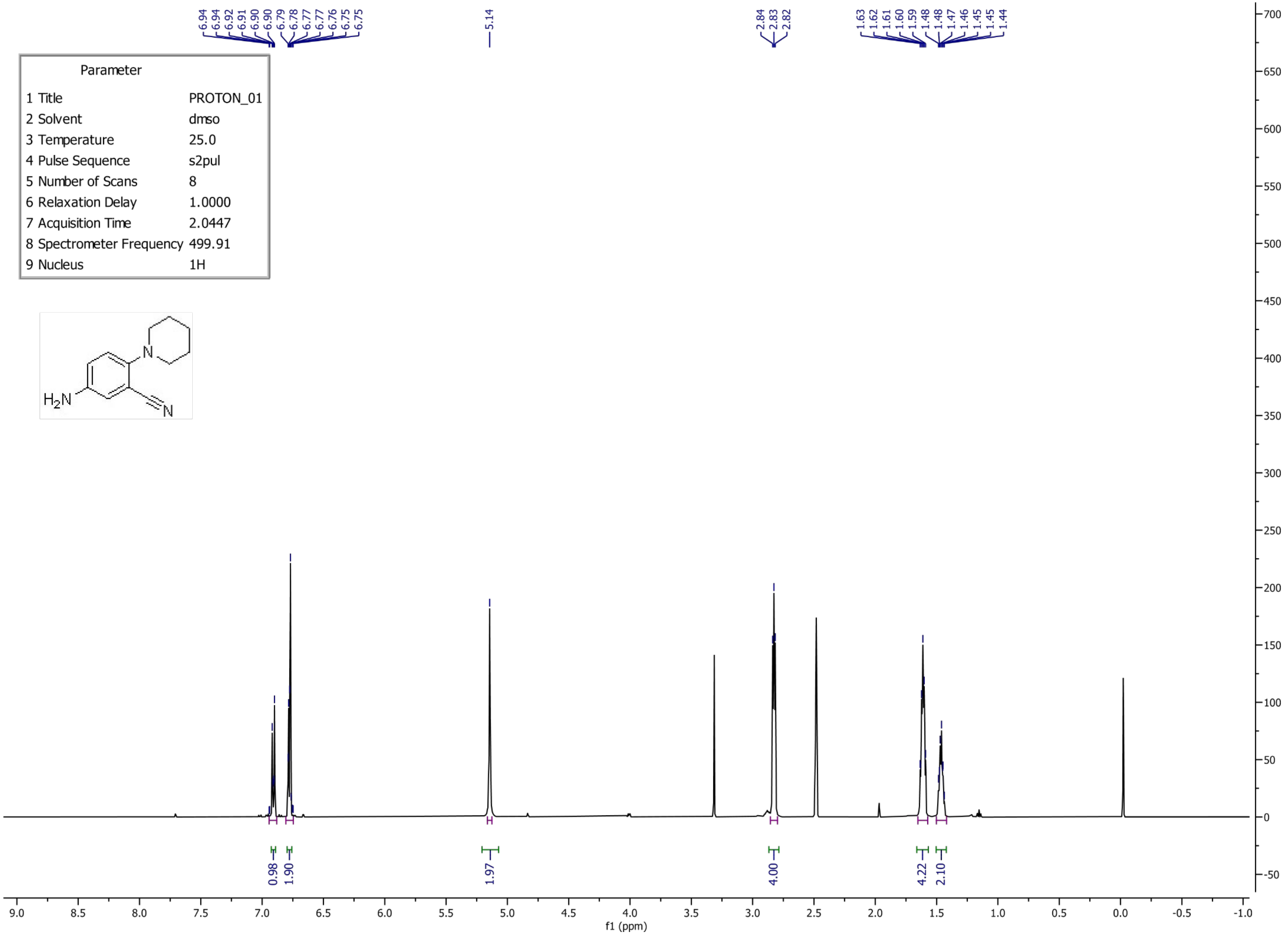

Figure S73 ^{1}H NMR spectrum of compound **32** (DMSO-d6, 500 MHz)

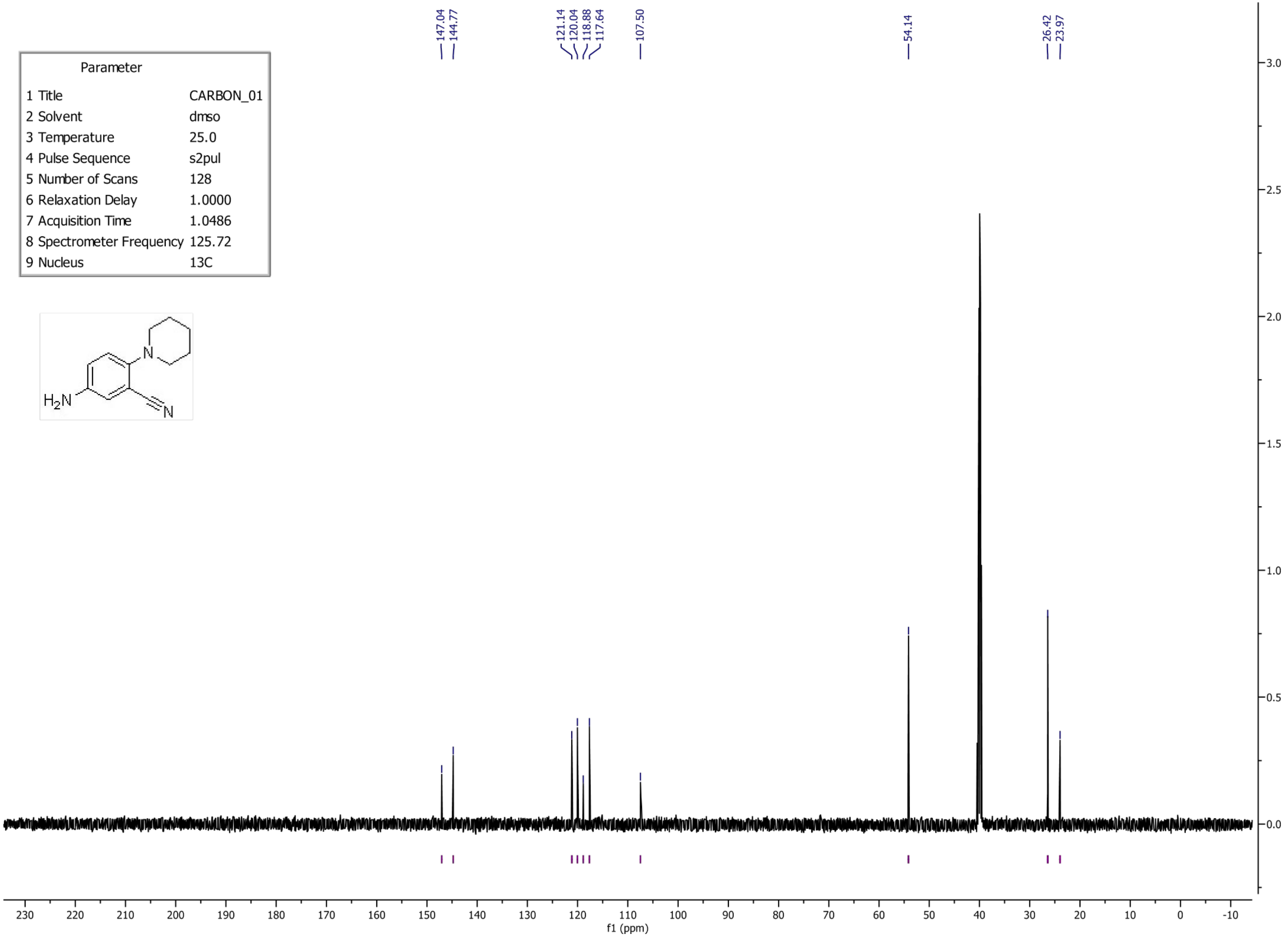

Figure S74 ^{13}C NMR spectrum of compound **32** (DMSO-d6, 126 MHz)

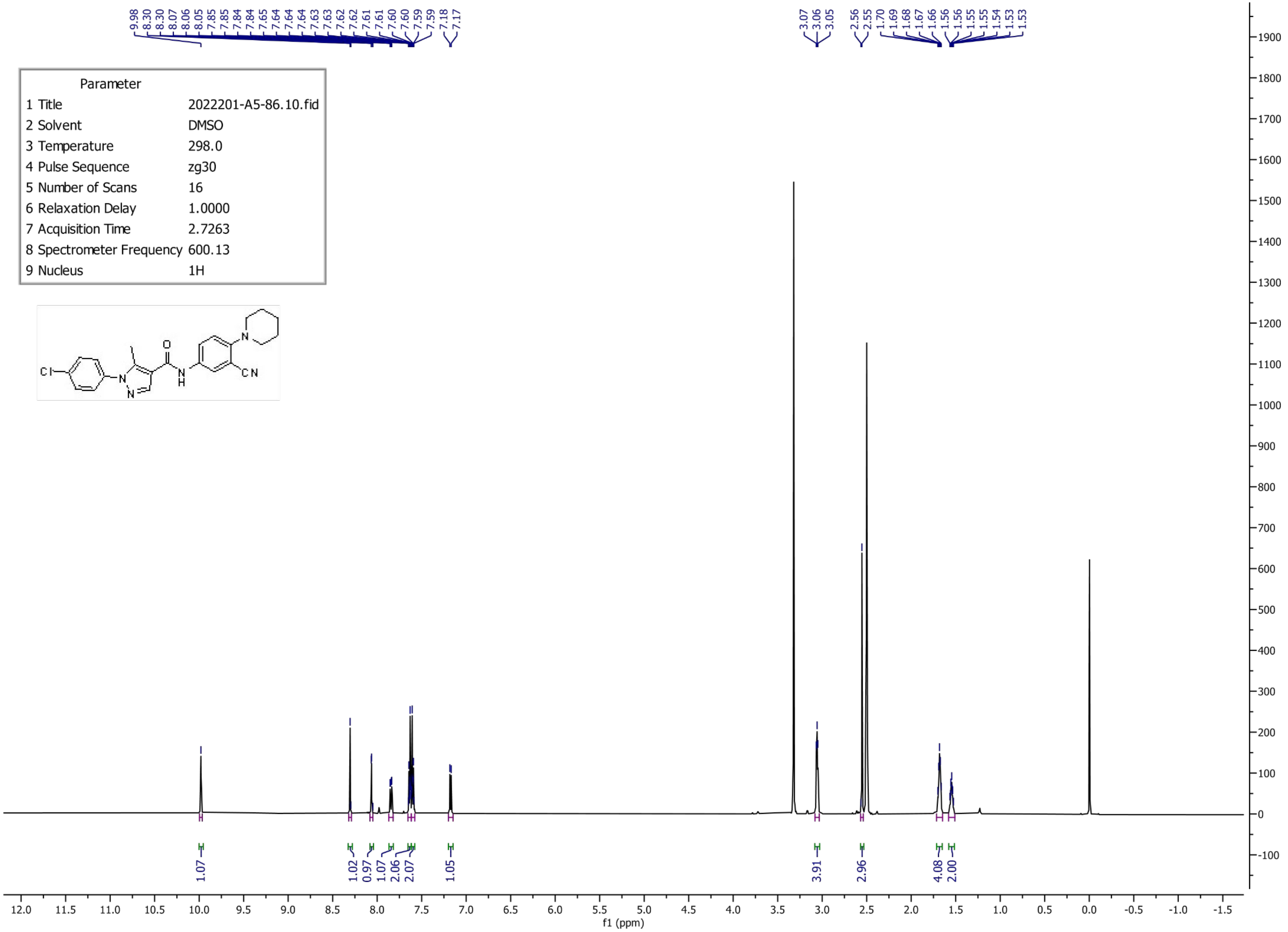

Figure S75 ^{1}H NMR spectrum of compound **33 (BY-003)** (DMSO-d6, 600 MHz)

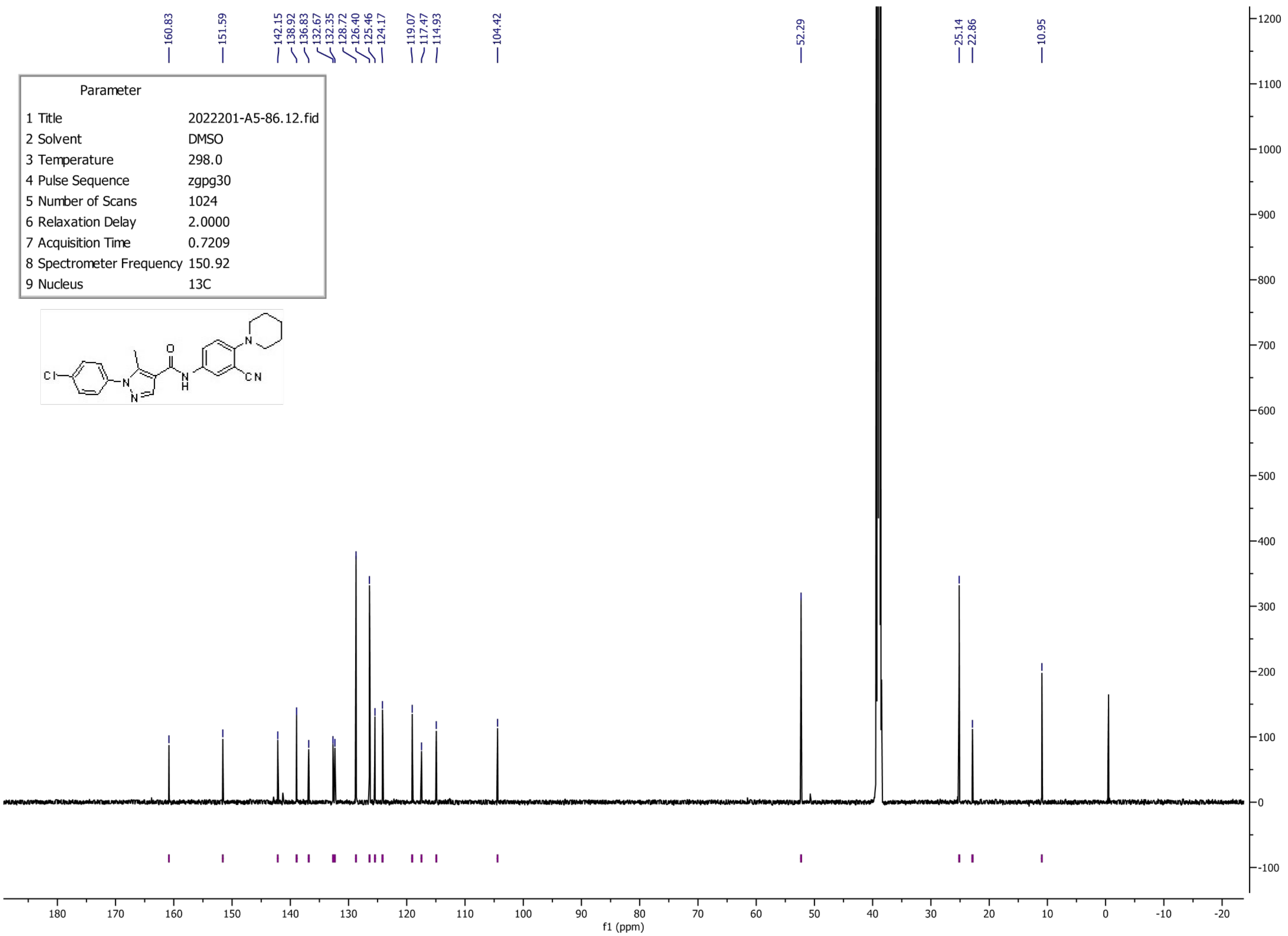

Figure S76 ^{13}C NMR spectrum of compound **33 (BY-003)** (DMSO-d6, 151 MHz)

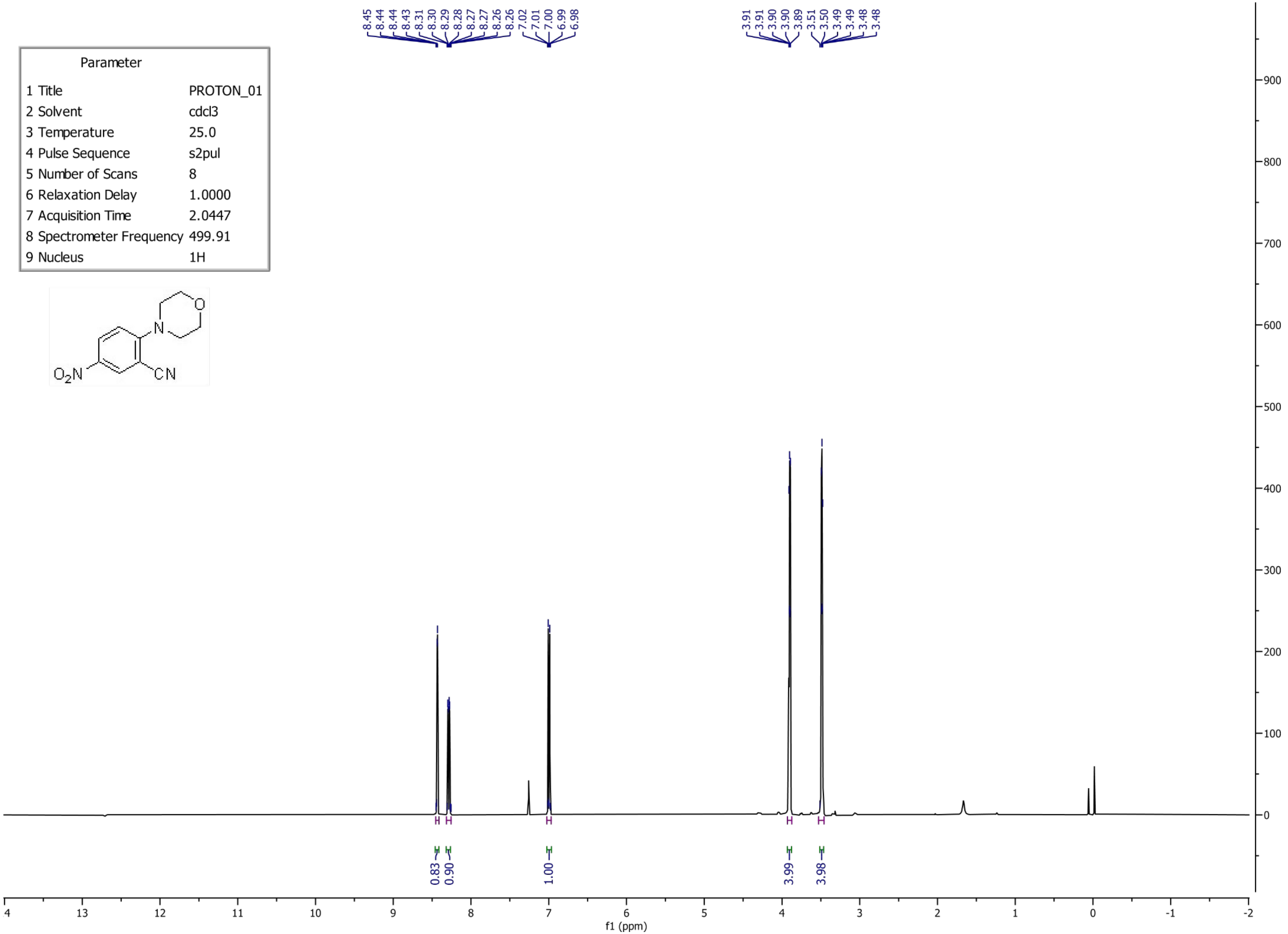

Figure S77 ^{1}H NMR spectrum of compound **334** ($CDCl_3$, 500 MHz)

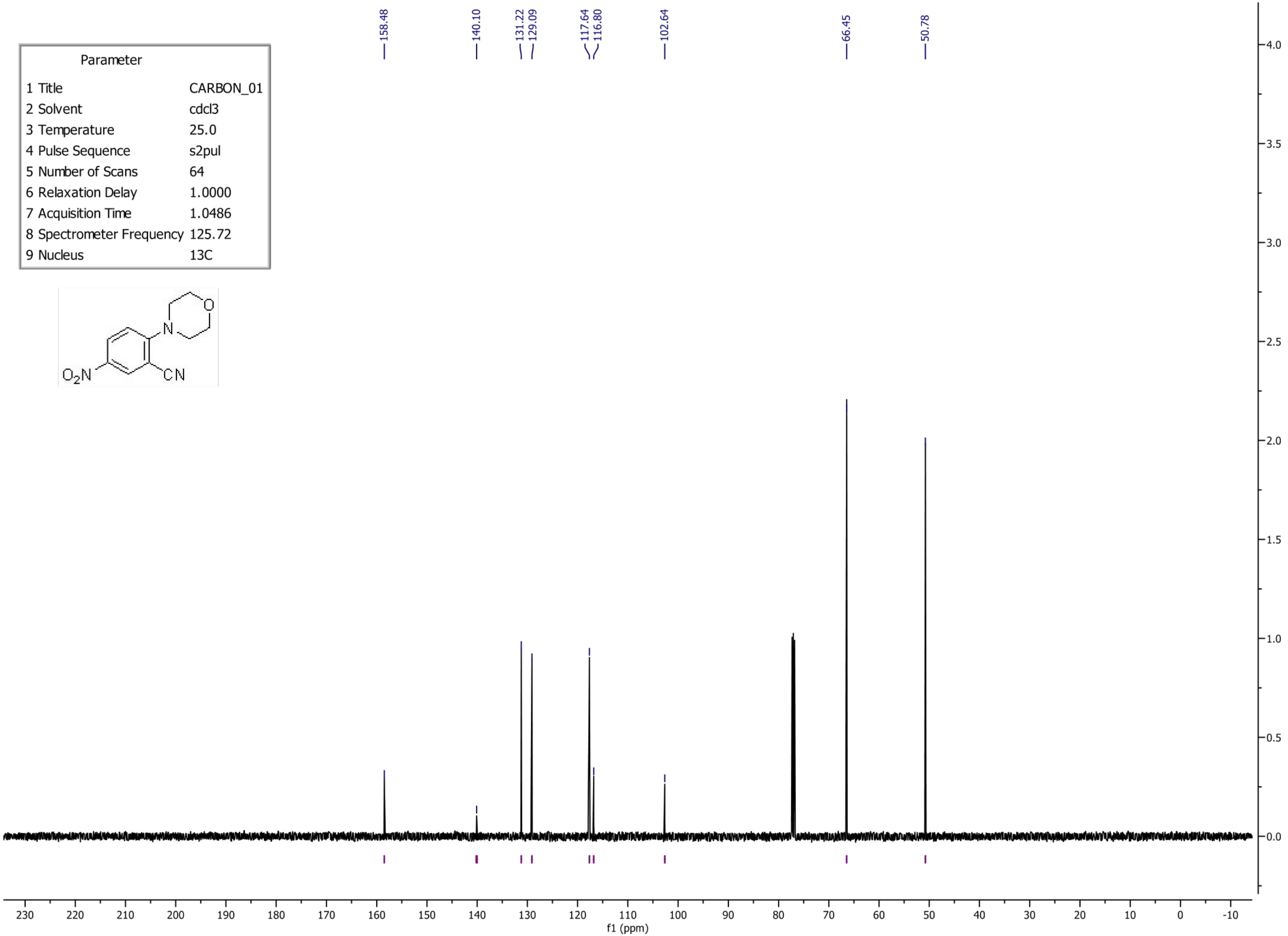

Figure S78 ^{13}C NMR spectrum of compound **34** ($CDCl_3$, 126 MHz)

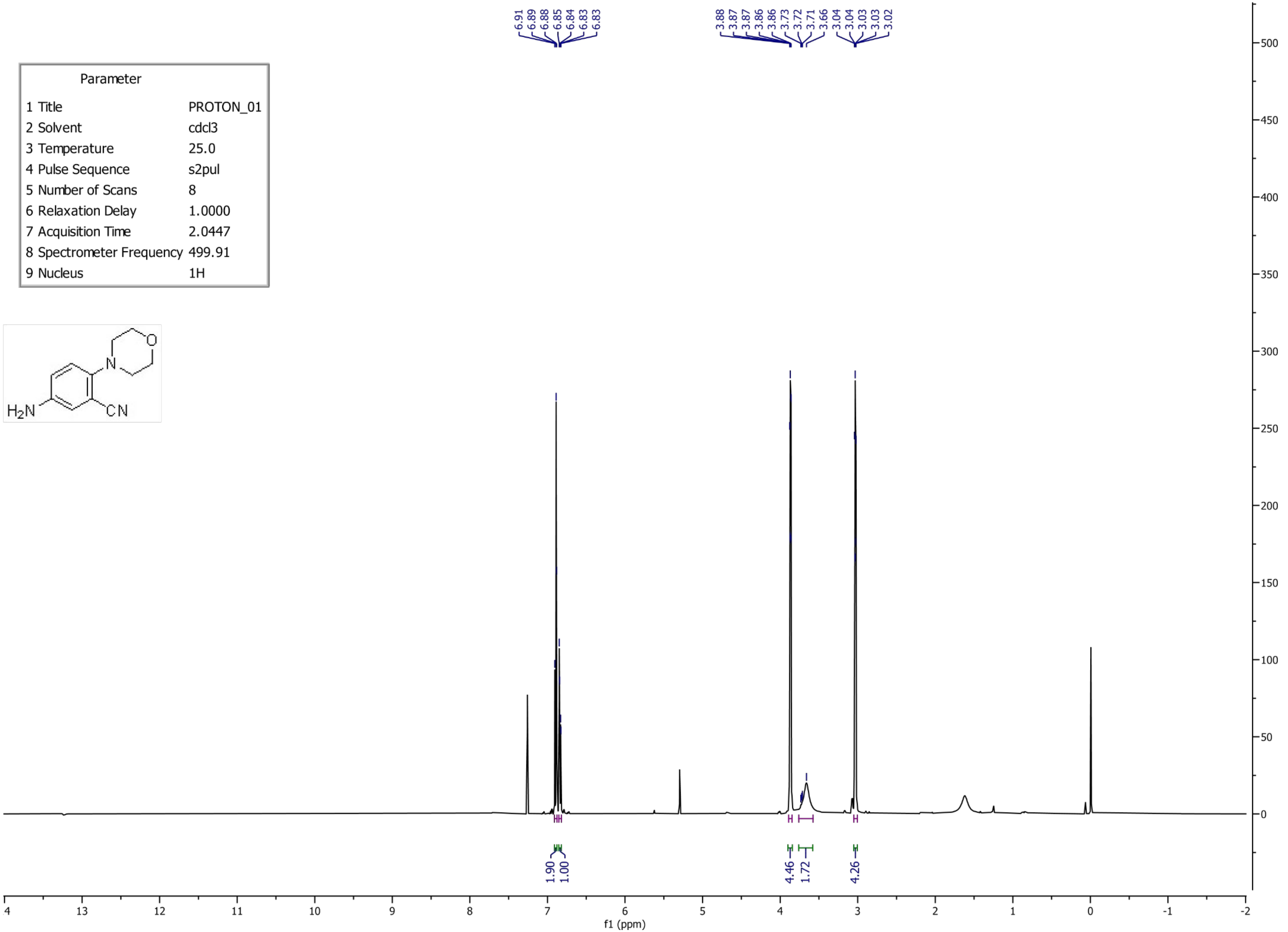

Figure S79 ^{1}H NMR spectrum of compound **35** ($CDCl_3$, 500 MHz)

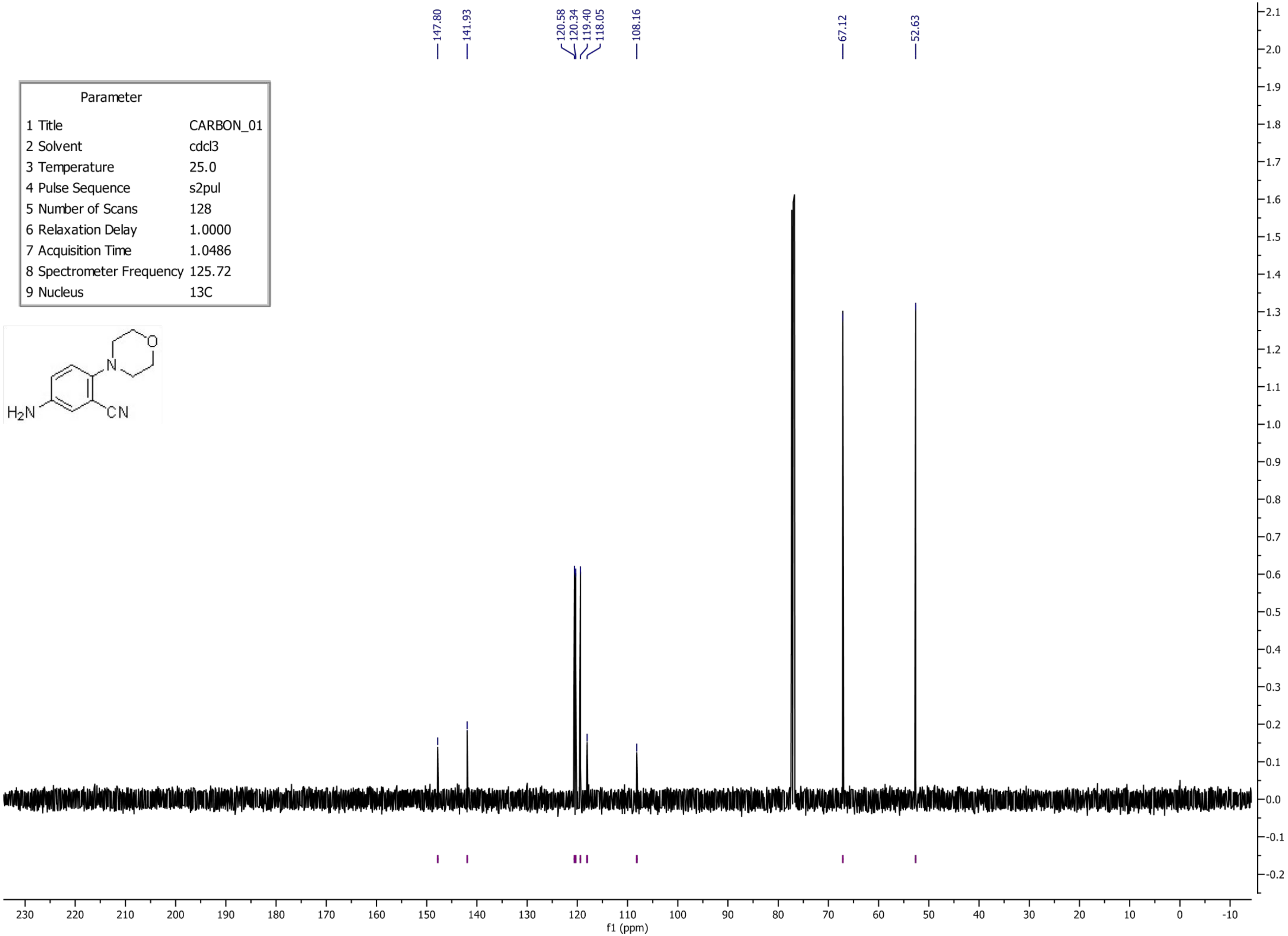

Figure S80 ^{13}C NMR spectrum of compound **35** ($CDCl_3$, 126 MHz)

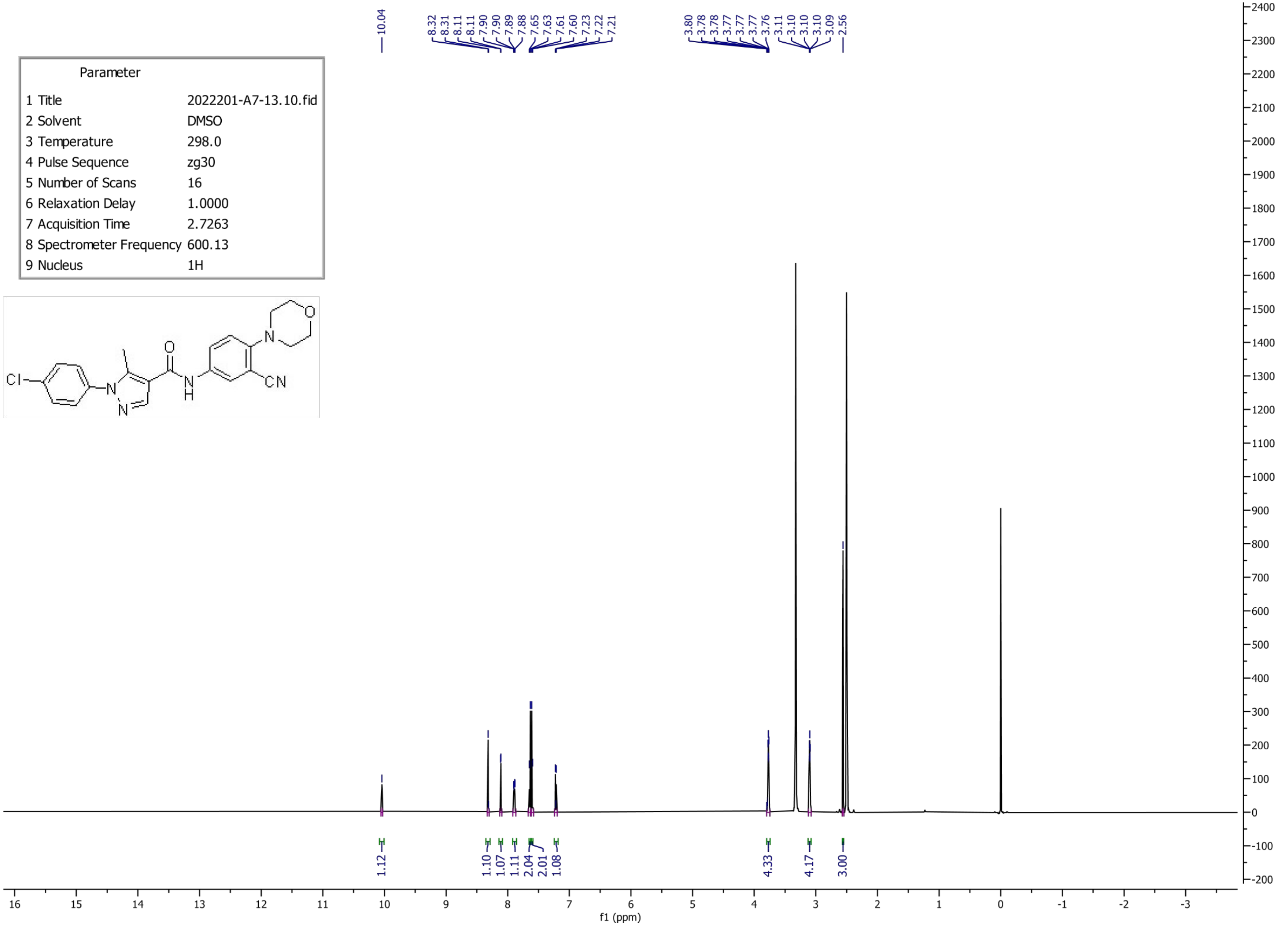

Figure S81 ^{1}H NMR spectrum of compound **36 (BY-010)** (DMSO-d6, 600 MHz)

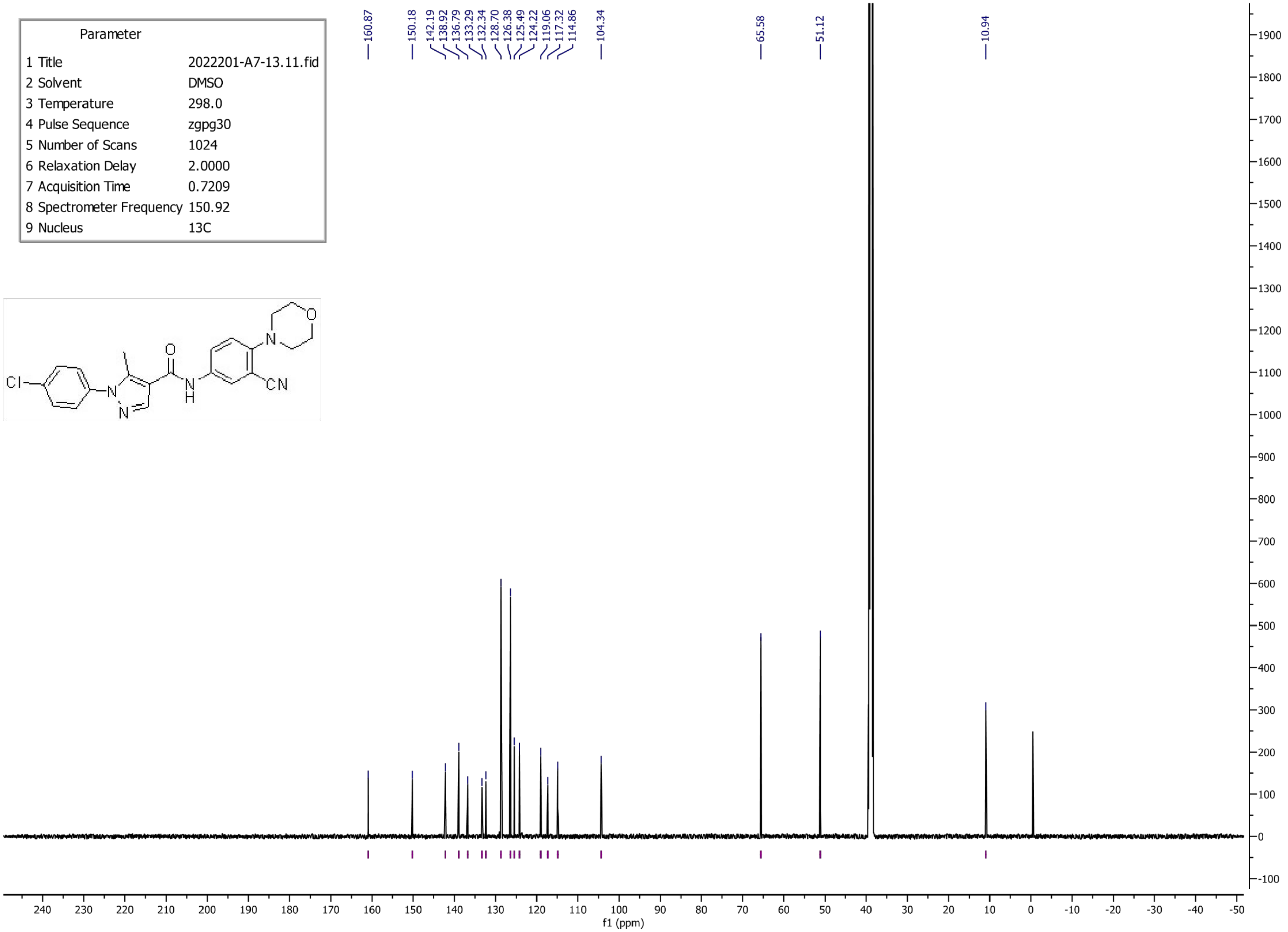

Figure S82 ^{13}C NMR spectrum of compound **36 (BY-010)** (DMSO-d6, 151 MHz)

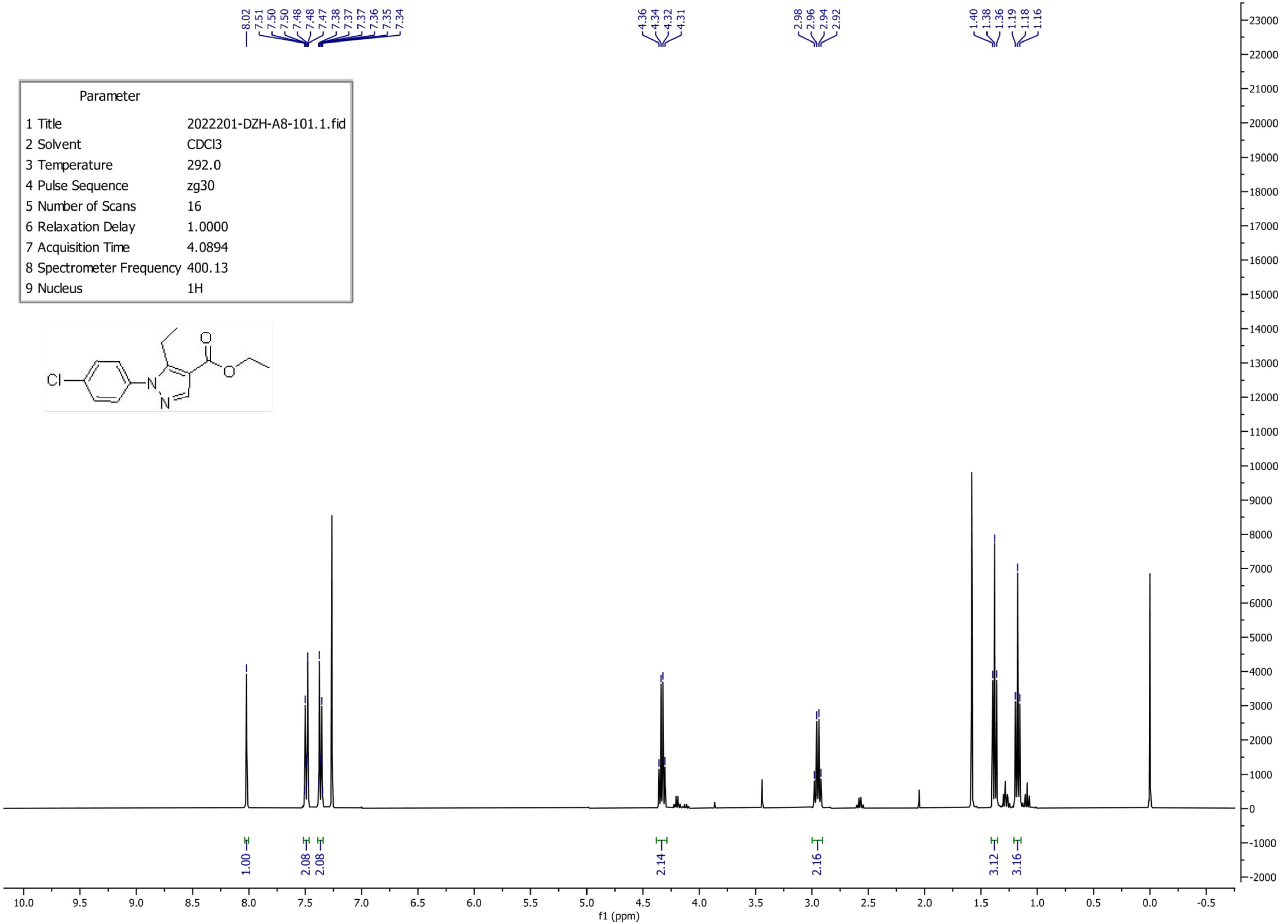

Figure S83 ^{1}H NMR spectrum of compound **37** ($CDCl_3$, 400 MHz)

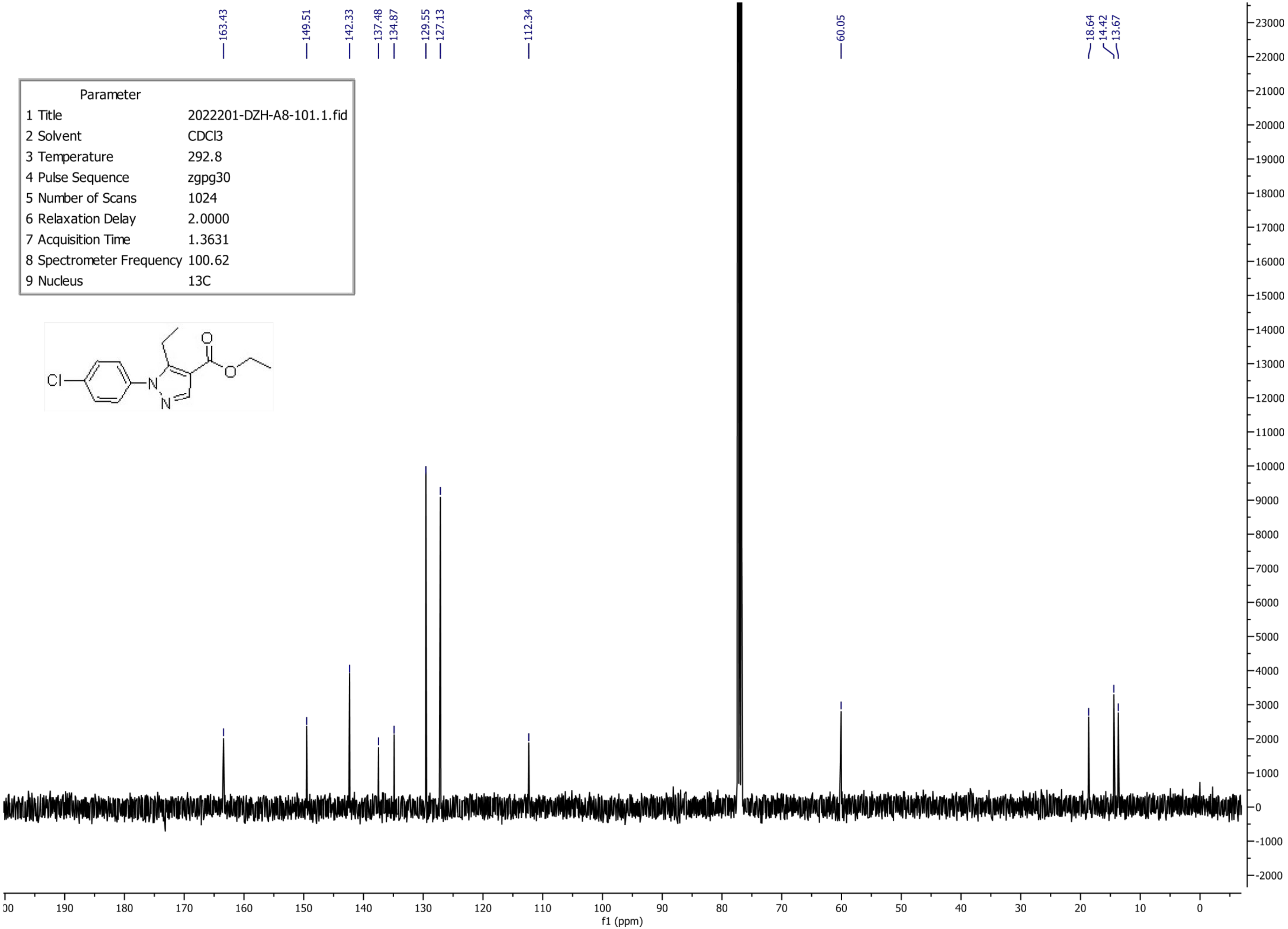

Figure S84 ^{13}C NMR spectrum of compound **37** ($CDCl_3$, 100 MHz)

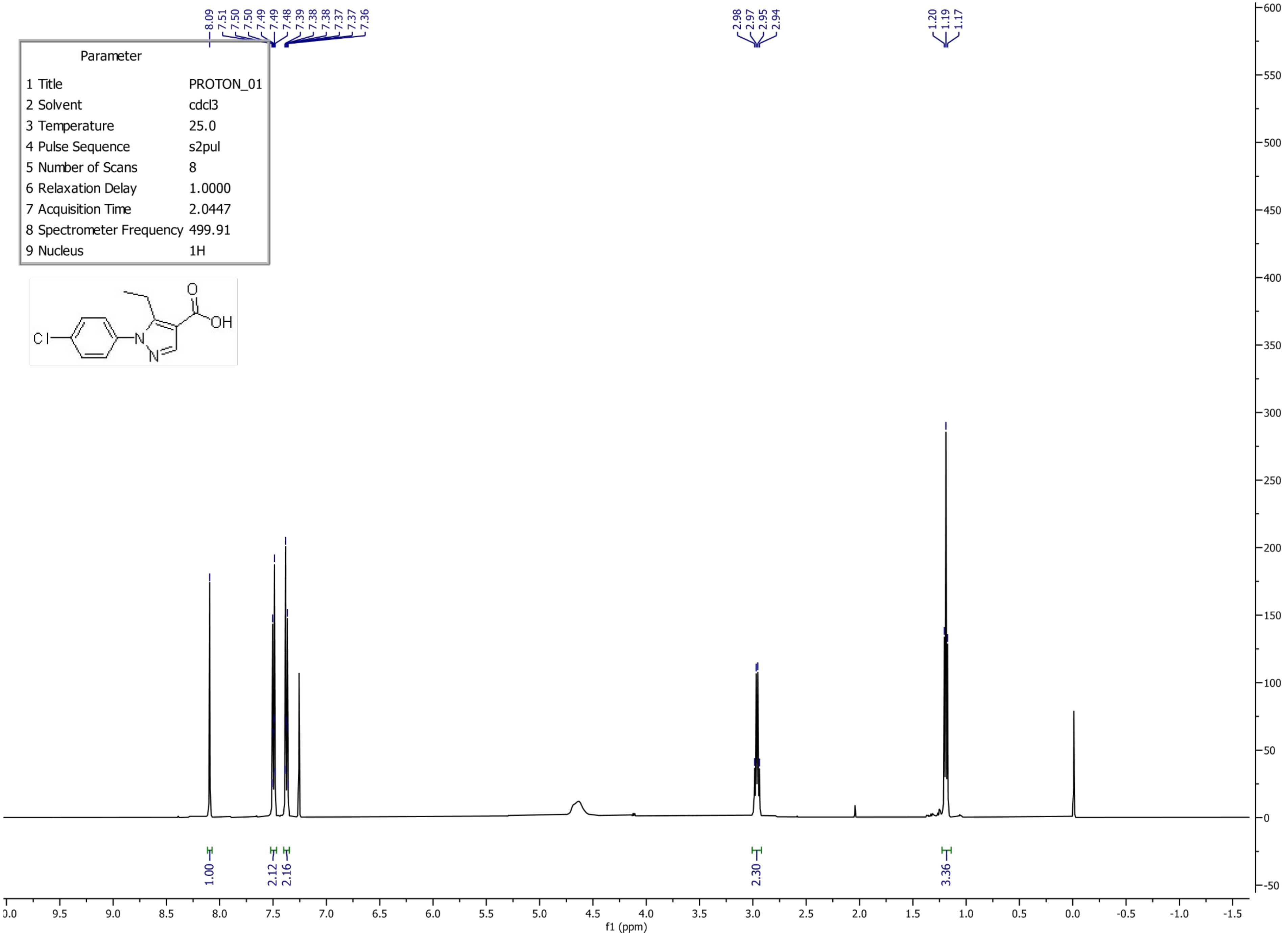

Figure S85 ^{1}H NMR spectrum of compound **38** ($CDCl_3$, 500 MHz)

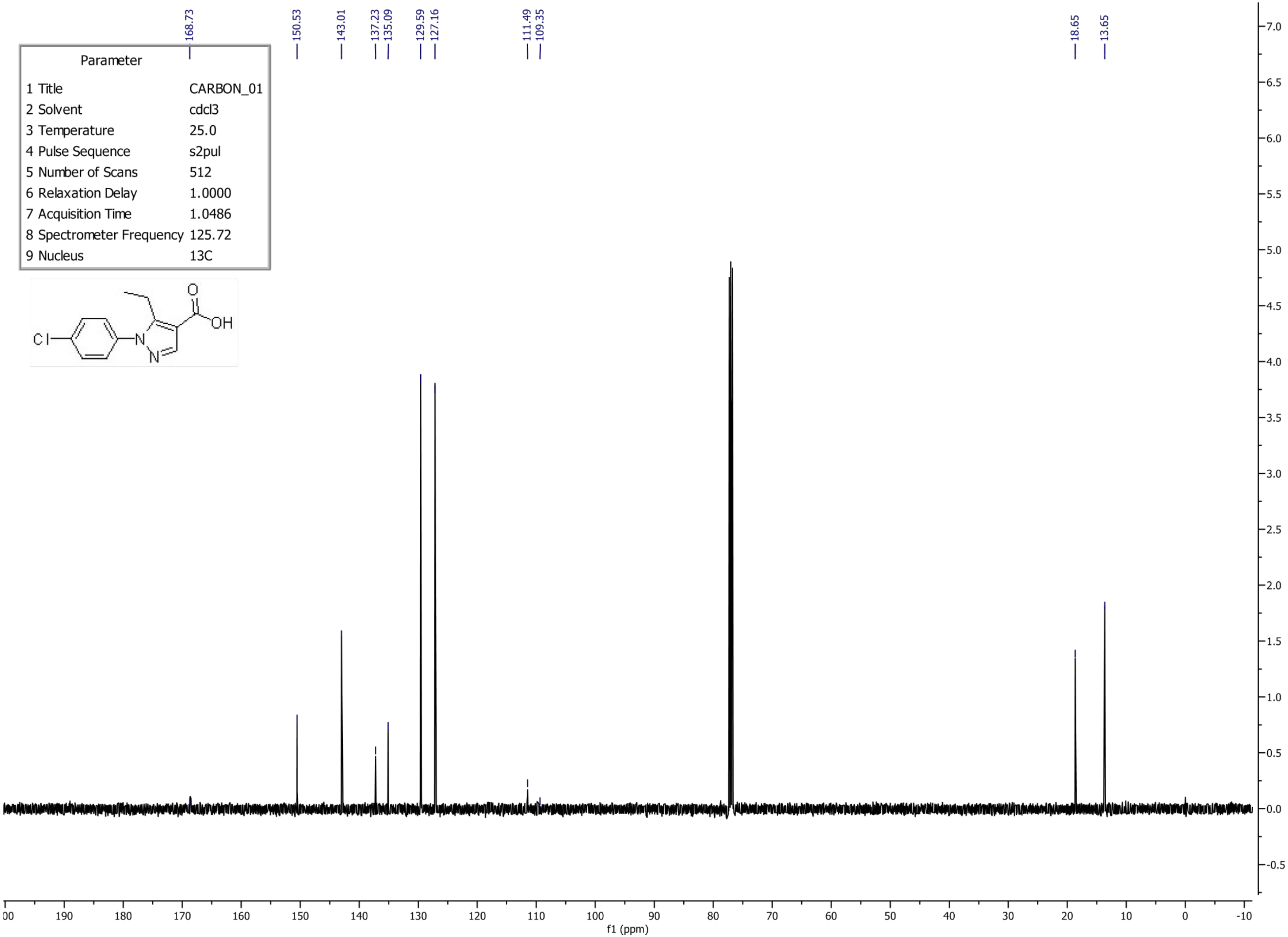

Figure S86 ^{13}C NMR spectrum of compound **38** ($CDCl_3$, 126 MHz)

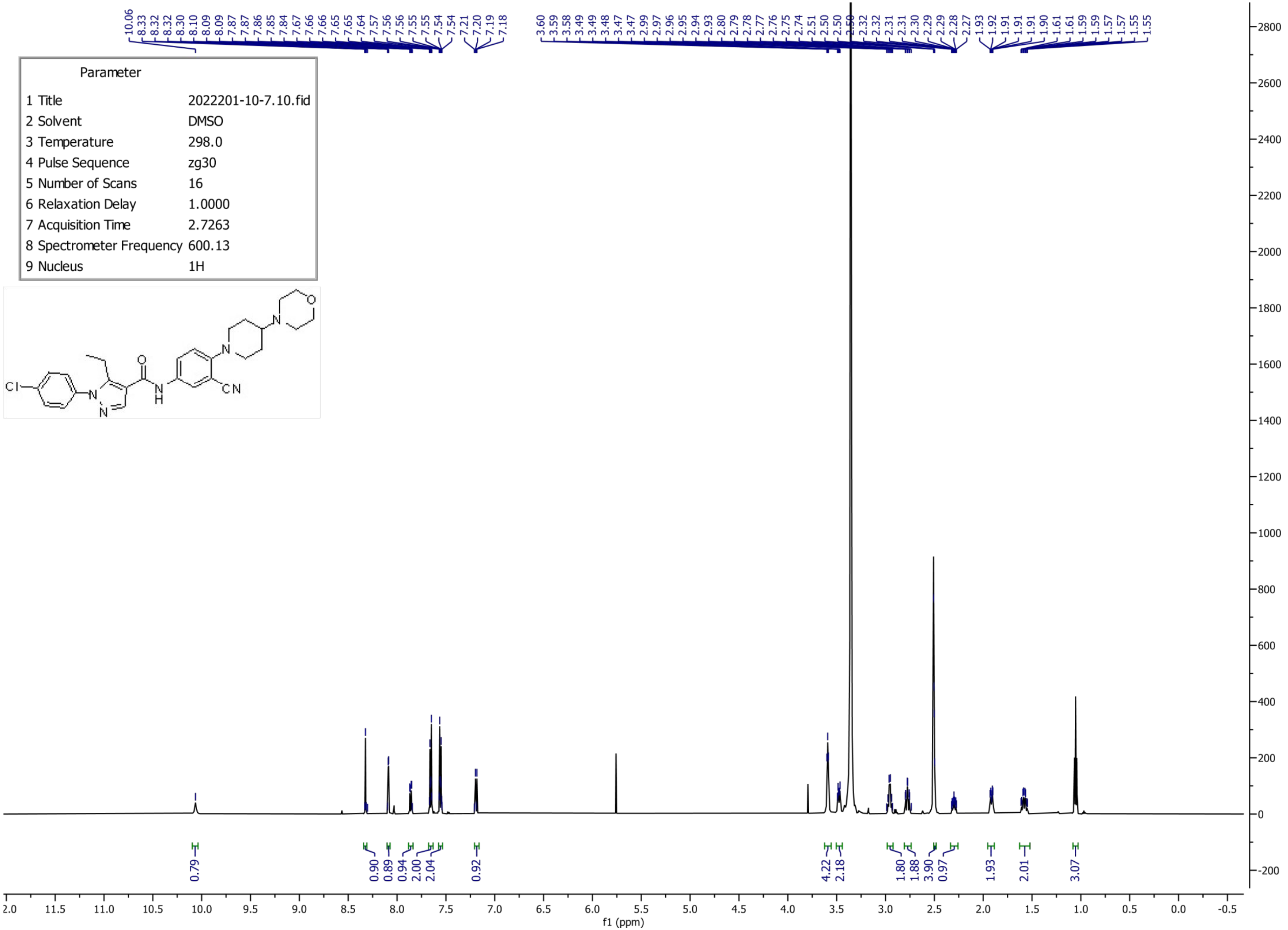

Figure S87 ^{1}H NMR spectrum of compound **39 (BY-019)** (DMSO-d6, 600 MHz)

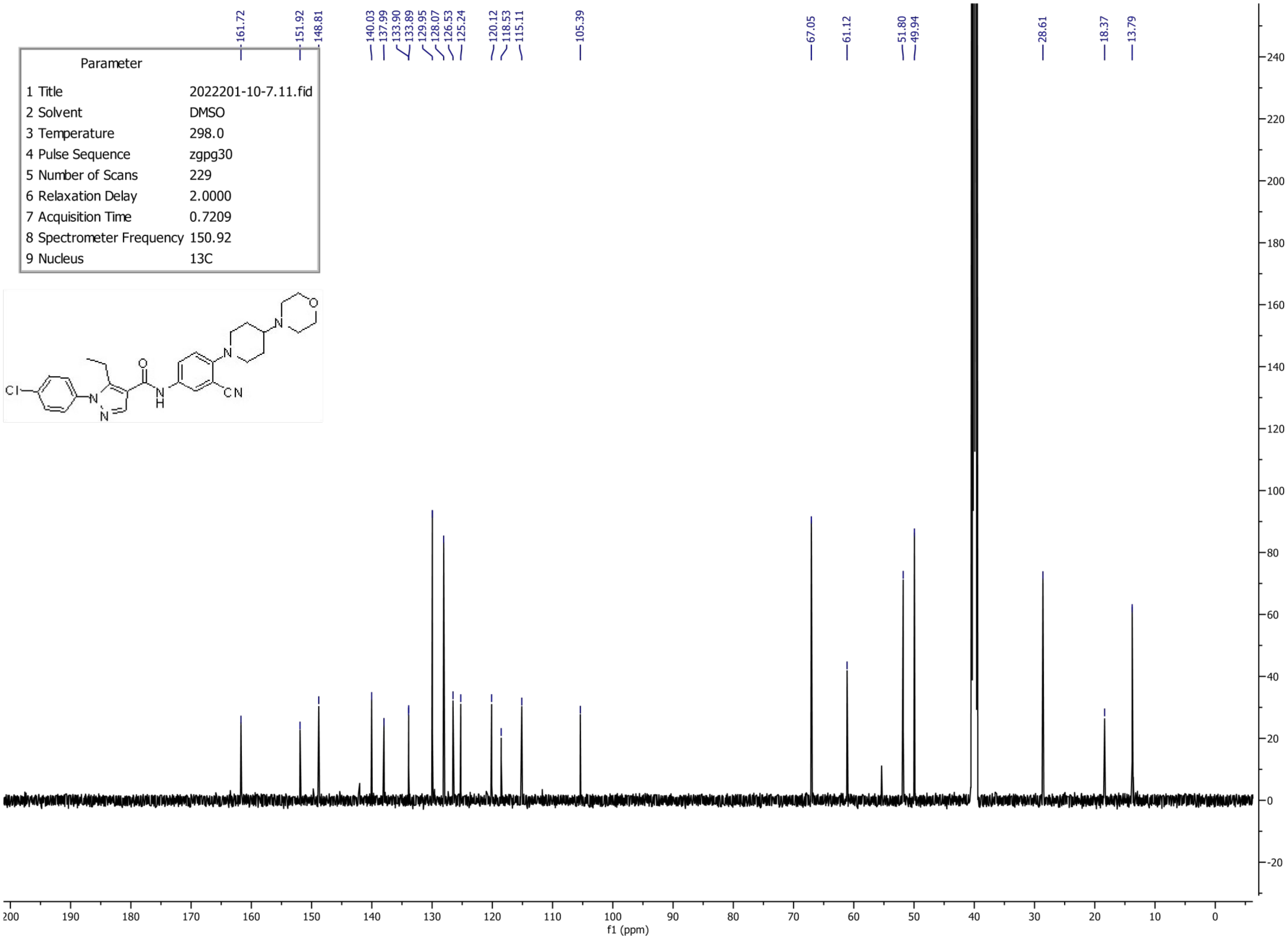

Figure S88 ^{13}C NMR spectrum of compound **39 (BY-019)** (DMSO-d6, 126 MHz)

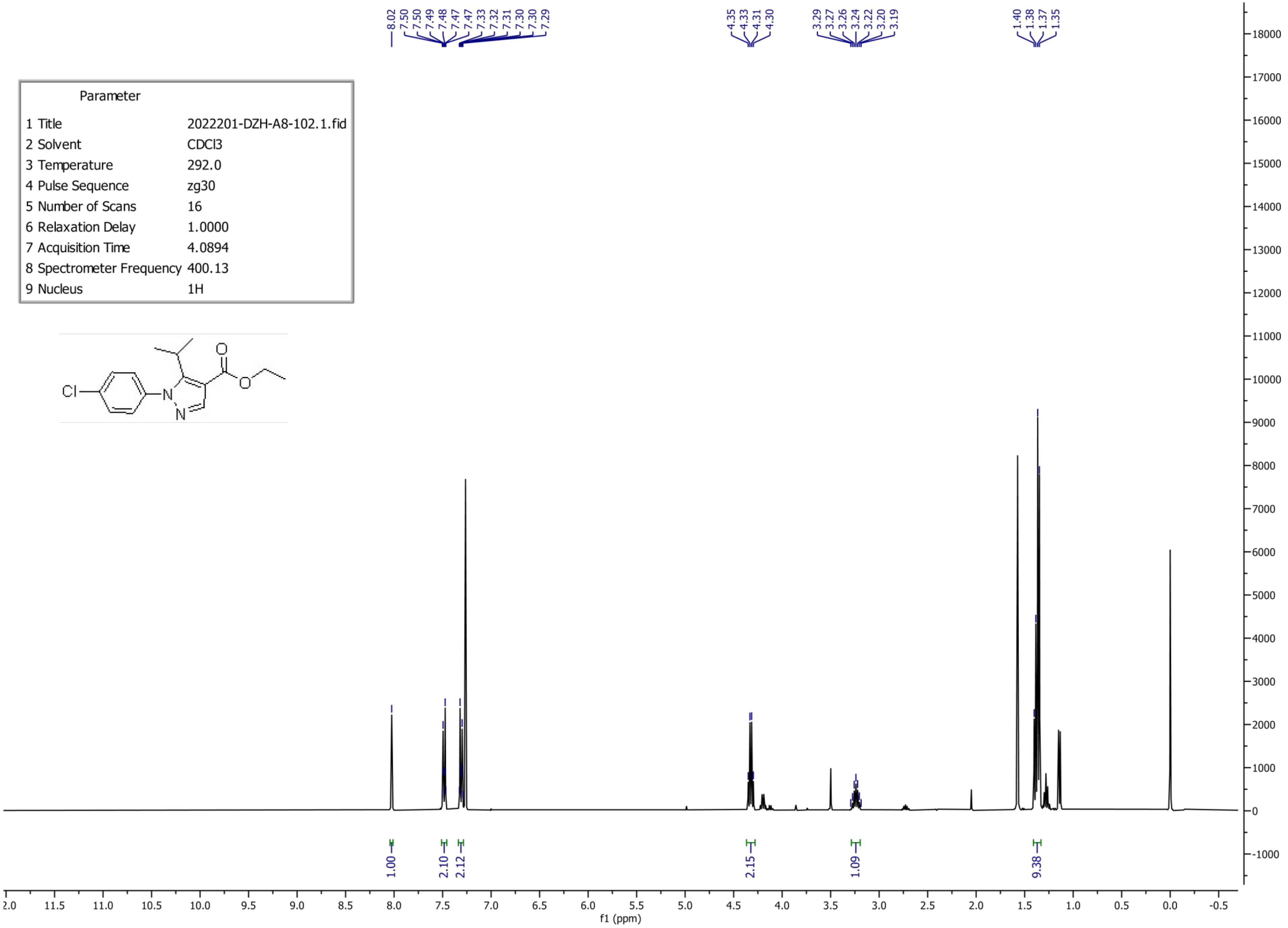

Figure S89 ^{1}H NMR spectrum of compound **40** ($CDCl_3$, 400 MHz)

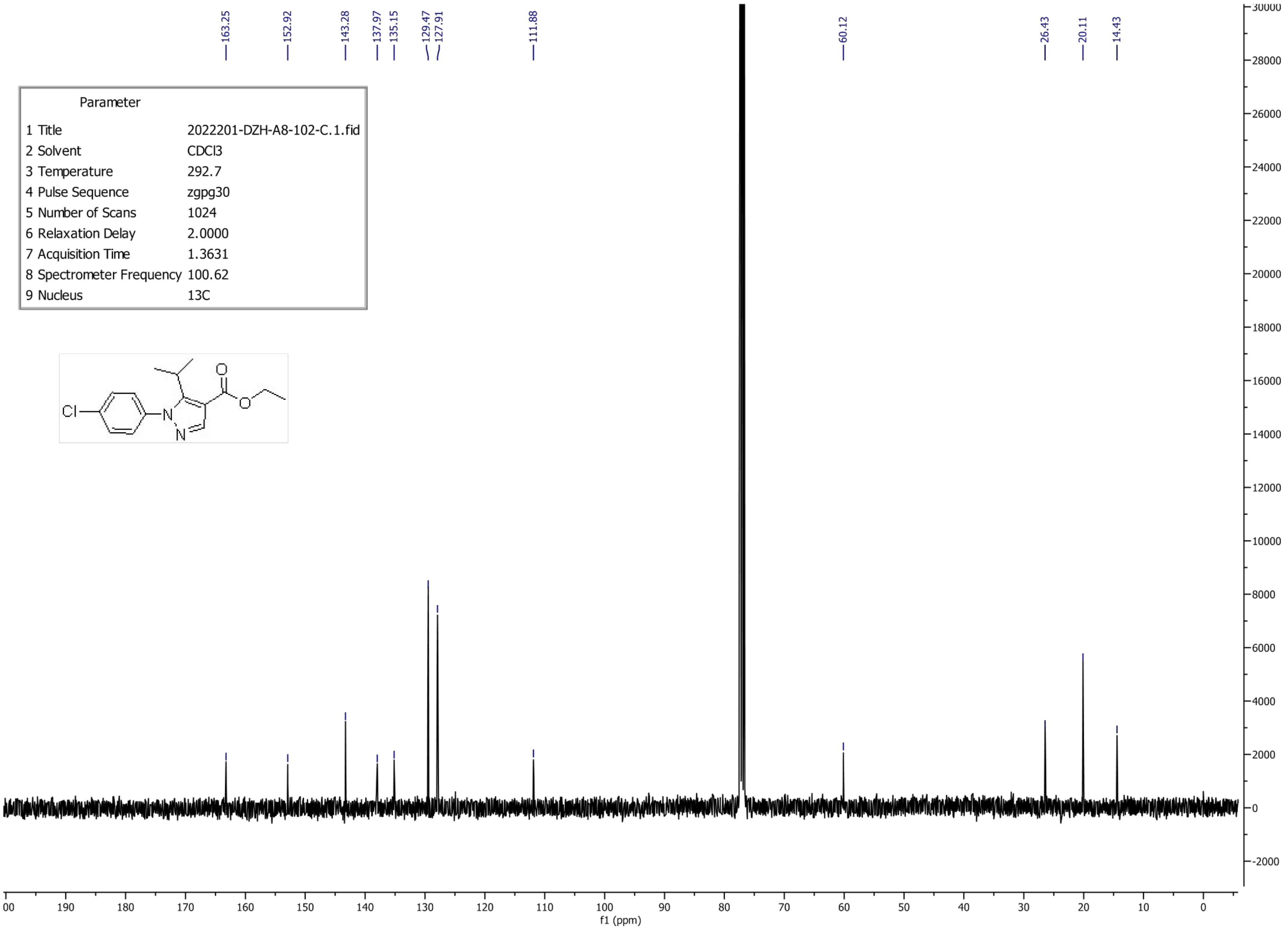

Figure S90 ^{13}C NMR spectrum of compound **40** ($CDCl_3$, 100 MHz)

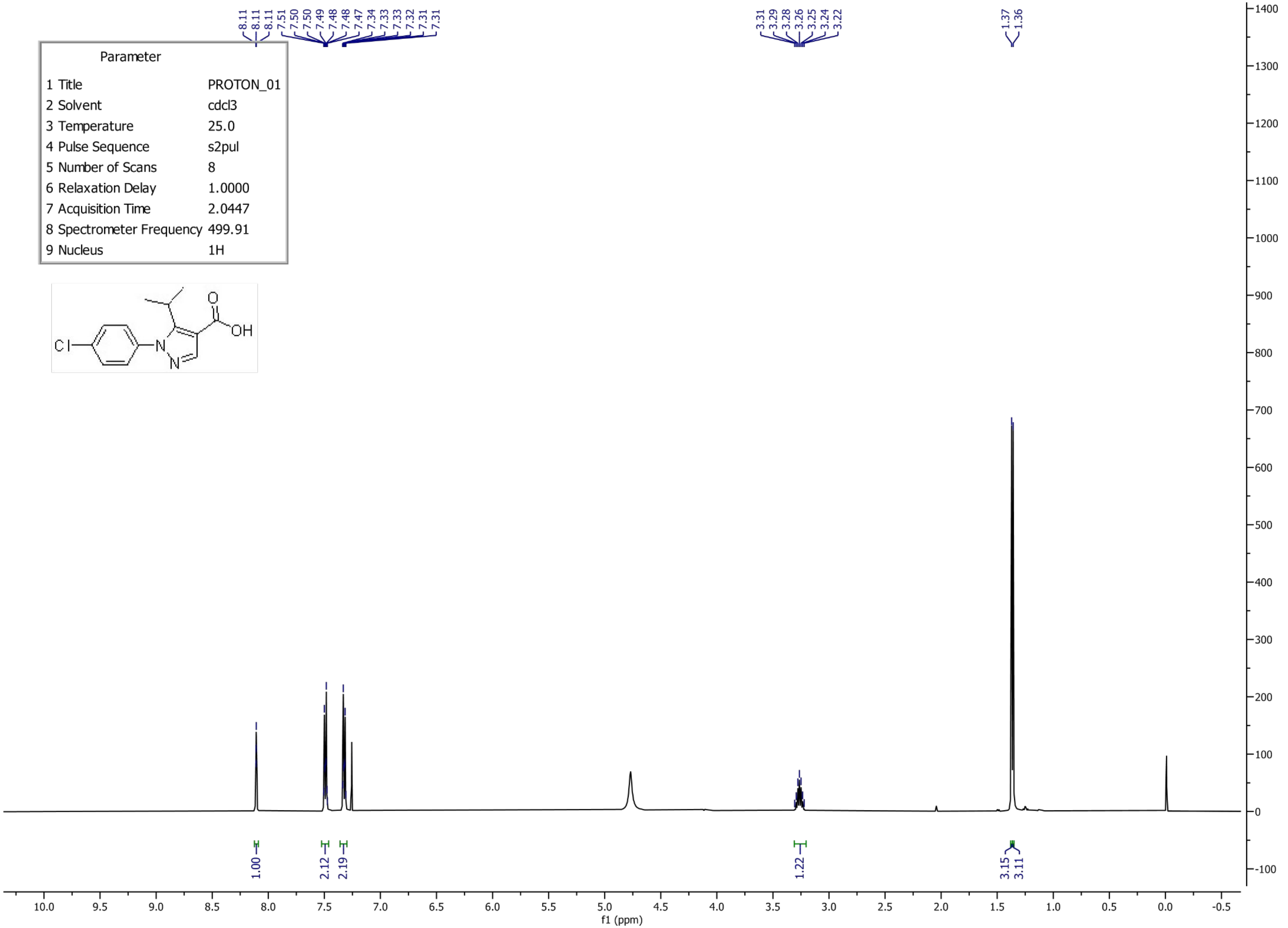

Figure S91 ^{1}H NMR spectrum of compound **41** ($CDCl_3$, 500 MHz)

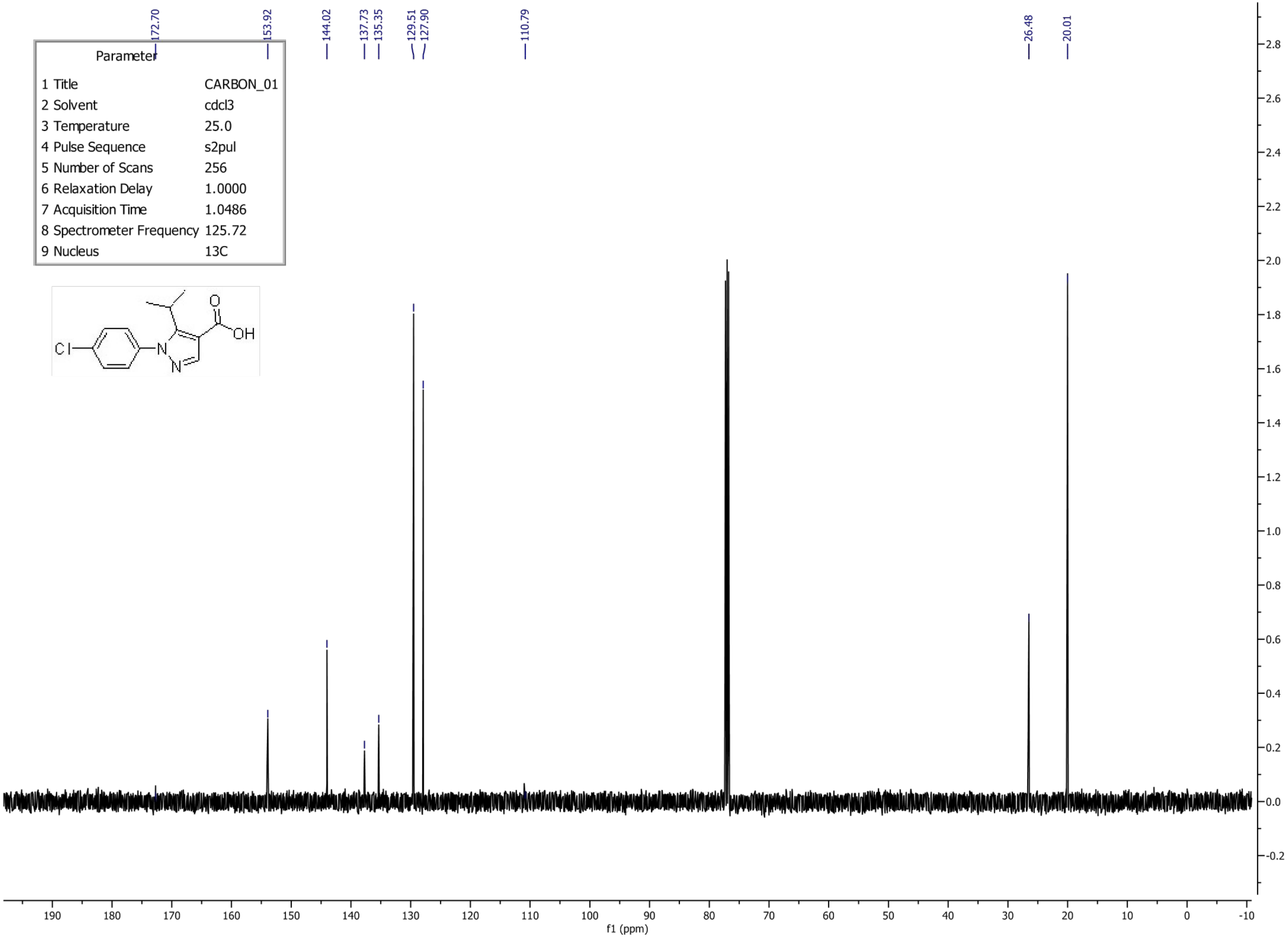

Figure S92 ^{13}C NMR spectrum of compound **41** ($CDCl_3$, 126 MHz)

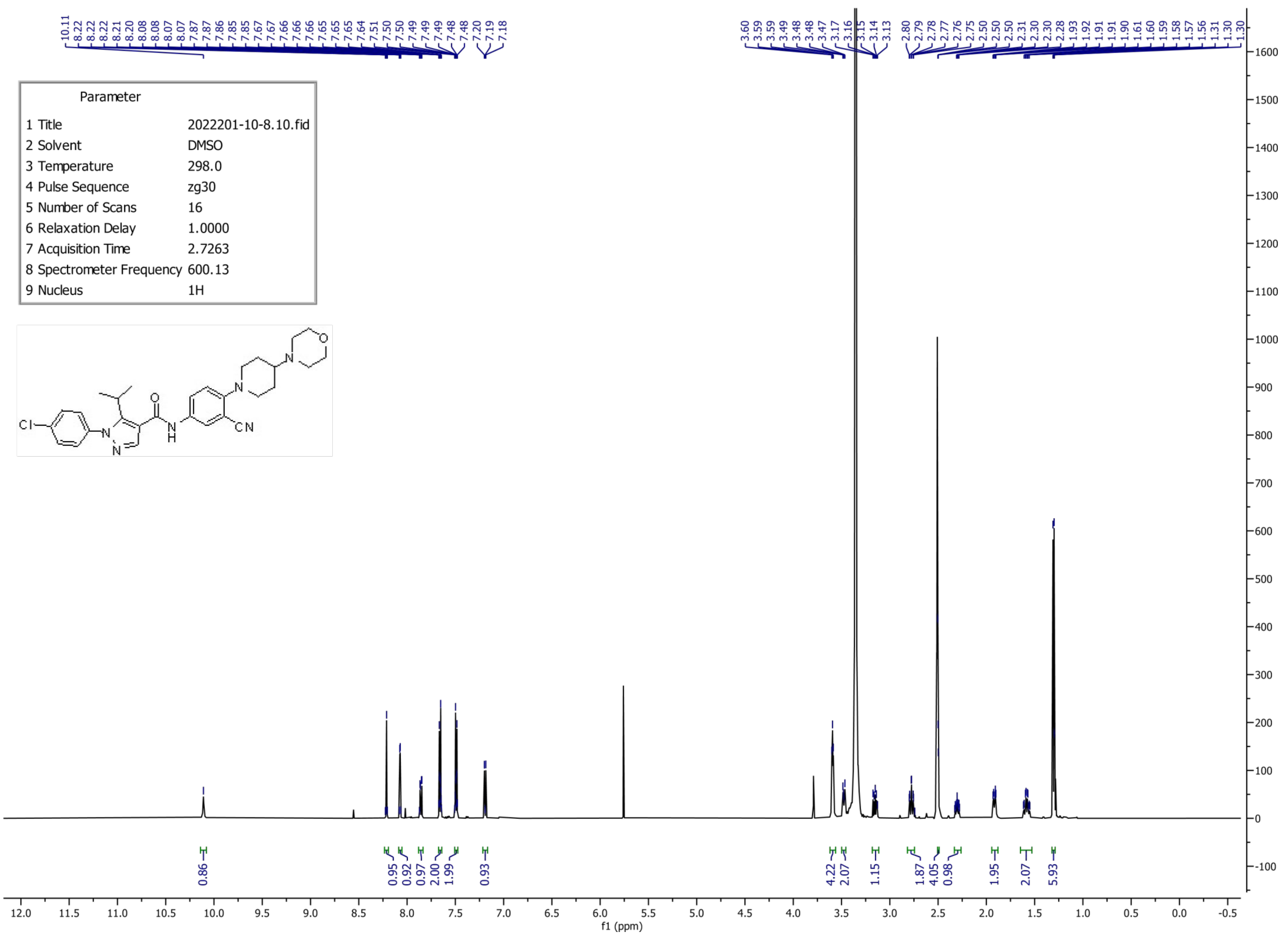

Figure S93 ^{1}H NMR spectrum of compound **42 (BY-024)** (DMSO-d6, 600 MHz)

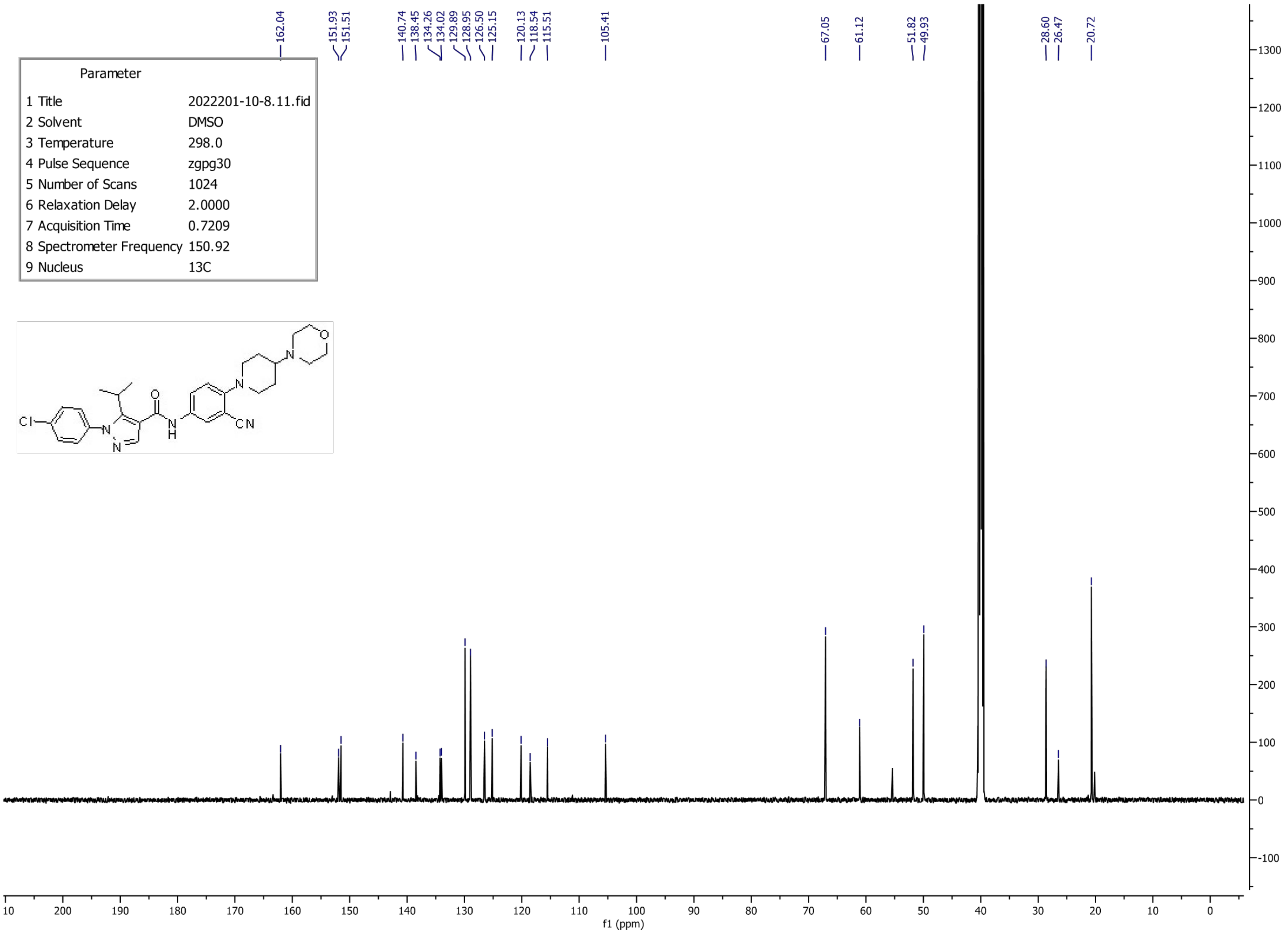

Figure S94 ^{13}C NMR spectrum of compound **42 (BY-024)** (DMSO-d6, 126 MHz)
