# Supplementary material for: A Potent Oral Sialylation Inhibitor Augments the Immunotherapy in Pancreatic Ductal Adenocarcinoma
Source: ACS Cent Sci. 2025 Sep 3;11(10):1969–83. doi: 10.1021/acscentsci.5c00939 (PMC12550625; doi:10.1021/acscentsci.5c00939)
Supplement: Supplementary file 2 [file oc5c00939_si_002.pdf]

oc-2025-00939t.R1

Name: Peer Review Information for "A potent oral sialylation inhibitor augments the immunotherapy in pancreatic ductal adenocarcinoma"

First Round of Reviewer Comments

Reviewer: 1

Comments to the Author

This manuscript, titled "A potent oral sialyltransferase inhibitor augments the immunotherapy in pancreatic ductal adenocarcinoma" presents a highly impressive and timely study that addresses an important challenge in cancer immunotherapy through the regulation of cell surface glycans.

Y-320 is positioned as the first potent and orally available pan-sialyltransferase inhibitor, exhibiting strong inhibitory activity against both α2,3- and α2,6-sialylation. Its cellular IC_{50} (~200 nM) is 300–500 times more potent than the well-known benchmark inhibitor P-3Fax-Neu5Ac. Mechanistic studies, including SAR and binding assays, convincingly demonstrate its mode of action through competitive inhibition of multiple sialyltransferases. The determination of the association rate constant, dissociation rate constant, and equilibrium dissociation constant of Y-320 for ST3Gal1 and ST6Gal1 by SPR represents valuable data that will serve as a useful reference for the future development of sialyltransferase inhibitors.

Moreover, the manuscript convincingly demonstrates that Y-320 modulates the immunosuppressive tumor microenvironment in PDAC, activating $CD8^+$ T cells and NK cells while suppressing immunosuppressive M2 macrophages. Particularly noteworthy is the synergistic effect observed when Y-320 is combined with anti-PD-1 therapy, suggesting its strong potential for overcoming immune checkpoint blockade resistance in pancreatic ductal adenocarcinoma.

While the study does not directly assess Siglec signaling, the observed desialylation of tumor surfaces and enhancement of cytotoxic immune responses are consistent with a disruption of Siglec-mediated immune suppression. The manuscript does not explore regulatory T cells or immunosuppressive cytokines such as IL-10 or TGF-β; however, the dominant mechanism of action appears to rely on $CD8^+$ T cell and macrophage-mediated immunomodulation. It is hoped that these aspects may be addressed in future studies.

Overall, this study provides strong evidence that sialyltransferase inhibition is a viable strategy for modulating the glycoimmune checkpoint, with strong translational potential given the oral bioavailability, synthetic accessibility, and immune-activating effects of Y-320. The quality of the study is extremely high, and it is very much in line with the interdisciplinary and impactful nature expected of ACS Central Science. I believe the manuscript is suitable for publication with minimal revisions.

In the abstract:

Mechanistic studies establish that Y-320's therapeutic efficacy requires the coordinately engagement between CD8+ T cell and macrophage.

" the coordinated engagement" seems to be better.

The fitted curve in the SPR plot appears too bold, which obscures the experimental data traces. It would be helpful to reduce the line thickness for better clarity.

Reviewer: 2

Comments to the Author

This is an interesting report that describes the identification and characterization of a potent, orally available pan-sialyltransferase inhibitor (Y-320), which exhibits anti-cancer activity by stimulating anti-tumor immune cell responses. Remarkably, Y-320 is a pan-sialyltransferase inhibitor unrelated in structure to the donor or acceptor substrates, making it the first of a novel class of sialyltransferase inhibitors.

The data very nicely document that Y-320 is a pan sialyltransferase inhibitor, and remarkably can be given orally to suppress growth of cancer in several tumor models. Moreover, it is shown that the tumor control is mediated by enhanced immune responses of several immune cell types in experiments depleting animals of specific cell types involved in innate and adaptive immune responses. Finally, it shows clear synergy with anti-PD1 to suppress tumor growth.

Overall, the manuscript is well organized, and the data are complete and convincing. There are a few points that could be addressed to strengthen the conclusions and provide a more balanced interpretation of the findings.

Specific comments:

1. The abstract ends by stating that Y-320 is a promising clinical candidate. The phrase 'clinical candidate' has a very specific meaning in the pharmaceutical industry; it means that it has been selected by a pharma company to move forward for clinical trials. I suggest that a different wording be used such as 'shows promise for use as a therapeutic agent for cancer'.

2. Page 9 line 4: binded should be bound.

3. While the paper is well-written and the language is clear, the grammar is occasionally awkward. Recommend it be carefully reviewed for English grammar, even by ChatGPT or other AI language. While the latter may be a controversial suggestion, if the prompt includes fixing grammar without altering any scientific language, content, or flow, there will be minimal changes.

4. Results in Figure 4 show that the avidity of Y-320 is impacted by substitutions at either end, and that neither the 'left' nor 'right' fragments exhibit inhibition. Molecular modeling suggests the molecule binds to the catalytic site of one of the sialyltransferases. While this data nicely shows molecular features involved in the inhibition, they do not

describe the 'mechanism of action'. Can another phrase be used in the Figure legend and heading in the main text.

5. Missing are simple enzyme kinetics with one more sialyltransferases to determine if Y-230 is competitive with CMP-NeuAc, the acceptor substrate or both. While the docking suggests Y-320 binds to the catalytic site, kinetics or crystal structure would be needed to better characterize the binding mode of Y-320 as a pan-sialyltransferase inhibitor.

6. Data showing that treatment with Y-320 increase cytokine expression and activation of various immune cell types nicely demonstrate that it has an impact on immune cell responses. As for innate and adaptive immune responses, there are no data looking at antigen-specific B or T cell responses, or innate immune responses. Rather, the data is generated for immune cell types associated with adaptive and innate immune responses. It is sufficient to say that Y-320 alters immune cell responses, or that Y-320 alters immune responses of immune cells associated with innate and adaptive immunity.

7. Have other normal tissues been evaluated for desialylation by Y-320? This is relevant to the potential use of Y-320 in patients.

8. The authors demonstrate that Y-320 is a more potent pan-sialyltransferase inhibitor than the previously described 3axF-NeuAc. This sialyltransferase inhibitor induced profound kidney damage in mice after only one week, and kidney failure in one month, likely due to suppression of NeuAcα2-8NeuAc sialylation on the glomerulus protein podocalyxin. The fact that the Y-320 mice showed no weight loss or gain over 15 days is encouraging that Y-320 has less toxicity to the kidneys. How long was treatment with Y-320 followed? Was any toxicity seen at longer times? If not, this is a significant positive and should be commented on.

Author's Response to Peer Review Comments:

Response to Reviewer Comments

We sincerely appreciate the reviewers' valuable comments, which indeed help us to improve our manuscript and to consider following-up studies. Below are our point-by-point responses to the reviewers' concerns; the corresponding revisions in the manuscript are highlighted in yellow for easy reference.

Reviewer #1:

Comment 1: While the study does not directly assess Siglec signaling, the observed desialylation of tumor surfaces and enhancement of cytotoxic immune responses are consistent with a disruption of Siglec-mediated immune suppression. The manuscript does not explore regulatory T cells or immunosuppressive cytokines such as IL-10 or TGF-β; however, the dominant mechanism of action appears to rely on $CD8^{+}$ T cell and macrophage-mediated immunomodulation. It is hoped that these aspects may be addressed in future studies.

Response: Thank you for the valuable suggestion. We fully agree on the importance of clearly identification of the impact of Y-320 on the tumor immune microenvironment. As mentioned, Siglec signaling has been shown to be involved in the functional regulation of CD8+ T cells and macrophages, which has been verified by our immune cell deletion experiments, flow cytometry immunophenotyping, and in vitro cell model data. Indeed, in recent years, other immune cells in the complex tumor immune microenvironment, such as Tregs and B cells, have been reported to be regulated by Siglec signaling, suggesting that we need to pay attention to the effects of Y-320 on the functions of these cells in the future.

Following the review suggestion, we have added the following discussion in the manuscript (Page 15, Lines 10-14): Recent advances in research on the regulatory role of Siglec signaling in immune cells, such as Tregs and B cells, within the tumor microenvironment suggest that we need to conduct more in-depth studies in the future to evaluate the effects of Y-320 on various players in the tumor immune microenvironment, as well as on specific innate and adaptive immune responses.

Ref:

1. Perdicchio M, Ilarregui JM, Verstege MI, et al. Sialic acid-modified antigens impose tolerance via inhibition of T-cell proliferation and de novo induction of regulatory T cells. Proc Natl Acad Sci U S A. 2016; 113(12): 3329-3334. doi:10.1073/pnas.1507706113

2. Özgör L, Meyer SJ, Korn M, Terörde K, Nitschke L. Sialic acid ligand binding of CD22 and Siglec-G determines distinct B dell functions but is dispensable for B cell tolerance induction. J Immunol. 2018; 201(7): 2107-2116. doi:10.4049/jimmunol.1800296

Comment 2: In the abstract: Mechanistic studies establish that Y-320's therapeutic efficacy requires the coordinately engagement between CD8+ T cell and macrophage.

"the coordinated engagement" seems to be better.

Response: Thank you for the suggestion. This has been revised in the revised manuscript (Page 2, Line 12).

Comment 3: The fitted curve in the SPR plot appears too bold, which obscures the experimental data traces. It would be helpful to reduce the line thickness for better clarity.

Response: Thank you for the suggestion. We have reduced the line thickness of the SPR plot (Figure 3F, Figure S6C).

Reviewer #2:

Comment 1: The abstract ends by stating that Y-320 is a promising clinical candidate. The phrase ‘clinical candidate’ has a very specific meaning in the pharmaceutical industry; it means that it has been selected by a pharma company to move forward for clinical trials. I suggest that a different wording be used such as ‘shows promise for use as a therapeutic agent for cancer’.

Response: Thank you for the suggestion. This has been revised in the revised manuscript (Page 2, Lines 15-16).

Comment 2: Page 9 line 4: binded should be bound.

Response: Thank you for the comment. This has been corrected in the revised manuscript (Page 8, Line 26).

Comment 3: While the paper is well-written and the language is clear, the grammar is occasionally awkward. Recommend it be carefully reviewed for English grammar, even by ChatGPT or other AI language. While the latter may be a controversial suggestion, if the prompt includes fixing grammar without altering any scientific language, content, or flow, there will be minimal changes.

Response: As suggested by the reviewer, we have corrected grammatical errors as much as possible. (Page 2, Line 5; Page 2, Line 12; Page 3, Line 16; Page 4, Line 8; Page 4, Line 22; Page 8, Line 3; Page 8, Line 4; Page 8, Line 9; Page 9, Line 14; Page 19, Line 27; Page 31, Line 4)

Comment 4: Results in Figure 4 show that the avidity of Y-320 is impacted by substitutions at either end, and that neither the 'left' nor 'right' fragments exhibit inhibition. Molecular modeling suggests the molecule binds to the catalytic site of one of the sialyltransferases. While this data nicely shows molecular features involved in the inhibition, they do not describe the 'mechanism of action'. Can another phrase be used in the Figure legend and heading in the main text.

Response: Thank you for the suggestion. We have replaced 'mechanism of action' with 'mode of action' in the revised manuscript (Page 9, Line 16; Page 32, Line 19).

Comment 5: Missing are simple enzyme kinetics with one more sialyltransferases to determine if Y-320 is competitive with CMP-NeuAc, the acceptor substrate or both. While the docking suggests Y-320 binds to the catalytic site, kinetics or crystal structure would be needed to better characterize the binding mode of Y-320 as a pan-sialyltransferase inhibitor.

Response: We fully agree with the reviewer's insightful suggestion that additional evidence, such as enzyme activity assays, enzyme kinetics, and co-crystal structure analysis, would further strengthen the present conclusion that Y-320 functions as a sialyltransferase inhibitor. These experiments are critical for a comprehensive mechanistic understanding, and we are actively pursuing these challenging studies and wish to complish these tasks in our future work.

In accordance with the reviewer's comments, we have revised some insufficiently rigorous descriptions regarding Y-320's characterization as a sialyltransferase inhibitor throughout the manuscript to ensure more precise presentation (Page 1, Line 1; Page 2, Line 16; Page 4, Line 15; Page 4, Line 27; Page 10, Line 20; Page 13, Line 20; Page 15, Line 21).

In the present study, we primarily focused on elucidating the biological mechanism by which Y-320 potently reduces cellular and tissue sialylation and subsequently activates immune responses to exert anti-tumor effects. While our current findings strongly support Y-320's functional role in modulating sialylation-dependent immune regulation, we acknowledge that the structural and enzymatic characterization proposed by the reviewer

would provide deeper mechanistic insights. We sincerely appreciate this valuable suggestion and will incorporate these experiments in our ongoing investigations.

Comment 6: Data showing that treatment with Y-320 increase cytokine expression and activation of various immune cell types nicely demonstrate that it has an impact on immune cell responses. As for innate and adaptive immune responses, there are no data looking at antigen-specific B or T cell responses, or innate immune responses. Rather, the data is generated for immune cell types associated with adaptive and innate immune responses. It is sufficient to say that Y-320 alters immune cell responses, or that Y-320 alters immune responses of immune cells associated with innate and adaptive immunity.

Response: Thank you for this insightful suggestion. We fully agree on the importance of clearly identifying the impact of Y-320 on the innate and adaptive immunity. In our manuscript, through immune cell deletion experiment, flow cytometry immunophenotyping, and in vitro cell model data, we have validated the important role of CD8+ T cells and macrophages in the tumor-suppressive effects of Y-320, consistent with recent advances in Siglec signaling research. Indeed, in recent years, other immune cells in the complex tumor immune microenvironment, such as Tregs and B cells, have been reported to be regulated by Siglec signaling, suggesting the need to further investigate the effects of Y-320 on the functions of these cells in the future.

Based on these suggestions, we revised the imprecise wording and replaced the original statement in the legend of Figure 5, 'Y-320 induces both innate and adaptive immune responses for anti-tumor effect,' with 'Y-320 induces anti-tumor effects in CD8+ T cells and macrophages.' In addition, we have added the following discussion in the manuscript (Page 15, Lines 10-14): Recent advances in research on the regulatory role of Siglec signaling in immune cells, such as Tregs and B cells, within the tumor microenvironment suggest that we need to conduct more in-depth studies in the future to evaluate the effects of Y-320 on various players in the tumor immune microenvironment, as well as on specific innate and adaptive immune responses.

Ref:

1. Perdicchio M, Ilarregui JM, Verstege MI, et al. Sialic acid-modified antigens impose tolerance via inhibition of T-cell proliferation and de novo induction of regulatory T cells. Proc Natl Acad Sci U S A. 2016; 113(12): 3329-3334. doi:10.1073/pnas.1507706113

2. Özgör L, Meyer SJ, Korn M, Terörde K, Nitschke L. Sialic acid ligand binding of CD22 and Siglec-G determines distinct B cell functions but is fispensable for B cell tolerance induction. J Immunol. 2018; 201(7): 2107-2116. doi:10.4049/jimmunol.1800296

Comment 7: Have other normal tissues been evaluated for desialylation by Y-320? This is relevant to the potential use of Y-320 in patients.

Response: In our work, Y-320 was administered orally, resulting in systemic exposure. Considering that these parameters mainly affect safety, although we have not analyzed drug distribution and desialylation in other organs, based on body weight data, Y-320 appeared to be tolerated by mice at our administered dose. Besides, Figure 2B shows that the expression of the Y-320 targets in tumors is higher than in normal tissues, which also suggests that Y-320 mainly works in tumors.

Based on the reviewer's suggestions, we will focus on the distribution of Y-320 in major organs and its desialylation effects after administration in our future studies. Additionally, we are considering precise delivery of Y-320 into tumors via antibody-dependent or liposome-dependent methods to enhance the efficacy and safety of Y-320.

Comment 8: The authors demonstrate that Y-320 is a more potent pan-sialyltransferase inhibitor than the previously described 3Fax-NeuAc. This sialyltransferase inhibitor induced profound kidney damage in mice after only one week, and kidney failure in one month, likely due to suppression of NeuAcα2-8NeuAc sialylation on the glomerulus protein podocalyxin. The fact that the Y-320 mice showed no weight loss or gain over 15 days is encouraging that Y-320 has less toxicity to the kidneys. How long was treatment with Y-320 followed? Was any toxicity seen at longer times? If not, this is a significant positive and should be commented on.

Response: We appreciate the reviewer's concern regarding potential kidney toxicity. In our work, the longest administration period for Y-320 was 25 days in the Pan-02 model (Figure 6A). Throughout this period, we did not observe any weight loss in the mice (Figure S9A) at our administered dose, no abnormalities were noted during cage-side observations, and no organ abnormalities were detected during the terminal dissection. Considering the risk of kidney toxicity associated with P-3Fax-Neu5Ac that you mentioned, we additionally measured serum kidney injury indicators UREA and CREA after Y-320 administration and found that Y-320 did not affect the levels of the two indicators, preliminarily suggesting that this dose did not affect kidney function (Figure S10A). In future studies, we need to

systematically evaluate the safety of Y-320, including dose gradient studies and major organ pathology analysis.

Based on the reviewer's suggestions, we have added the following discussion to the manuscript (Page 16, Lines 13-17): Although at the administered dose, Y-320 did not exhibit risks such as renal toxicity similar to that caused by P-3Fax-Neu5Ac, dose-response analysis and major organ pathology analysis still need to be conducted. And targeting delivery to tumors via antibody- or liposome-dependent strategies should be considered to enhance the safety and efficacy of Y-320.

Ref:

1. Macauley MS, Arlian BM, Rillahan CD, et al. Systemic blockade of sialylation in mice with a global inhibitor of sialyltransferases. J Biol Chem. 2014; 289(51): 35149-35158. doi:10.1074/jbc.M114.606517

We hope the revised manuscript meets the journal's standards. Thank you again for your constructive feedback. Please let us know if further modifications are needed.

oc-2025-00939t.R2

Name: Peer Review Information for "A potent oral sialylation inhibitor augments the immunotherapy in pancreatic ductal adenocarcinoma"

Second Round of Reviewer Comments

Reviewer: 2

Comments to the Author

The authors have adequately addressed the majority of the concerns raised by this reviewer. Several suggestions would require additional experiments that are within the scope of the investigations, but would delay the publication of the substantial novel

findings. The authors mention that these points are the subject of future investigations, and have appropriately tempered their conclusions in light of the work that is ongoing.

Reviewer: 1

Comments to the Author

The reviewer believe that the revisions made are sufficient. Glycans, including sialic acid residues, are ubiquitously present in vivo, and the enzymes responsible for their biosynthesis, including sialyltransferases, are distributed in all cells. Under such circumstances, whether glycan biosynthetic enzymes can be viable drug targets without causing adverse effects has been a major challenge for researchers in glycoscience and related fields.

One reviewer pointed out that "This sialyltransferase inhibitor (3Fax-NeuAc) induced profound kidney damage in mice after only one week, and kidney failure in one month, likely due to suppression of NeuAcα2-8NeuAc sialylation on the glomerulus protein podocalyxin.". This is an extremely important point. 3Fax-NeuAc, as a metabolic inhibitor, exhibits IC_{50} values in the range of 10–50 μM, depending on the types of enzymes and cells. To achieve therapeutic efficacy in vivo, relatively high doses of the compound are inevitably required. At such high systemic concentrations, cell-selective uptake of 3Fax-NeuAc is unlikely, and as a result, it is expected to affect glycans that are ubiquitously expressed across many cell types. This likely underlies the toxicity associated with metabolic inhibitors.

In contrast, the authors respond that: “Throughout this period, we did not observe any weight loss in the mice (Figure S9A) at our administered dose, no abnormalities were noted during cage-side observations, and no organ abnormalities were detected during the terminal dissection.”

One possible explanation, from the reviewer’s perspective, is that a glycosyltransferase inhibitor with high binding affinity may exert its effect at significantly lower doses. A potent inhibitor may preferentially act on disease sites where the target glycosyltransferase is overexpressed, thereby achieving organ or tissue selectivity in enzyme-concentration-dependent manner.

In support of this idea, although not conclusive, the authors mention that “Compound Y-320 was identified to inhibit the α-2,3SA and α-2,6SA levels with IC50s around 150–240 nM

in multiple PDAC cells, approximately 300 to 500-fold greater potency than the positive control P-3Fax-Neu5Ac."

The differences in the in vivo behavior, particularly in pharmacokinetics, between metabolic inhibitors and potent inhibitors of glycosyltransferases remain largely unrecognized, even among many researchers in the glycoscience field. Although the pharmacokinetics of compound Y-320 are not addressed in this study, such evaluations are generally conducted during the non-clinical stages of drug development and are not necessarily expected at this point. Rather, the reviewer supports the prompt publication of this work.

The present study is highly valued because it provides evidence suggesting therapeutic efficacy with reduced toxicity, which cannot be achieved using metabolic inhibitors. It thus represents an important contribution demonstrating that glycosyltransferases can indeed be druggable targets.

Author's Response to Peer Review Comments:

Dear editor:

We have selected one cover art for consideration.

Thank you for your help.
